# Supplementary material for: Structure Activity of β-Amidomethyl Vinyl Sulfones as Covalent Inhibitors of Chikungunya nsP2 Cysteine Protease with Anti-alphavirus Activity
Source: bioRxiv. 2024 Jun 13:2024.06.12.598722. Preprint. [Version 2] doi: 10.1101/2024.06.12.598722 (PMC11195264; doi:10.1101/2024.06.12.598722)
Supplement: Supplement 1 [file media-1.pdf]

Supporting Information

Structure Activity of β-Amidomethyl Vinyl Sulfones as Covalent Inhibitors of *Chikungunya* nsP2 Cysteine Protease with Anti-alphavirus Activity

Anirban Ghoshal[†,#], Kesatebrhan Haile Asressu[†,#], Mohammad Anwar Hossain[†,#], Peter J. Brown[†,#] , Eric M. Merten[‡,#], John D. Sears[§,#], Sumera Perveen[‖], Kenneth H. Pearce[‡,#], Konstantin I. Popov[‡,#], Nathaniel J. Moorman[§,#], Mark T. Heise[⊥,§,#], and Timothy M. Willson[†,#,*]

[†]Structural Genomics Consortium, UNC Eshelman School of Pharmacy, University of North Carolina at Chapel Hill, Chapel Hill, NC 27599, USA.

[#]READDI AViDD Center, University of North Carolina at Chapel Hill, Chapel Hill, NC 27599, USA.

[‡]UNC Eshelman School of Pharmacy, Center for Integrative Chemical Biology and Drug Discovery, University of North Carolina at Chapel Hill, Chapel Hill, NC 27599, USA

[§]Department of Microbiology and Immunology, University of North Carolina at Chapel Hill, Chapel Hill, NC, 27599, USA

[‖]Structural Genomics Consortium, University of Toronto, Toronto, Ontario, M5G 1L7, Canada

[⊥]Department of Genetics, University of North Carolina at Chapel Hill, Chapel Hill, NC, 27599, USA

*Correspondence: tim.willson@unc.edu

Table of Contents		Pages
Table S1	Binding kinetics of covalent nsP2 protease inhibitors	S2
Table S2	VEEV-nLuc pEC_{50} for compounds with pEC_{50} >6 on CHIKV-nLuc	S3
Figure S1	DLS data for compound **1a**	S4
Figures S2–S153	NMR spectra of final compounds	S5–S80
Figures S154–S206	HPLC analysis of pyrazole analogs	S81–S95
Figures S207–S208	Chiral SFC of (*R*)-**24a** and (*S*)-**24a**	S96–S97

Table S1. Binding kinetics of covalent nsP2 protease inhibitors

Binding Kinetics Assay. CHICKV nsP2 protease activity was measured using an internally quenched peptide substrate continuously over 45 min as previously described.[1] 2-fold serial-dilutions of inhibitor were used to measure k_{obs} values and the data were fit to the equation (k_{obs} = (k_{inact} x [I]) / (K_i + [I]) to determine the potency of the initial binding event (K_i) and maximum potential rate of covalent bond formation (k_{inact}).

**Compound**	**IC_{50} (μM)**	**K_i (μM)**	**k_{inact}/K_i ($M^{-1}s^{-1}$)**
**1a**	0.06	0.28	6400
**1b**	0.13	1.9	1500
**1c**	0.08	0.64	2600
**1e**	0.07	0.30	3600
**1g**	0.06	1.5	2000
**1h**	0.04	0.20	4500
**1k**	0.18	0.29	3300
**1l**	0.17	0.79	1400
**1o**	0.03	0.11	9100
**4c**	0.10	0.80	1500
**4d**	0.05	0.25	5900
**4f**	0.10	1.7	1000
**8d**	0.02	0.06	12000
**10**	0.04	0.29	6000
**14**	0.15	0.46	1900
**20**	1.4	16	100
**24a**	0.13	0.30	1500
**25d**	0.15	0.86	1900

(1) Merten, E. M.; Sears, J. D.; Leisner, T. M.; Hardy, P. B.; Ghoshal, A.; Hossain, M. A.; Asressu, K. H.; Brown, P. J.; Stashko, M. A.; Herring, L. E.; et al. Discovery of a cell-active chikungunya virus nsP2 protease inhibitor using a covalent fragment-based screening approach. *bioRxiv* **2024**. DOI: 10.1101/2024.03.22.586341.

Table S2. VEEV-nLuc pEC_{50} for compounds with pEC_{50} >6 on CHIKV-nLuc

Compound	CHIKV-nLuc (pEC_{50})	VEEV-nLuc (pEC_{50})
**1a**	7.4	6.4
**1b**	7.3	5.5
**1c**	7.3	5.9
**1d**	6.1	5.1
**1e**	7.1	6.1
**1f**	6.3	<5
**1g**	7.1	6.0
**1h**	7.0	6.2
**1j**	6.6	6.0
**1k**	6.7	5.7
**1l**	7.4	6.2
**1n**	7.2	6.5
**1o**	7.4	6.5
**4c**	7.1	6.2
**4d**	6.7	6.2
**4e**	7.0	6.2
**4f**	7.3	5.9
**4g**	6.9	6.0
**4i**	6.7	6.0
**5**	6.6	5.1
**6**	6.2	5.6
**8d**	6.5	5.8
**9**	7.1	6.9
**10**	7.3	6.3
**11**	6.6	5.8
**12**	6.5	5.6
**13**	6.7	5.7
**14**	7.2	6.1
**15**	7.2	6.5
**16**	6.9	6.1
**17**	6.8	6.0
**18**	6.4	5.8
**19**	7.0	6.0
**20**	6.2	5.7
**21**	6.0	5.8
**23b**	6.5	5.6
**24a**	6.4	5.7
**(*R*)-24a**	6.3	6.1
**24e**	6.5	5.6
**25a**	7.1	6.1
**25b**	6.5	5.5
**25c**	6.5	5.2
**25e**	6.6	5.7
**27**	6.3	5.6
**28**	6.0	<5
**31**	6.2	5.4

Figure S1. DLS data for **1a**

Dynamic Light Scattering (DLS) Assay. Aggregation behavior of **1a** was determined by DLS in duplicate at 25 ºC in 25mM HEPES buffer (pH 7.4) containing 0.003% Tween-20 and 1mM DTT, using a 10 mM stock solution contianing 2% DMSO. Light scattering was measured using a DynaPro Plate Reader III. Buffer with 2% DMSO control produced an average laser intensity of 858 kCnts/s.

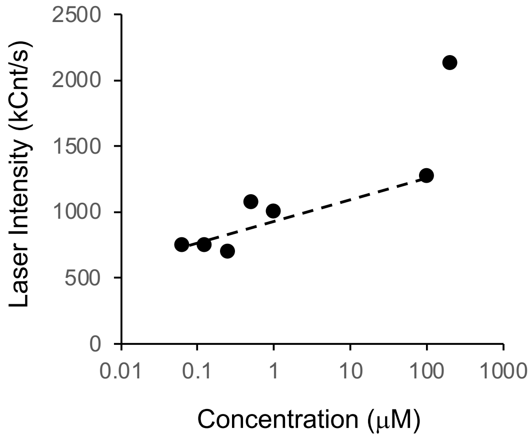

NMR spectra of final compounds

Figure S2: ^{1}H NMR (500 MHz, DMSO-d_6) for **1a**

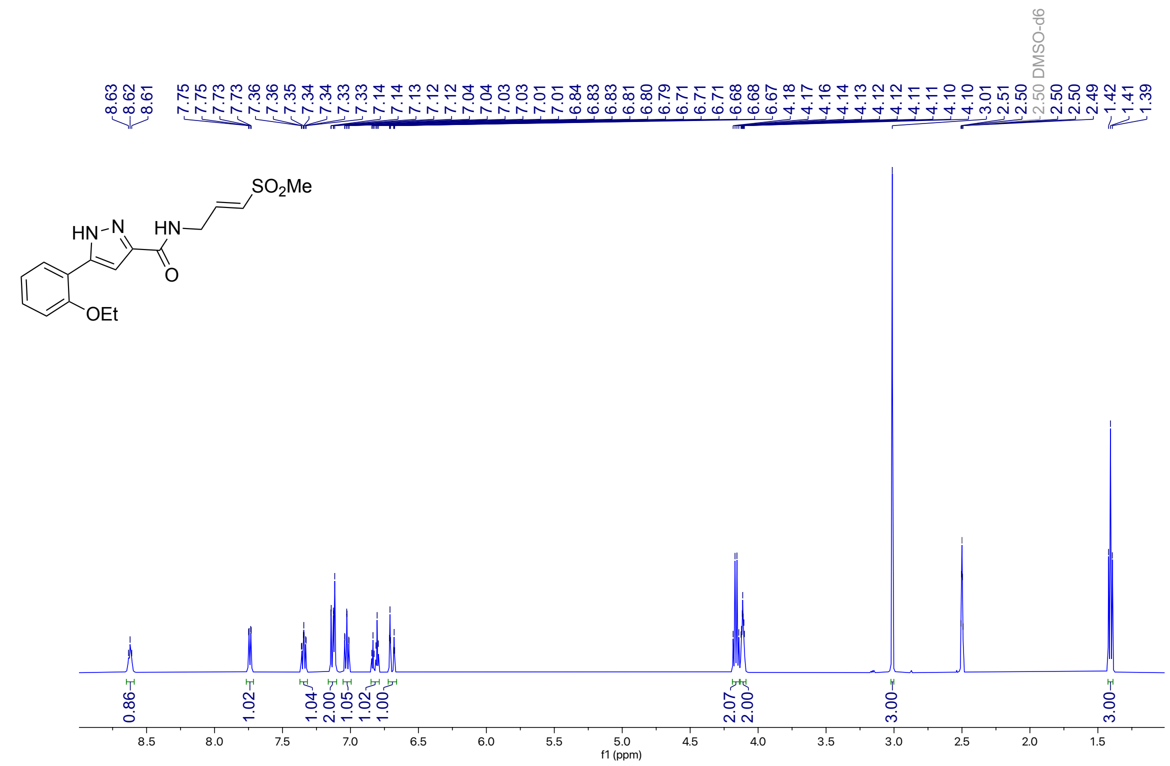

Figure S3: ^{13}C NMR (126 MHz, DMSO-d_6) for **1a**

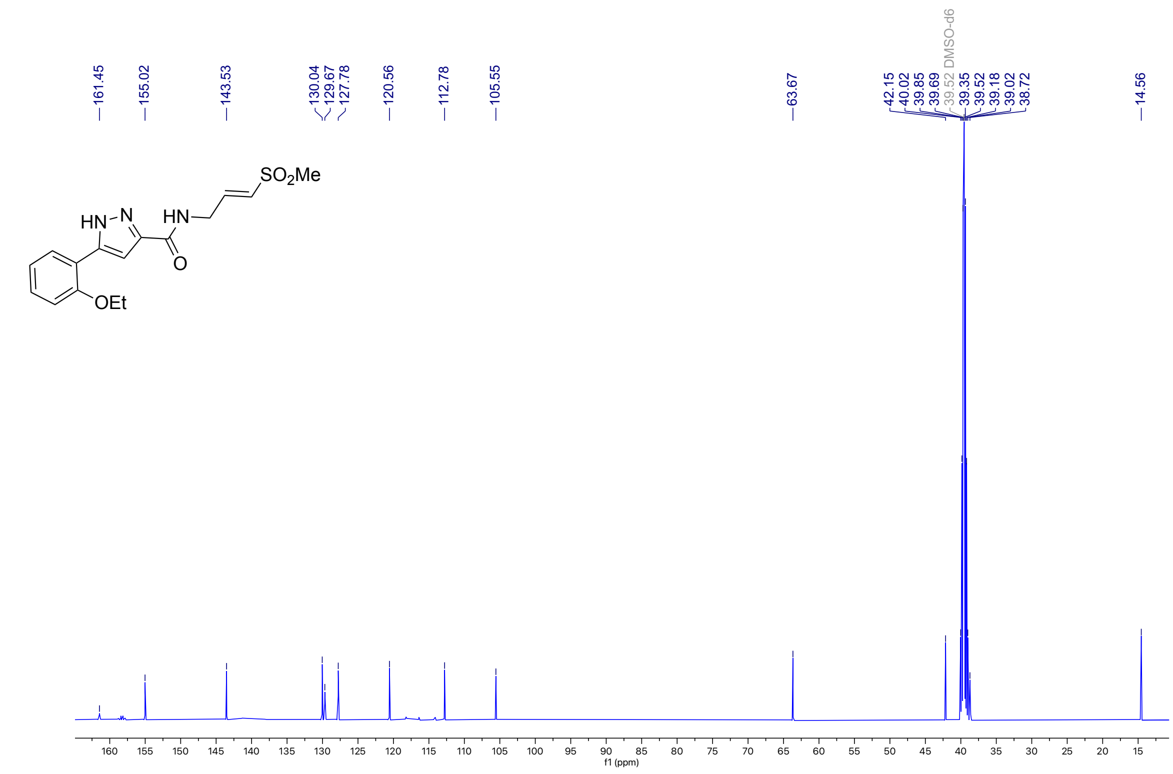

Figure S4: ^{1}H NMR (500 MHz, DMSO-d_6) for **2**

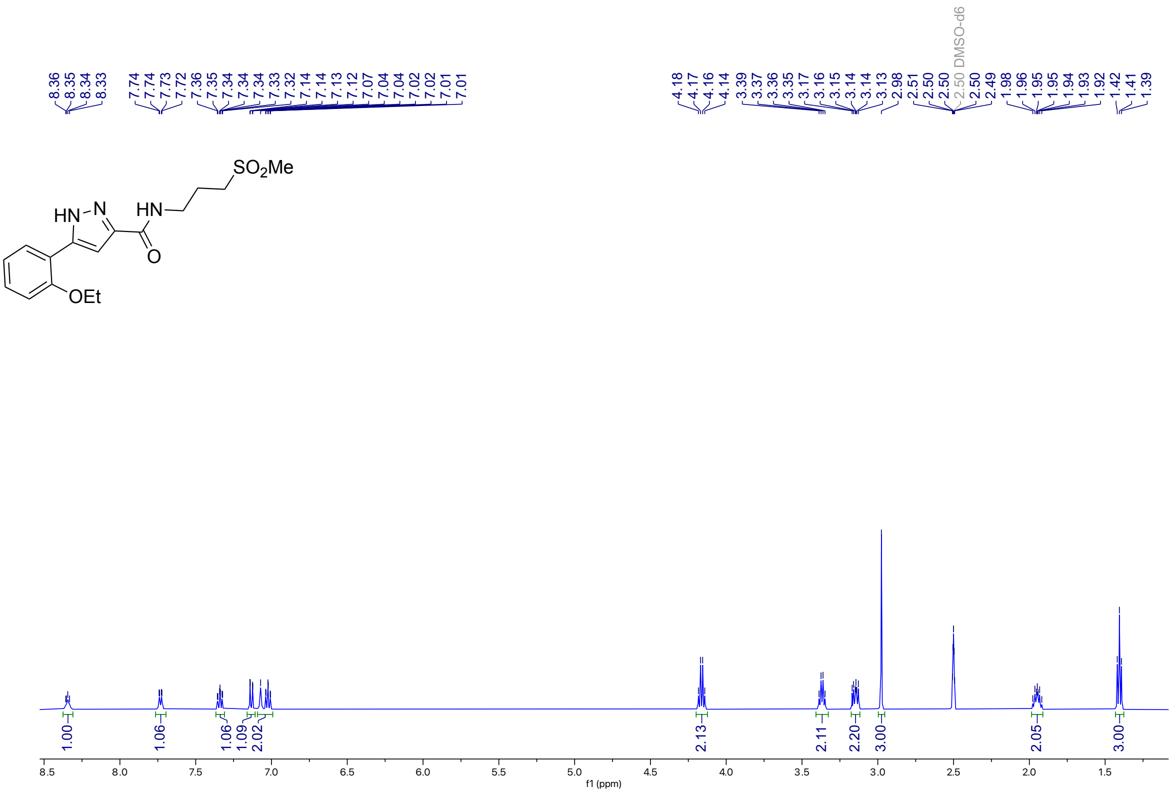

Figure S5: ^{13}C NMR (126 MHz, DMSO-d_6) for **2**

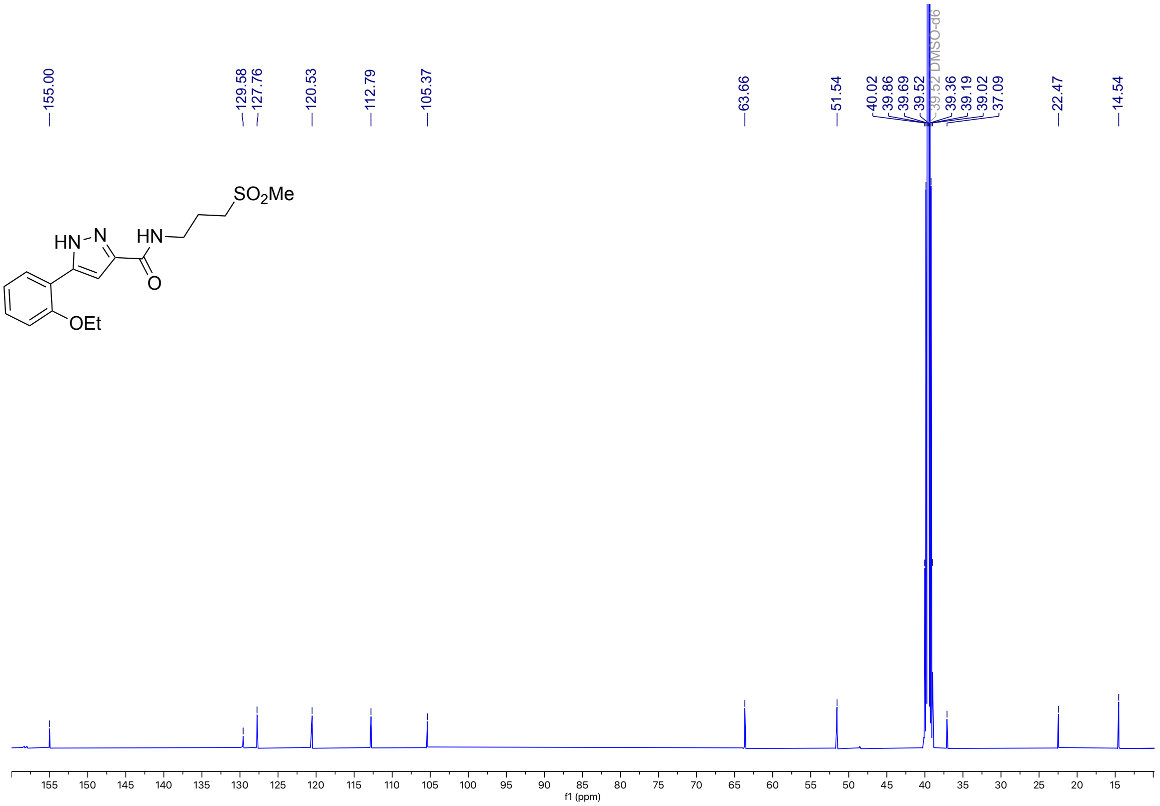

Figure S6: ^{1}H NMR (400 MHz, DMSO-d_6) for RA-0002993-01 **3**

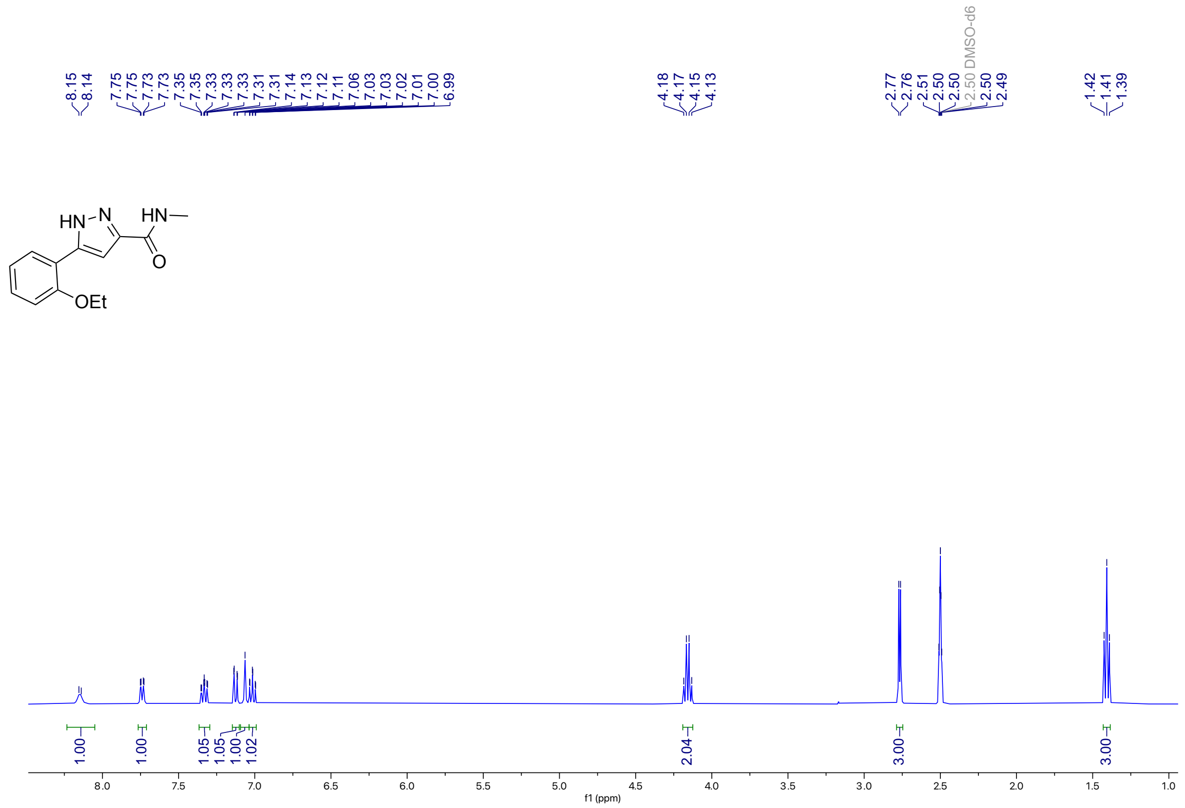

Figure S7: ^{13}C NMR (126 MHz, DMSO-d_6) for **3**

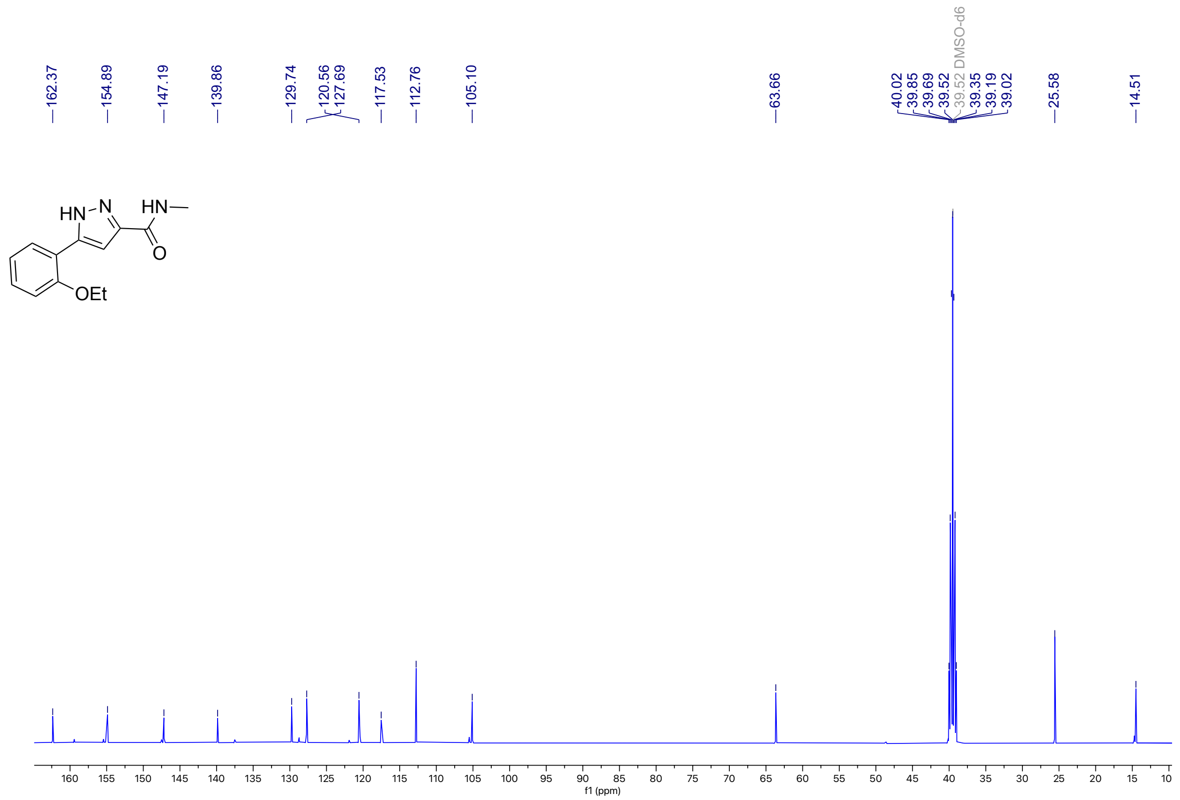

Figure S8: ^{1}H NMR (400 MHz, DMSO-d_6) for **1b**

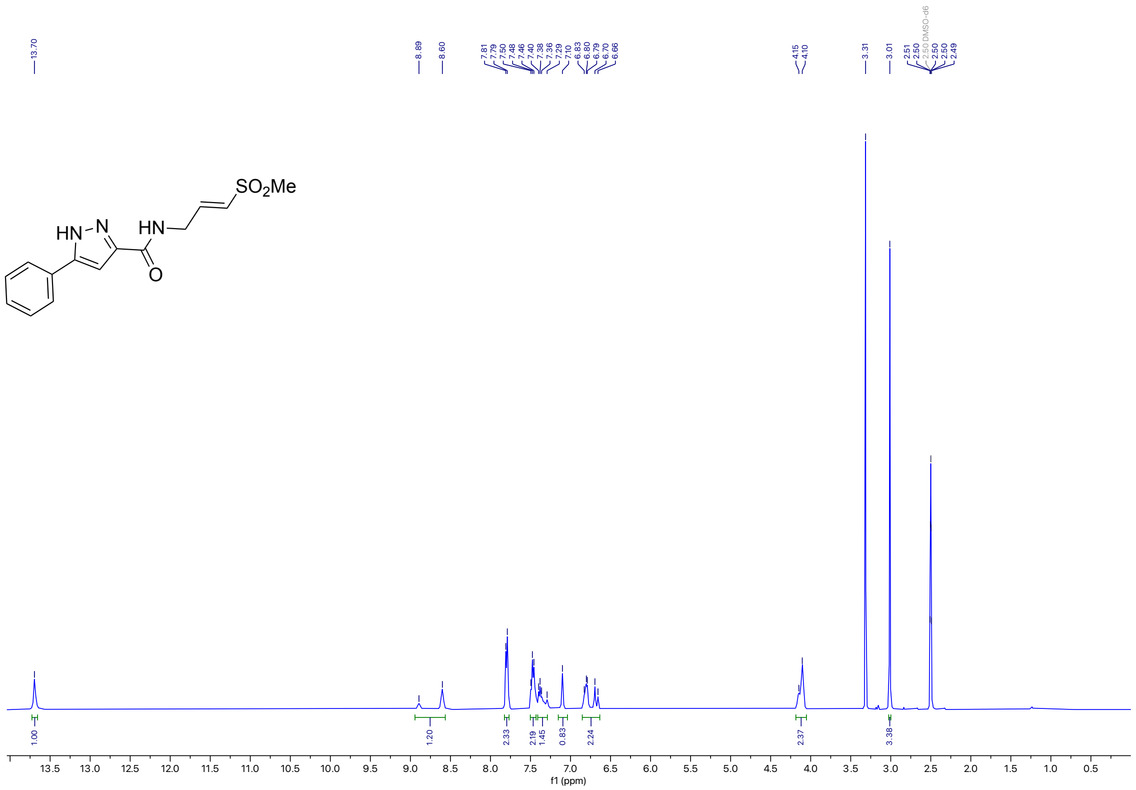

Figure S9: ^{13}C NMR (100 MHz, DMSO-d_6) for **1b**

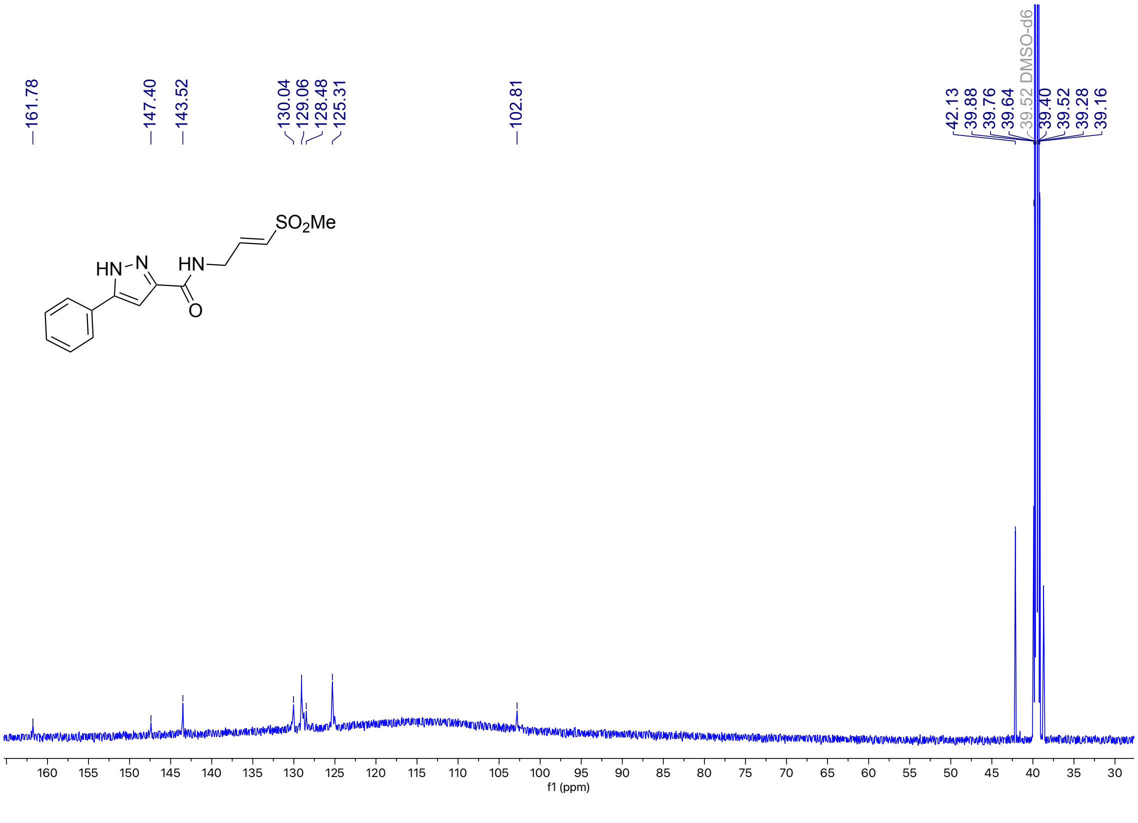

Figure S10: ^{1}H NMR (500 MHz, DMSO-d_6) for **1c**

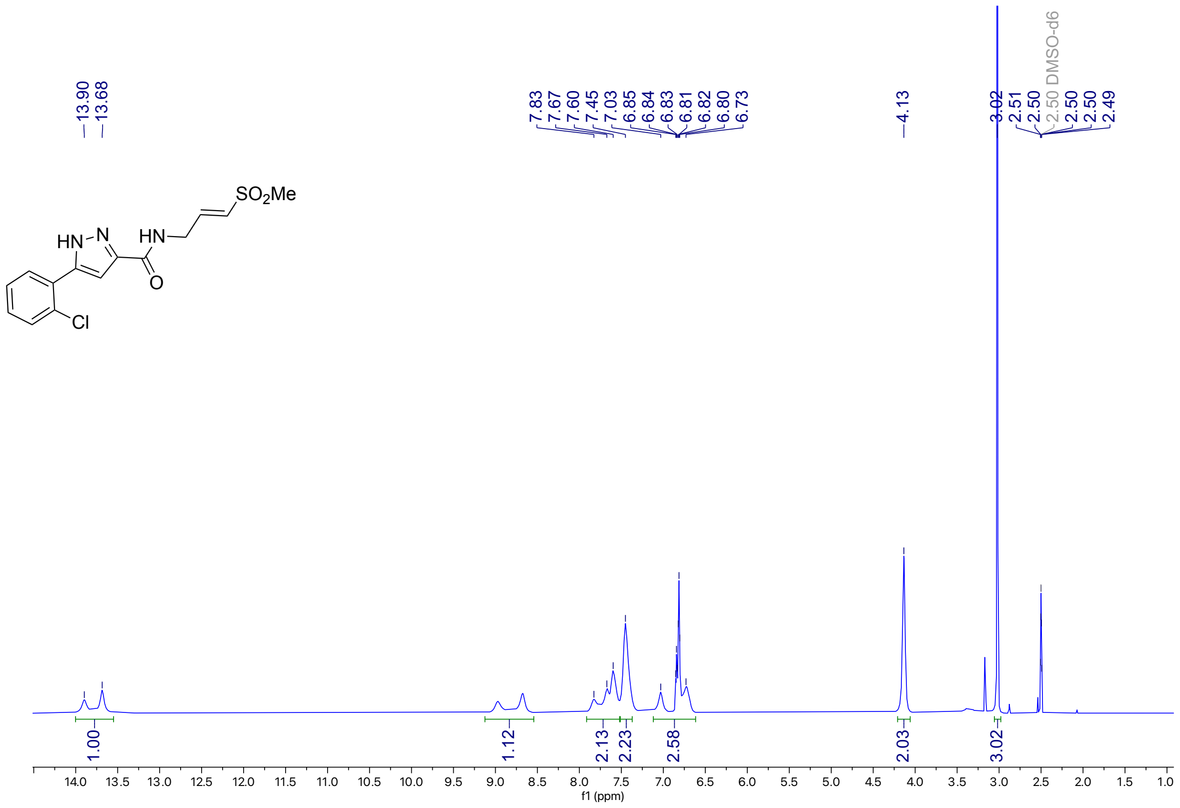

Figure S11: ^{13}C NMR (176 MHz, DMSO-d_6) for **1c**

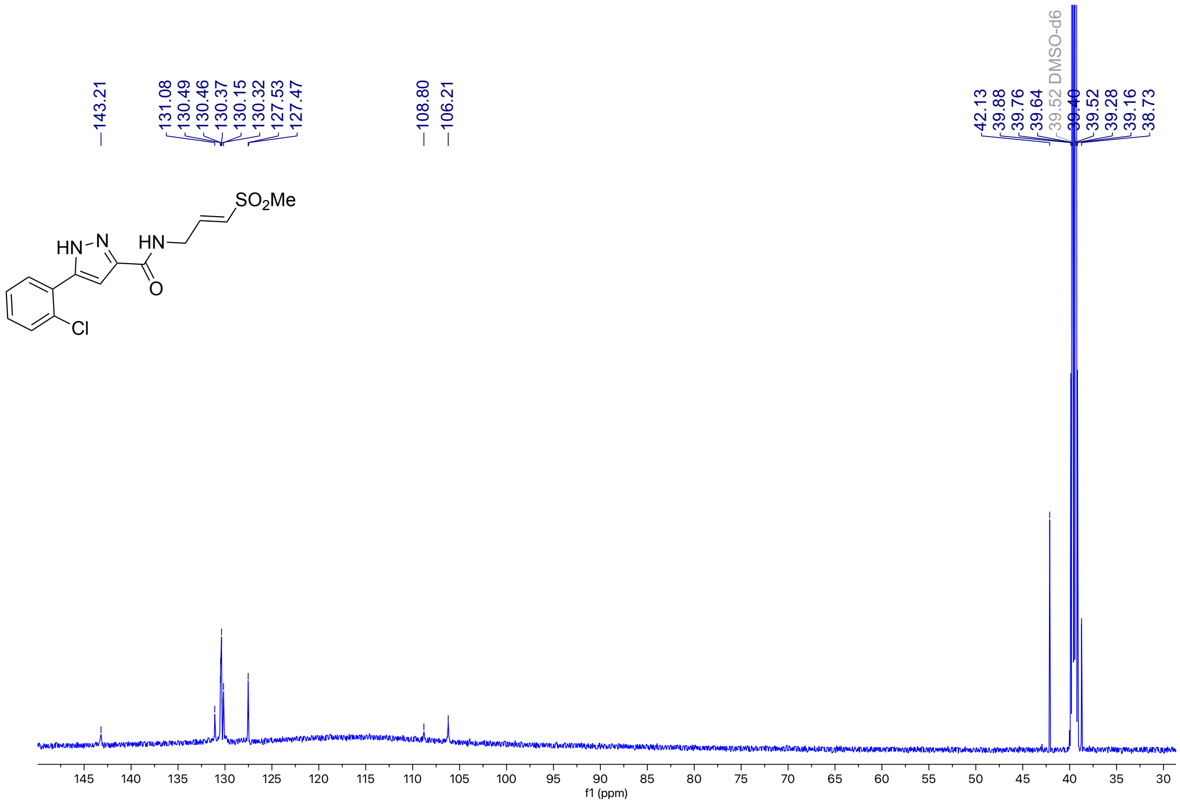

Figure S12: ^{1}H NMR (500 MHz, DMSO-d_6) for **1d**

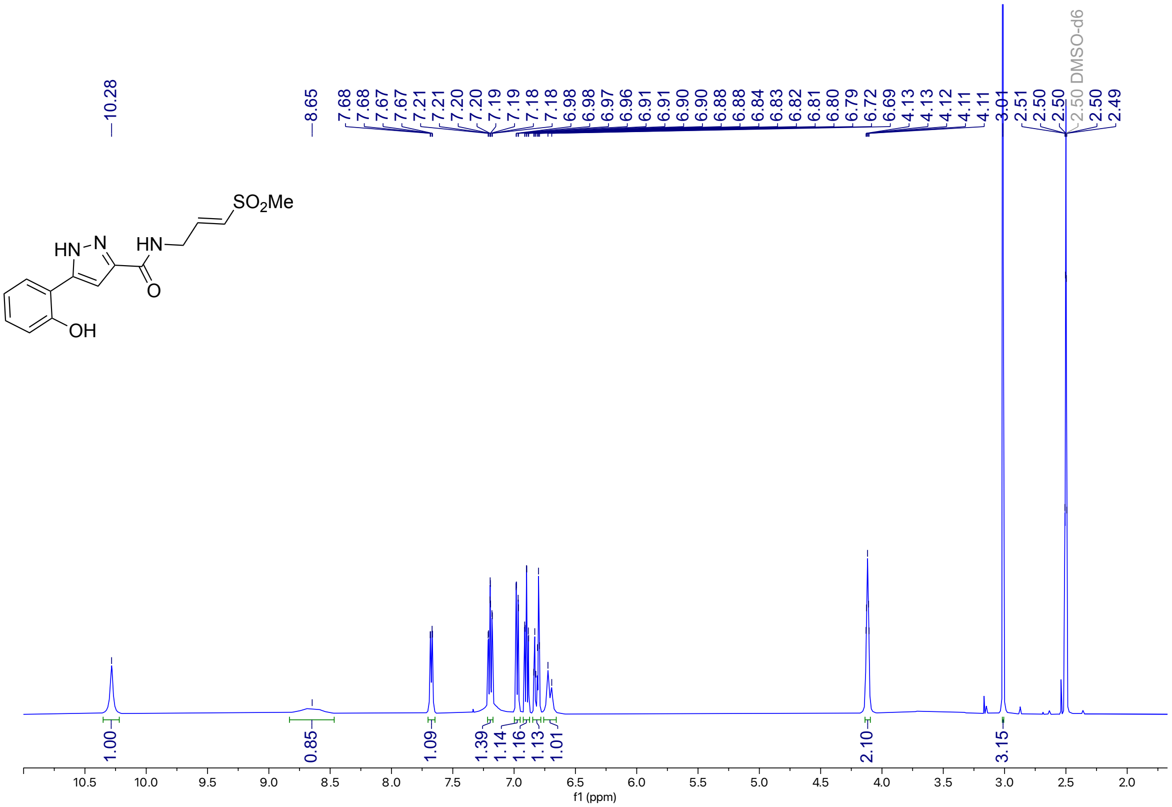

Figure S13: ^{13}C NMR (126 MHz, DMSO-d_6) for **1d**

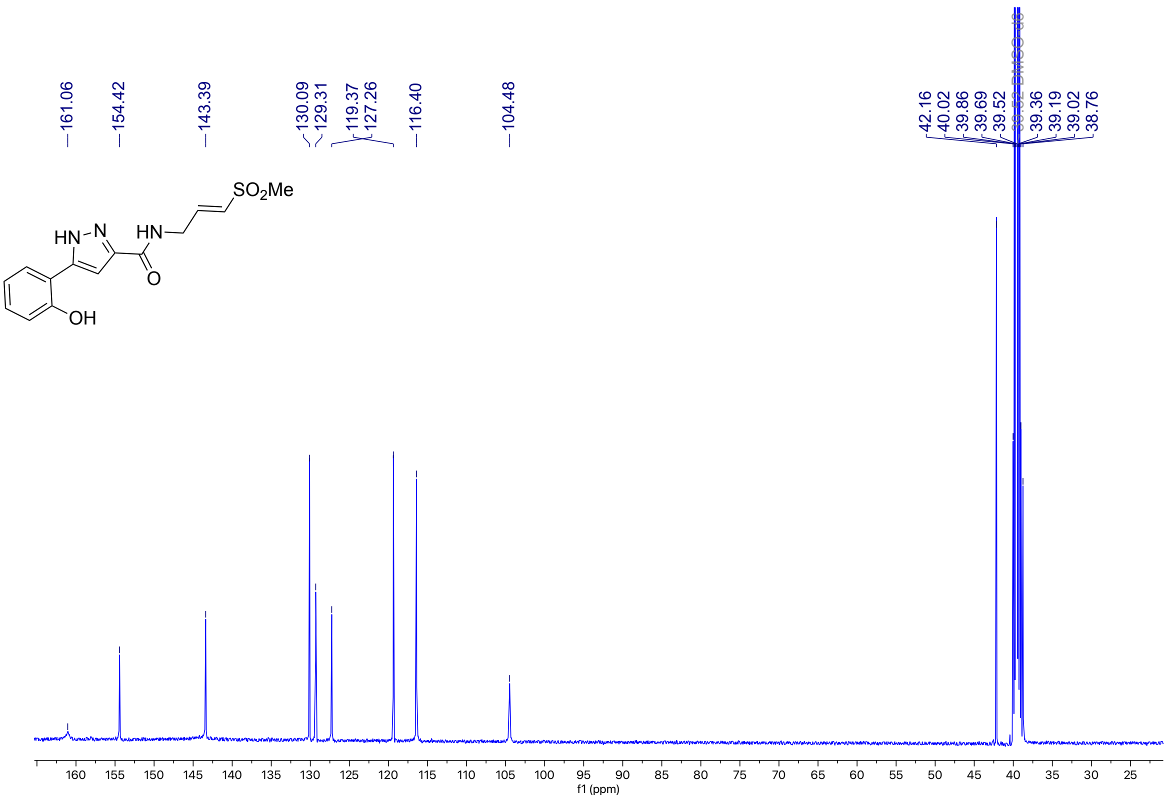

Figure S14: ^{1}H NMR (500 MHz, DMSO-d_6) for **1e**

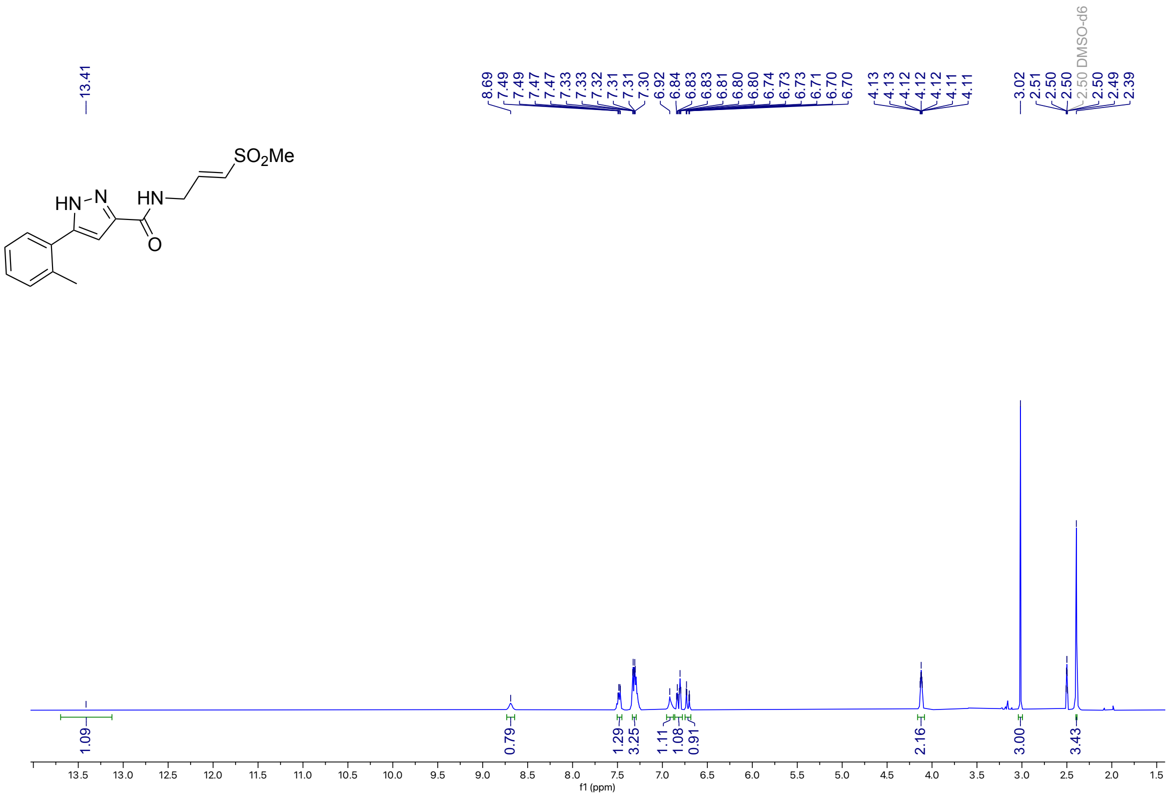

Figure S15: ^{13}C NMR (214 MHz, DMSO-d_6) for **1e**

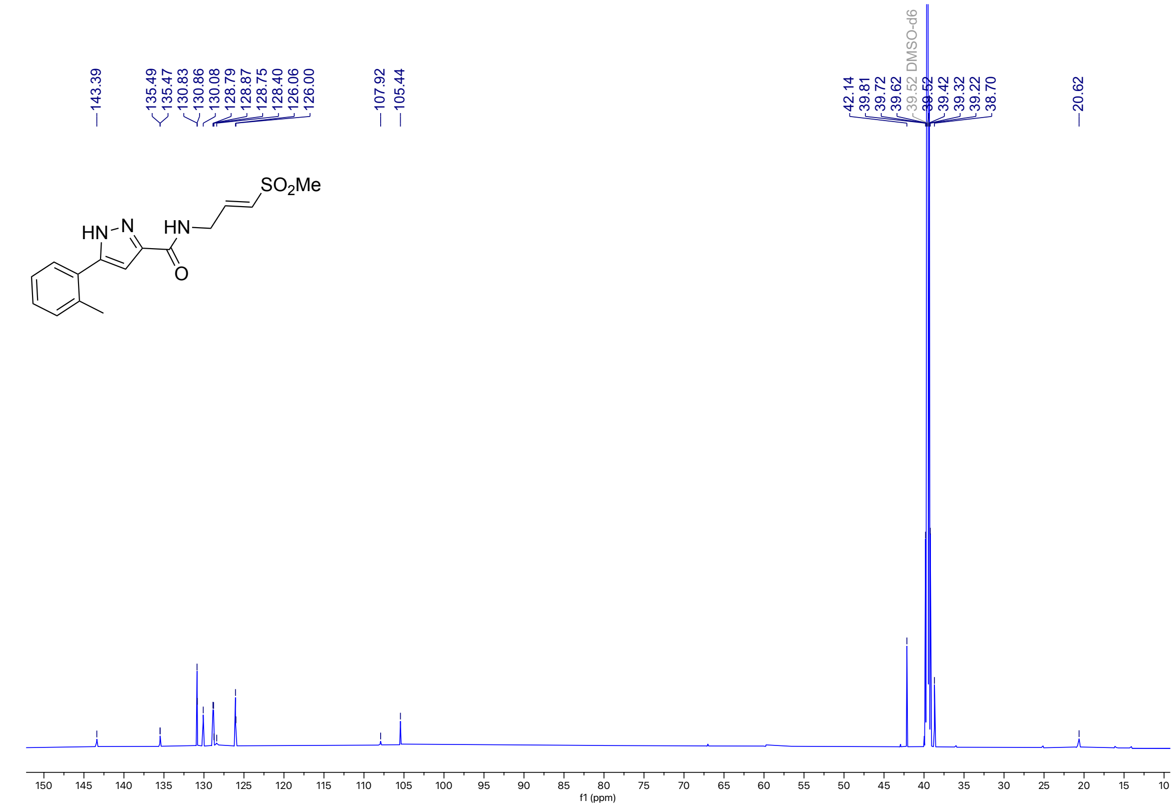

Figure S16: ^{1}H NMR (400 MHz, DMSO-d_6) for **1f**

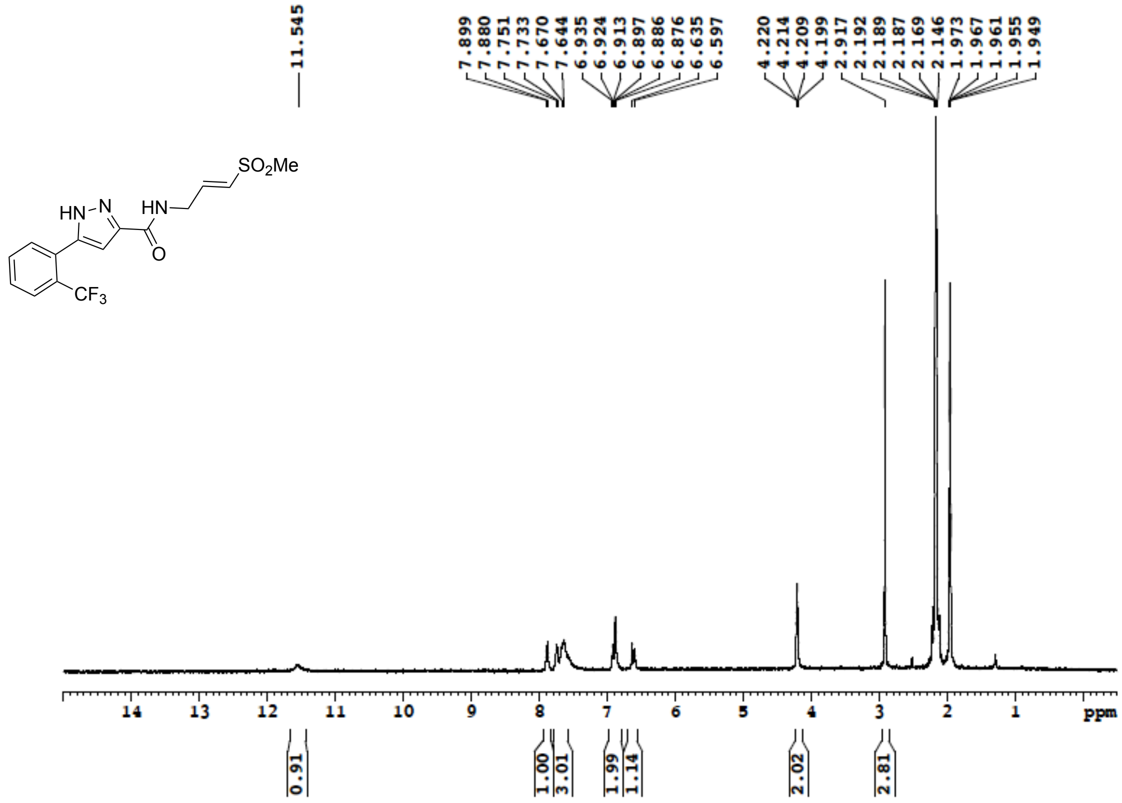

Figure S17: ^{13}C NMR (100 MHz, DMSO-d_6) for **1f**

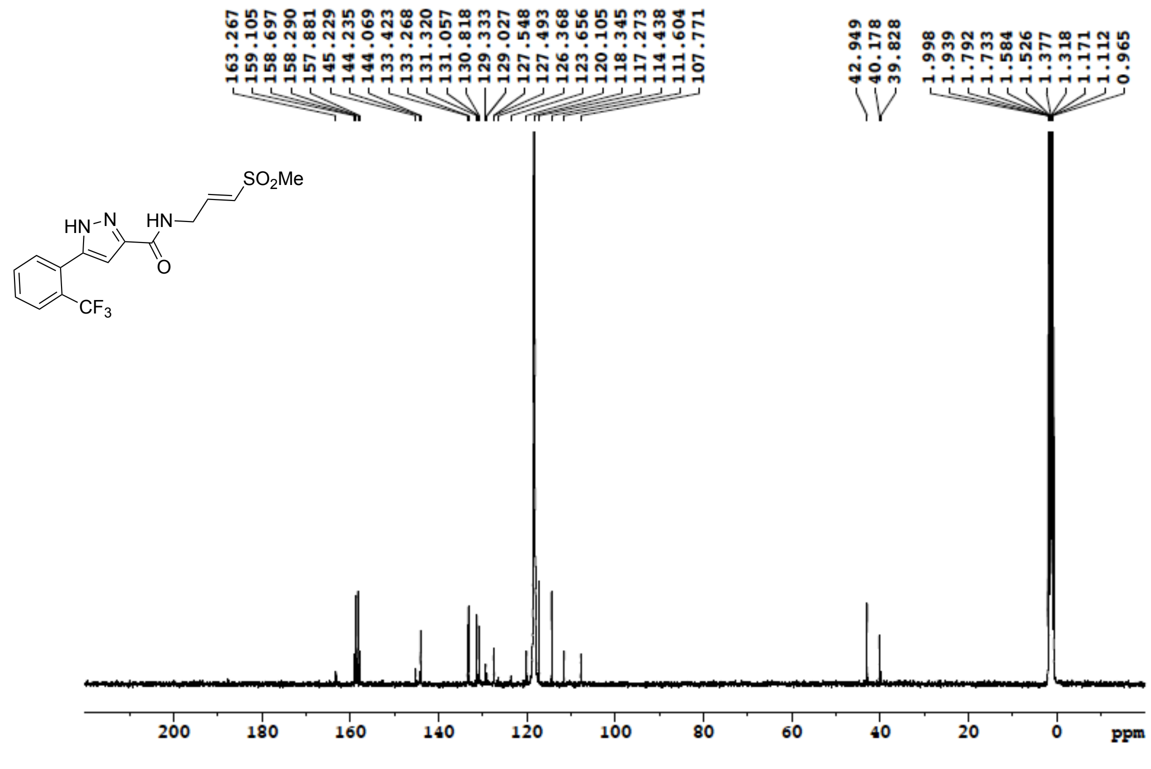

Figure S18: ^{1}H NMR (500 MHz, DMSO-d_6) for **1g**

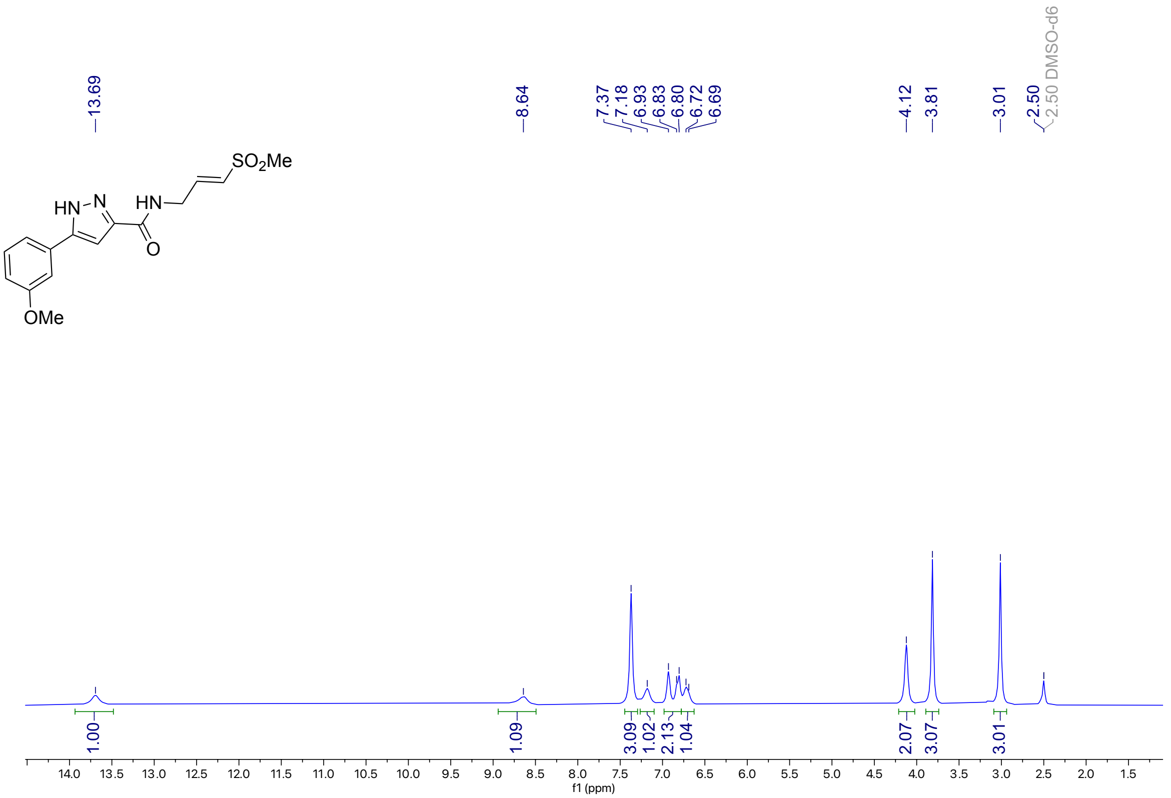

Figure S19: ^{13}C NMR (126 MHz, DMSO-d_6) for **1g**

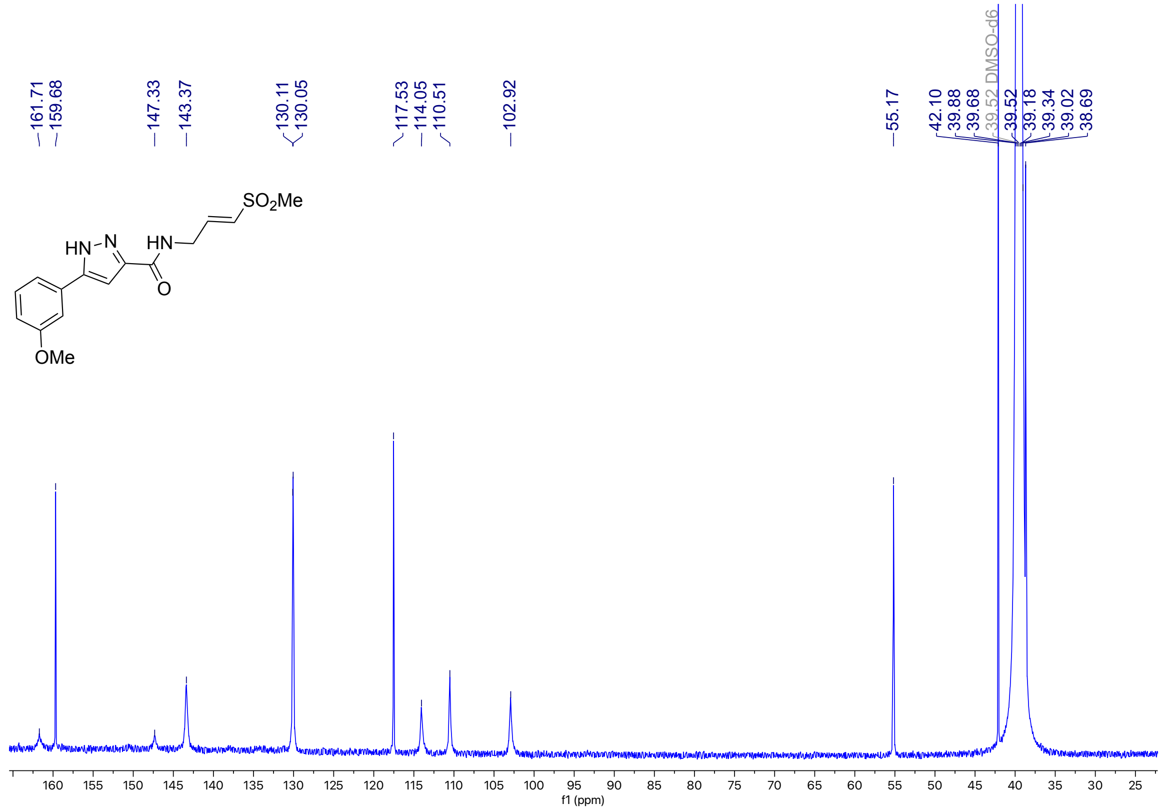

Figure S20: ^{1}H NMR (400 MHz, DMSO-d_6) for **1h**

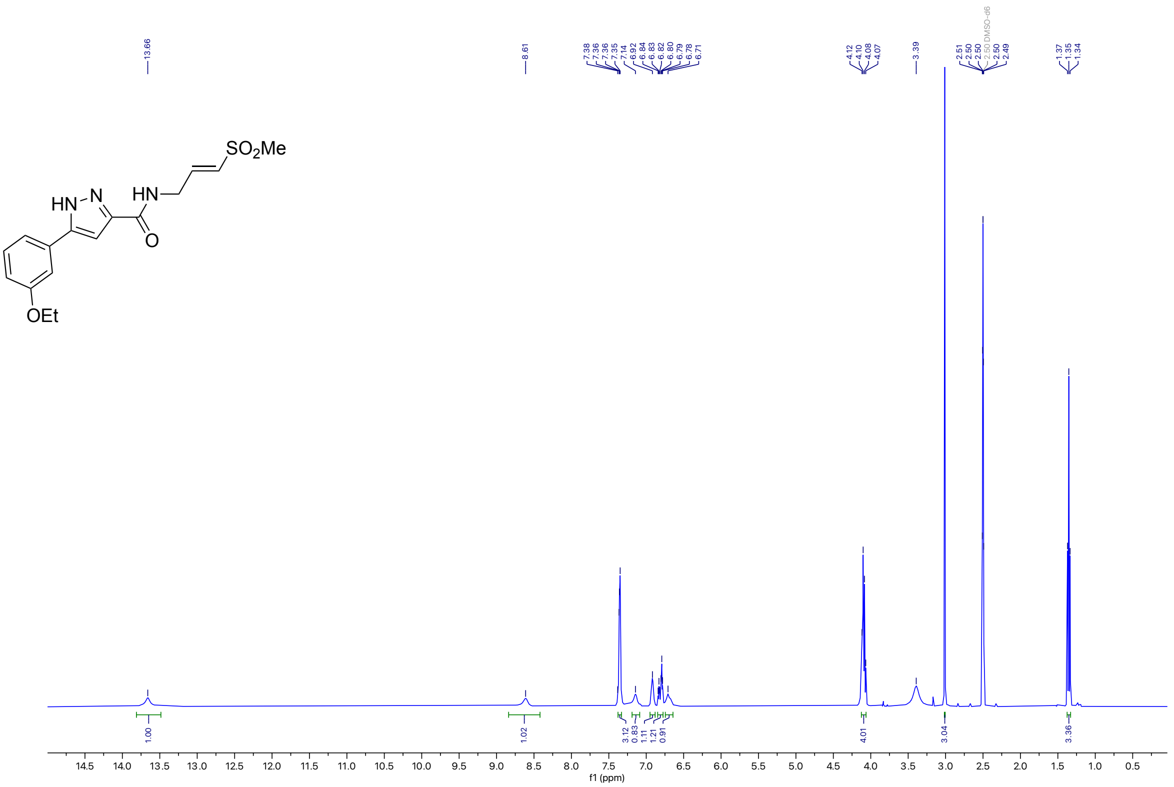

Figure S21: ^{13}C NMR (214 MHz, DMSO-d_6) for **1h**

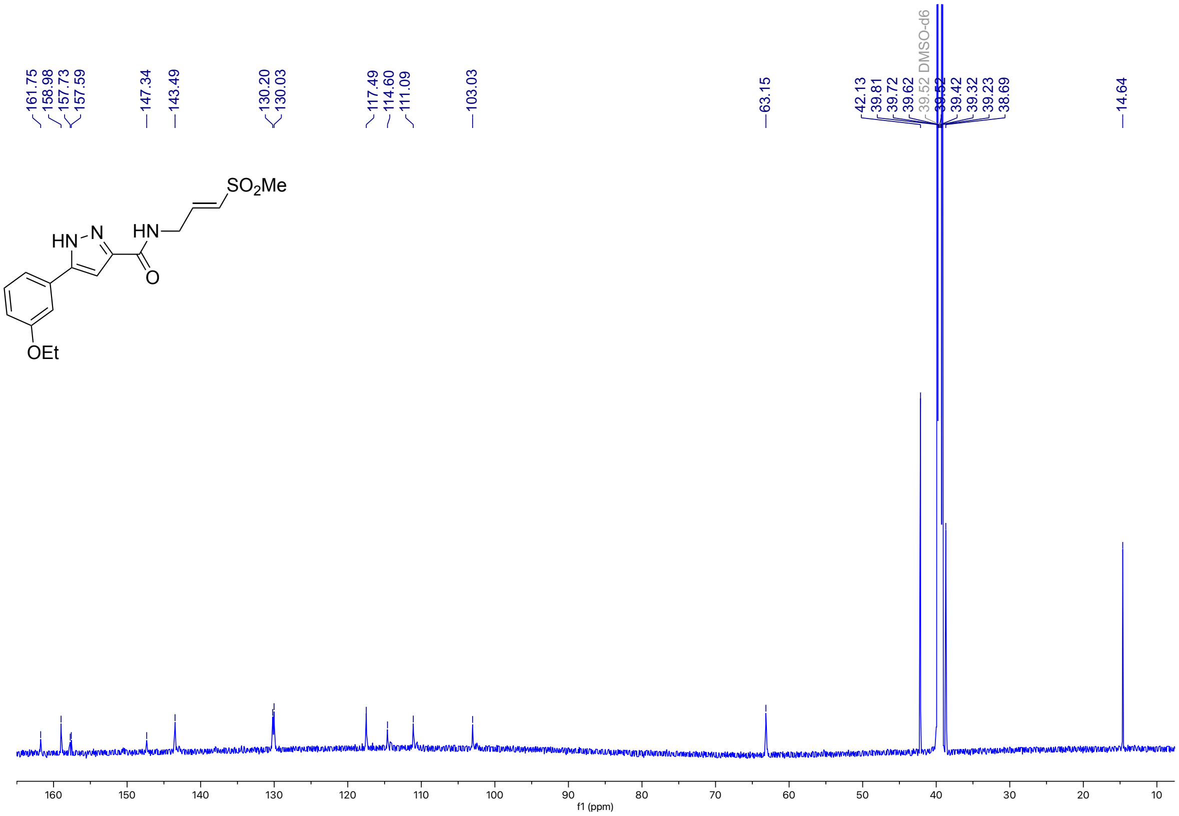

Figure S22: ^{1}H NMR (400 MHz, DMSO-d_6) for **1i**

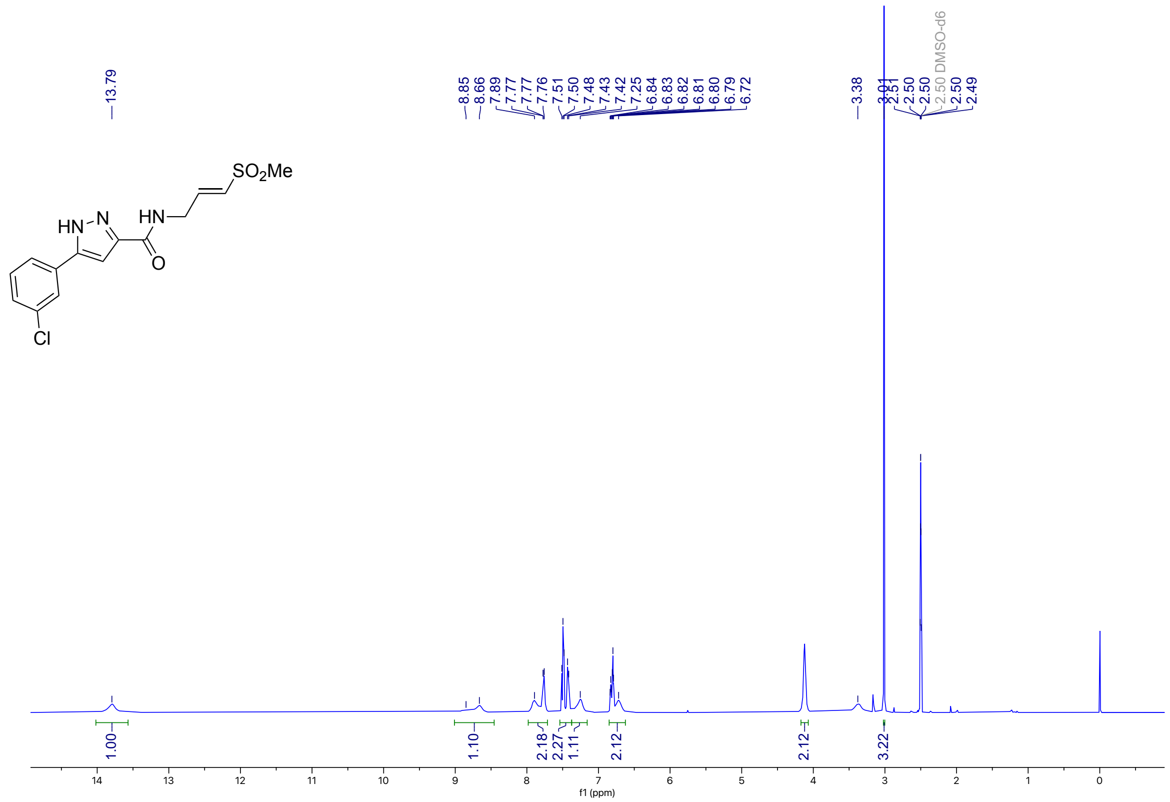

Figure S23: ^{13}C NMR (100 MHz, DMSO-d_6) for **1i**

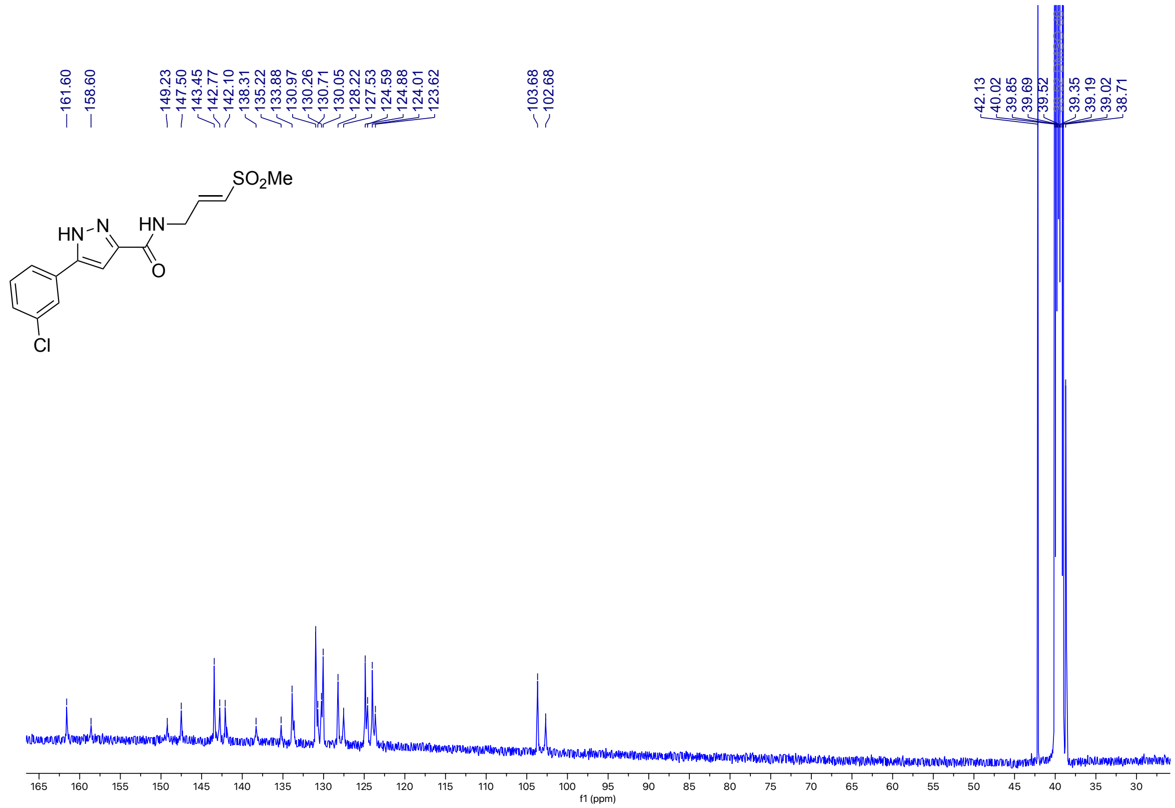

Figure S24: ^{1}H NMR (500 MHz, MeOD) for **1j**

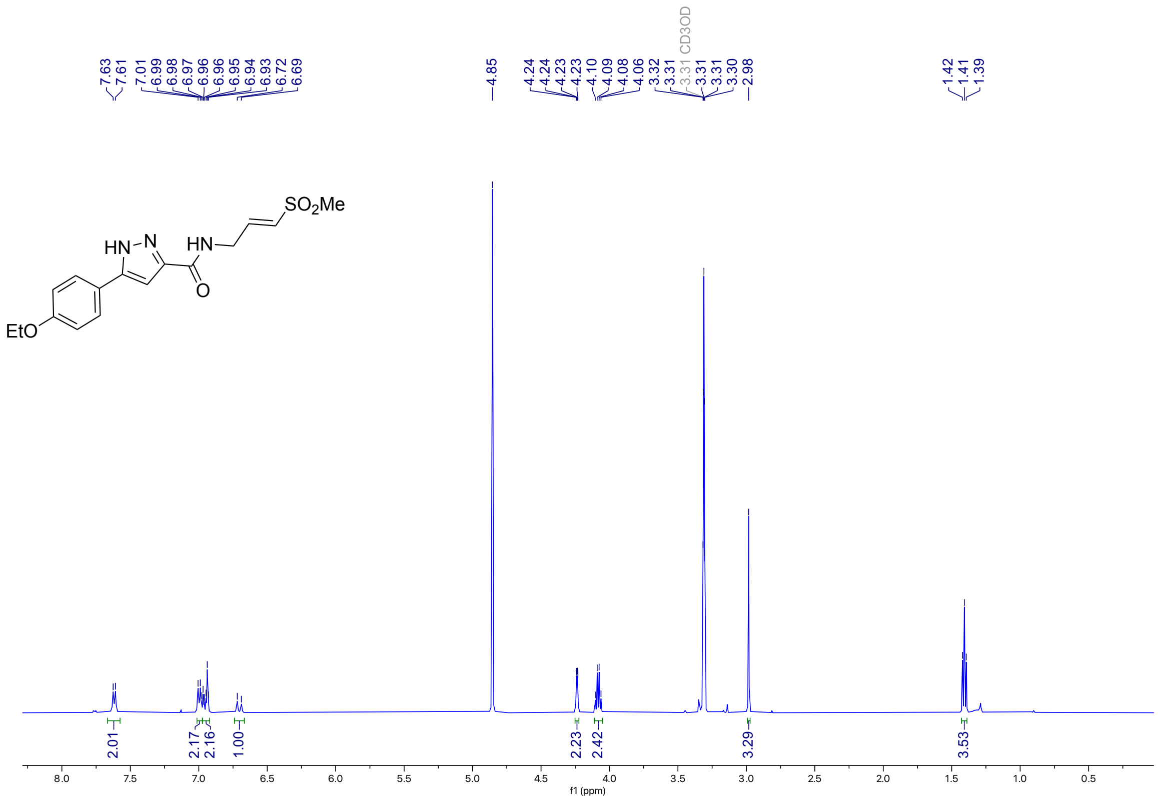

Figure S25: ^{13}C NMR (100 MHz, DMSO-d_6) for **1j**

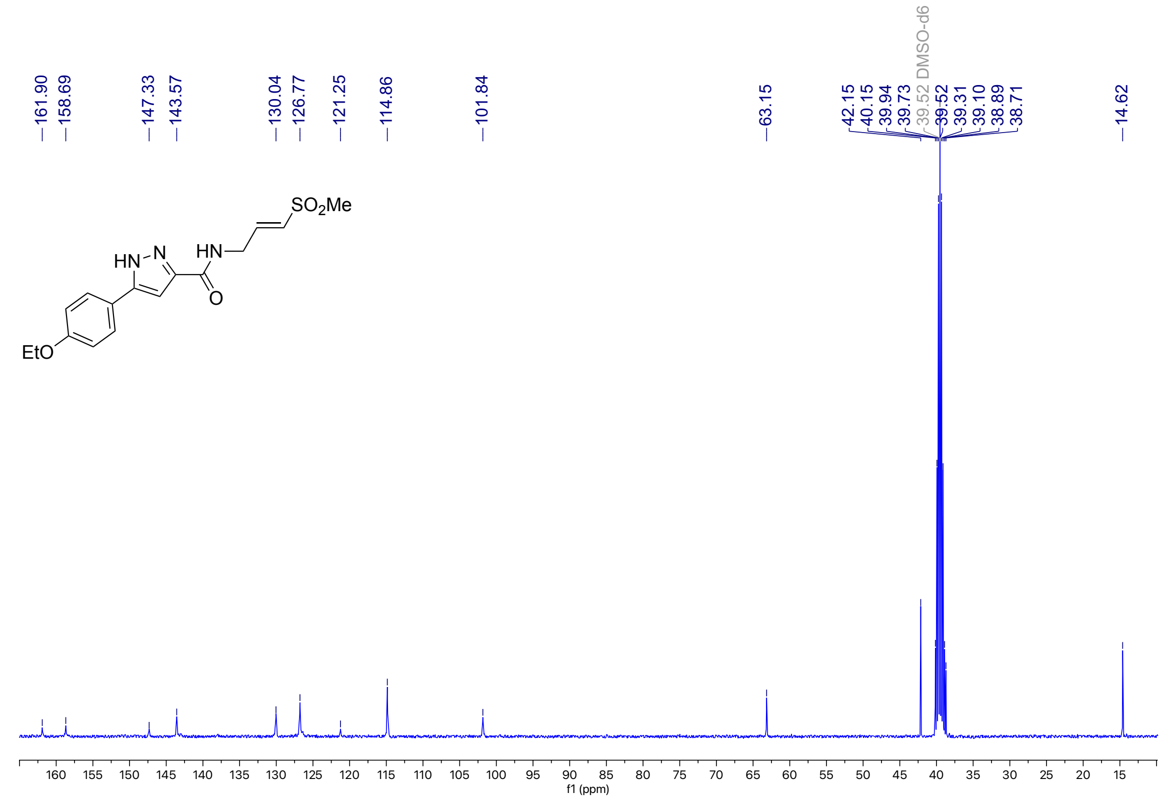

Figure S26: ^{1}H NMR (500 MHz, MeOD) for **1k**

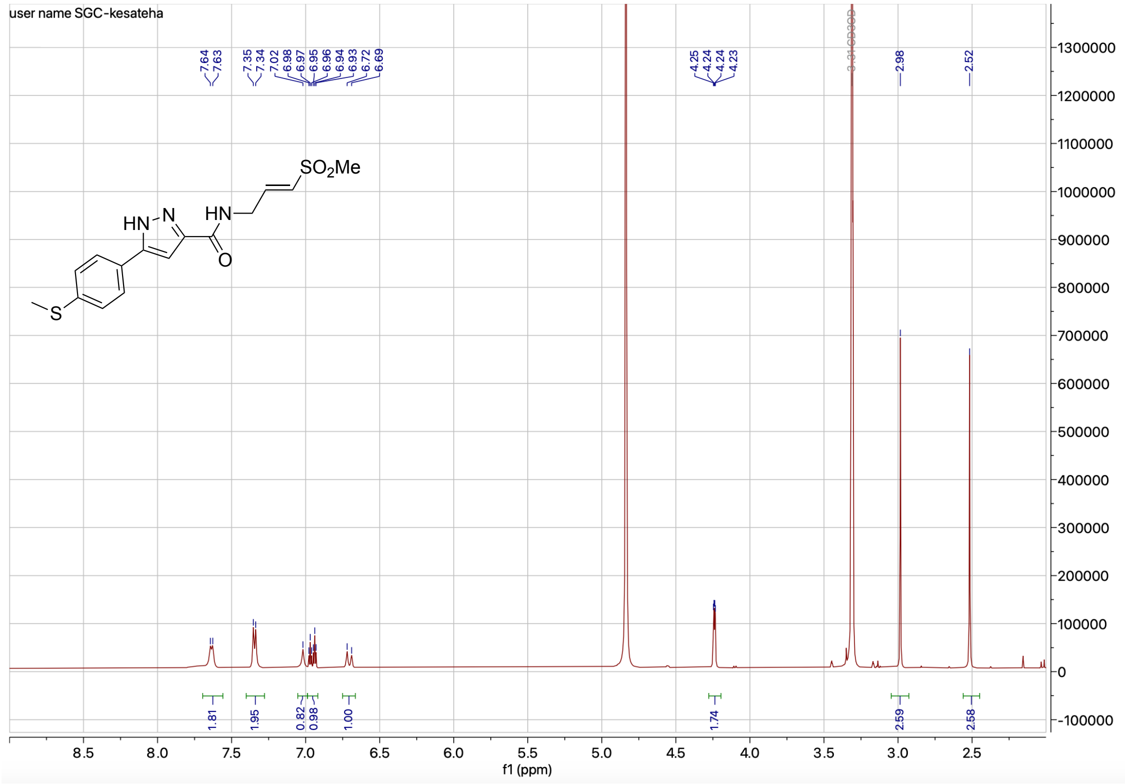

Figure S27: ^{13}C NMR (100 MHz, DMSO-d_6) for **1k**

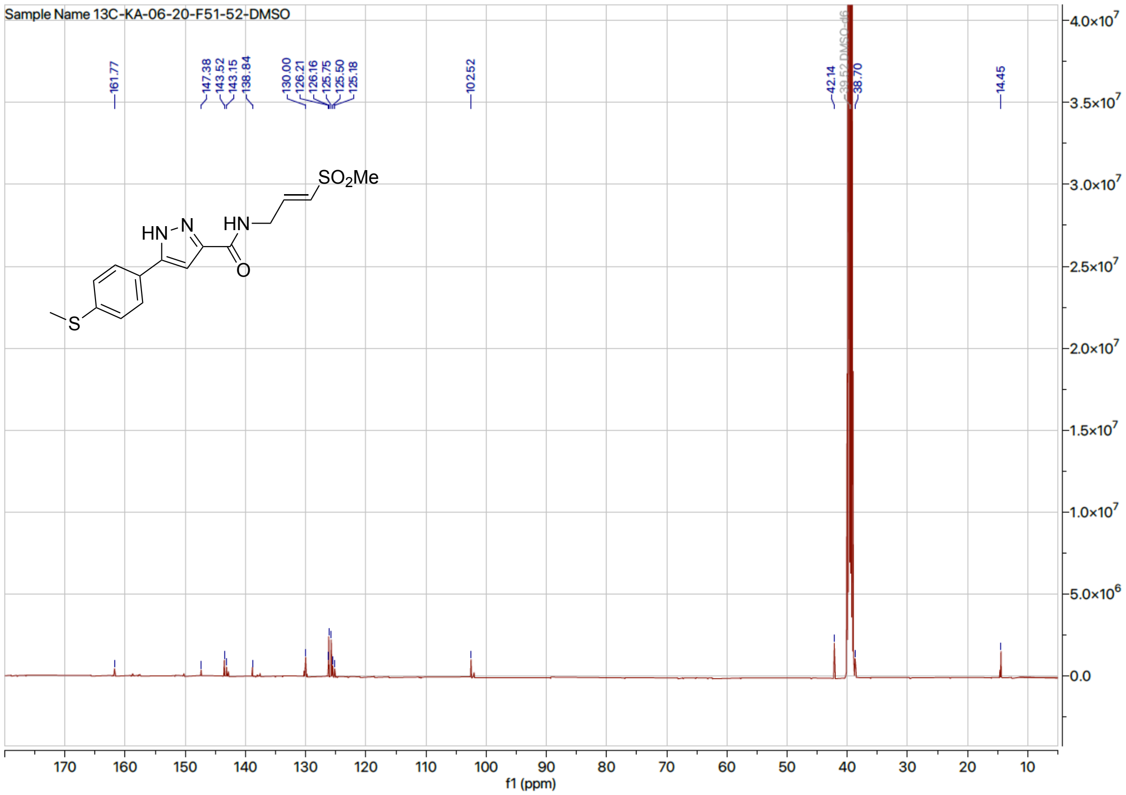

Figure S28: ^{1}H NMR (500 MHz, MeOD) for **1l**

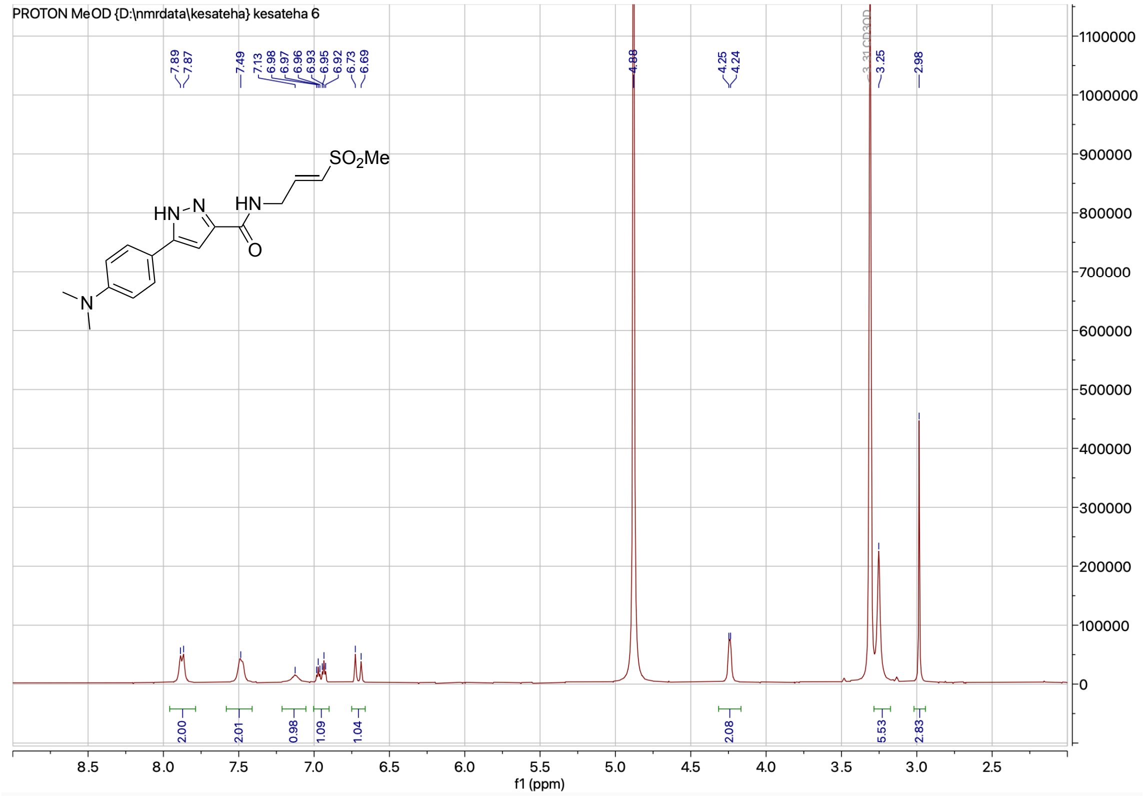

Figure S29: ^{13}C NMR (126 MHz, DMSO-d_6) for **1l**

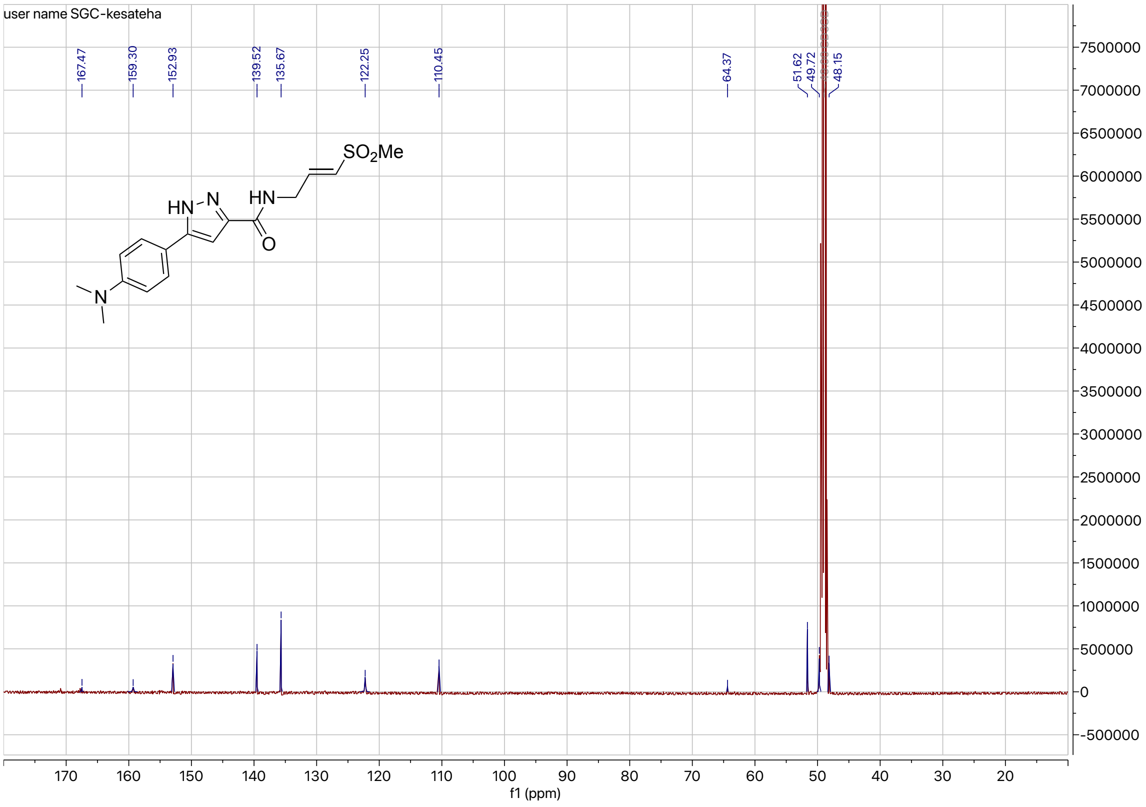

Figure S30: ^{1}H NMR (400 MHz, DMSO-d_6) for **1m**

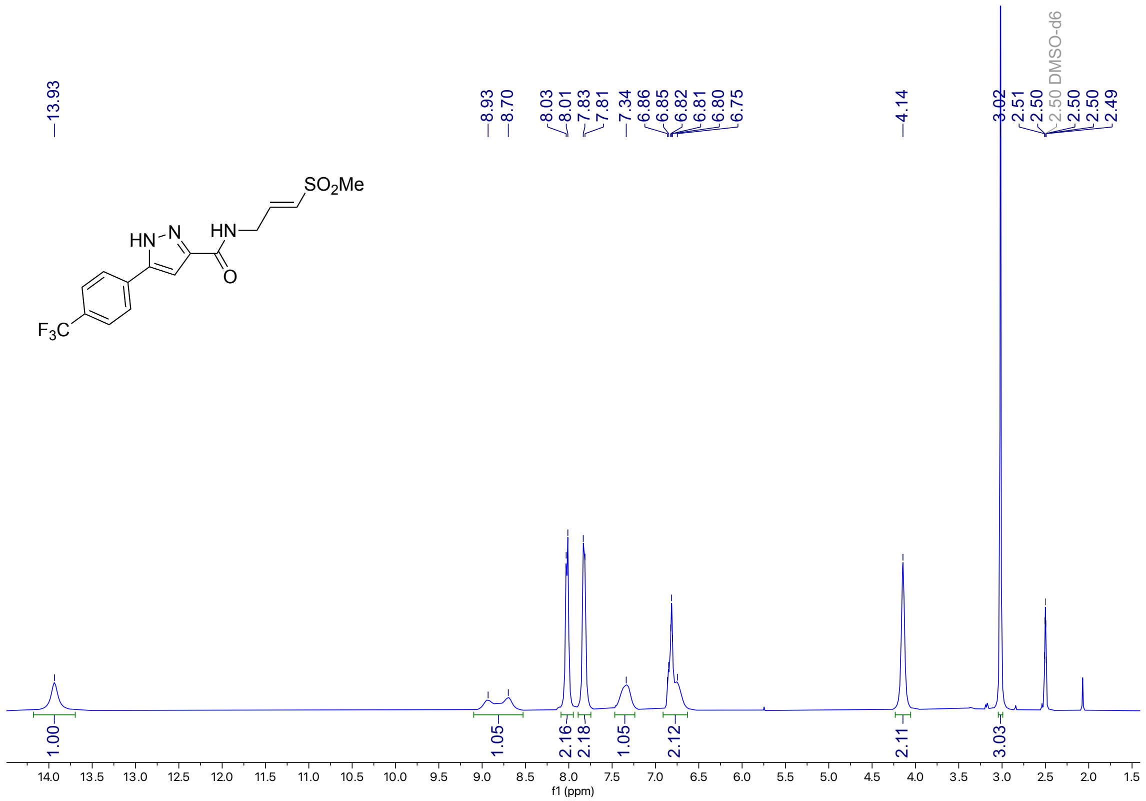

Figure S31: ^{13}C NMR (214 MHz, DMSO-d_6) for **1m**

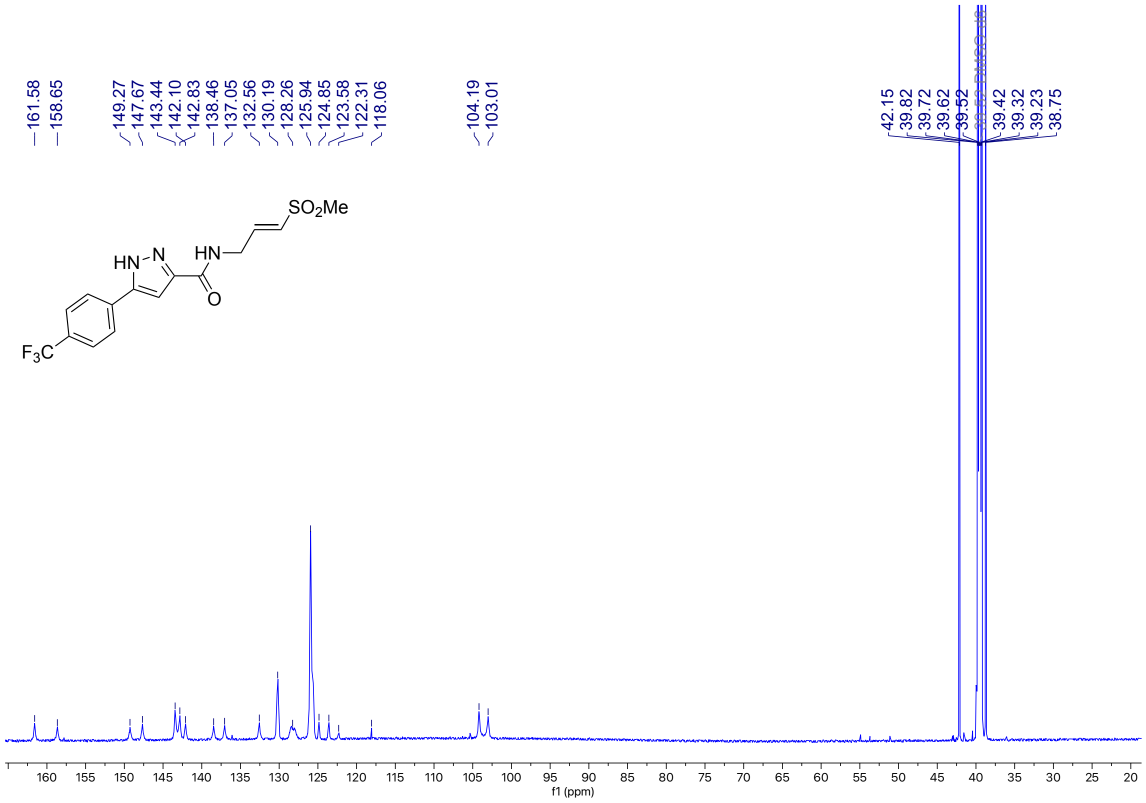

Figure S32: ^{1}H NMR (500 MHz, DMSO-d_6) for **1n**

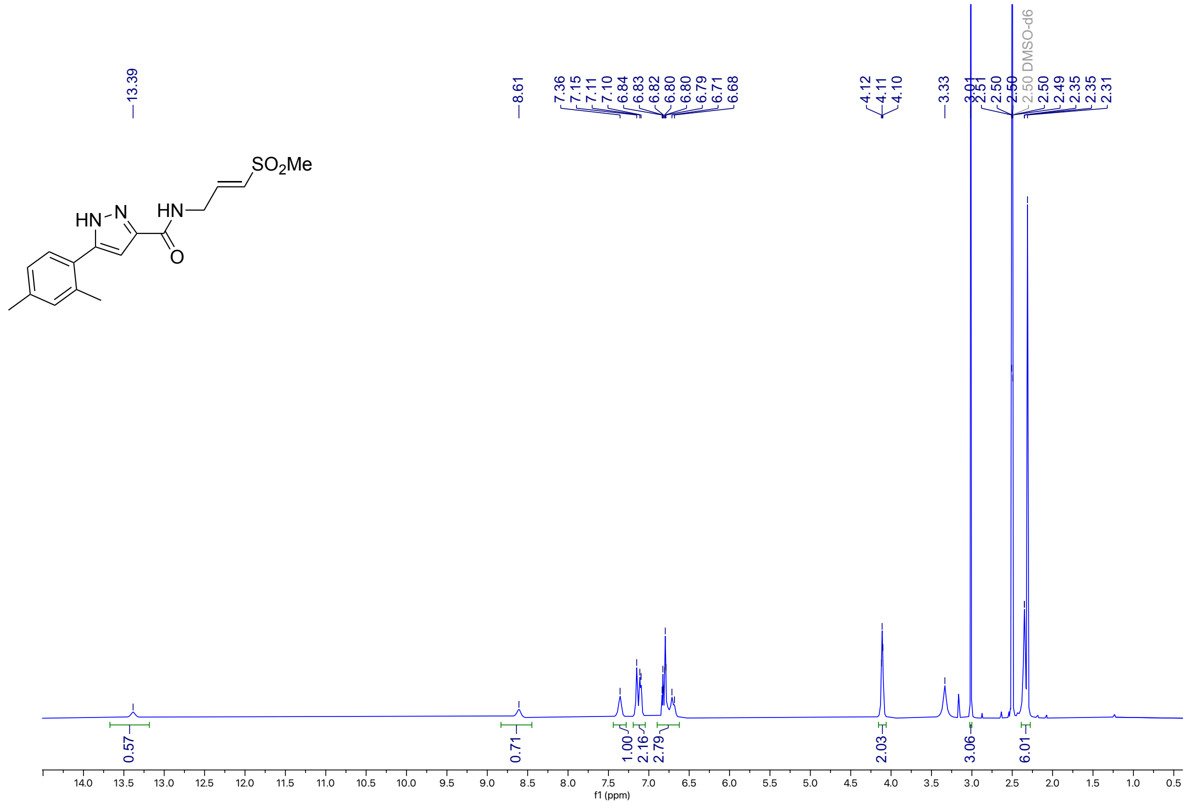

Figure S33: ^{13}C NMR (126 MHz, DMSO-d_6) for **1n**

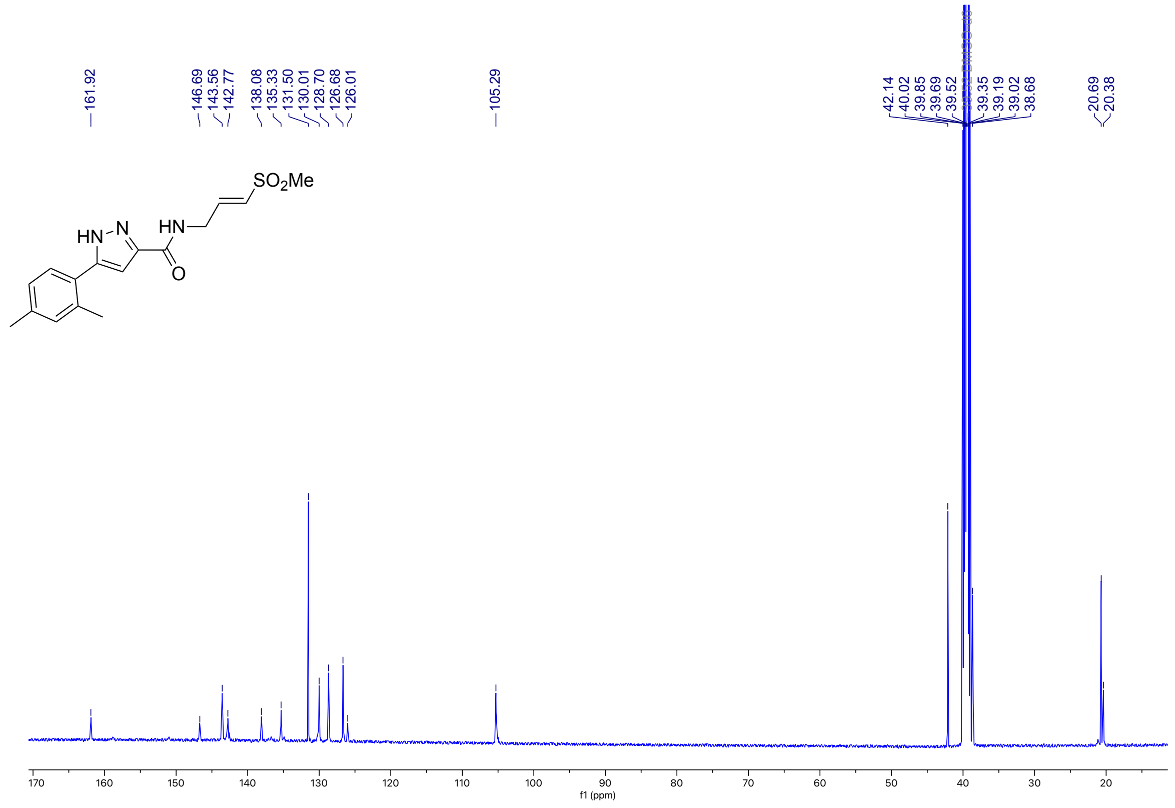

Figure S34: ^{1}H NMR (500 MHz, DMSO-d_6) for **1o**

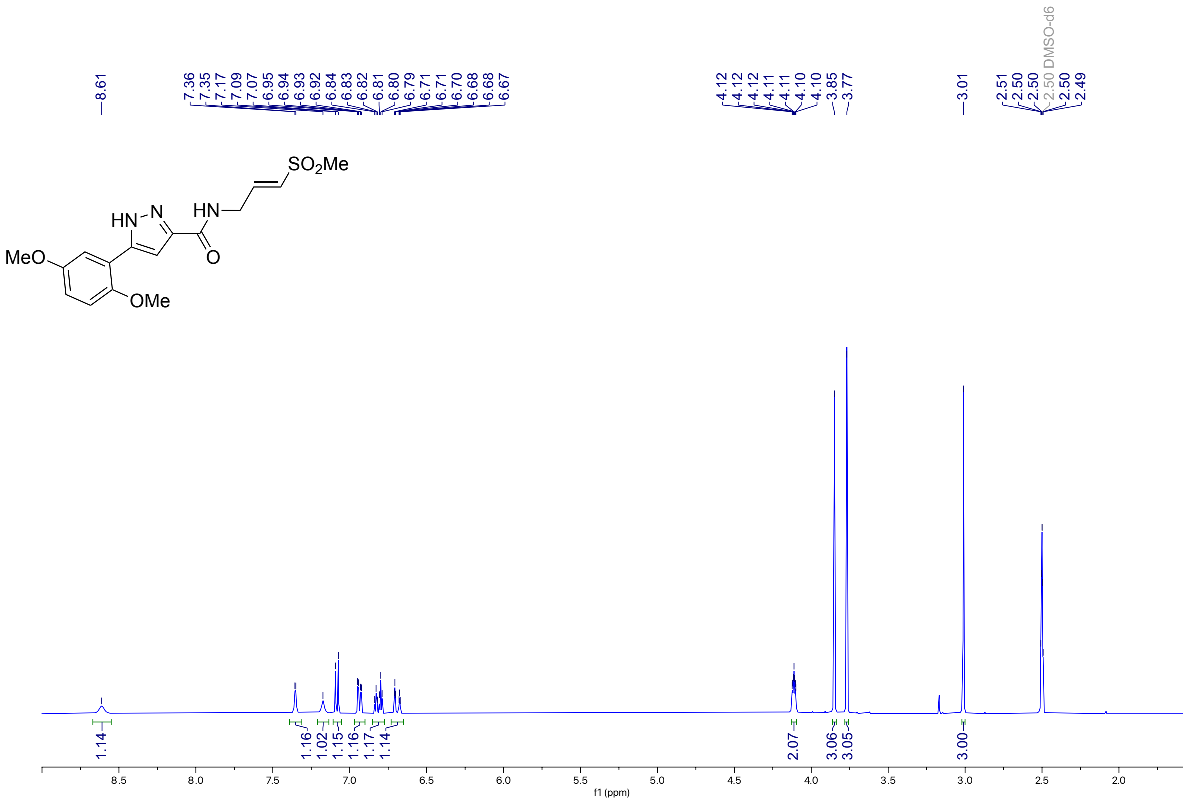

Figure S35: ^{13}C NMR (126 MHz, DMSO-d_6) for **1o**

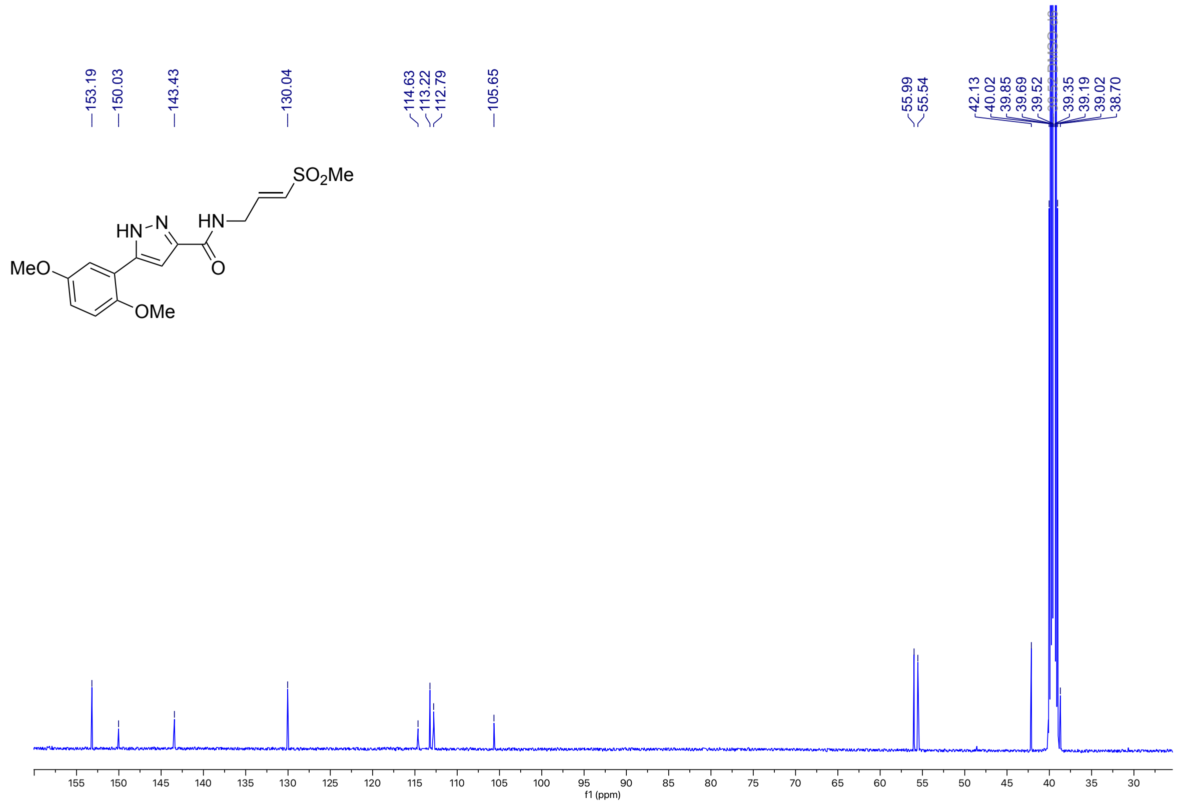

Figure S36: ^{1}H NMR (500 MHz, DMSO-d_6) for **1p**

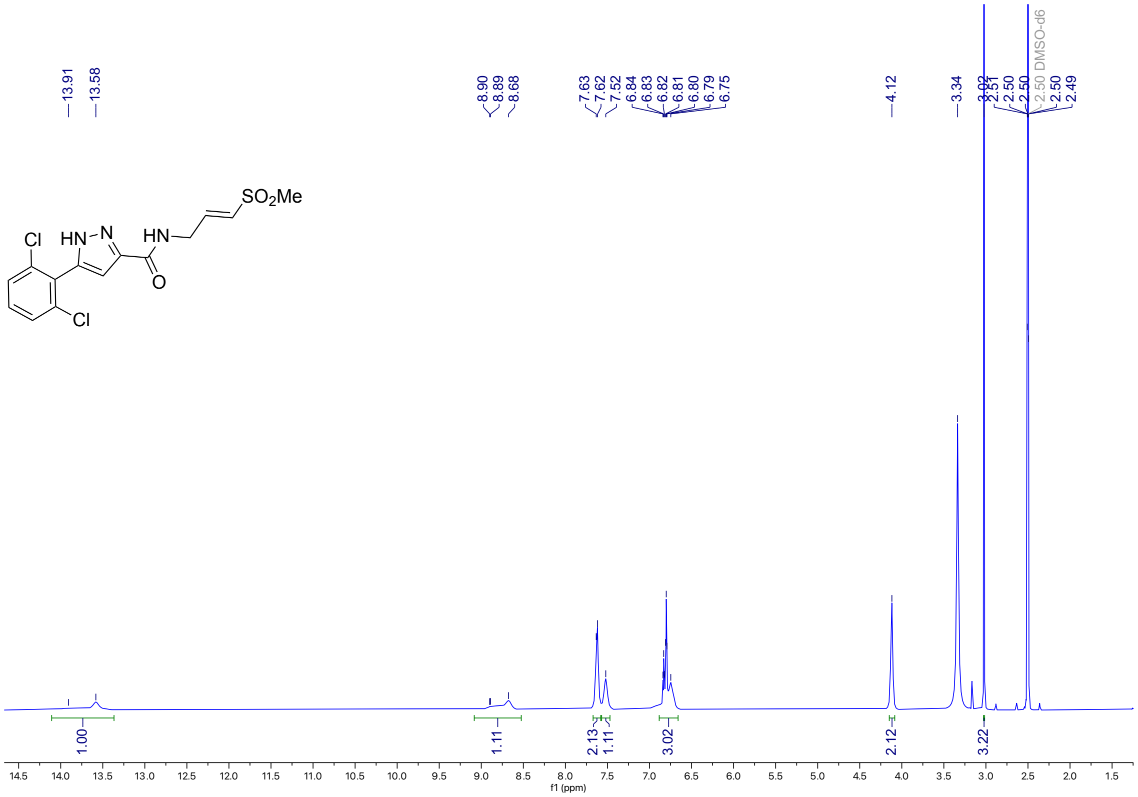

Figure S37: ^{13}C NMR (126 MHz, DMSO-d_6) for **1p**

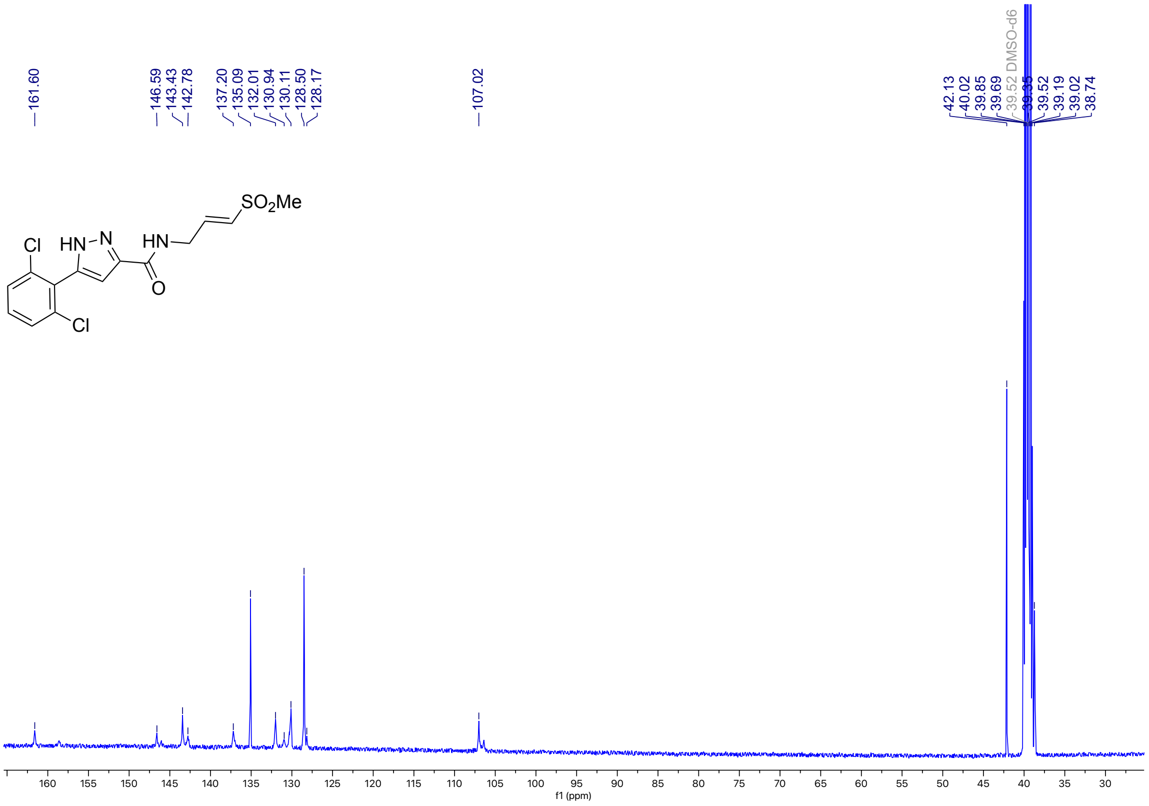

Figure S38: ^{1}H NMR (500 MHz, DMSO-d_6) for **1q**

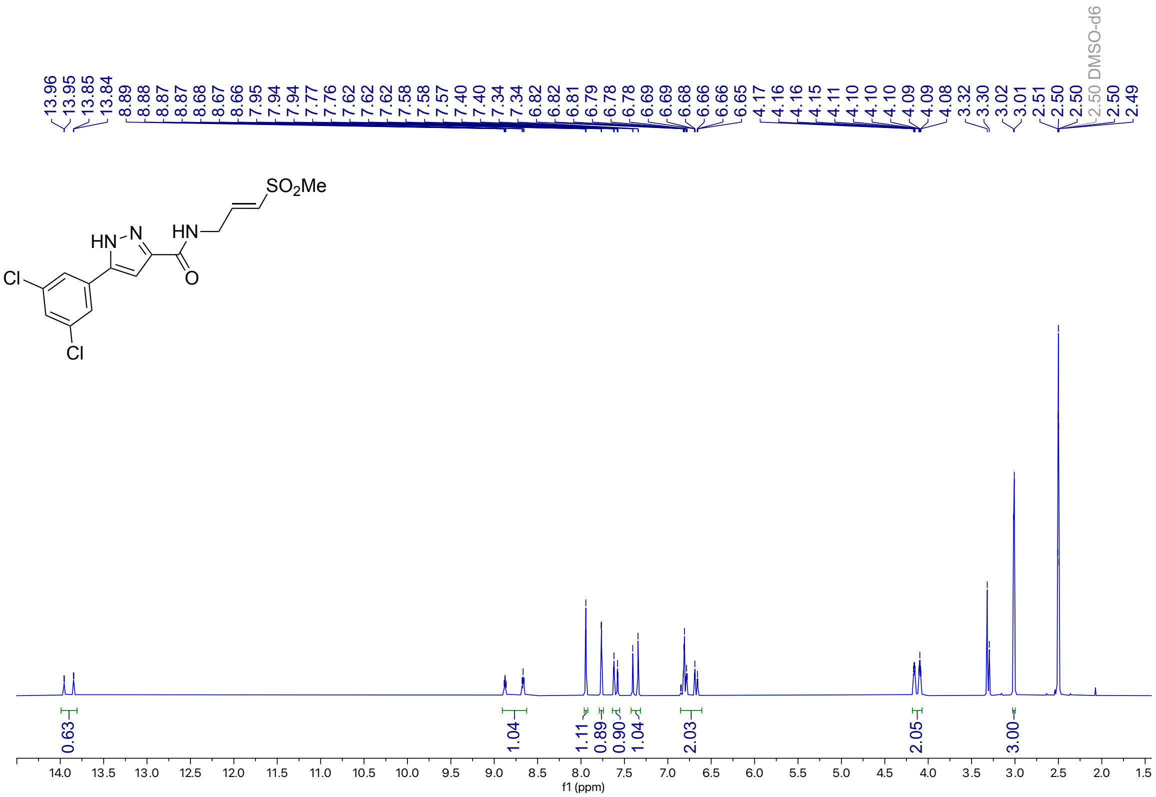

Figure S39: ^{13}C NMR (126 MHz, DMSO-d_6) for **1q**

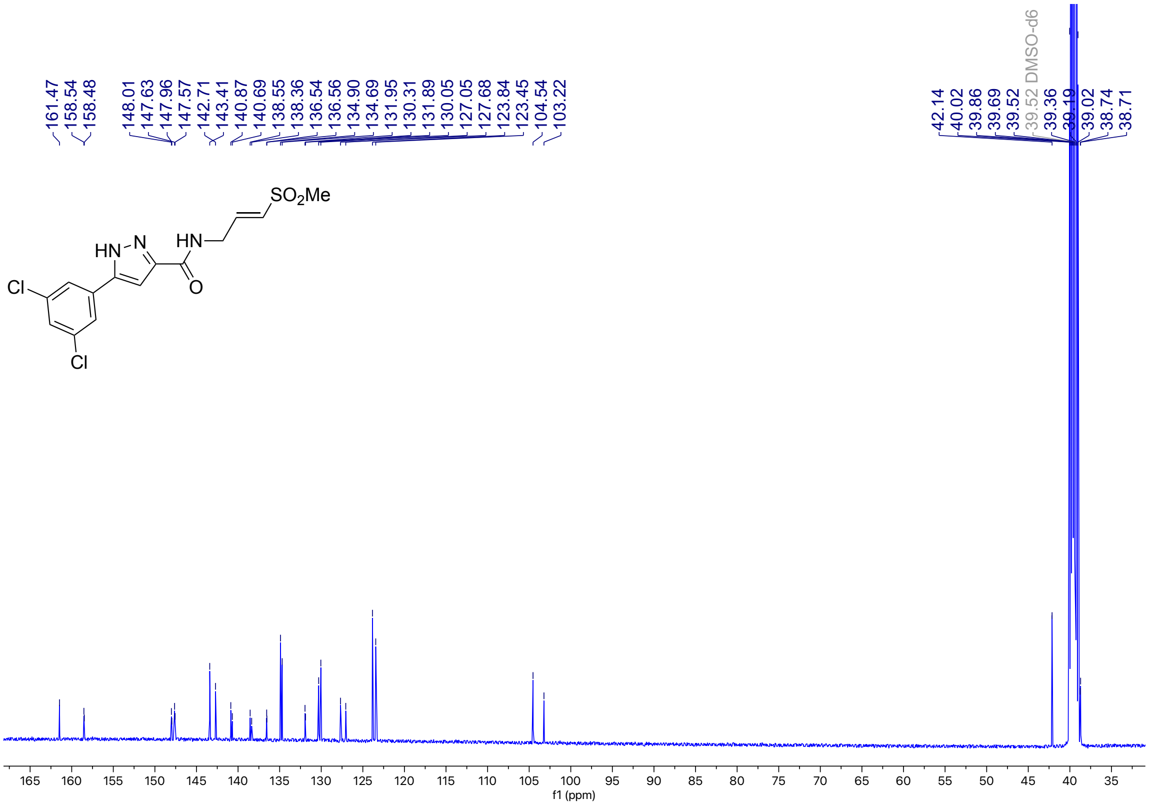

Figure S40: ^{1}H NMR (500 MHz, DMSO-d_6) for **4c**

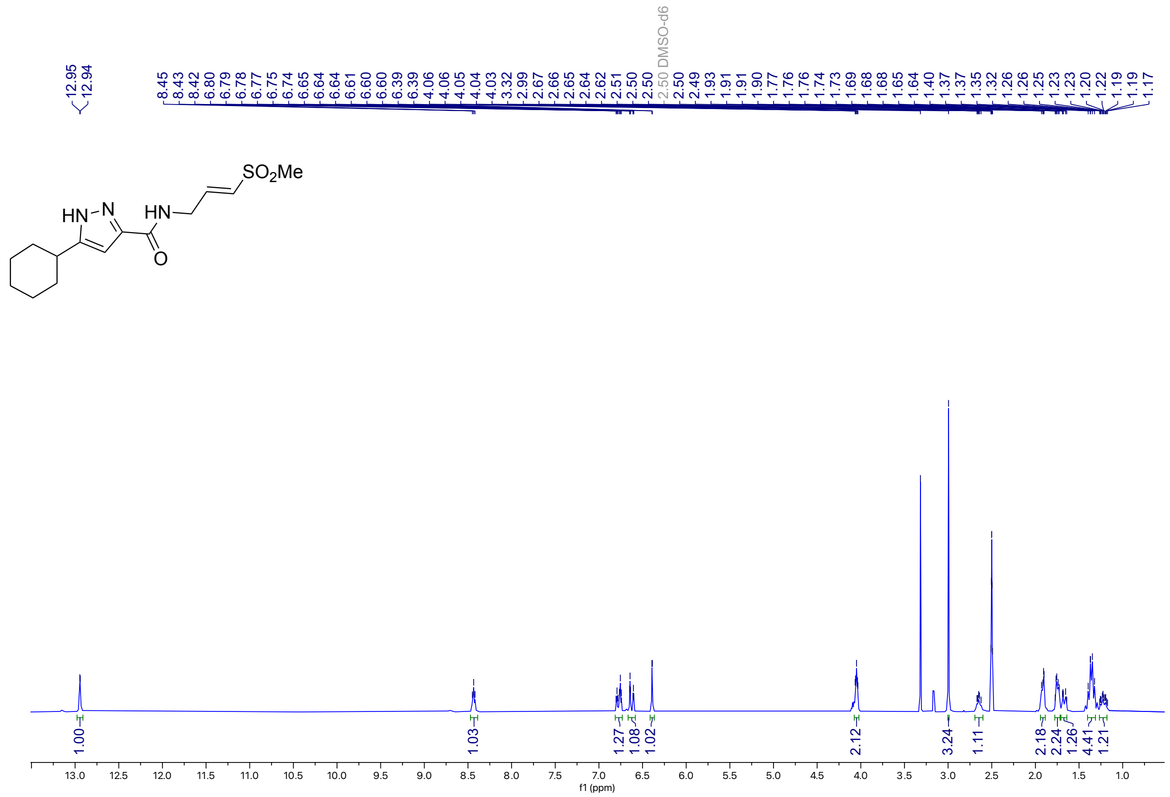

Figure S41: ^{13}C NMR (126 MHz, DMSO-d_6) for **4c**

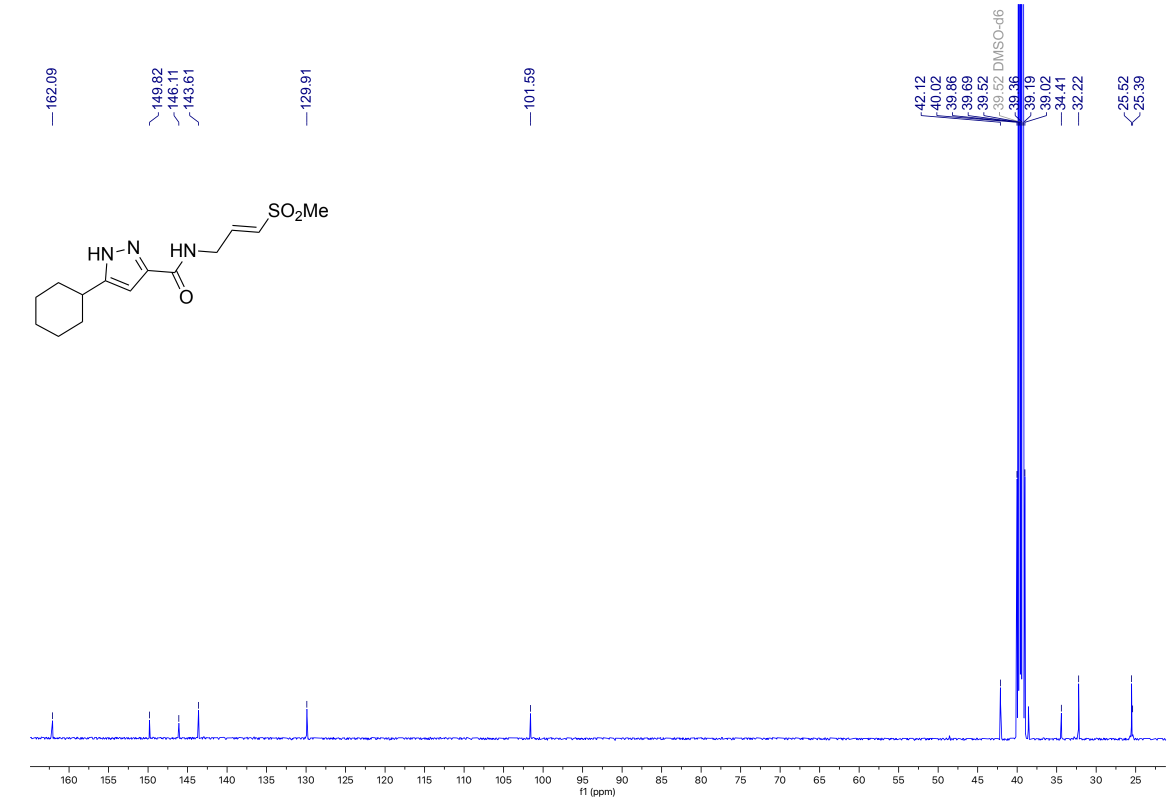

Figure S42: ^{1}H NMR (400 MHz, DMSO-d_6) for **4d**

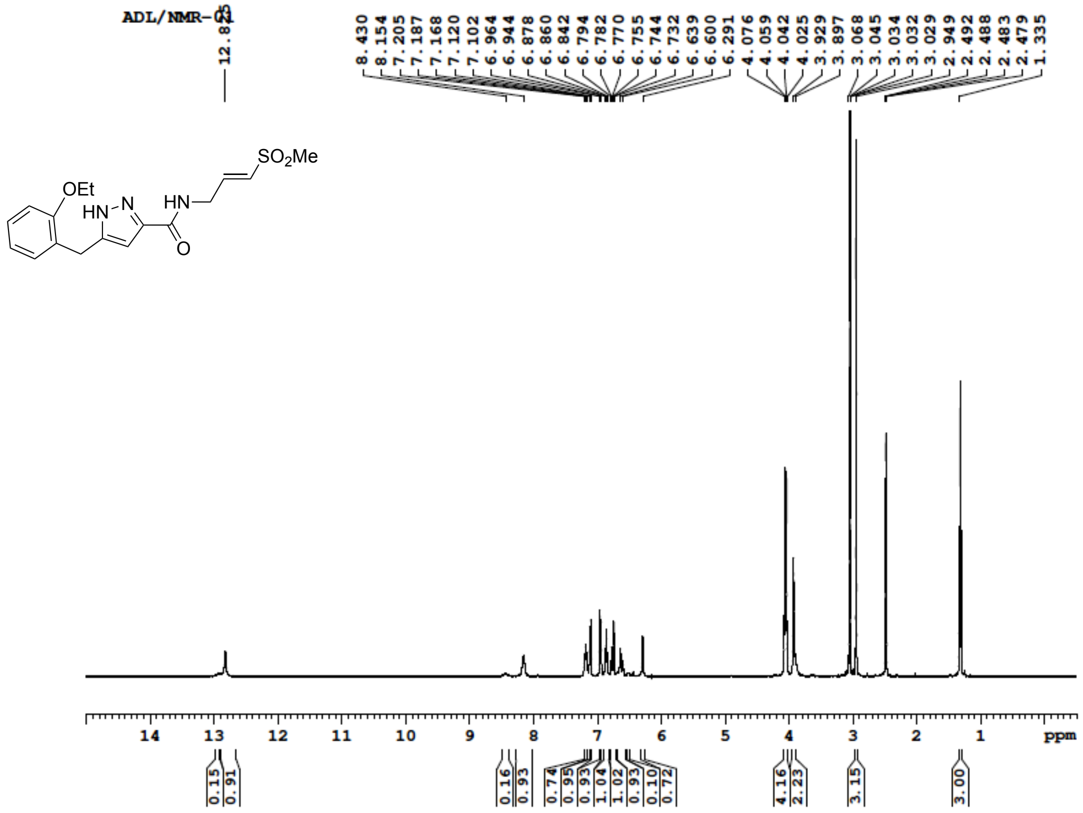

Figure S43: ^{13}C NMR (100 MHz, DMSO-d_6) for **4d**

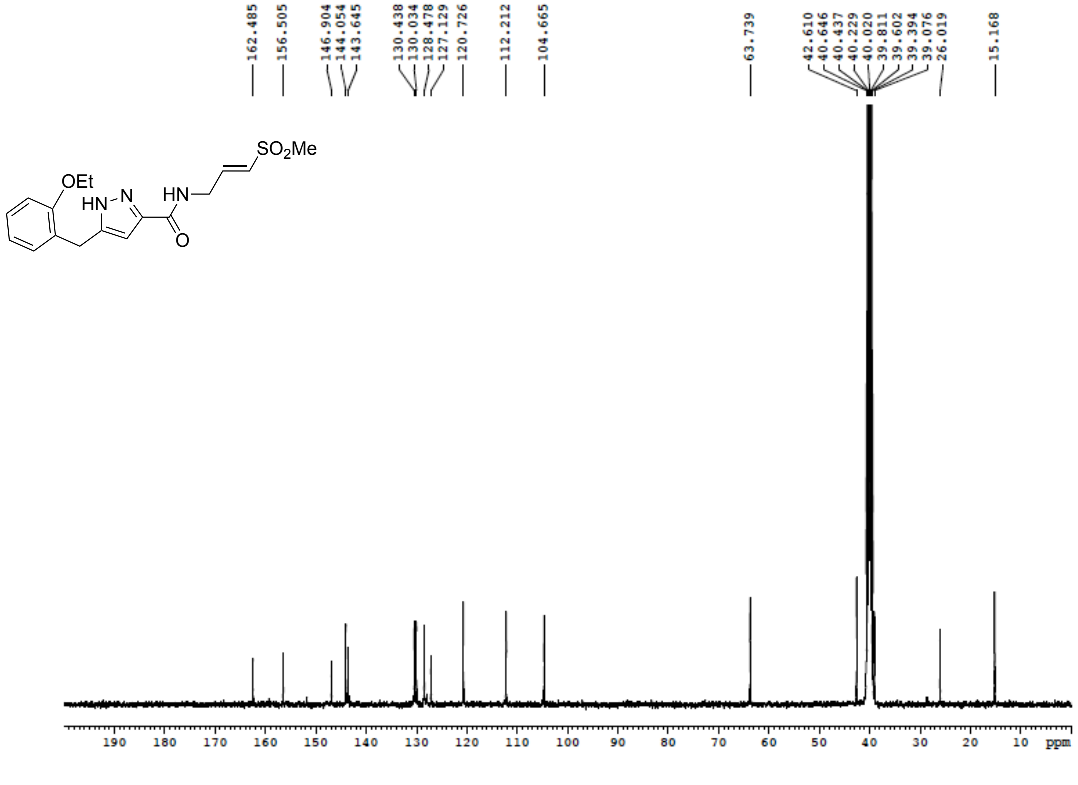

Figure S44: ^{1}H NMR (400 MHz, DMSO-d_6) for **4e**

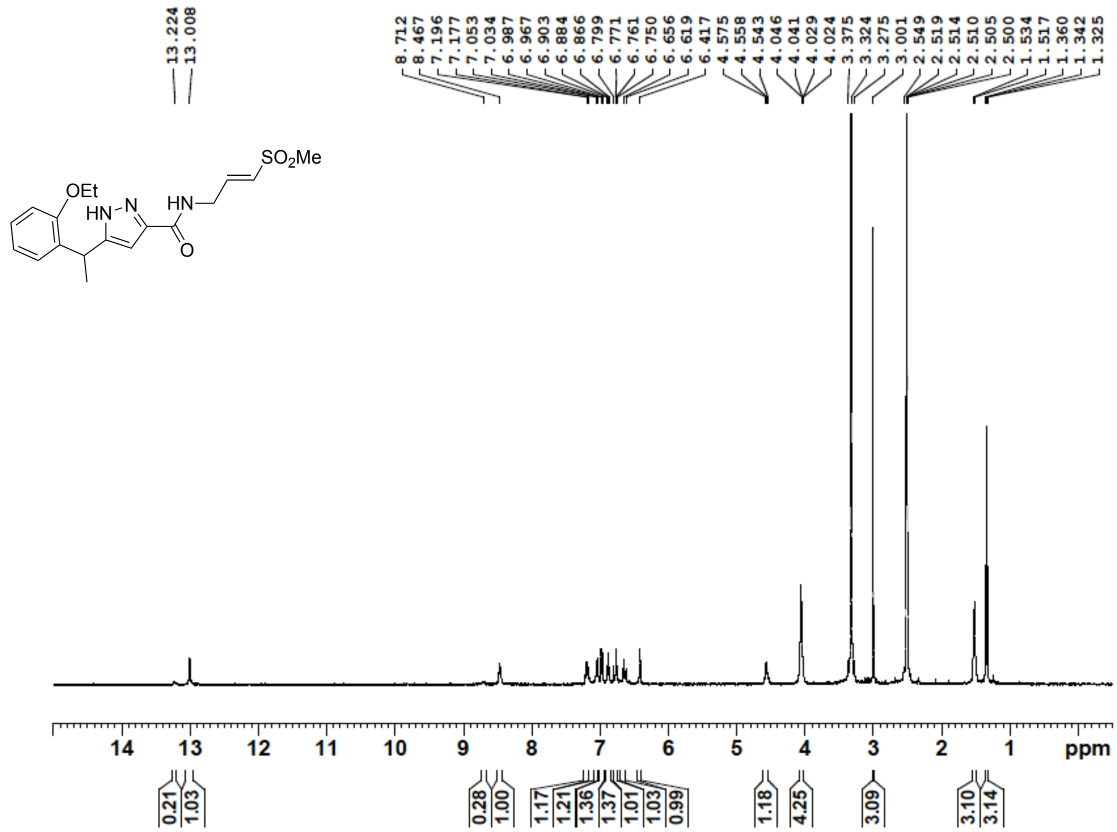

Figure S45: ^{13}C NMR (100 MHz, DMSO-d_6) for **4e**

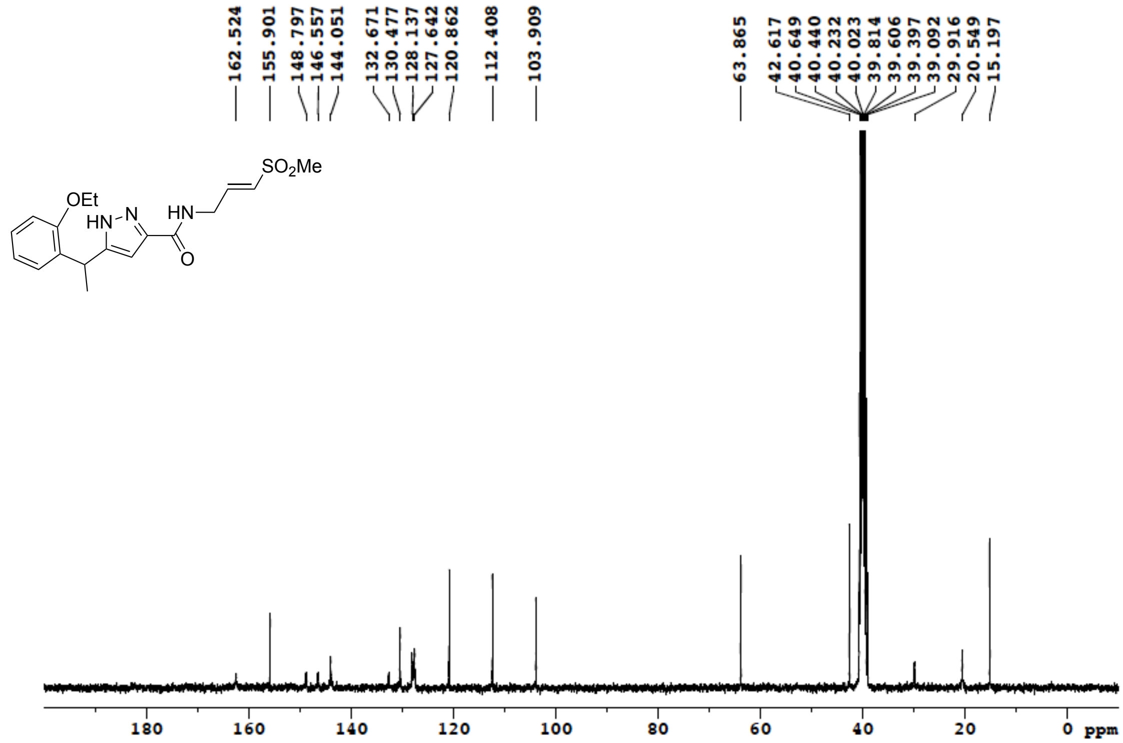

Figure S46: ^{1}H NMR (400 MHz, DMSO-d_6) for **4g**

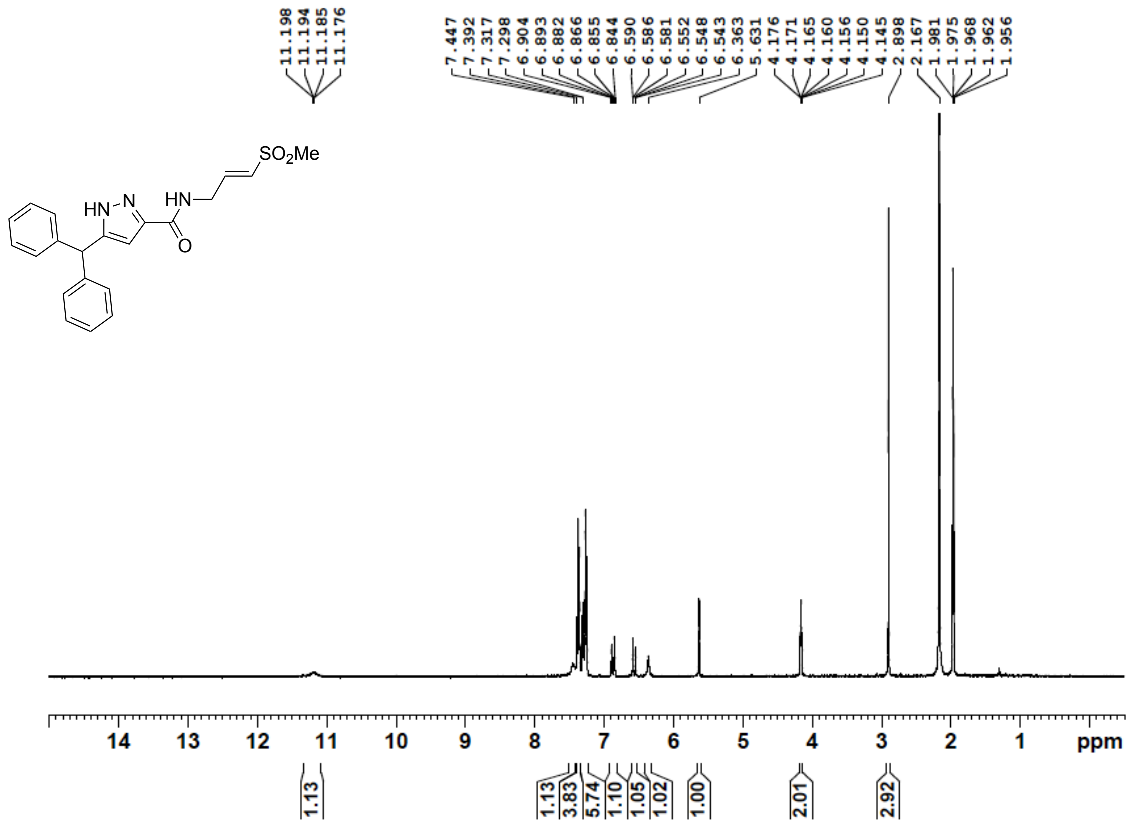

Figure S47: ^{13}C NMR (100 MHz, DMSO-d_6) for **4g**

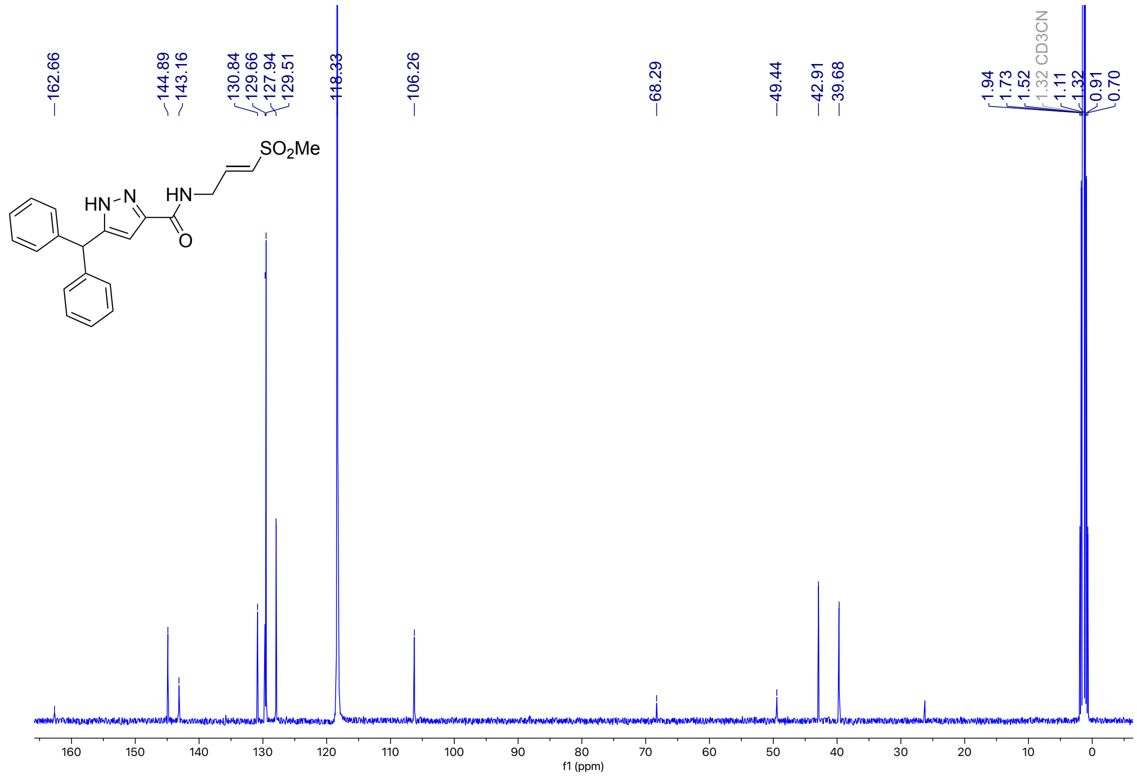

Figure S48: ^{1}H NMR (500 MHz, DMSO-d_6) for **4h**

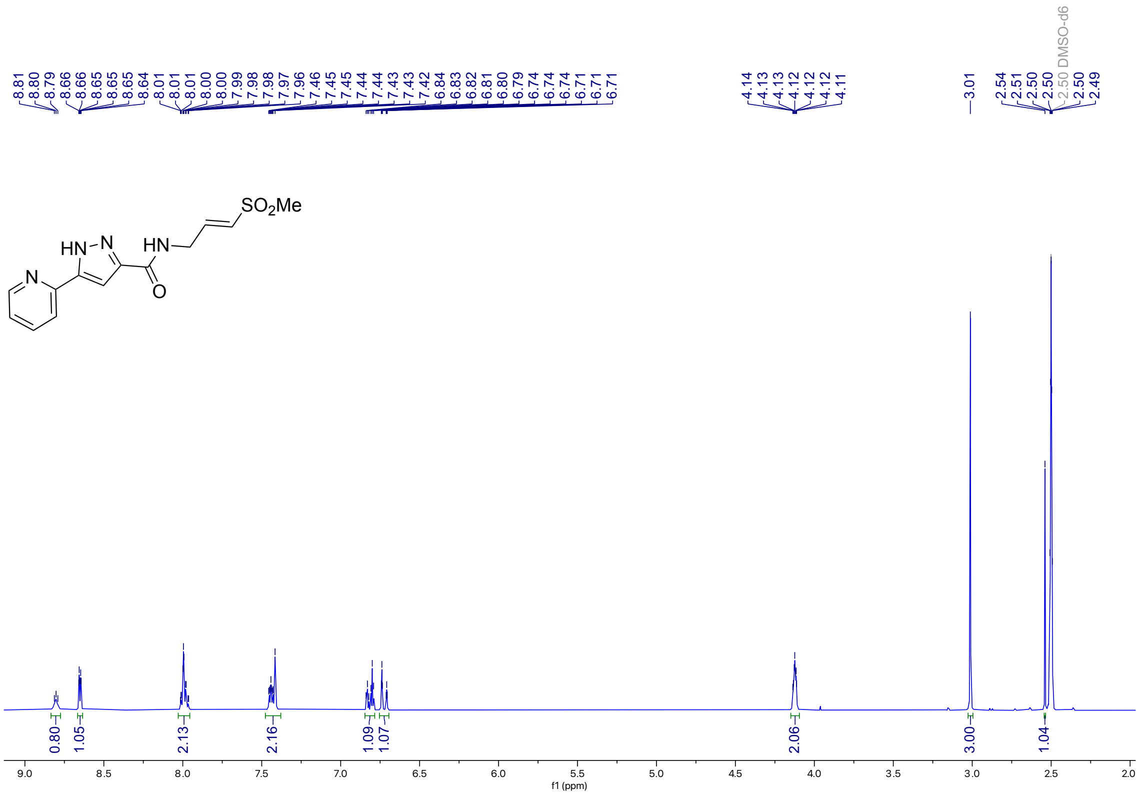

Figure S49: ^{13}C NMR (126 MHz, DMSO-d_6) for **4h**

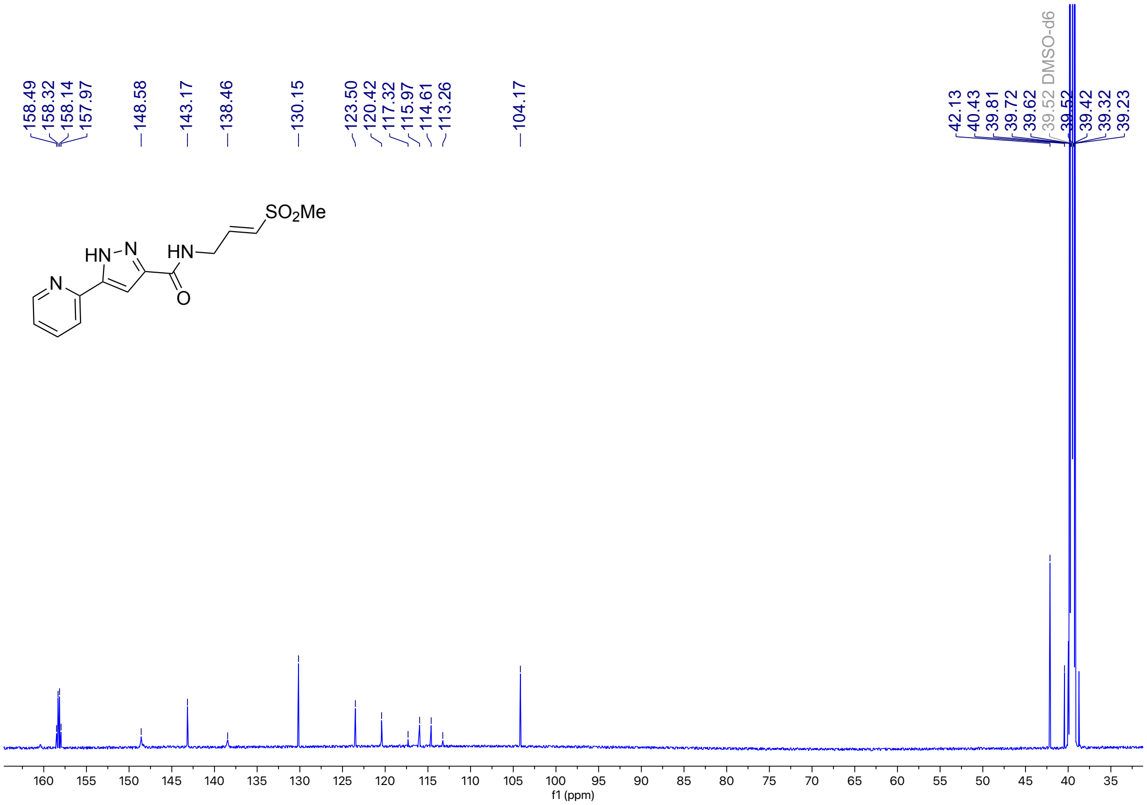

Figure S50: ^{1}H NMR (500 MHz, DMSO-d_6) for **4i**

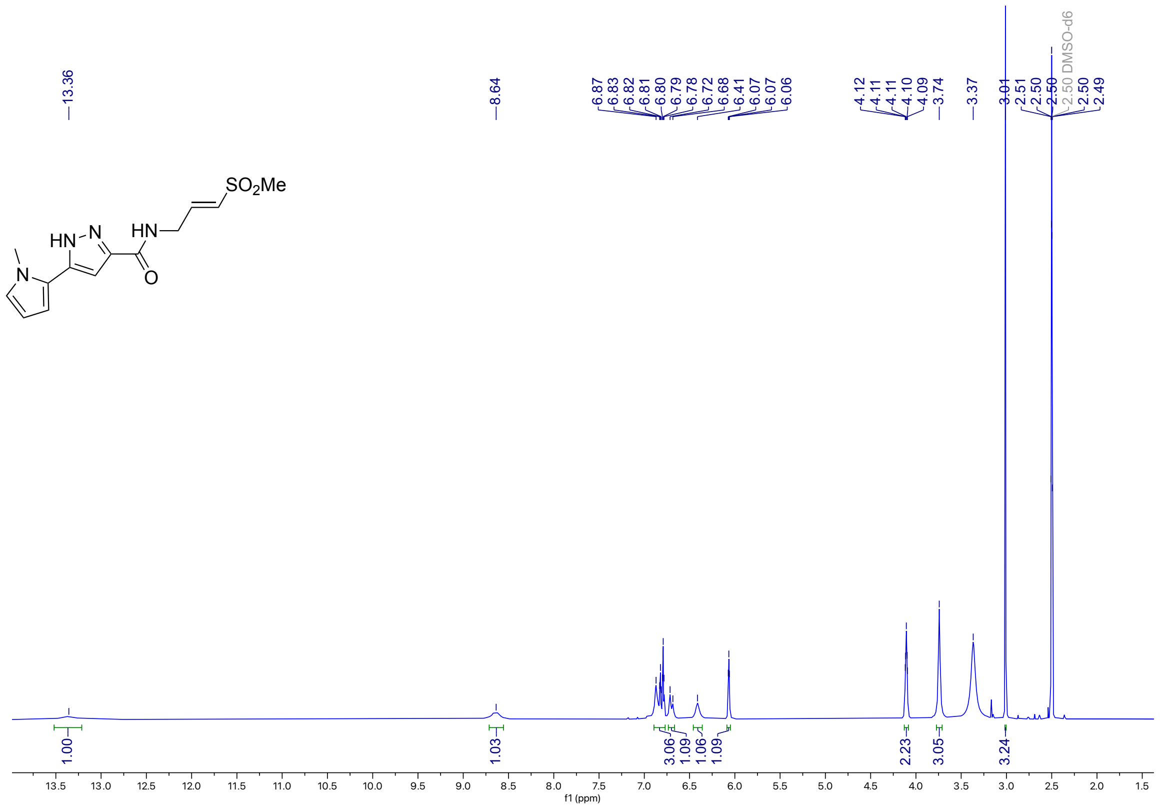

Figure S51: ^{13}C NMR (214 MHz, DMSO-d_6) for **4i**

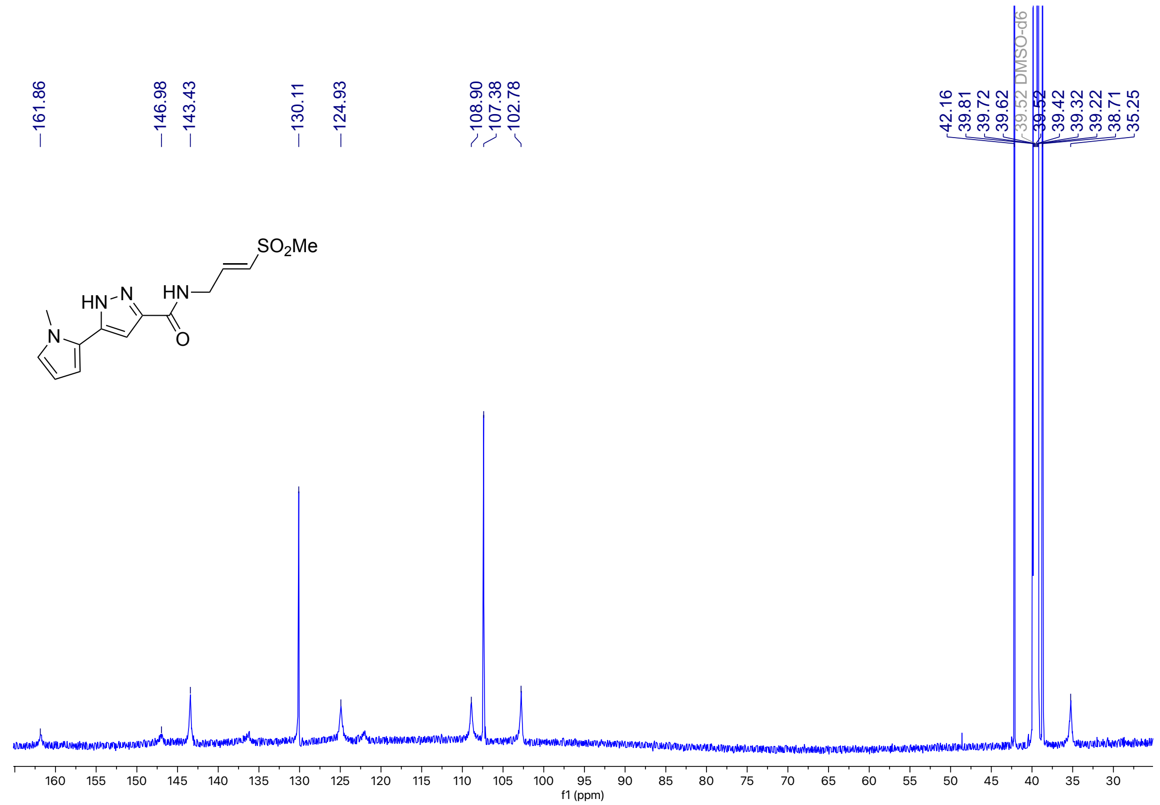

Figure S52: ^{1}H NMR (400 MHz, DMSO-d_6) for **5**

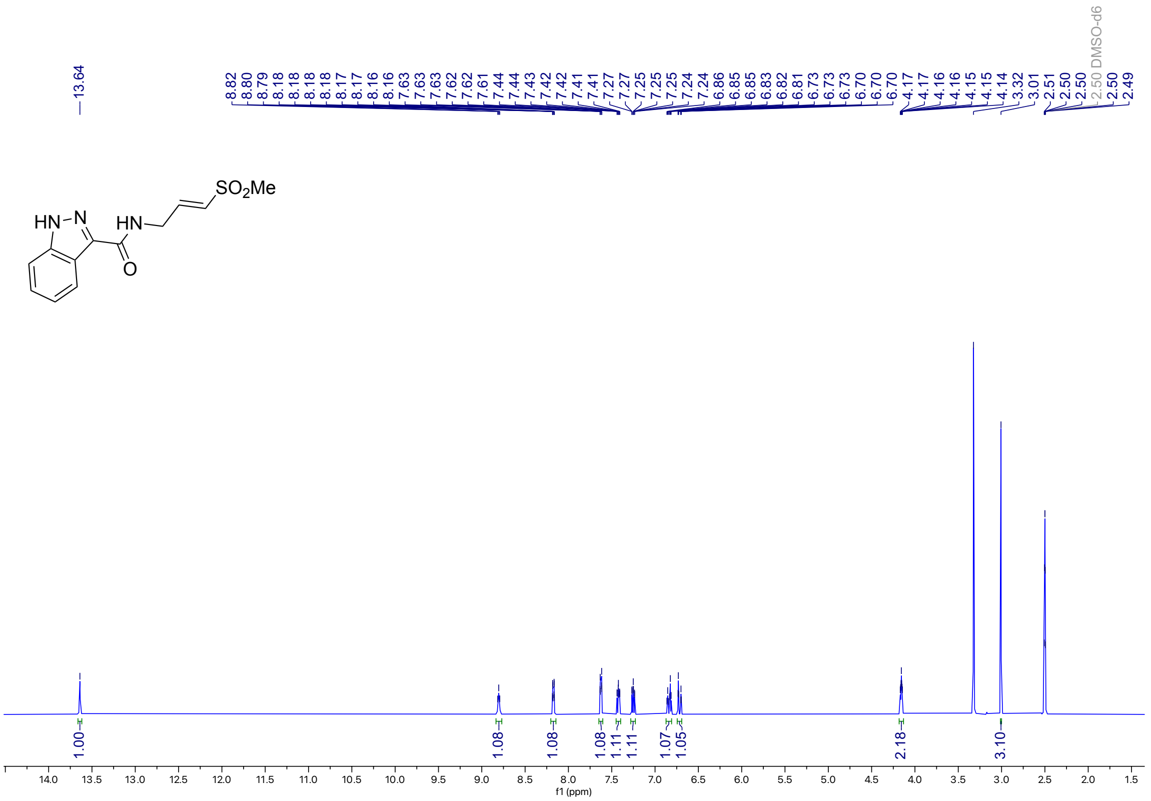

Figure S53: ^{13}C NMR (100 MHz, DMSO-d_6) for **5**

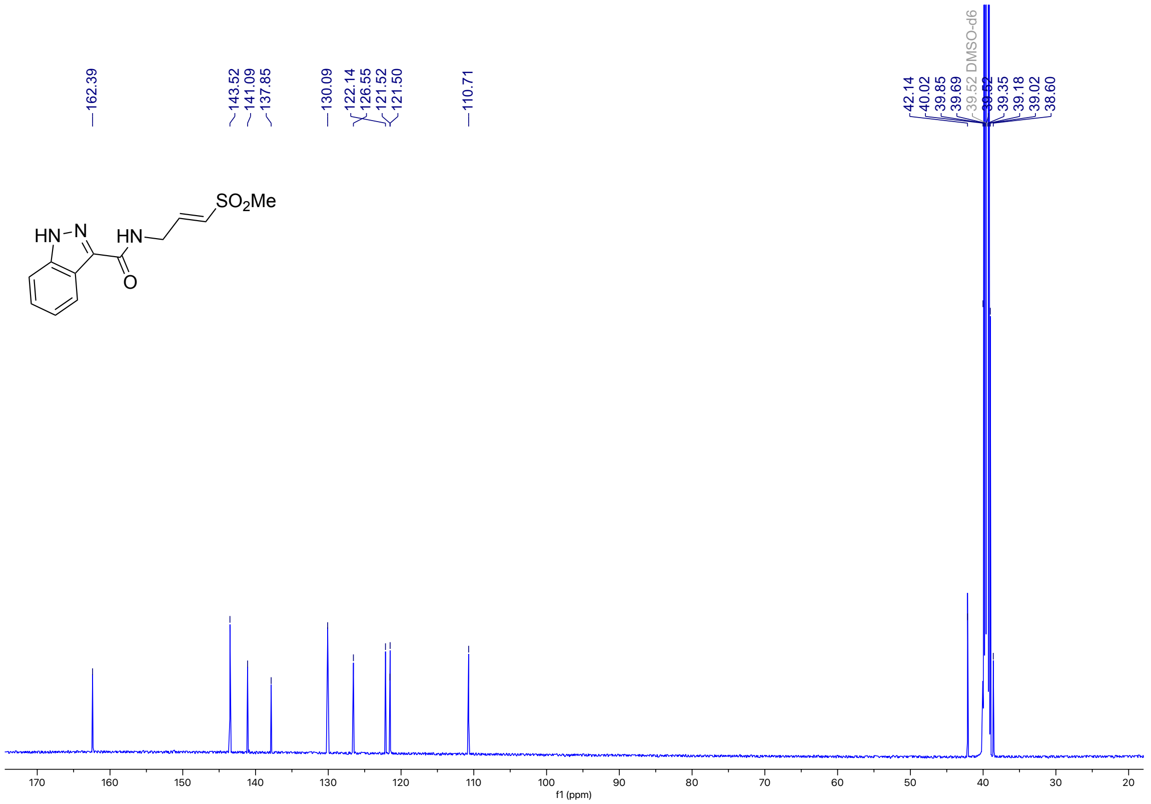

Figure S54: ^{1}H NMR (500 MHz, DMSO-d_6) for **6**

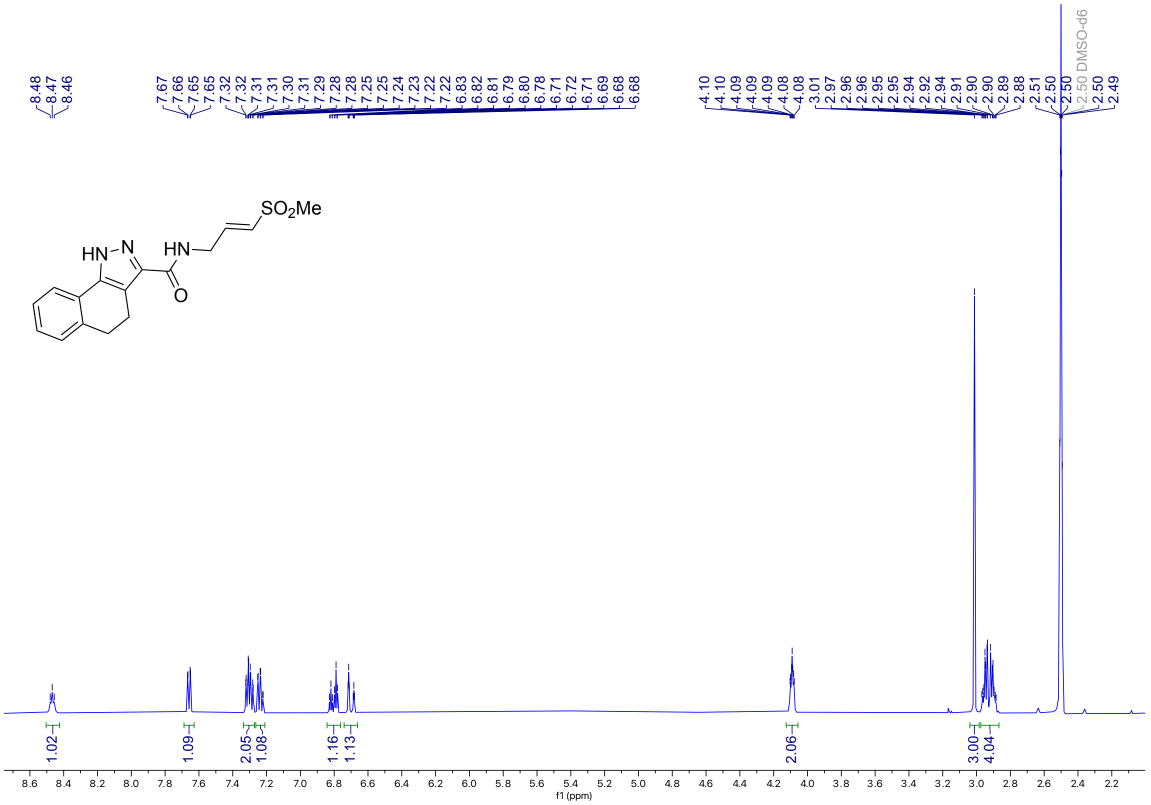

Figure S55: ^{13}C NMR (214 MHz, DMSO-d_6) for **6**

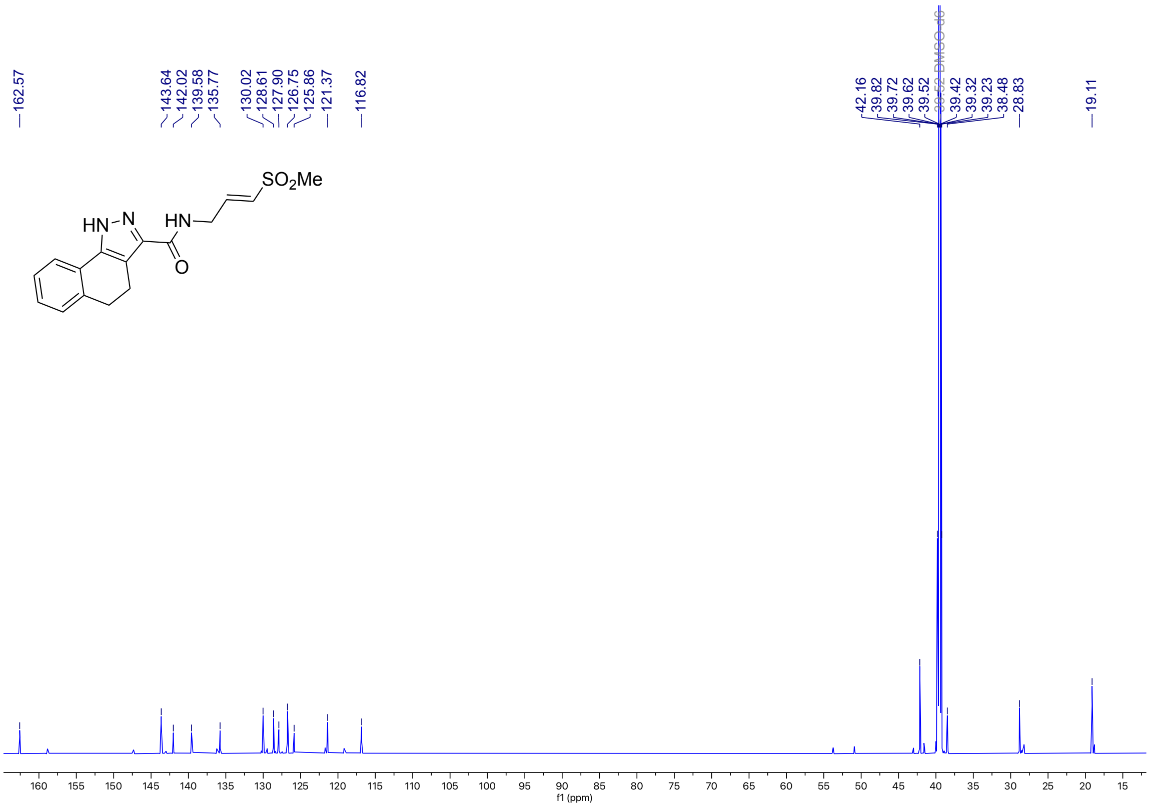

Figure S56: ^{1}H NMR (500 MHz, DMSO-d_6) for **7a**

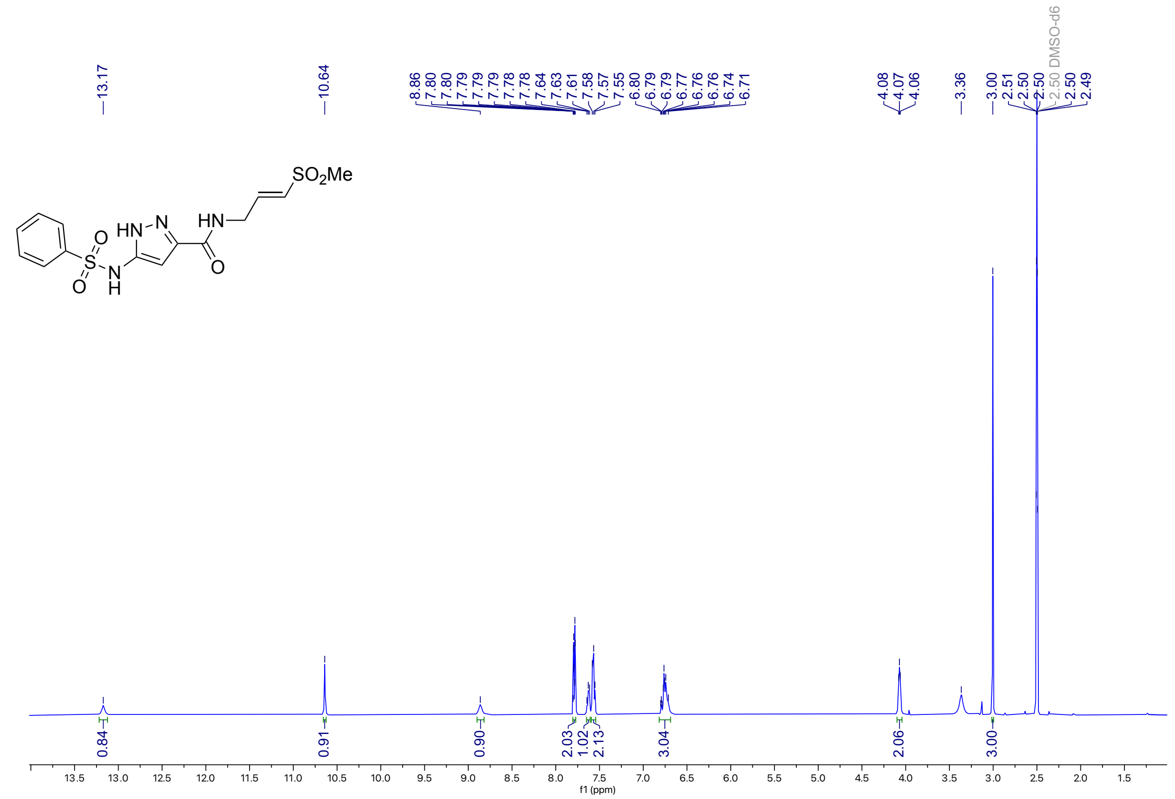

Figure S57: ^{13}C NMR (126 MHz, DMSO-d_6) for **7a**

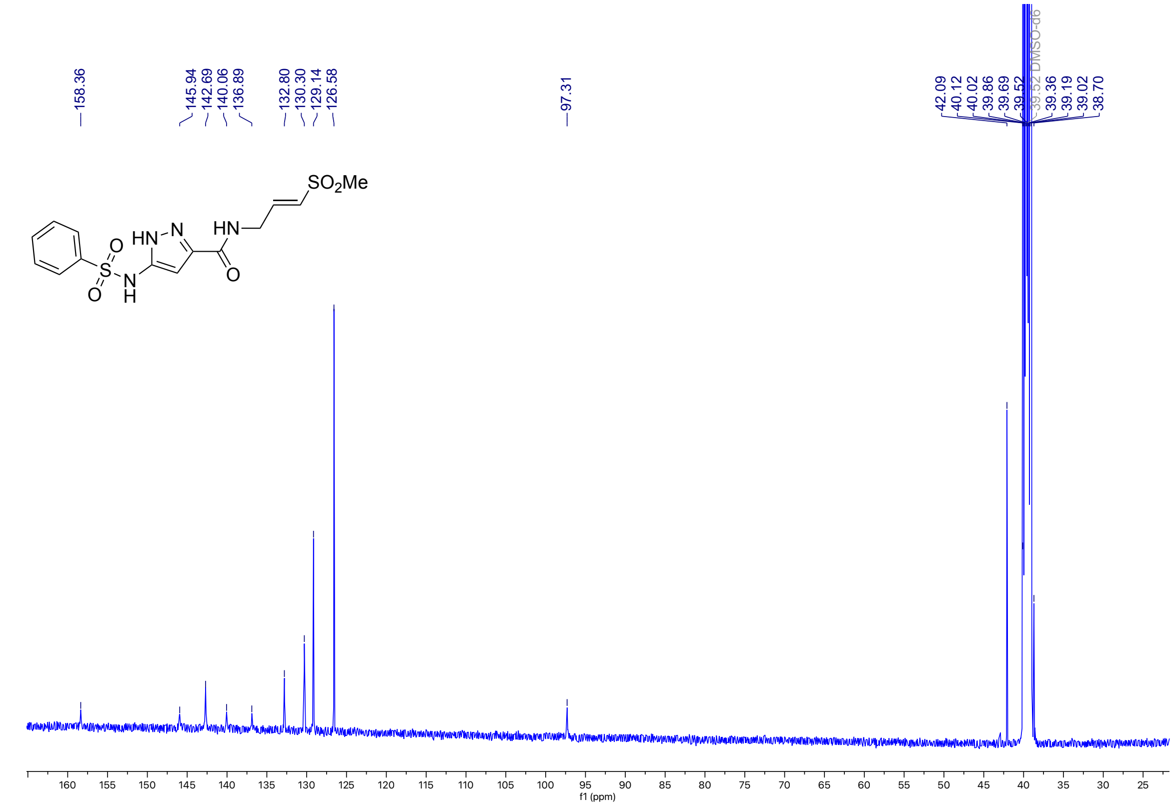

Figure S58: ^{1}H NMR (400 MHz, DMSO-d_6) for **7b**

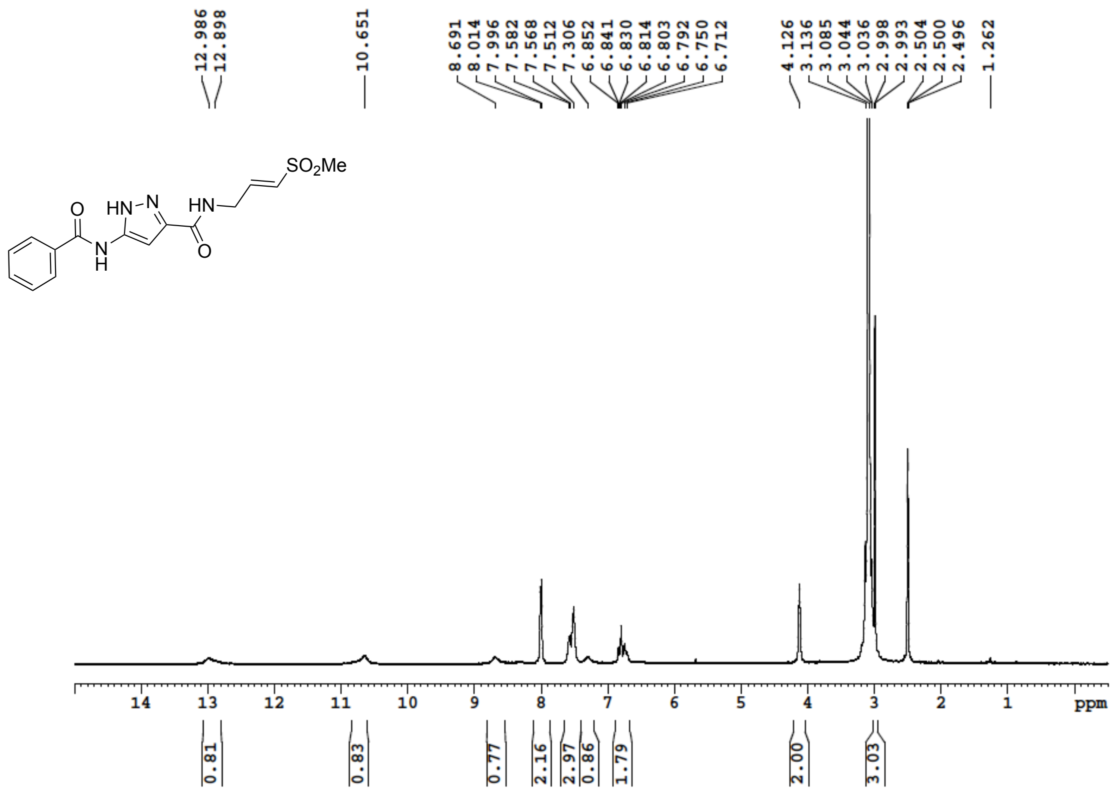

Figure S59: ^{13}C NMR (100 MHz, DMSO-d_6) for **7b**

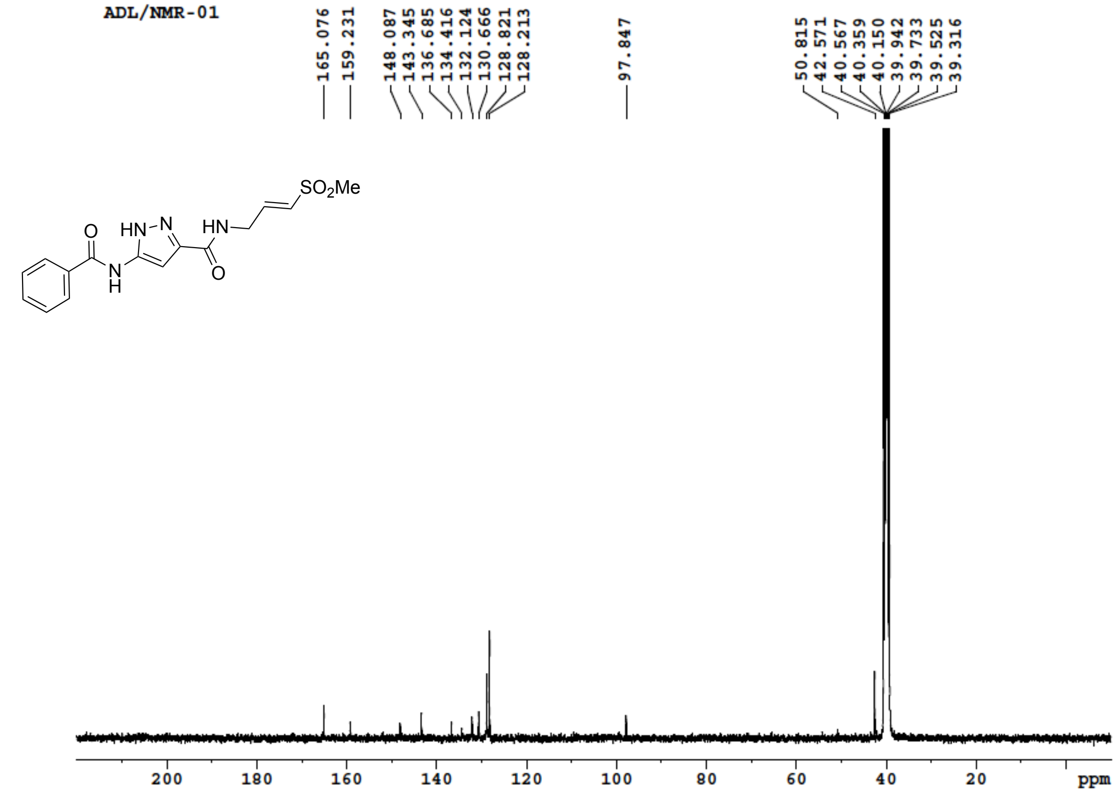

Figure S60: ^{1}H NMR (400 MHz, DMSO-d_6) for **7c**

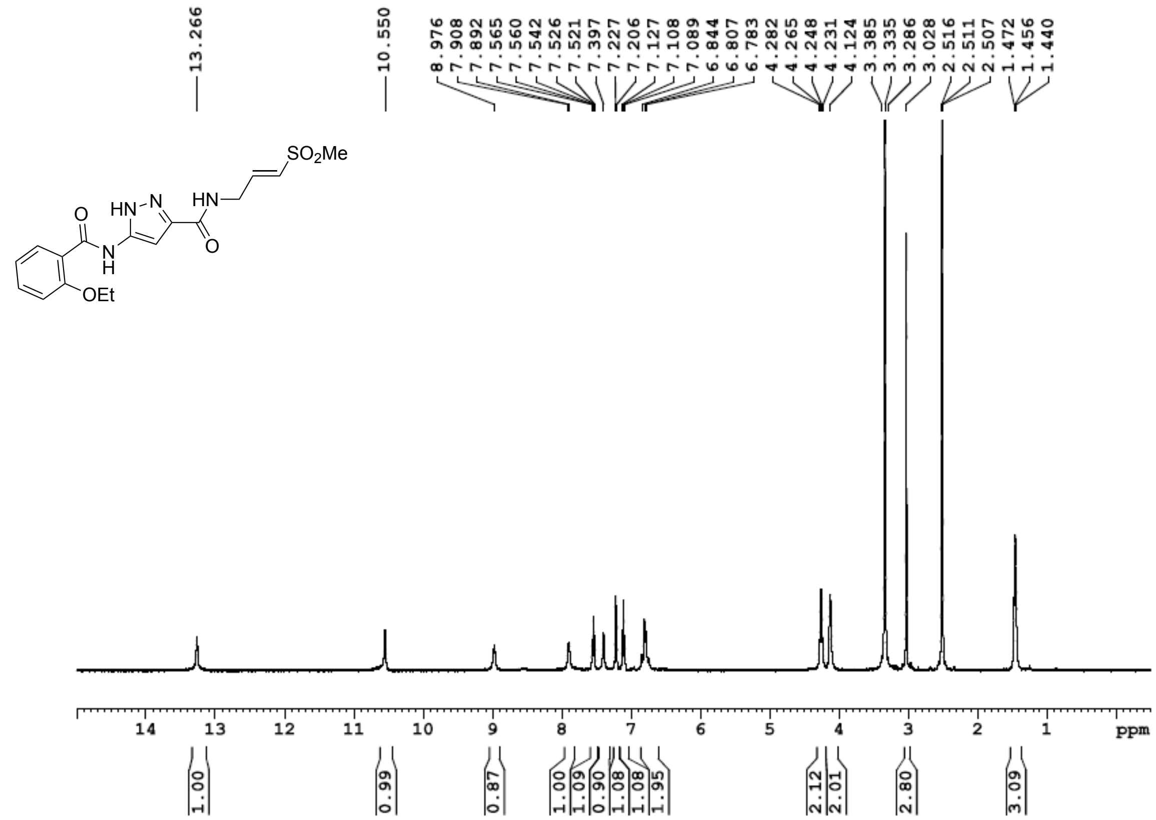

Figure S61: ^{13}C NMR (100 MHz, DMSO-d_6) for **7c**

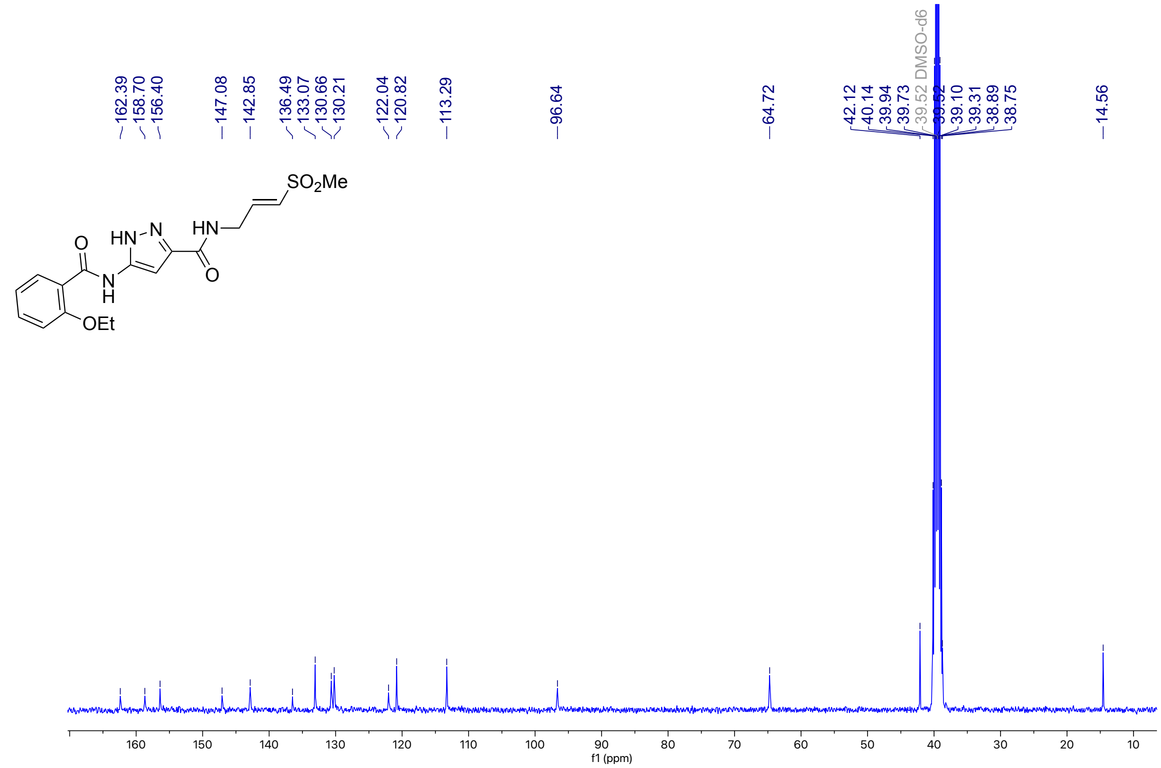

Figure S62: ^{1}H NMR (400 MHz, DMSO-d_6) for **7d**

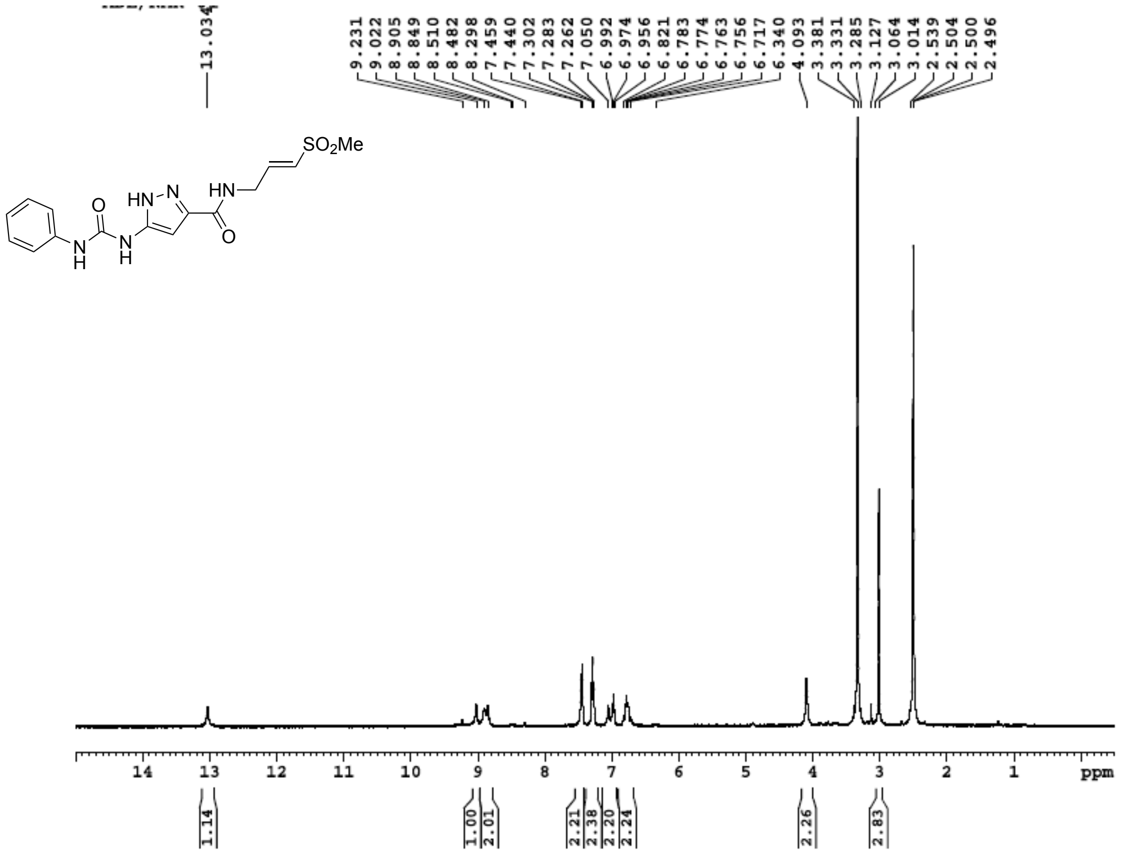

Figure S63: ^{1}H NMR (101 MHz, DMSO-d_6) for **7d**

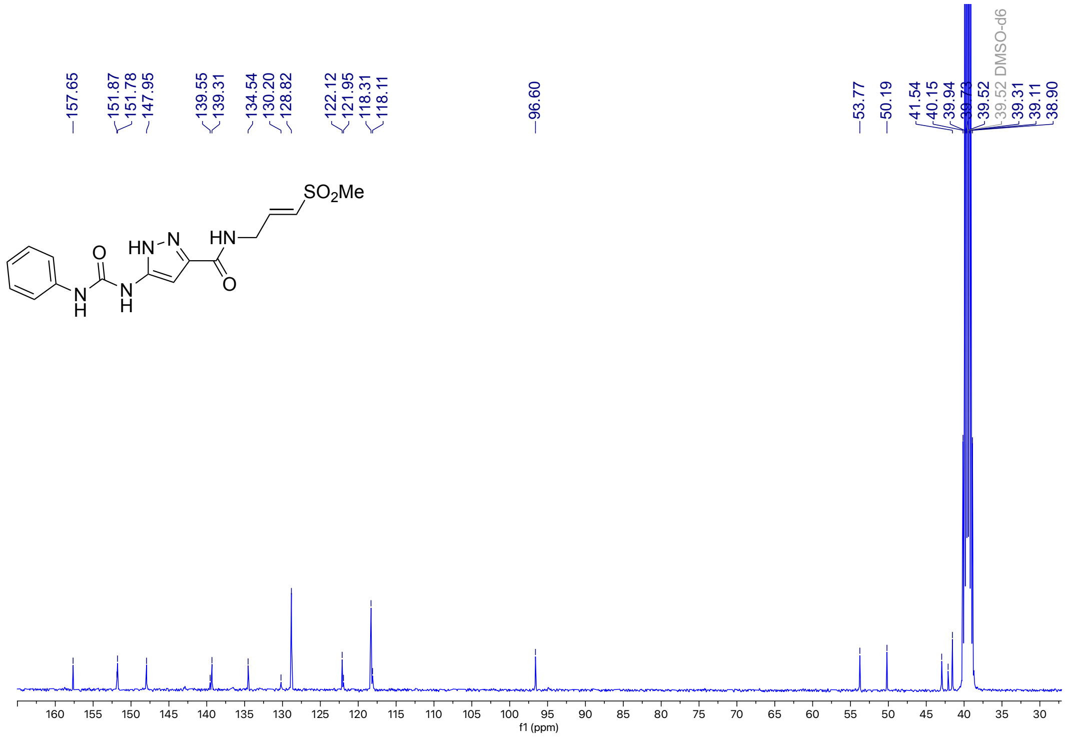

Figure S64: ^{1}H NMR (400 MHz, DMSO-d_6) for **7e**

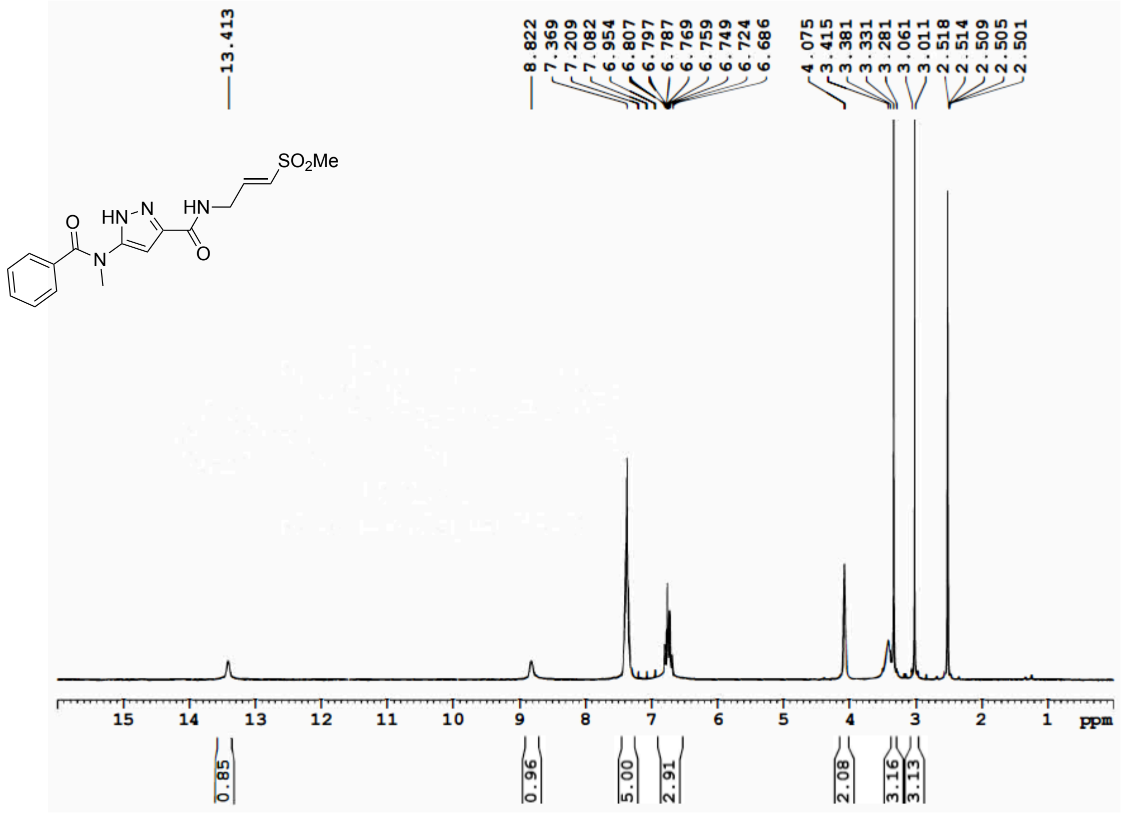

Figure S65: ^{13}C NMR (100 MHz, DMSO-d_6) for **7e**

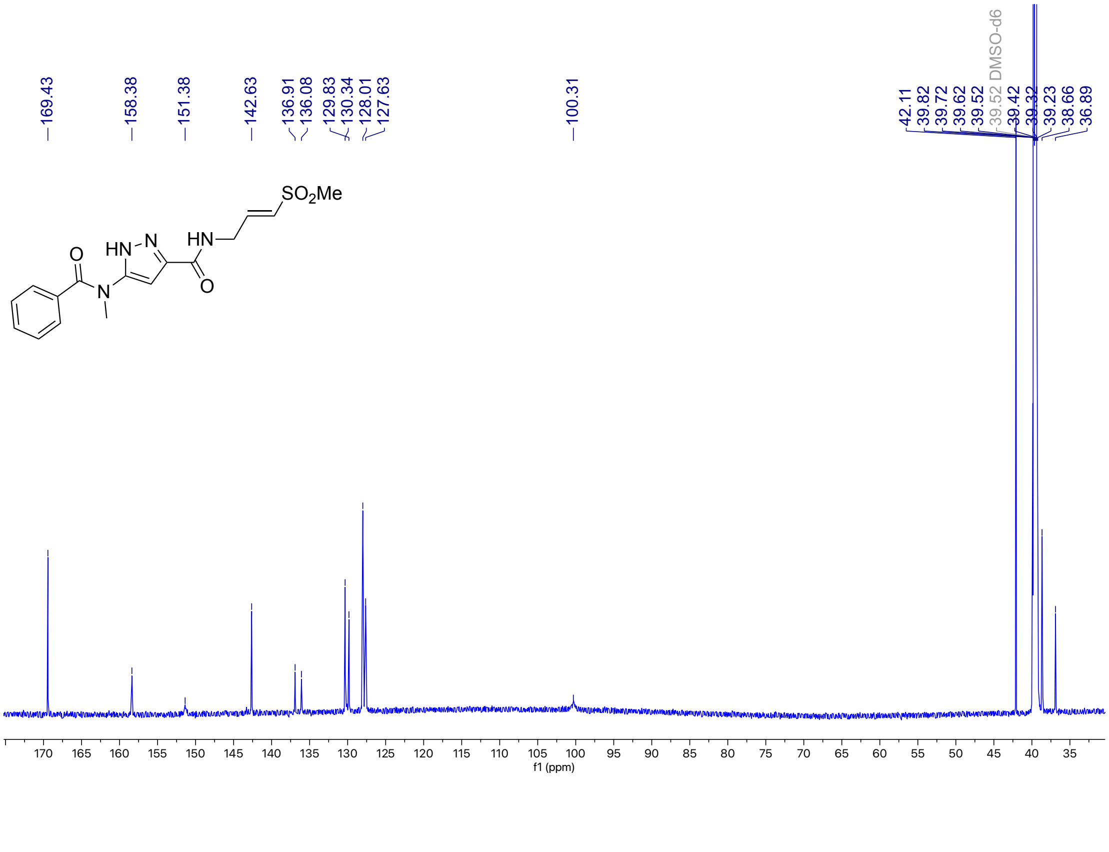

Figure S66: ^{1}H NMR (400 MHz, DMSO-d_6) for **7f**

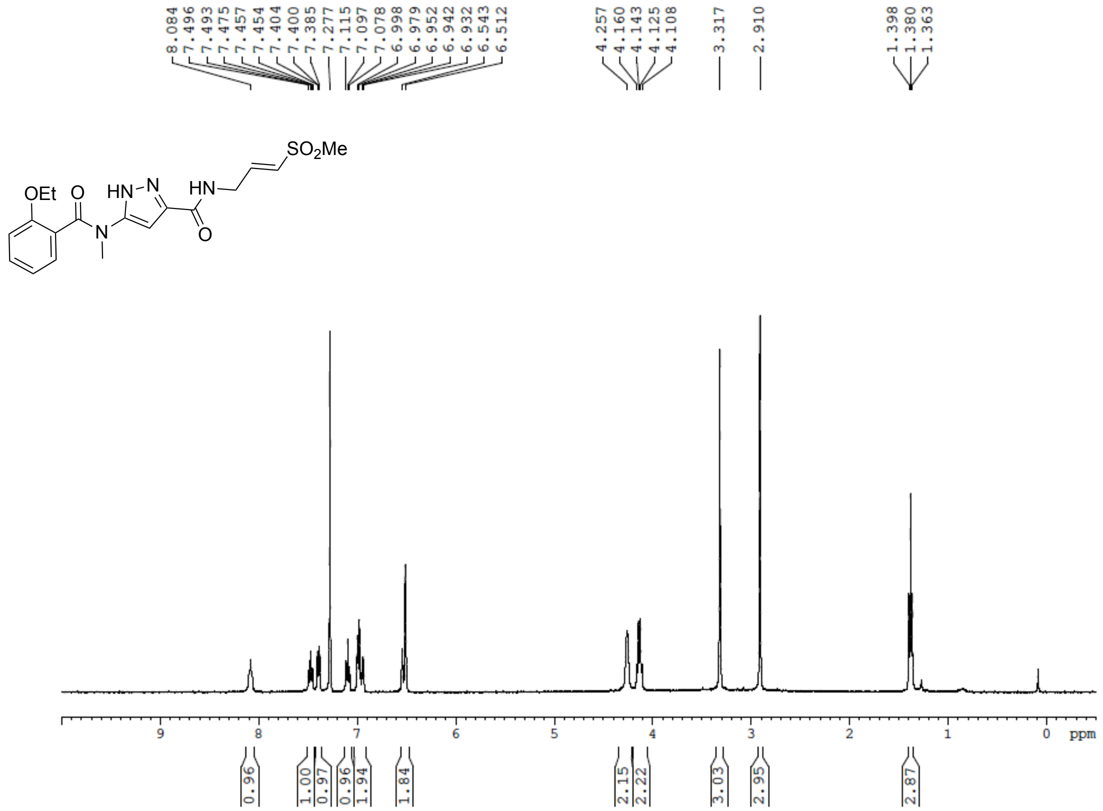

Figure S67: ^{13}C NMR (100 MHz, DMSO-d_6) for **7f**

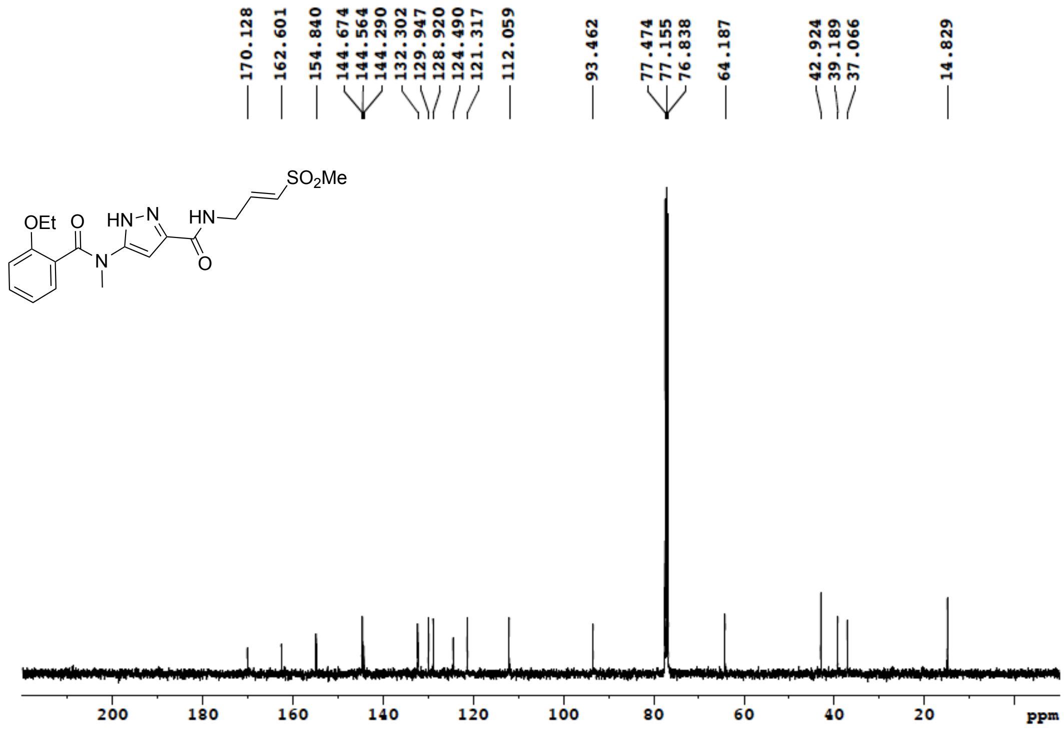

Figure S68: ^{1}H NMR (400 MHz, DMSO-d_6) for **8a**

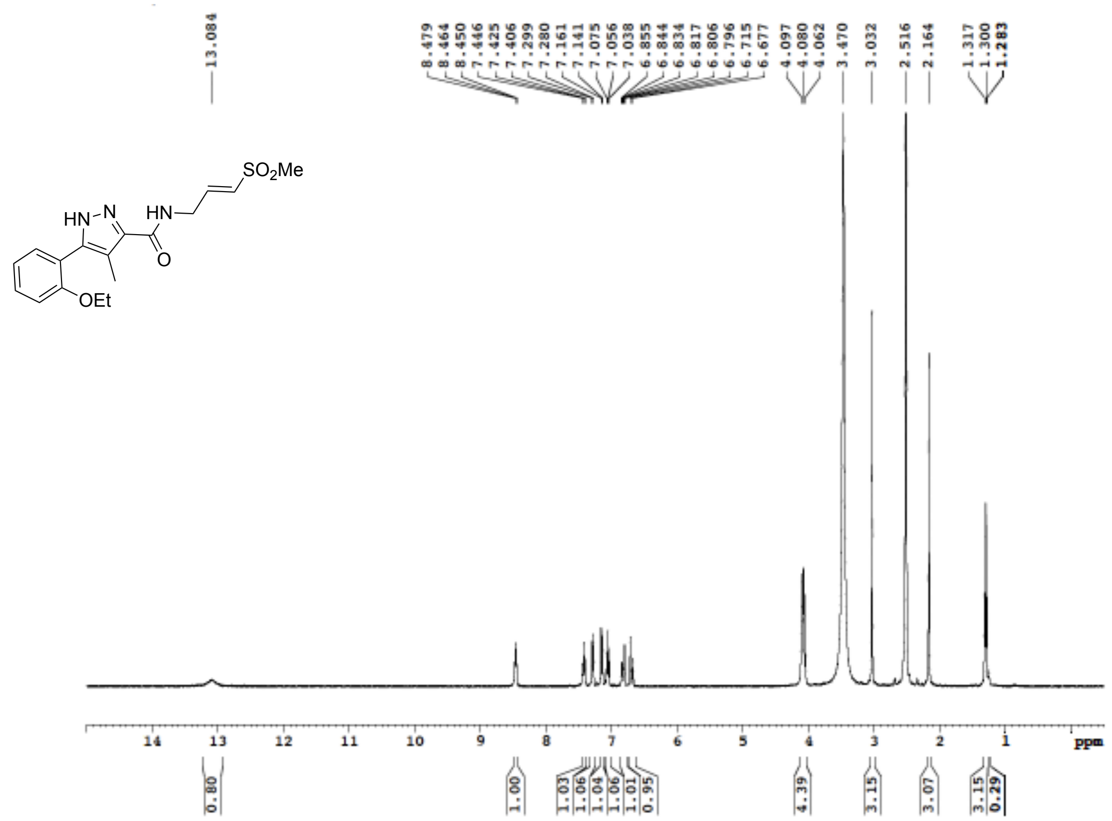

Figure S69: ^{13}C NMR (100 MHz, DMSO-d_6) for **8a**

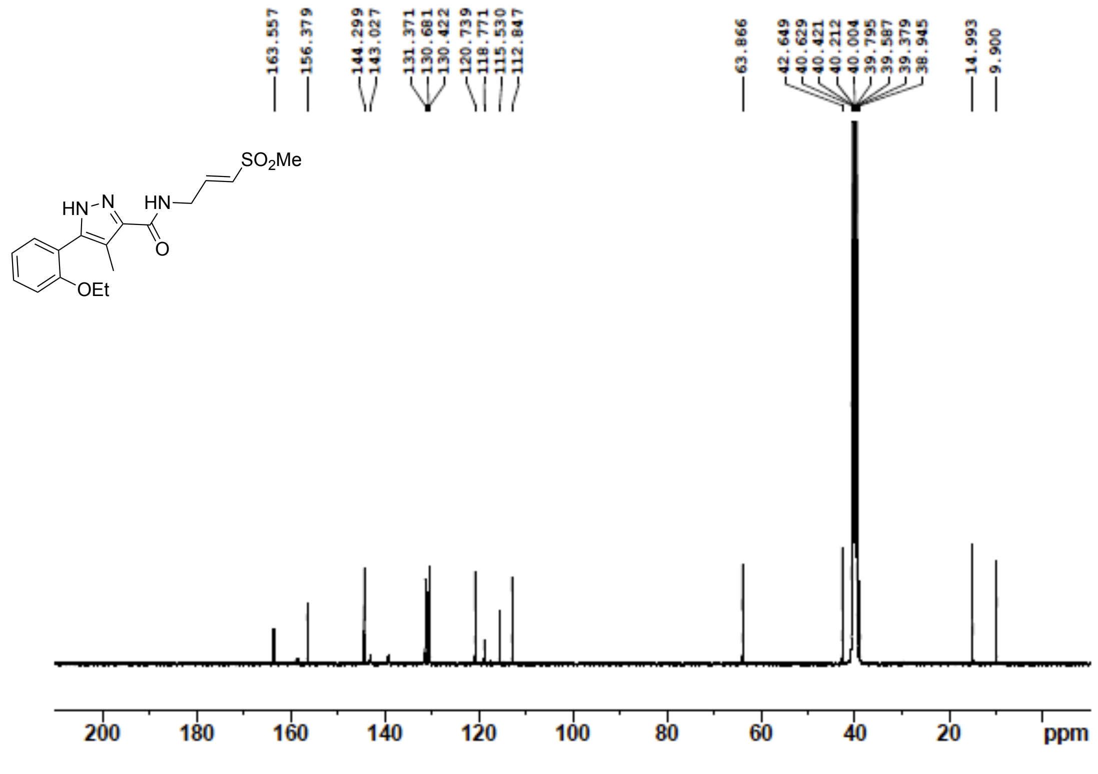

Figure S70: ^{1}H NMR (400 MHz, DMSO-d_6) for **8b**

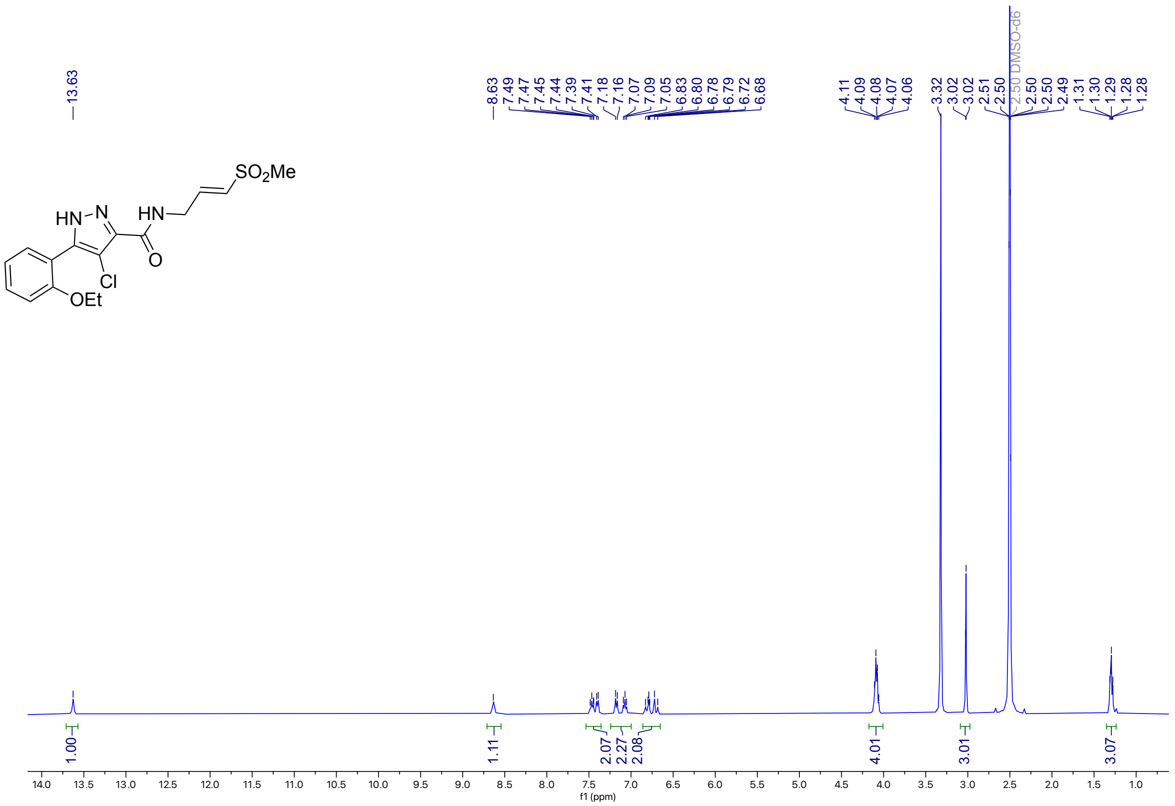

Figure S71: ^{13}C NMR (100 MHz, DMSO-d_6) for **8b**

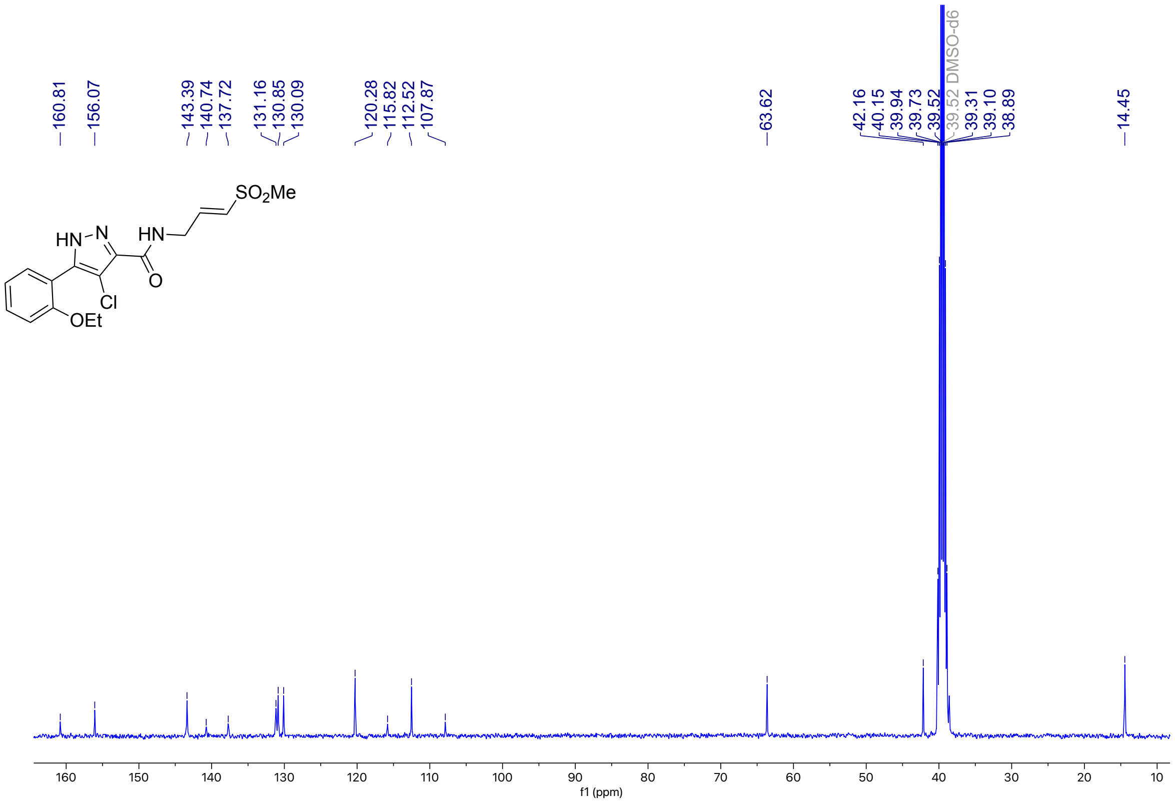

Figure S72: ^{1}H NMR (400 MHz, DMSO-d_6) for **8c**

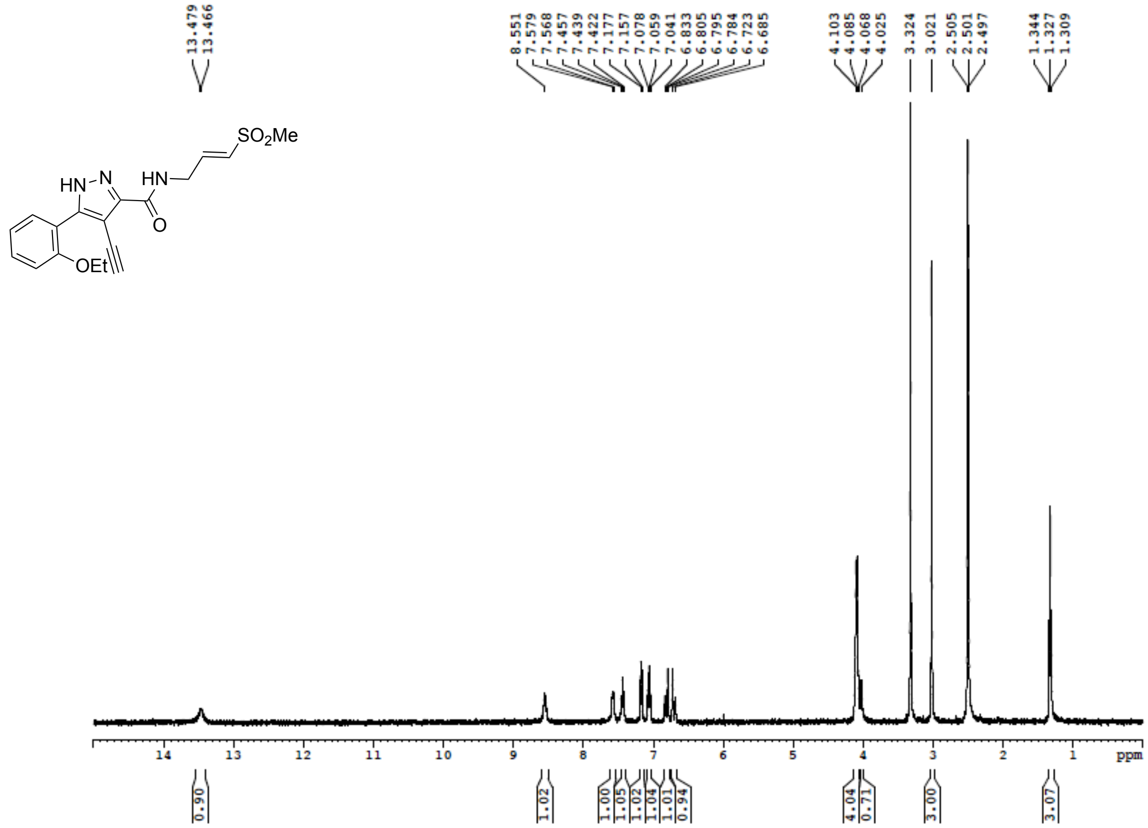

Figure S73: ^{13}C NMR (100 MHz, DMSO-d_6) for **8c**

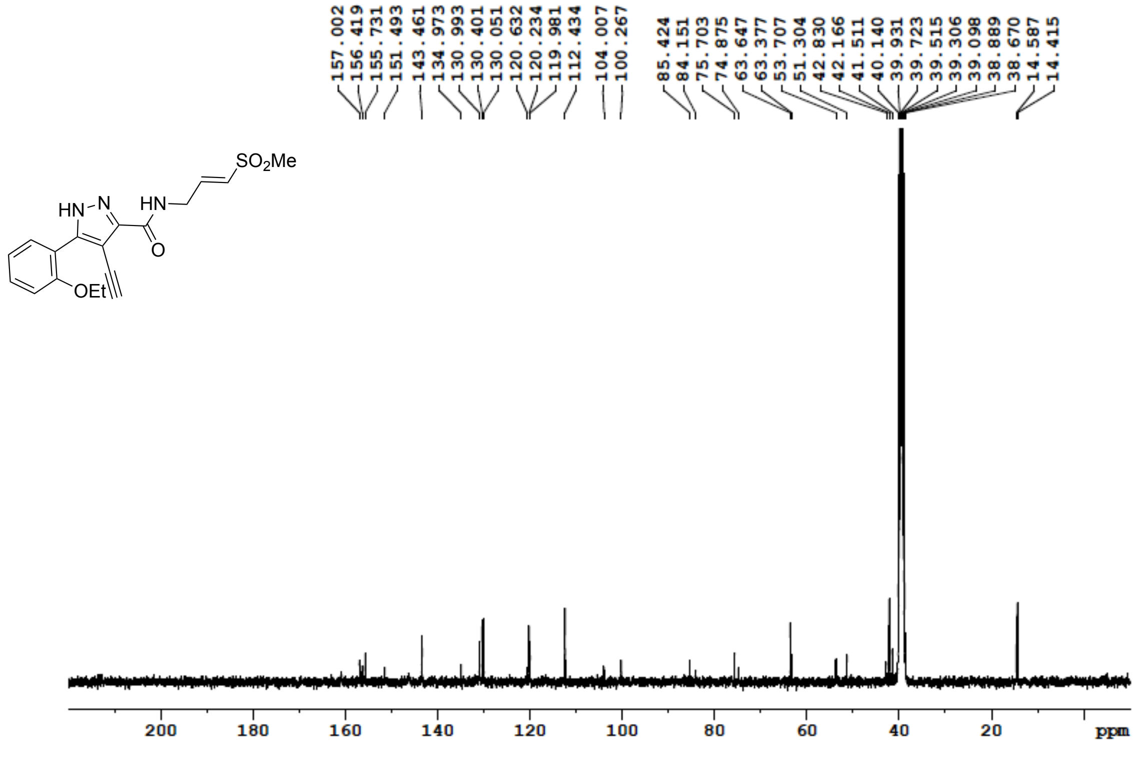

Figure S74: ^{1}H NMR (400 MHz, DMSO-d_6) for **8d**

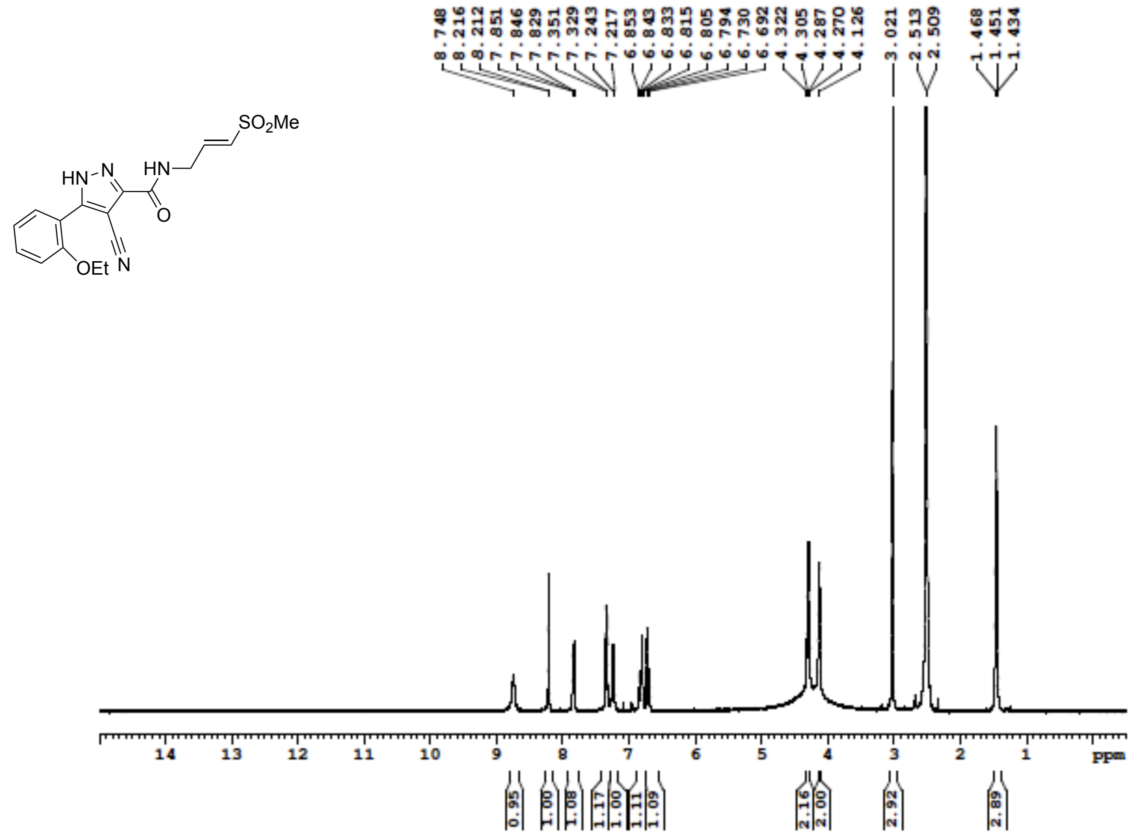

Figure S75: ^{13}C NMR (100 MHz, DMSO-d_6) for **8d**

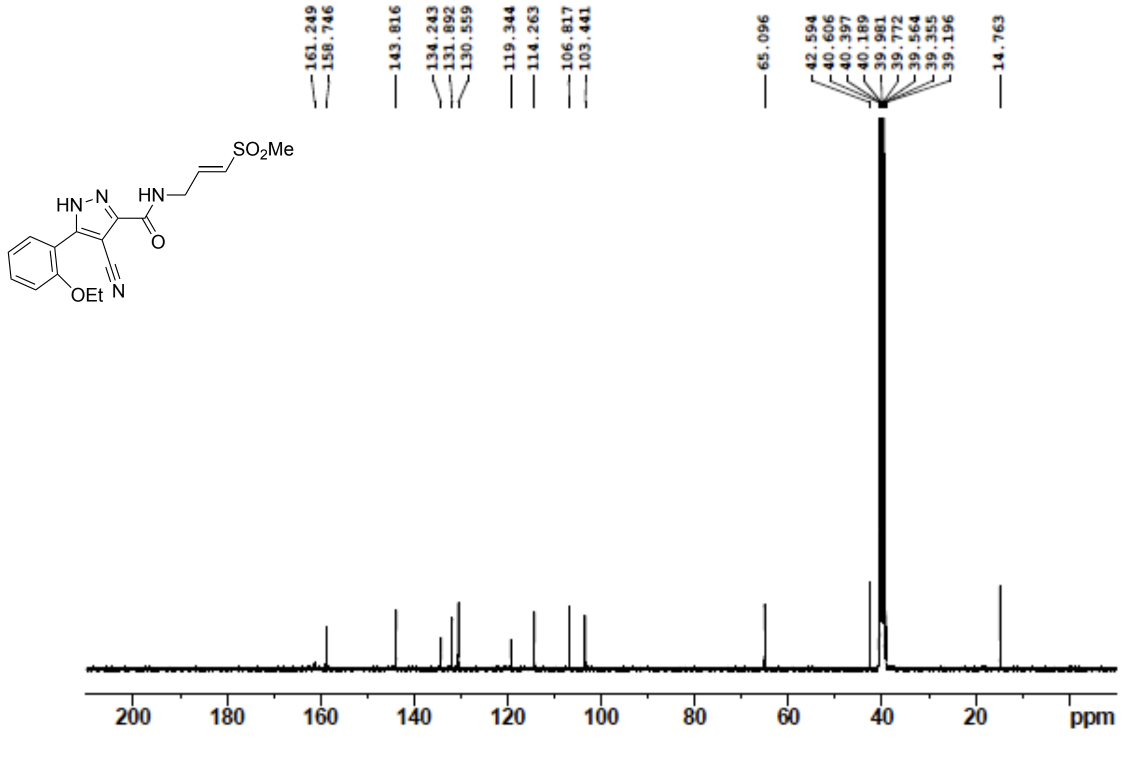

Figure S76: ^{1}H NMR (400 MHz, DMSO-d_6) for **9**

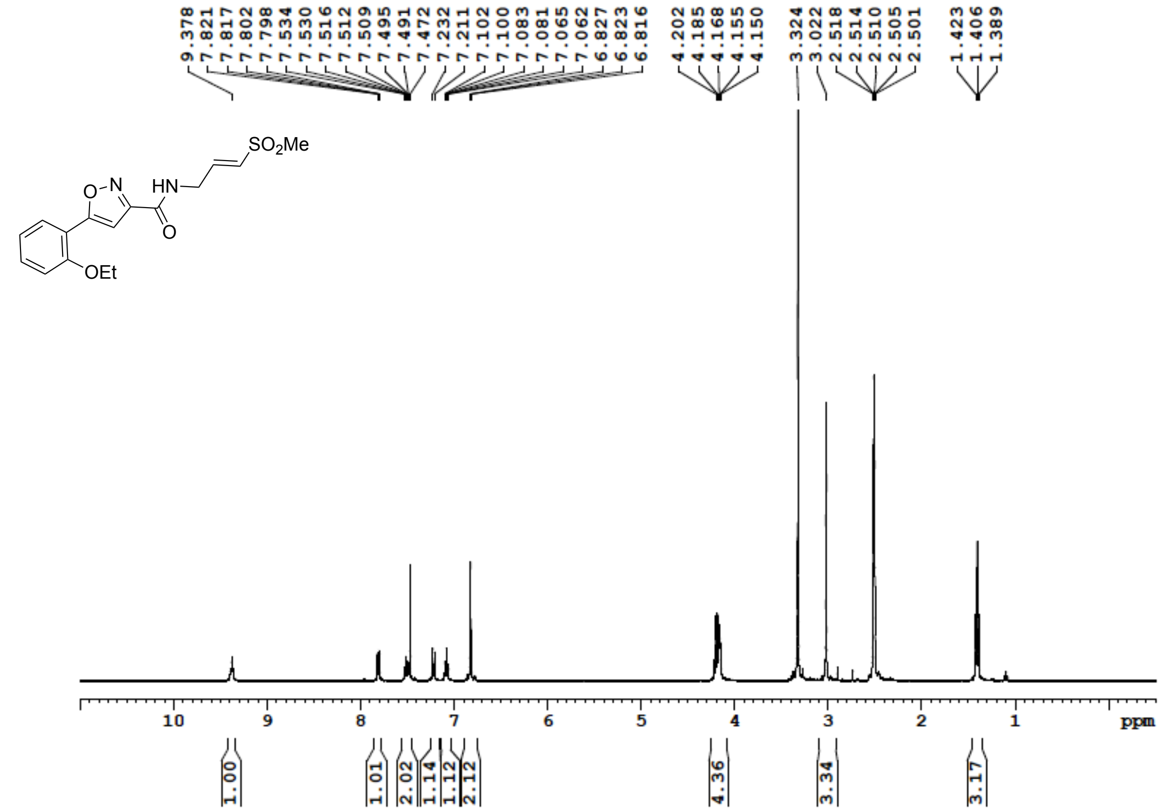

Figure S77: ^{13}C NMR (100 MHz, DMSO-d_6) for **9**

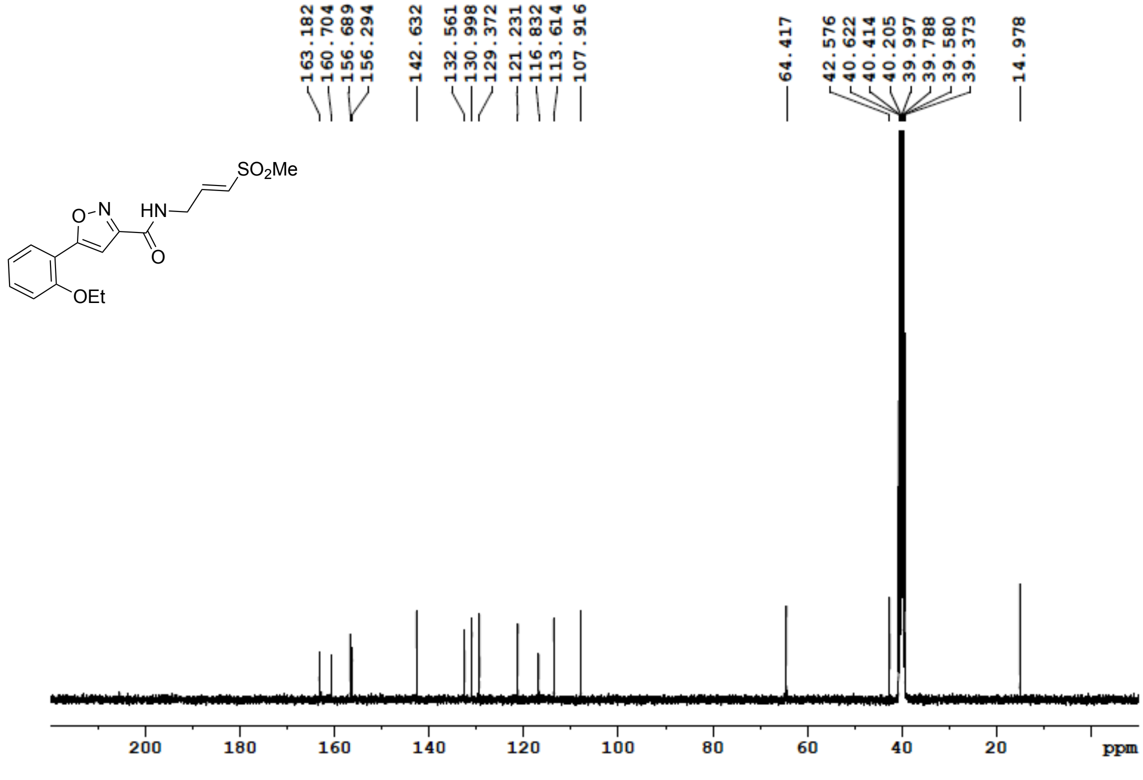

Figure S78: ^{1}H NMR (400 MHz, DMSO-d_6) for **10**

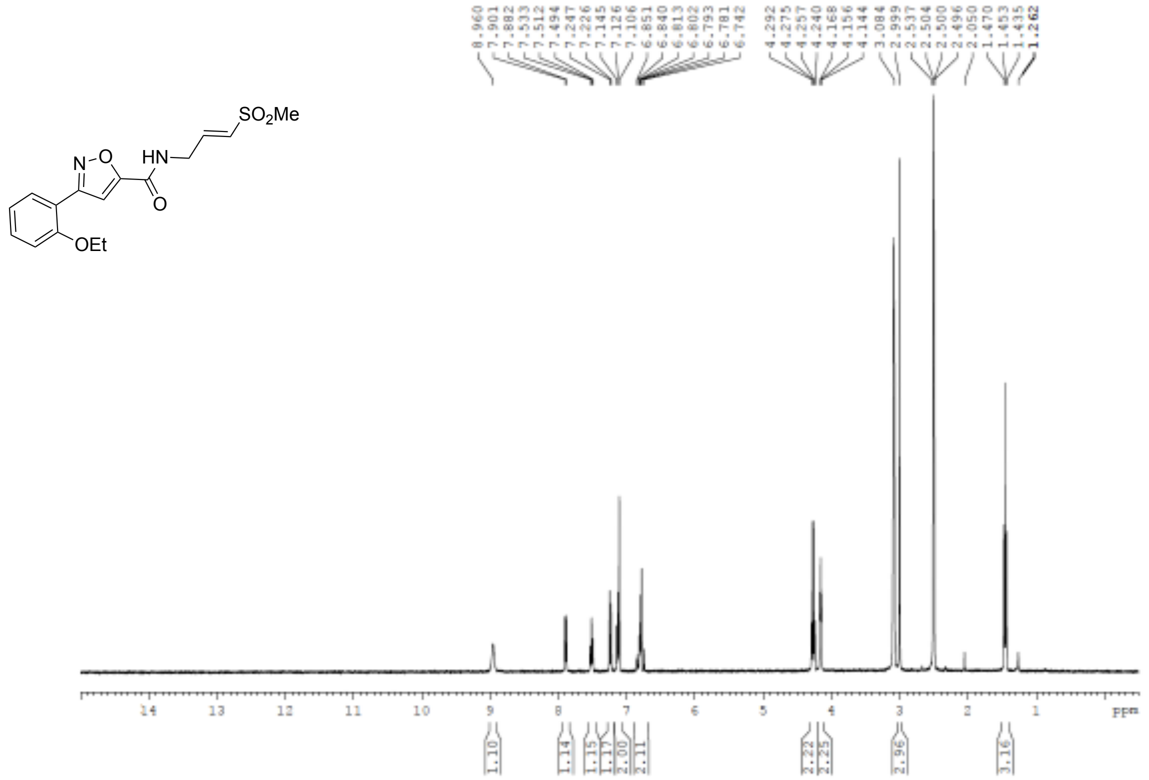

Figure S79: ^{13}C NMR (100 MHz, DMSO-d_6) for **10**

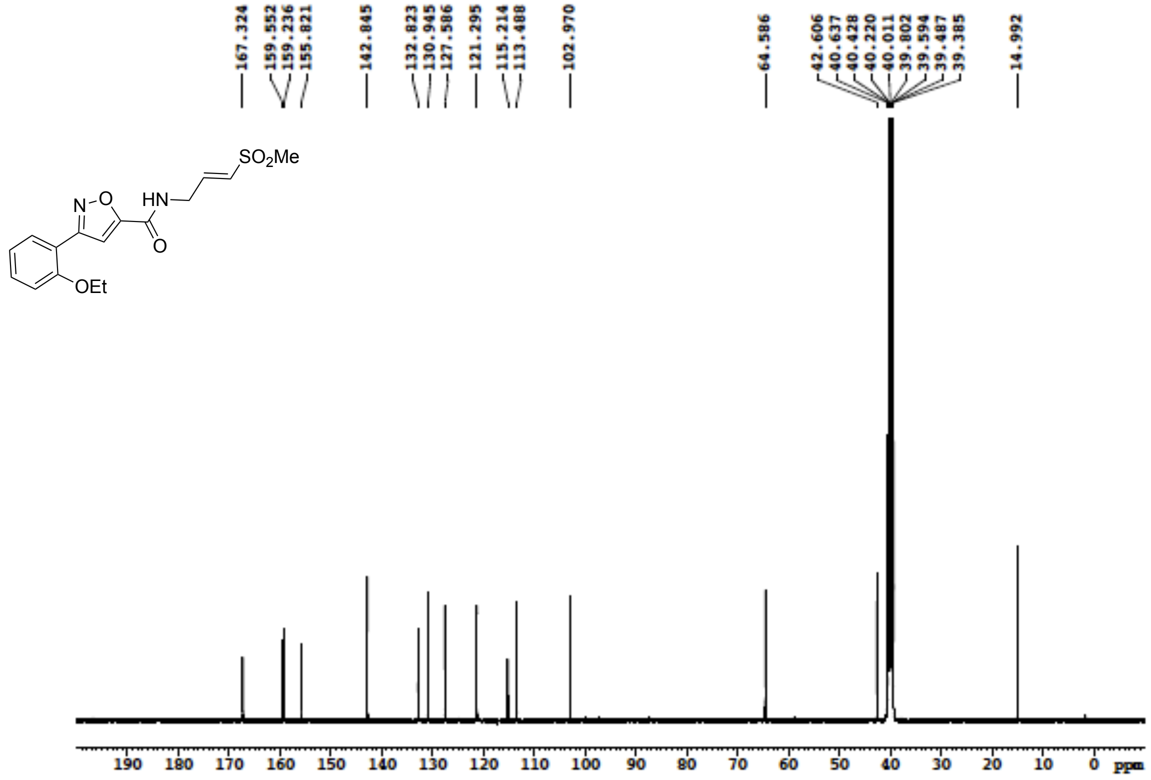

Figure S80: ^{1}H NMR (400 MHz, DMSO-d_6) for **11**

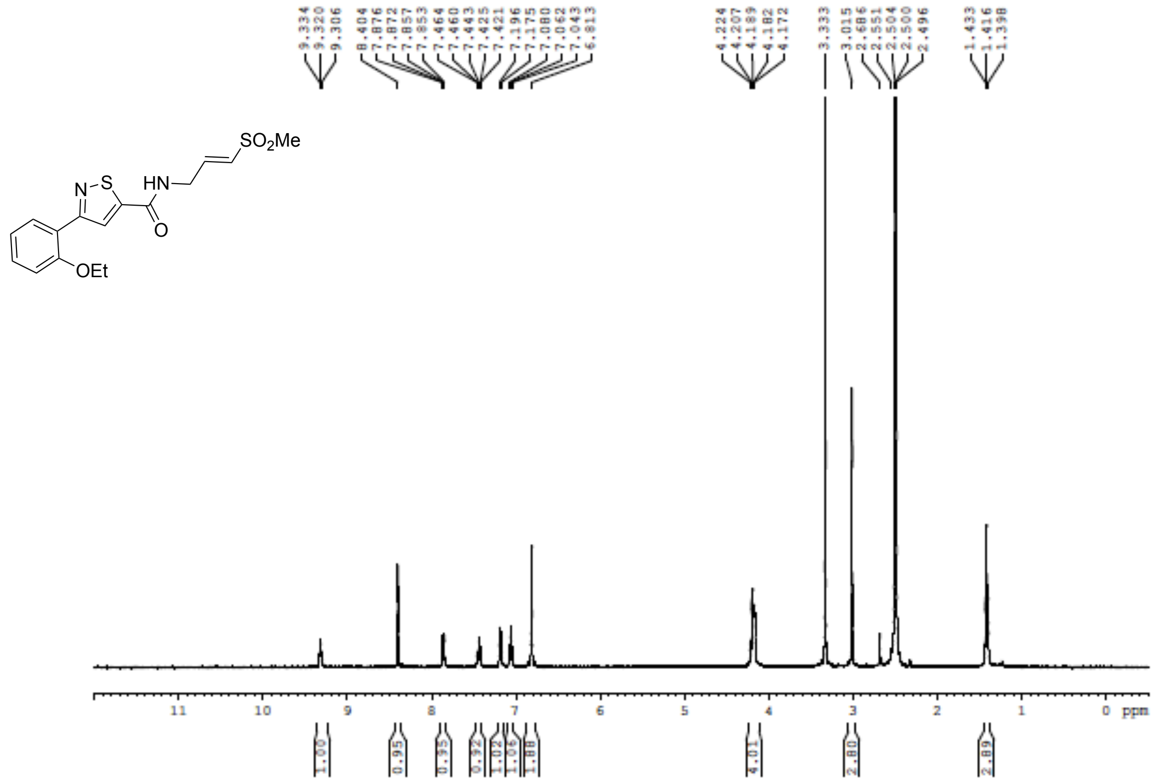

Figure S81: ^{13}C NMR (100 MHz, DMSO-d_6) for **11**

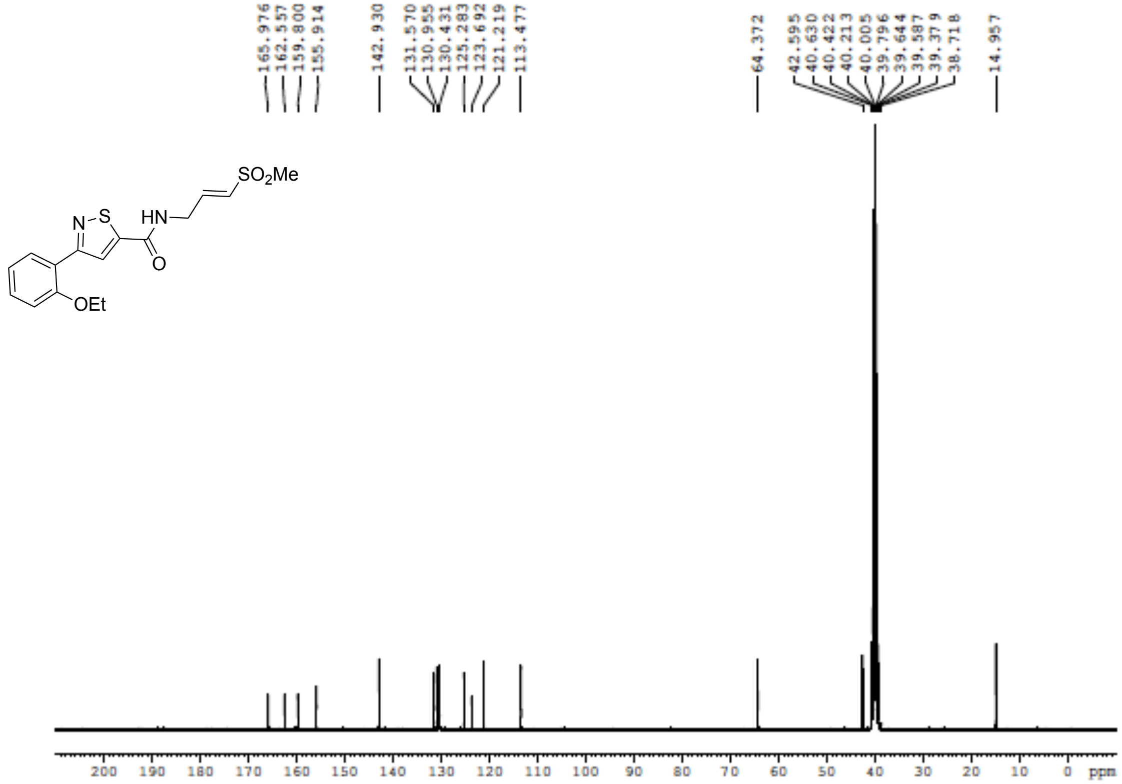

Figure S82: ^{1}H NMR (400 MHz, DMSO-d_6) for **12**

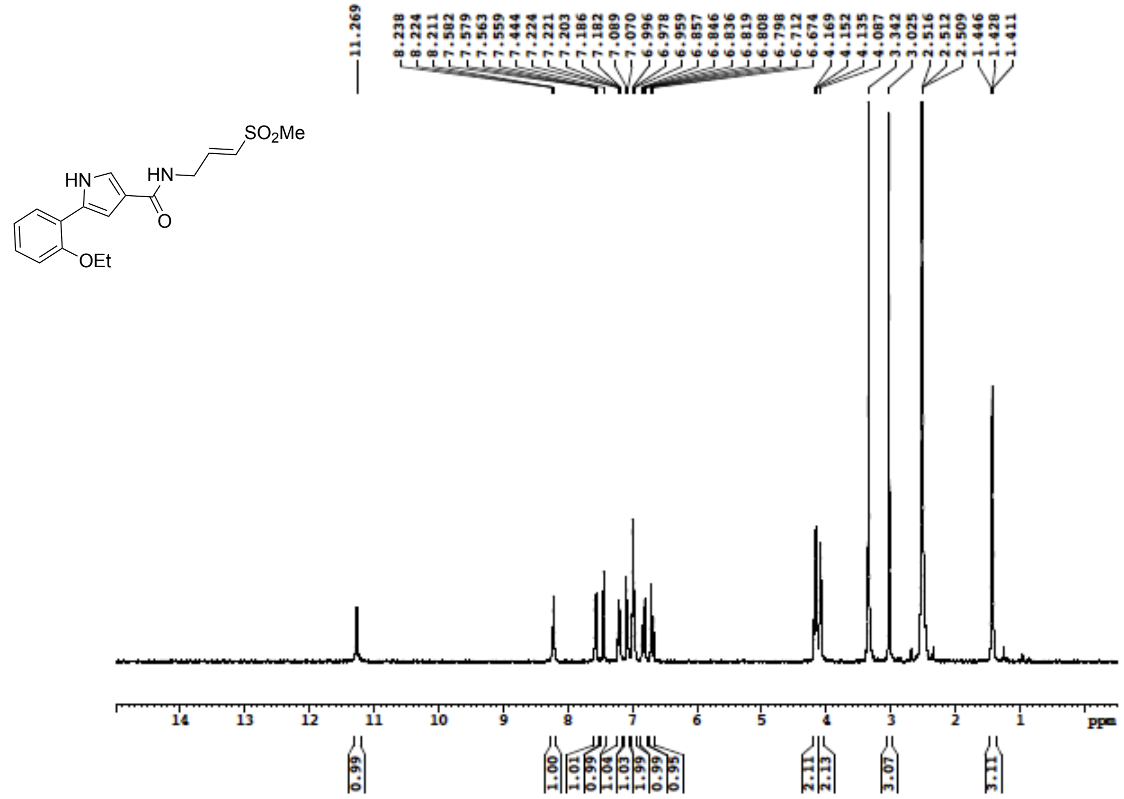

Figure S83: ^{13}C NMR (100 MHz, DMSO-d_6) for **12**

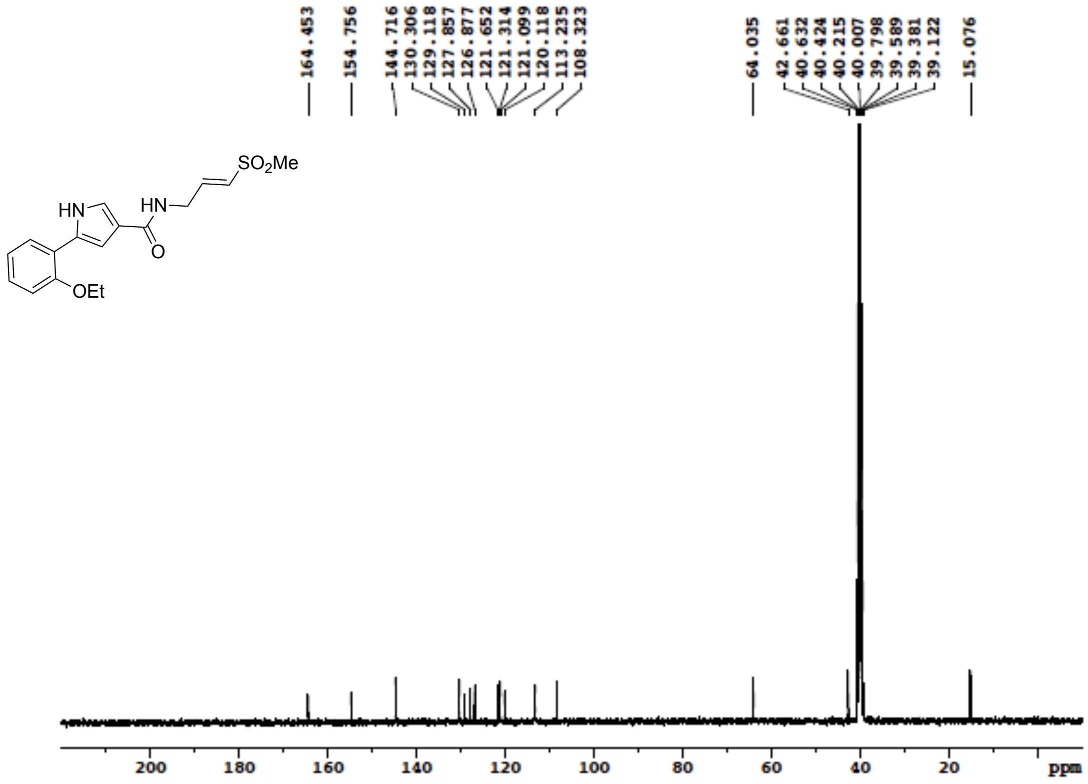

Figure S84: ^{1}H NMR (400 MHz, DMSO-d_6) for **13**

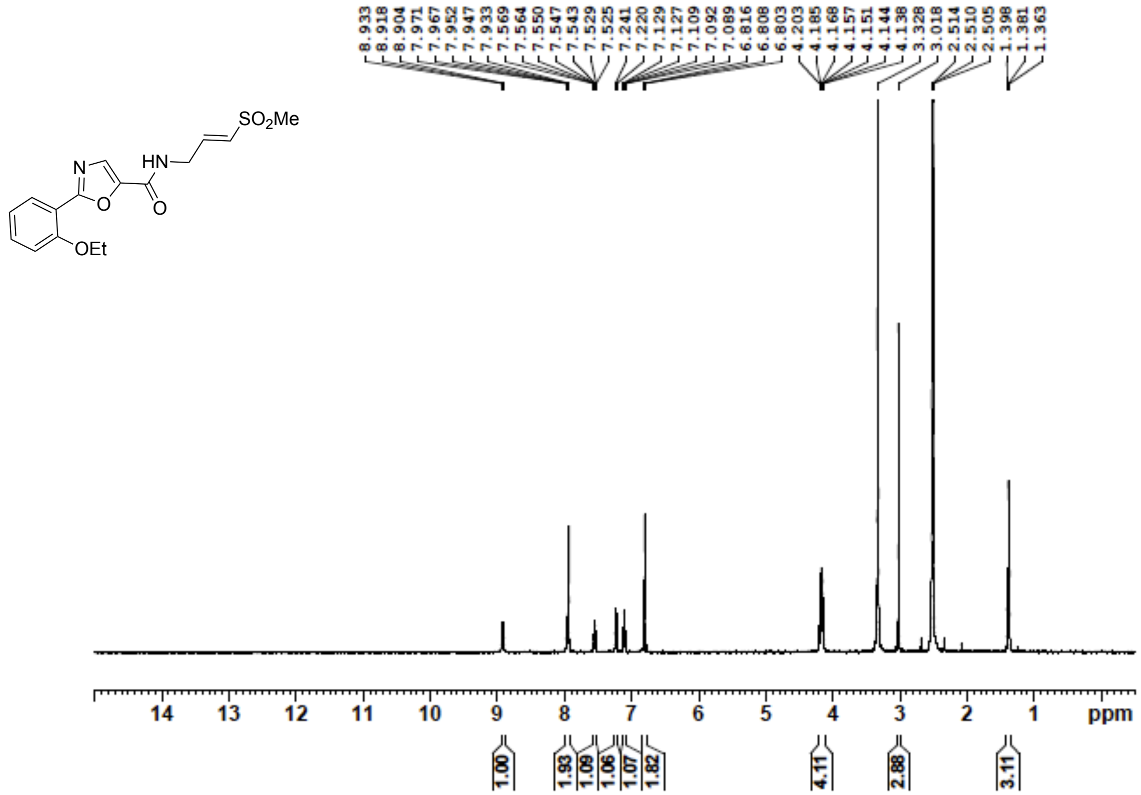

Figure S85: ^{13}C NMR (100 MHz, DMSO-d_6) for **13**

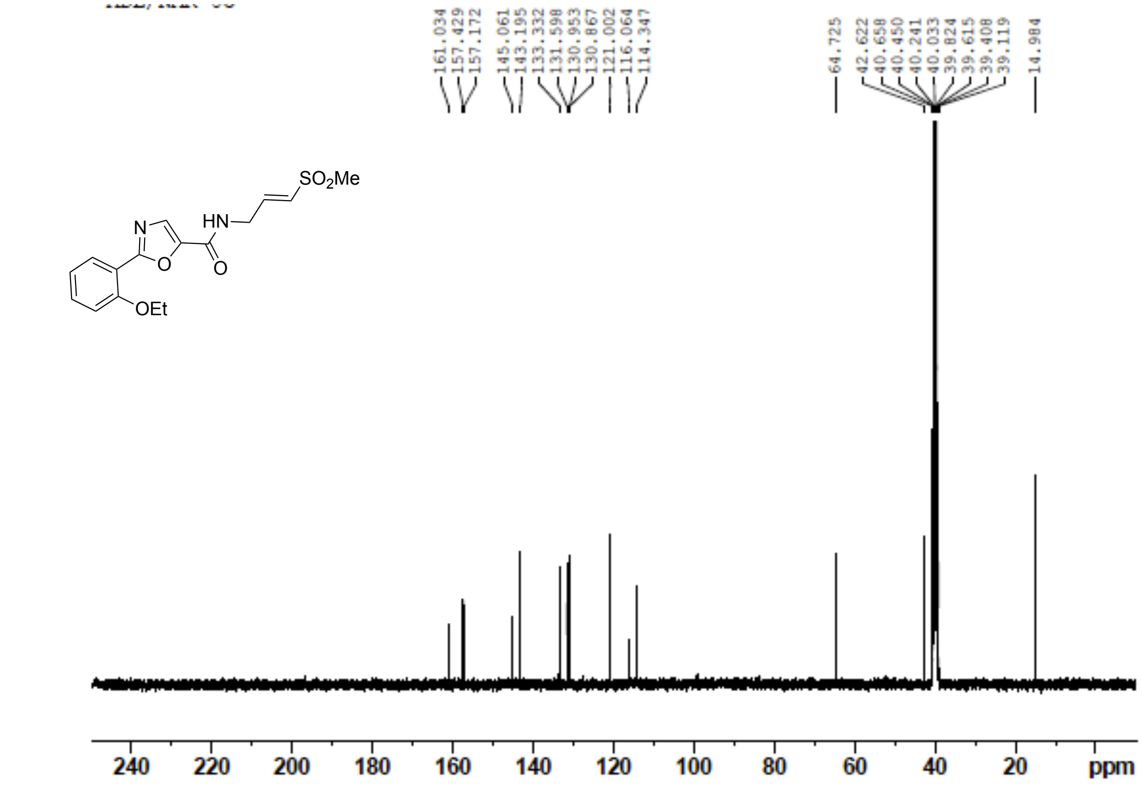

Figure S86: ^{1}H NMR (400 MHz, DMSO-d_6) for **14**

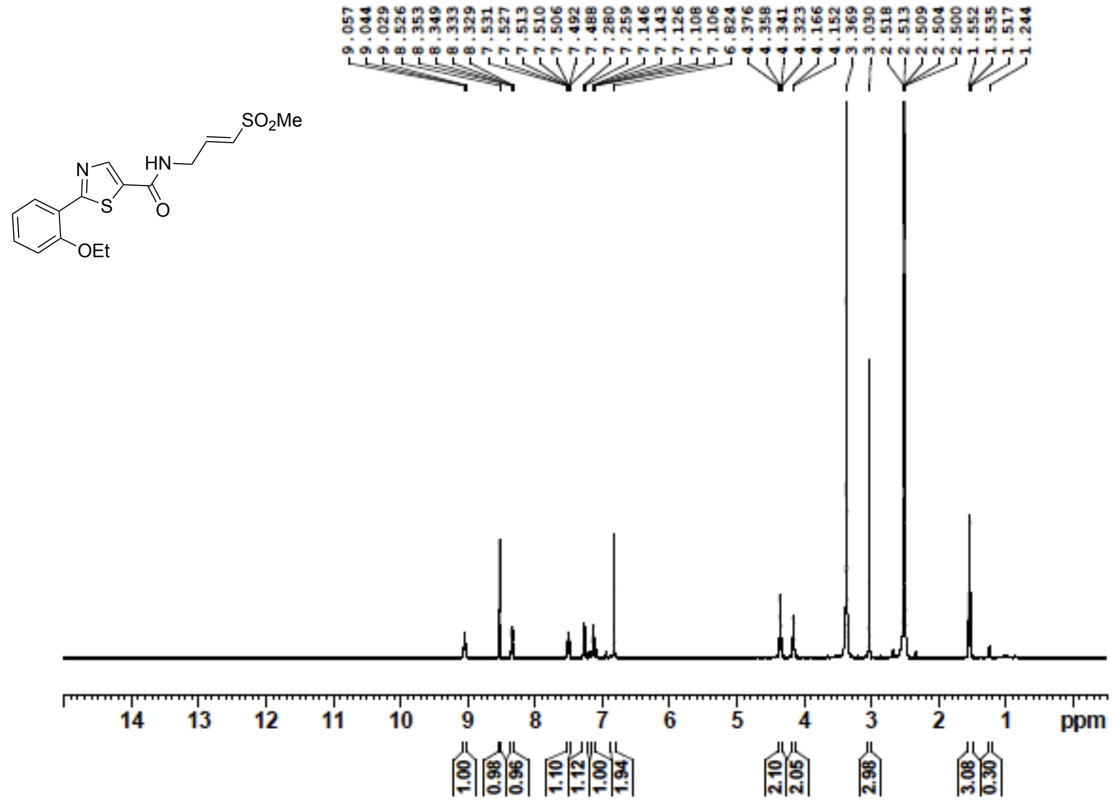

Figure S87: ^{13}C NMR (100 MHz, DMSO-d_6) for **14**

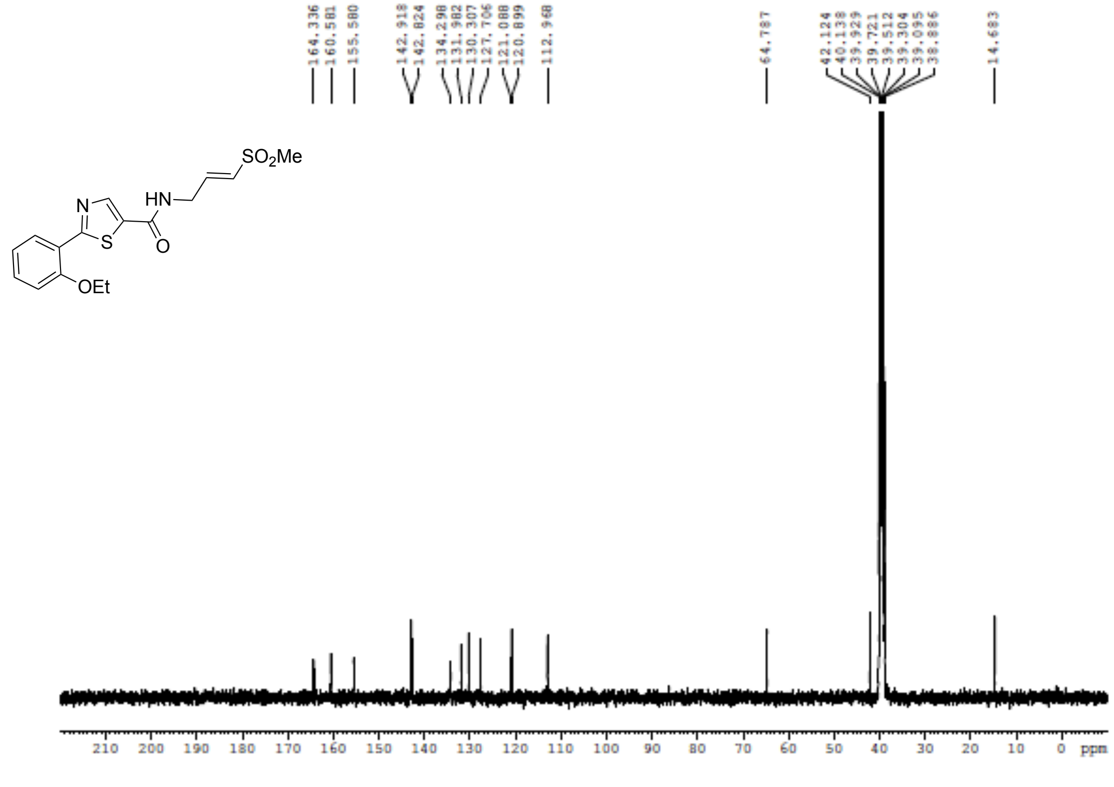

Figure S88: ^{1}H NMR (400 MHz, DMSO-d_6) for **15**

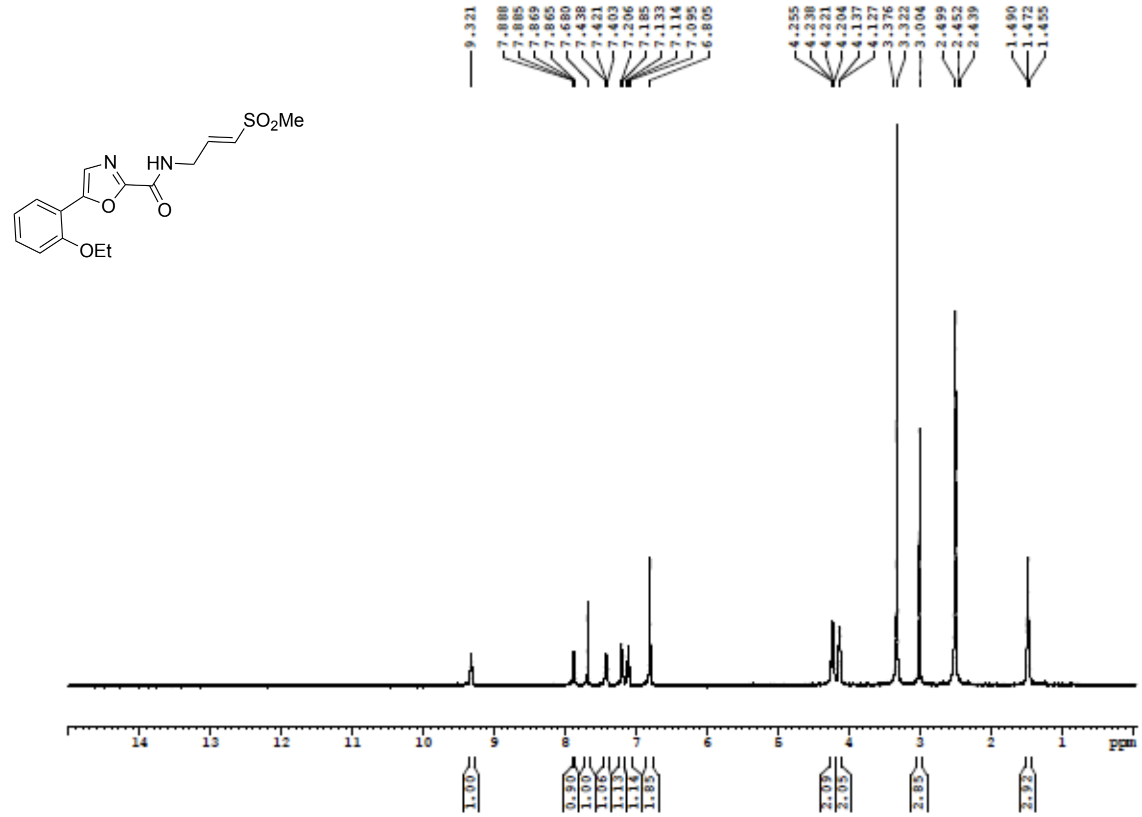

Figure S89: ^{13}C NMR (100 MHz, DMSO-d_6) for **15**

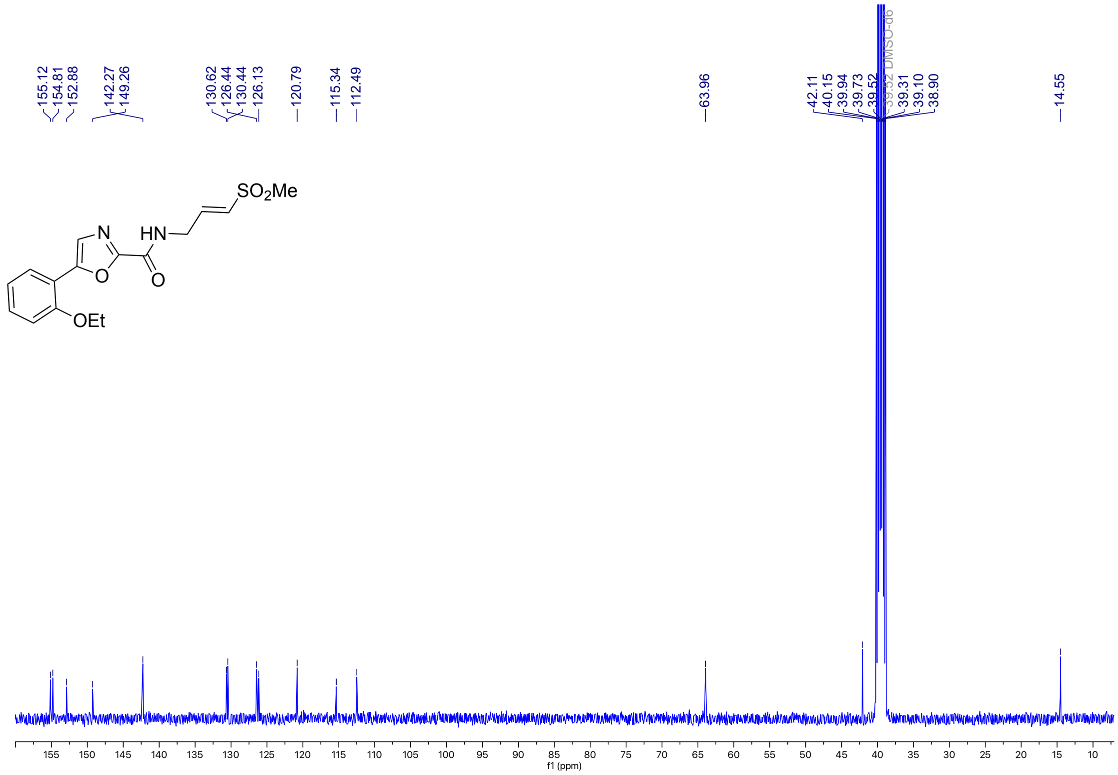

Figure S90: ^{1}H NMR (400 MHz, DMSO-d_6) for **16**

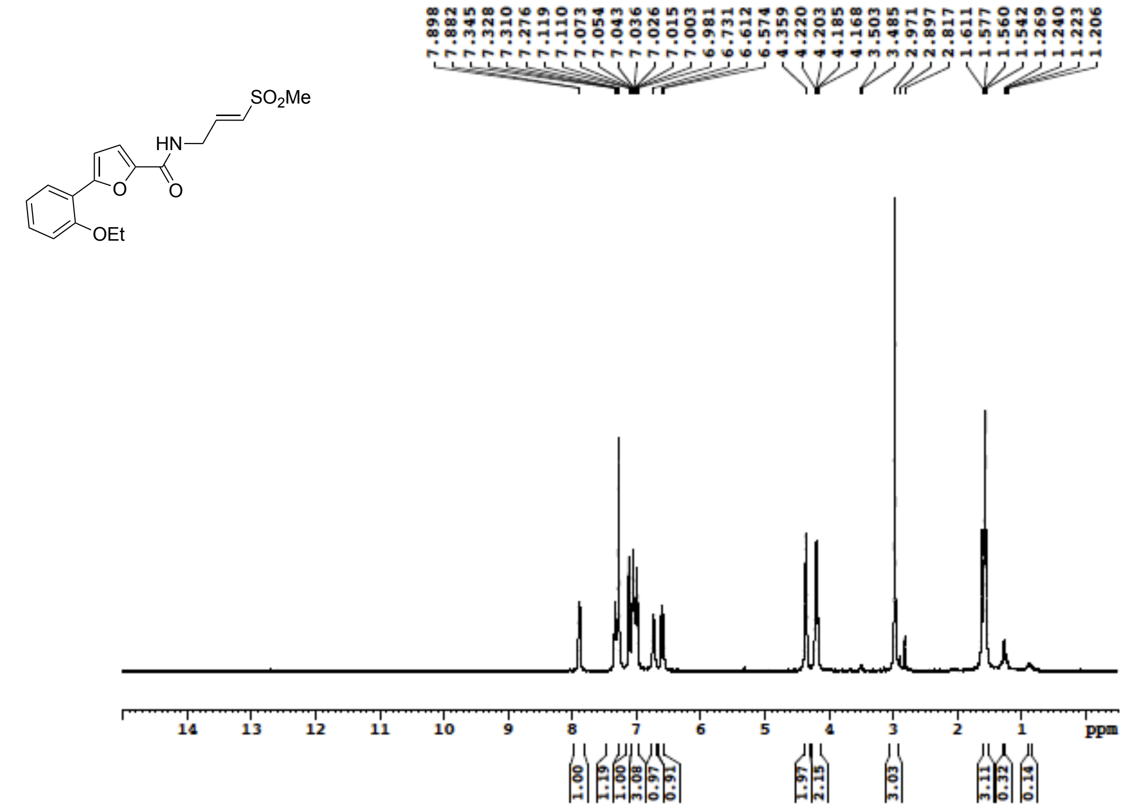

Figure S91: ^{13}C NMR (100 MHz, DMSO-d_6) for **16**

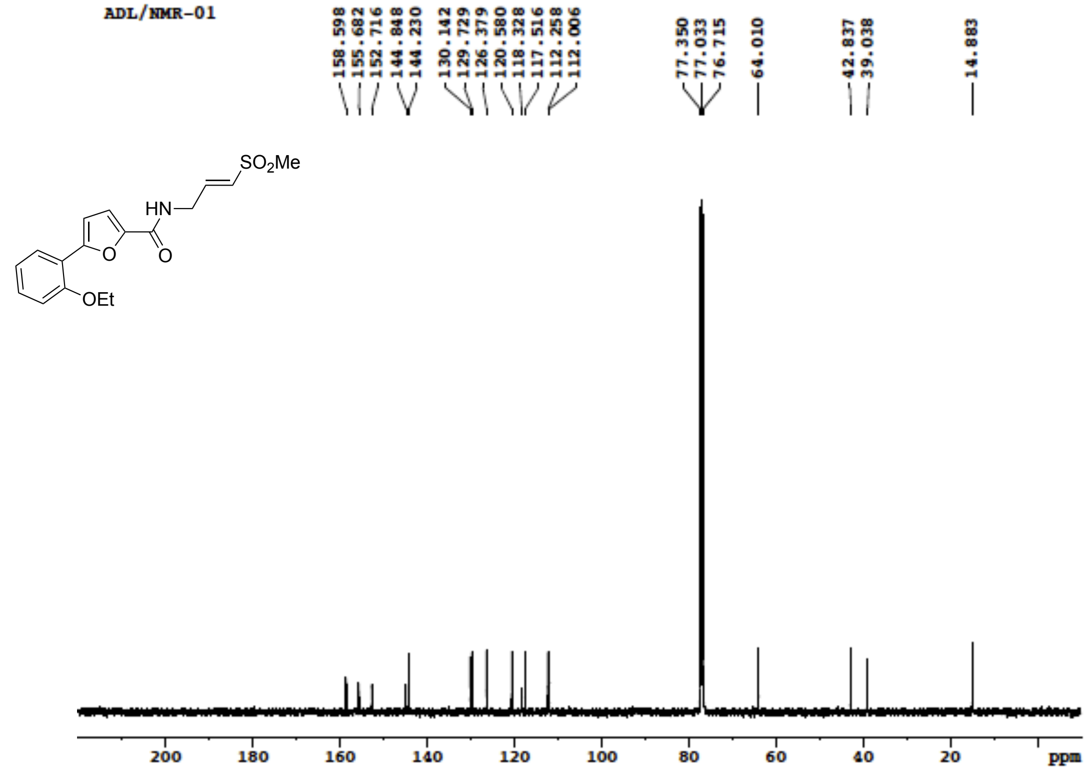

Figure S92: ^{1}H NMR (400 MHz, DMSO-d_6) for **17**

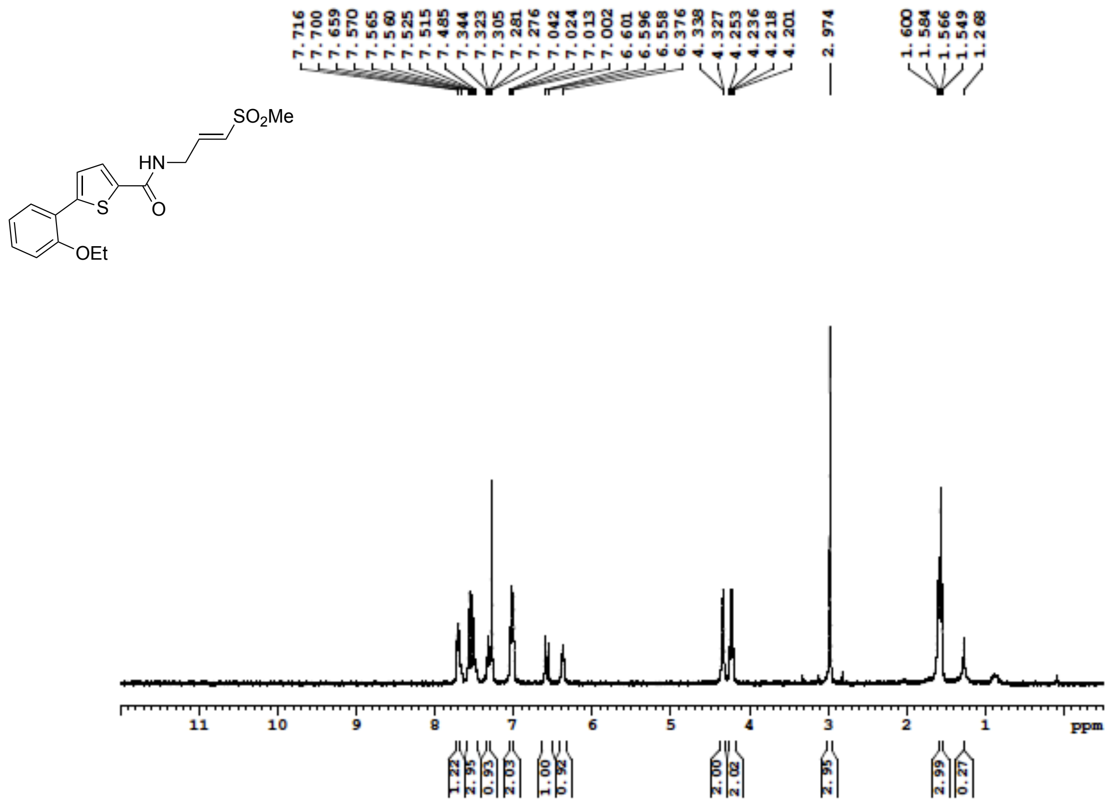

Figure S93: ^{13}C NMR (100 MHz, DMSO-d_6) for **17**

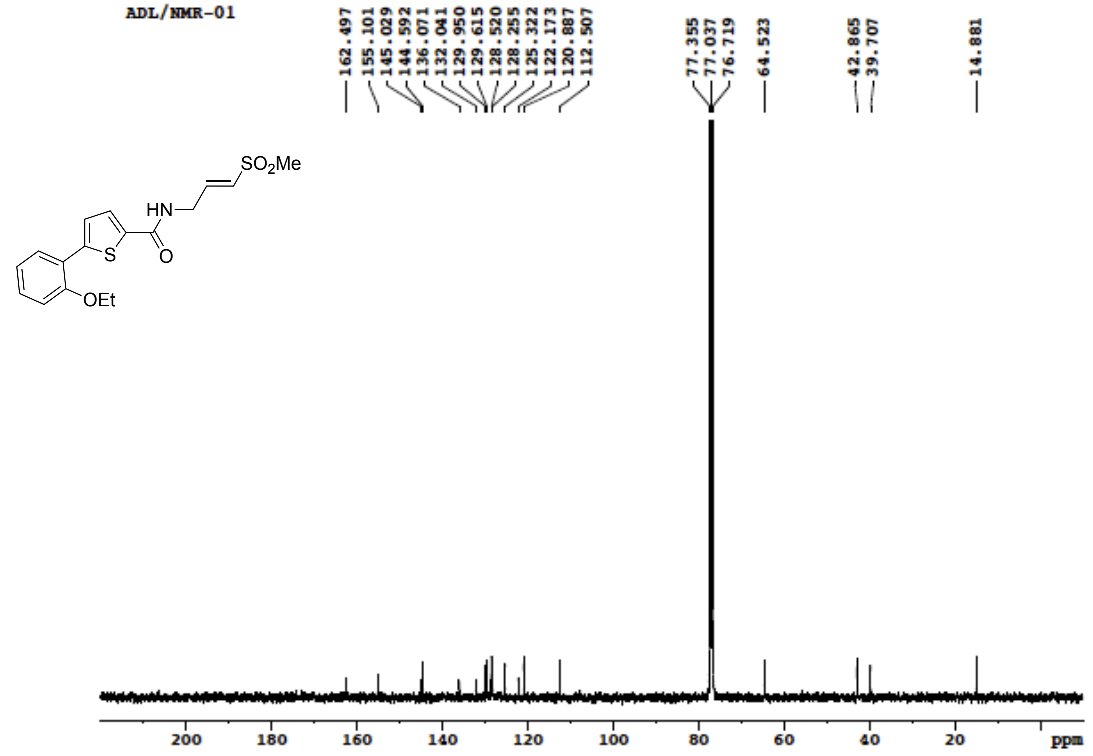

Figure S94: ^{1}H NMR (400 MHz, DMSO-d_6) for **18**

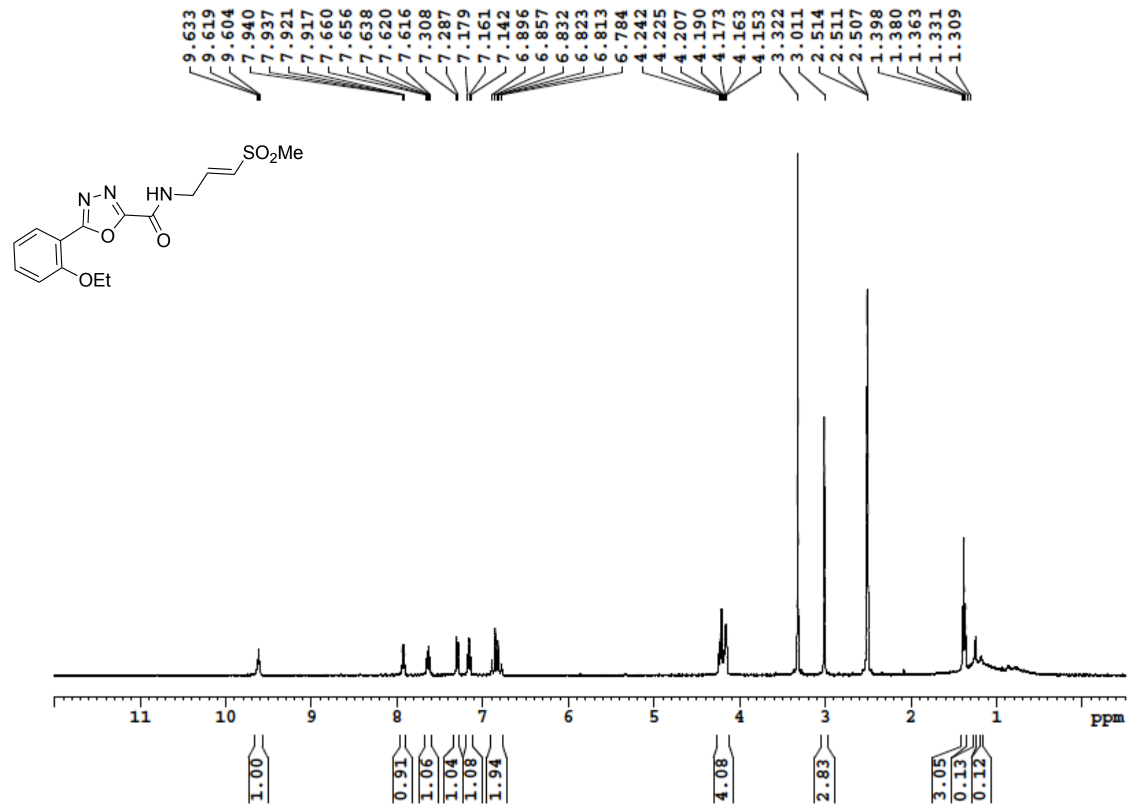

Figure S95: ^{13}C NMR (101 MHz, DMSO-d_6) for **18**

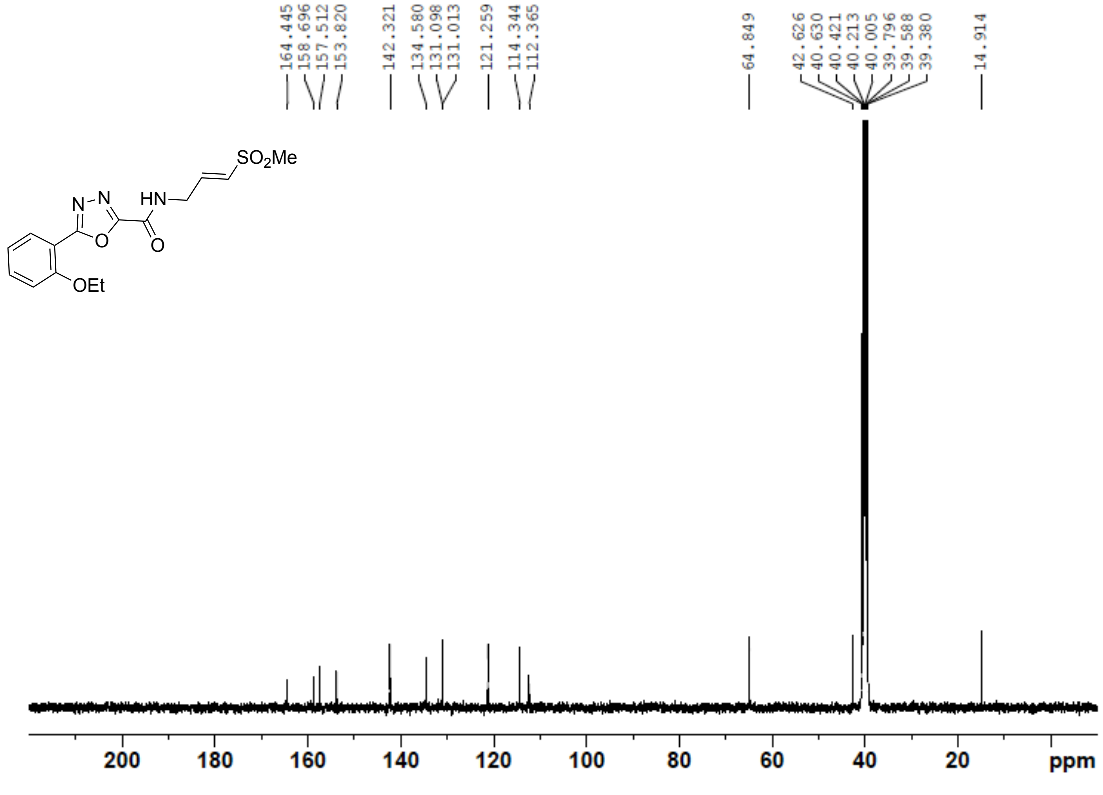

Figure S96: ^{1}H NMR (400 MHz, DMSO-d_6) for **19**

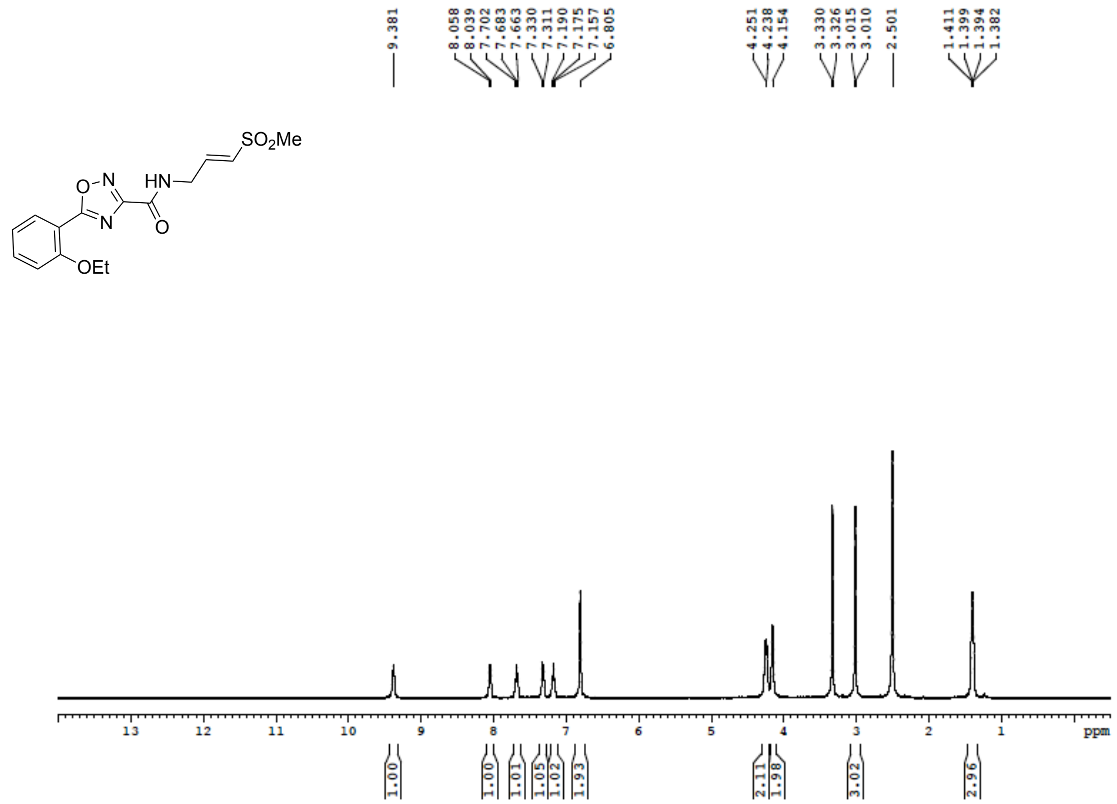

Figure S97: ^{13}C NMR (100 MHz, DMSO-d_6) for **19**

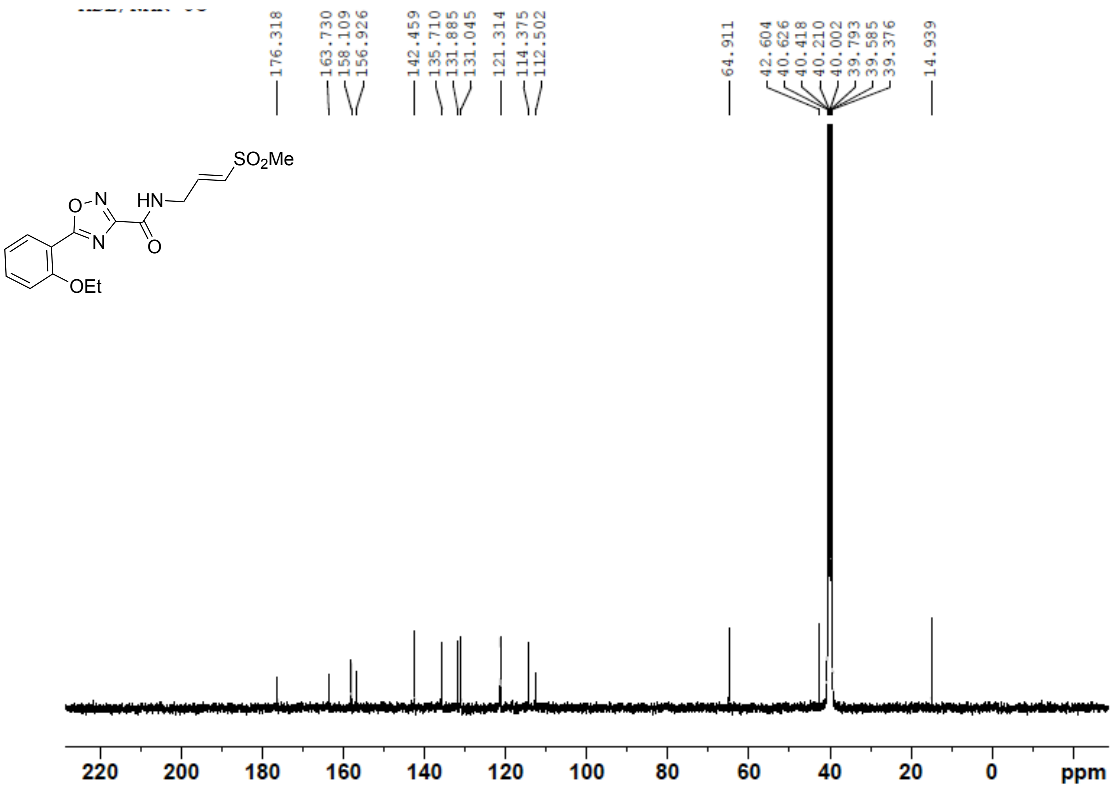

Figure S98: ^{1}H NMR (400 MHz, DMSO-d_6) for **20**

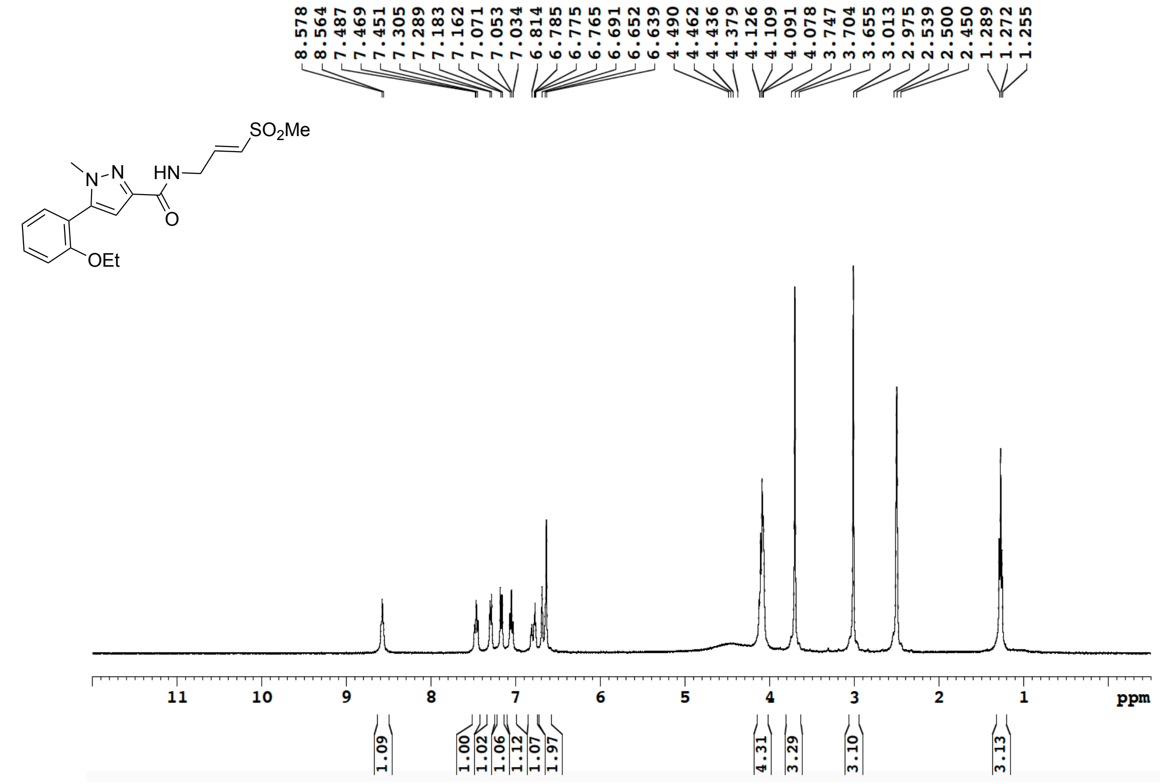

Figure S99: ^{13}C NMR (100 MHz, DMSO-d_6) for **20**

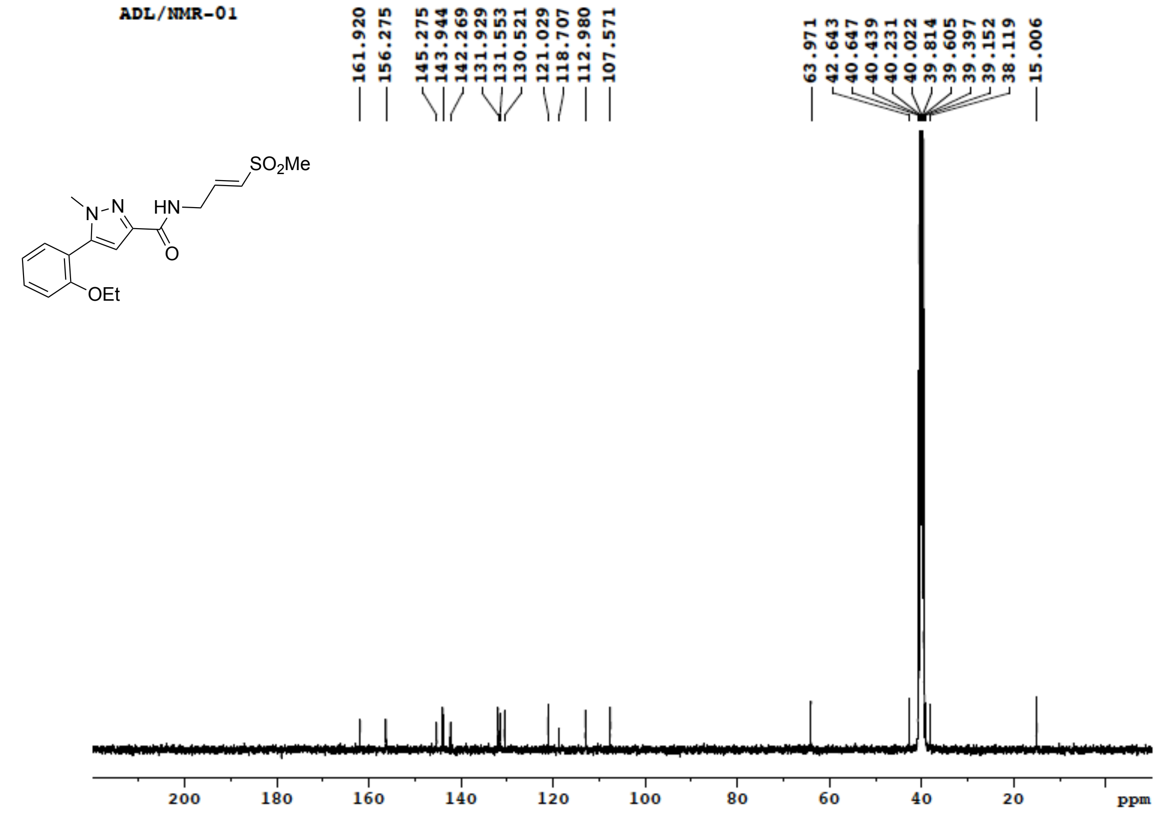

Figure S100: ^{1}H NMR (400 MHz, DMSO-d_6) for **21**

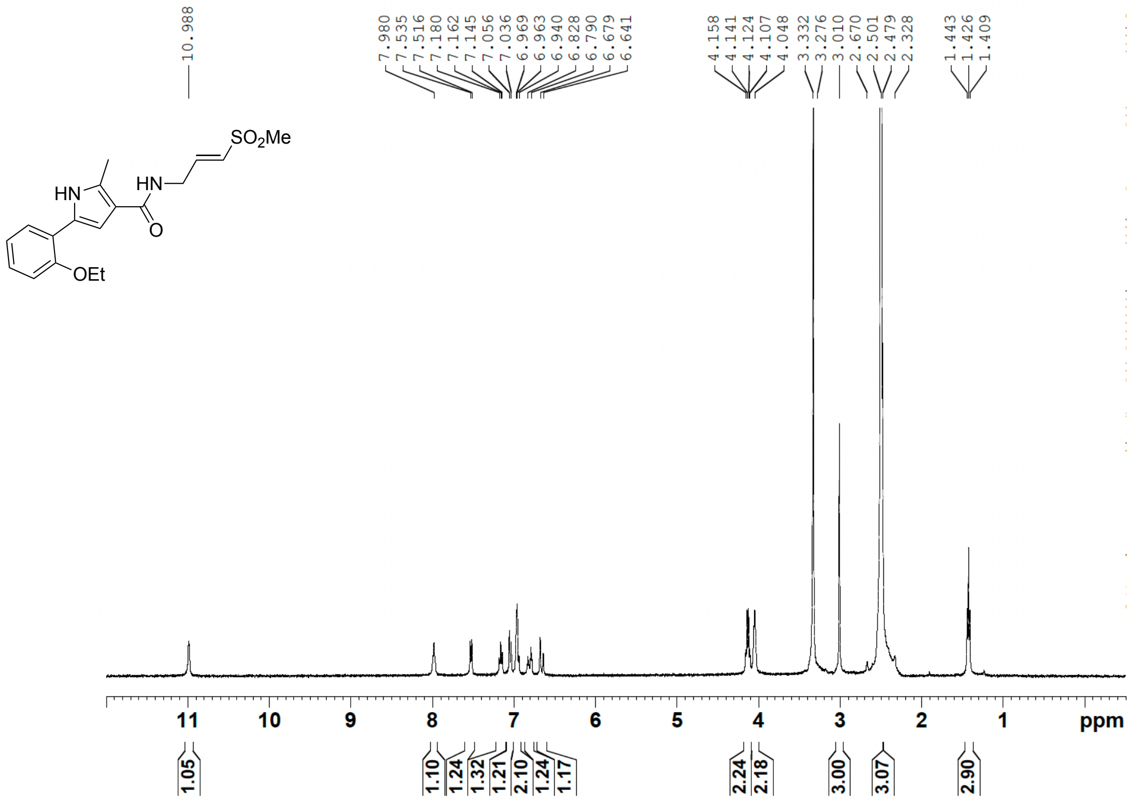

Figure S101: ^{13}C NMR (100 MHz, DMSO-d_6) for **21**

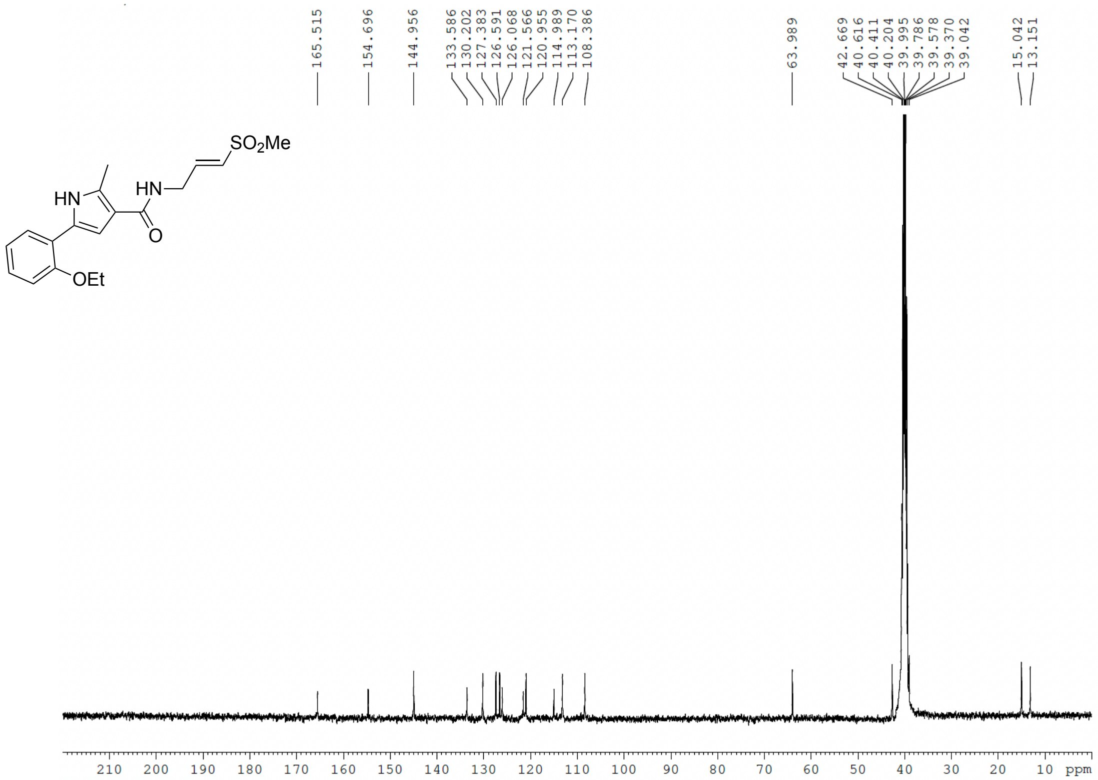

Figure S102: ^{1}H NMR (400 MHz, DMSO-d_6) for **22**

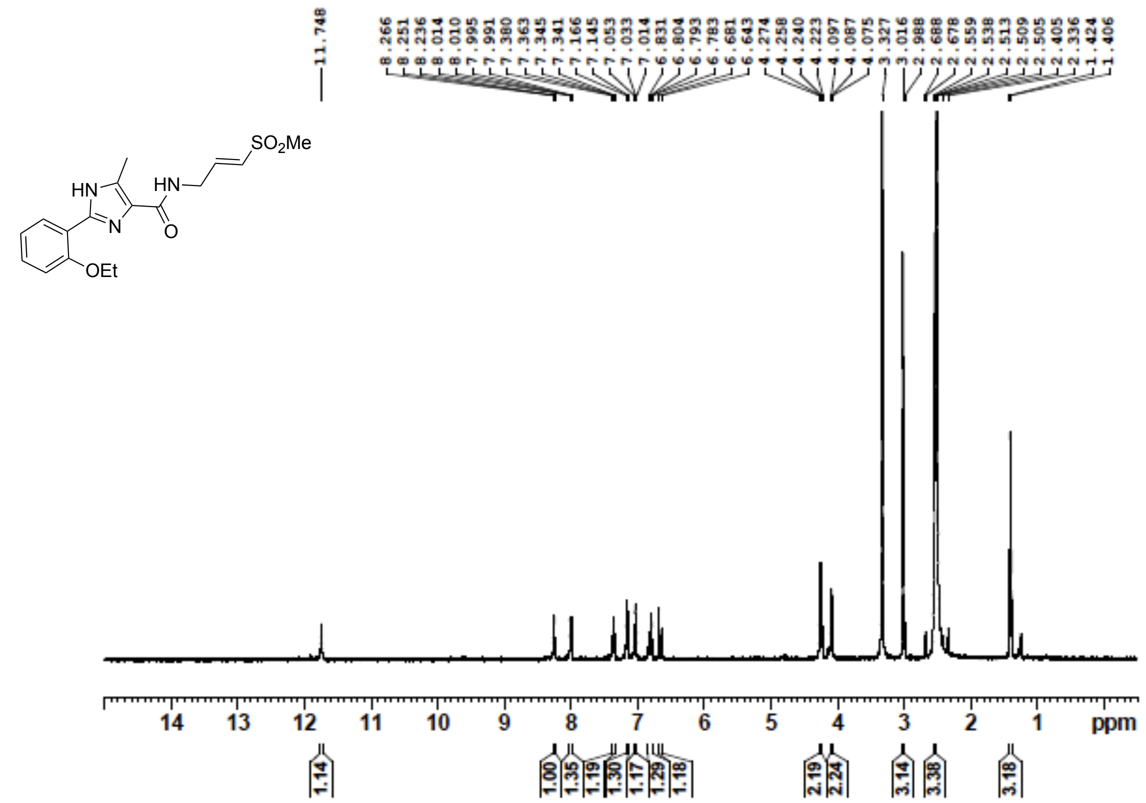

Figure S103: ^{13}C NMR (100 MHz, DMSO-d_6) for **22**

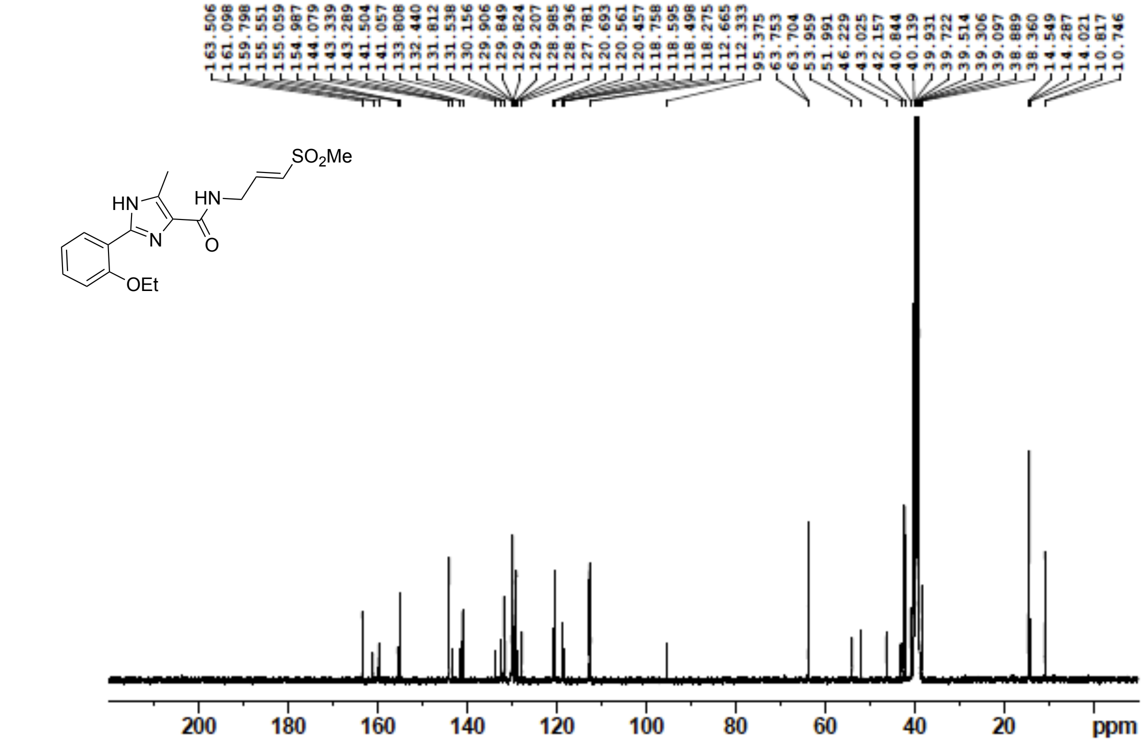

Figure S104: ^{1}H NMR (400 MHz, DMSO-d_6) for **23a**

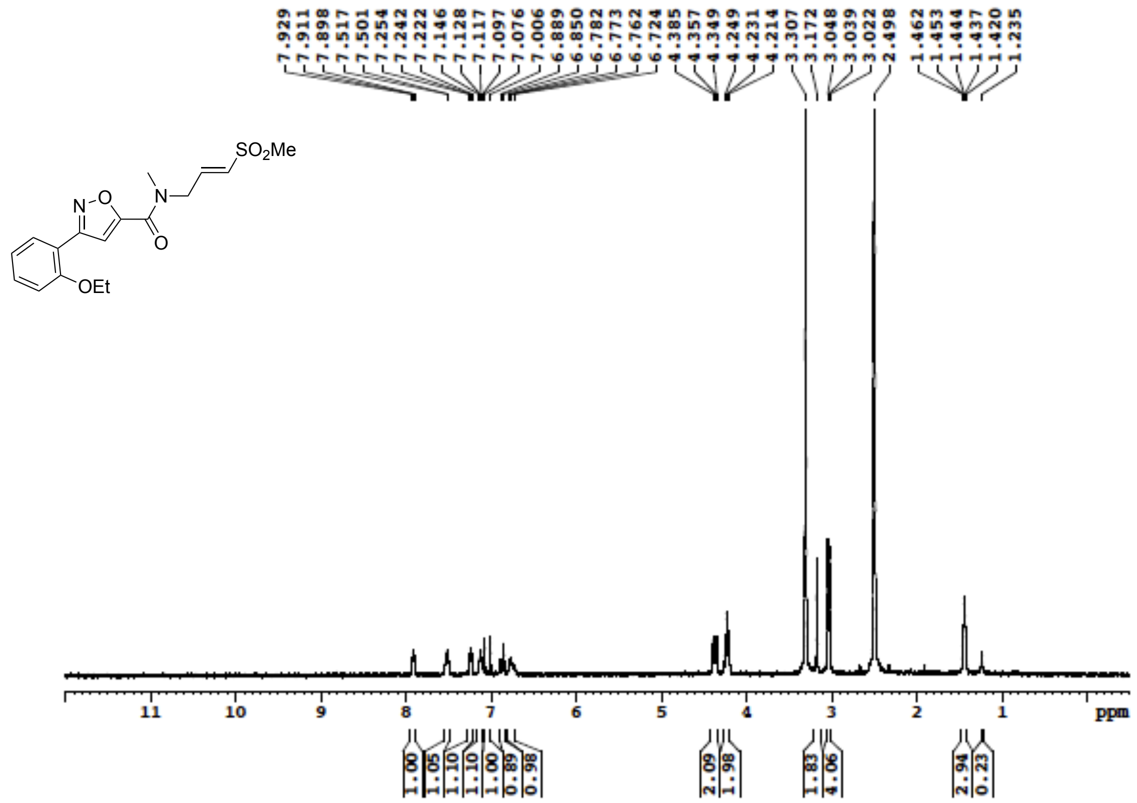

Figure S105: ^{13}C NMR (100 MHz, DMSO-d_6) for **23a**

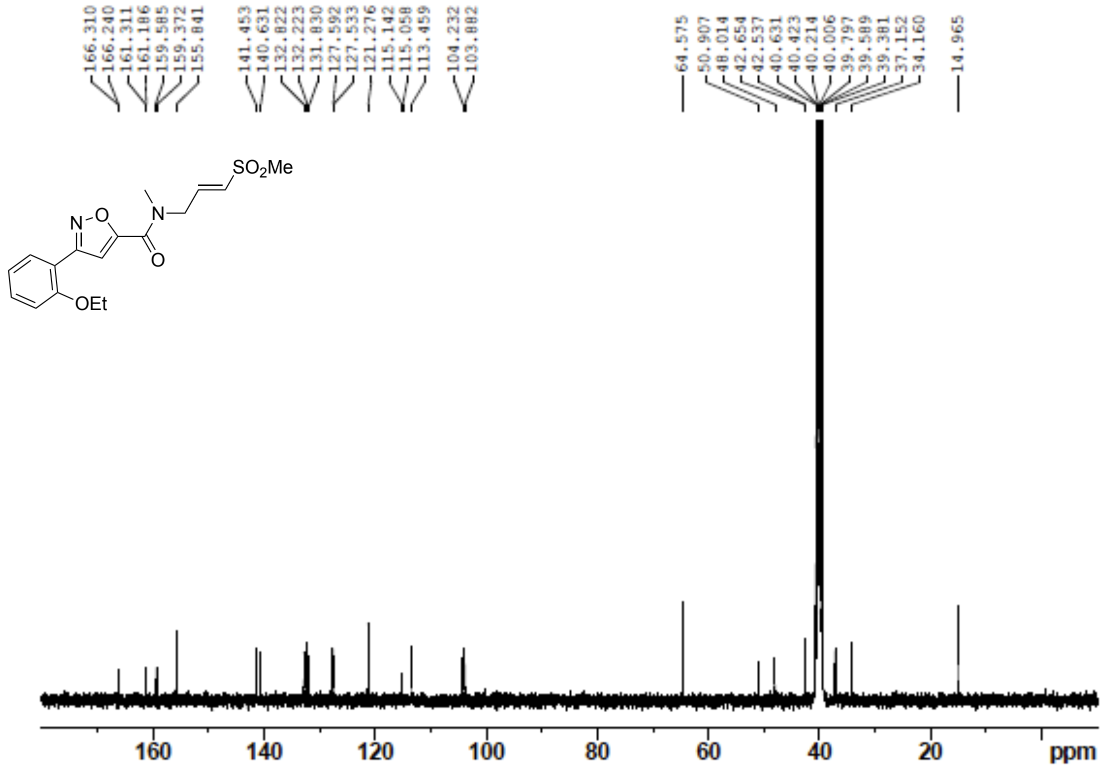

Figure S106: ^{1}H NMR (400 MHz, DMSO-d_6) for **23b**

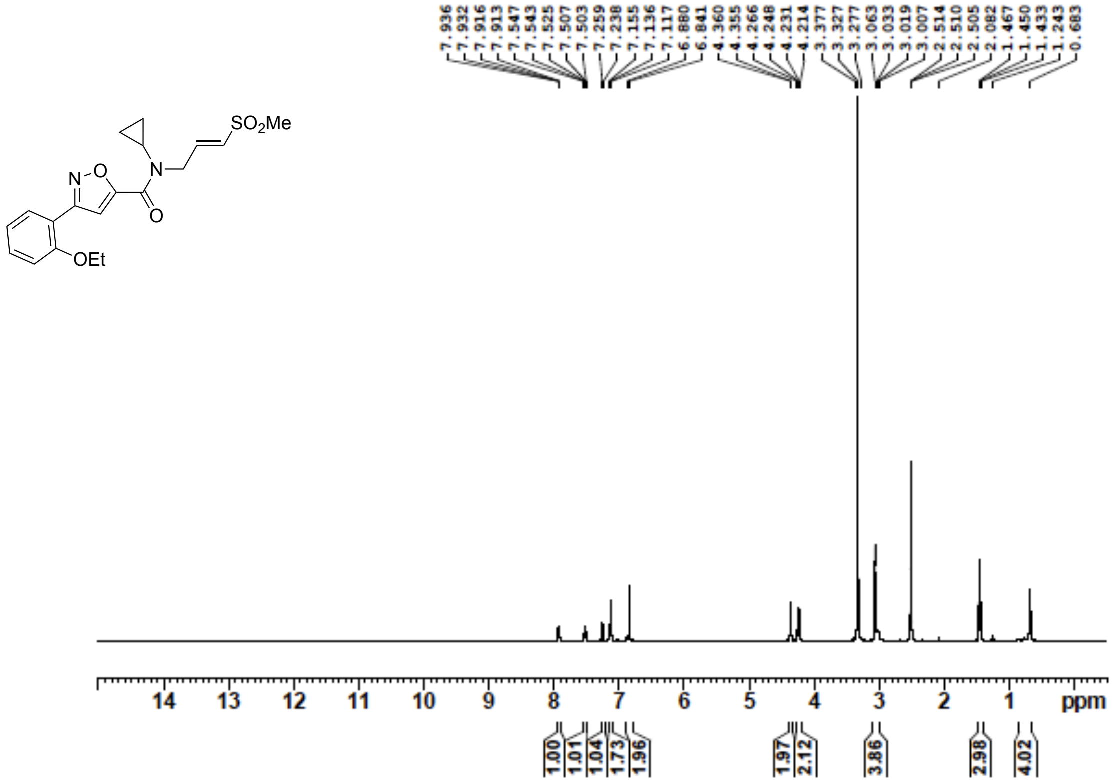

Figure S107: ^{13}C NMR (100 MHz, DMSO-d_6) for **23b**

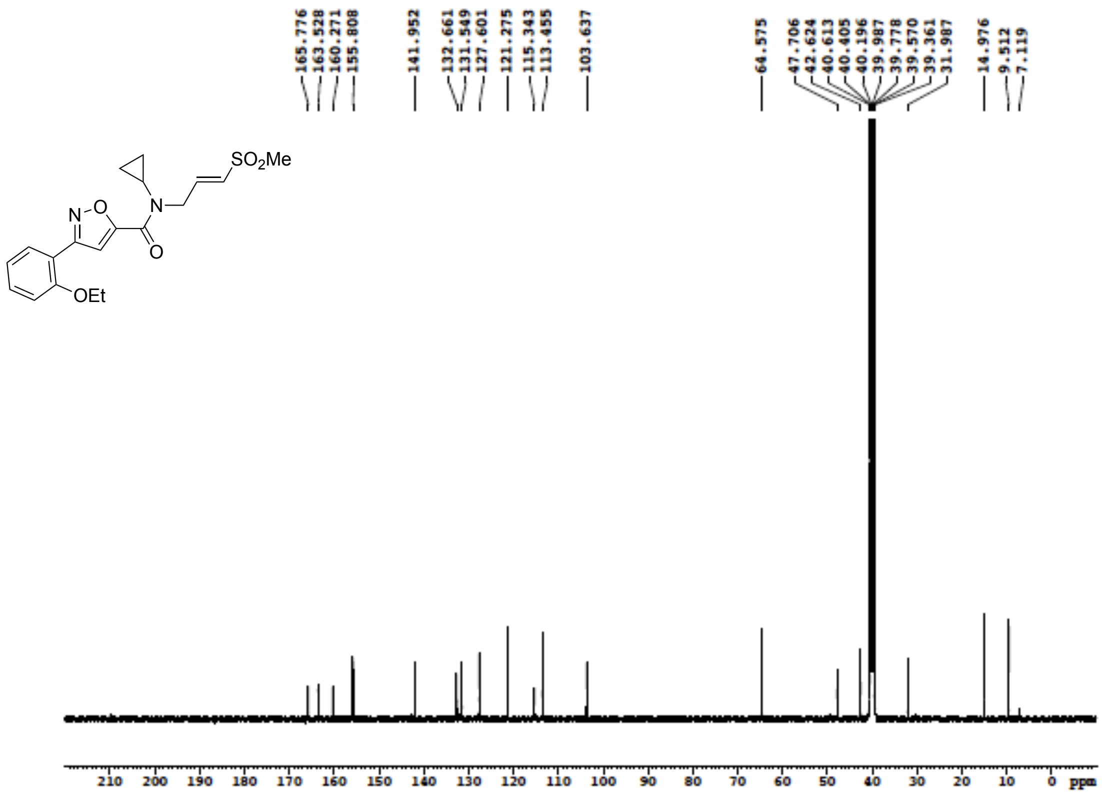

Figure S108: ^{1}H NMR (400 MHz, DMSO-d_6) for **23c**

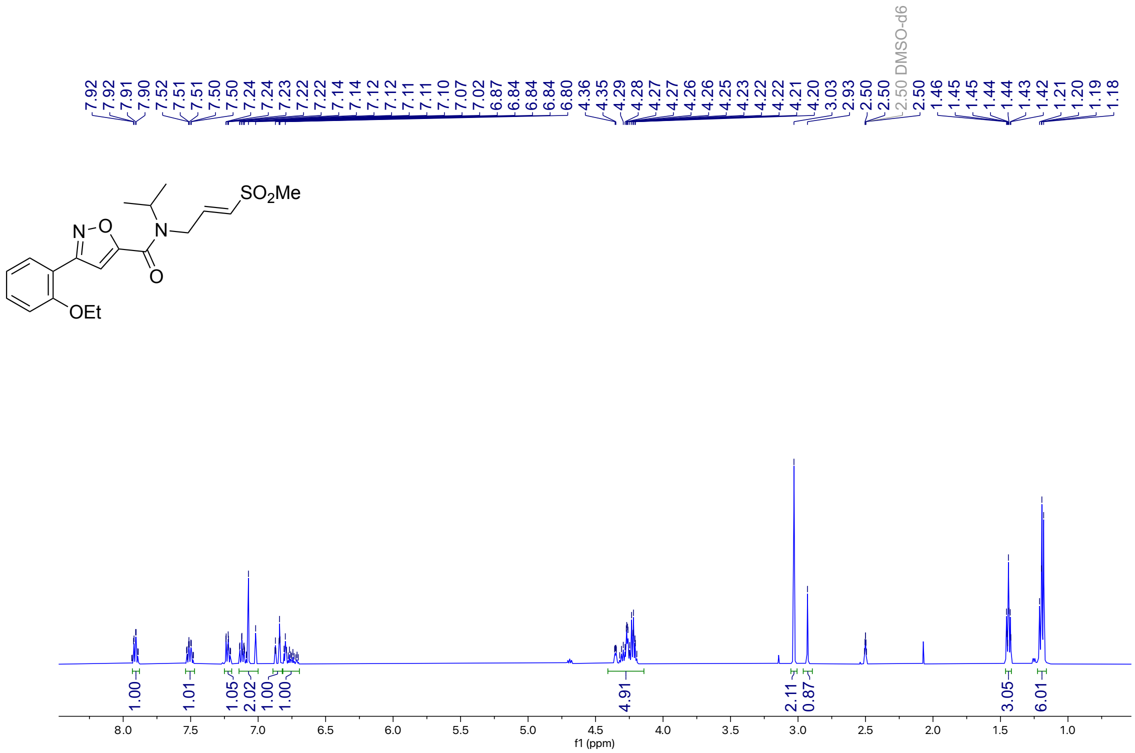

Figure S109: ^{13}C NMR (100 MHz, DMSO-d_6) for **23c**

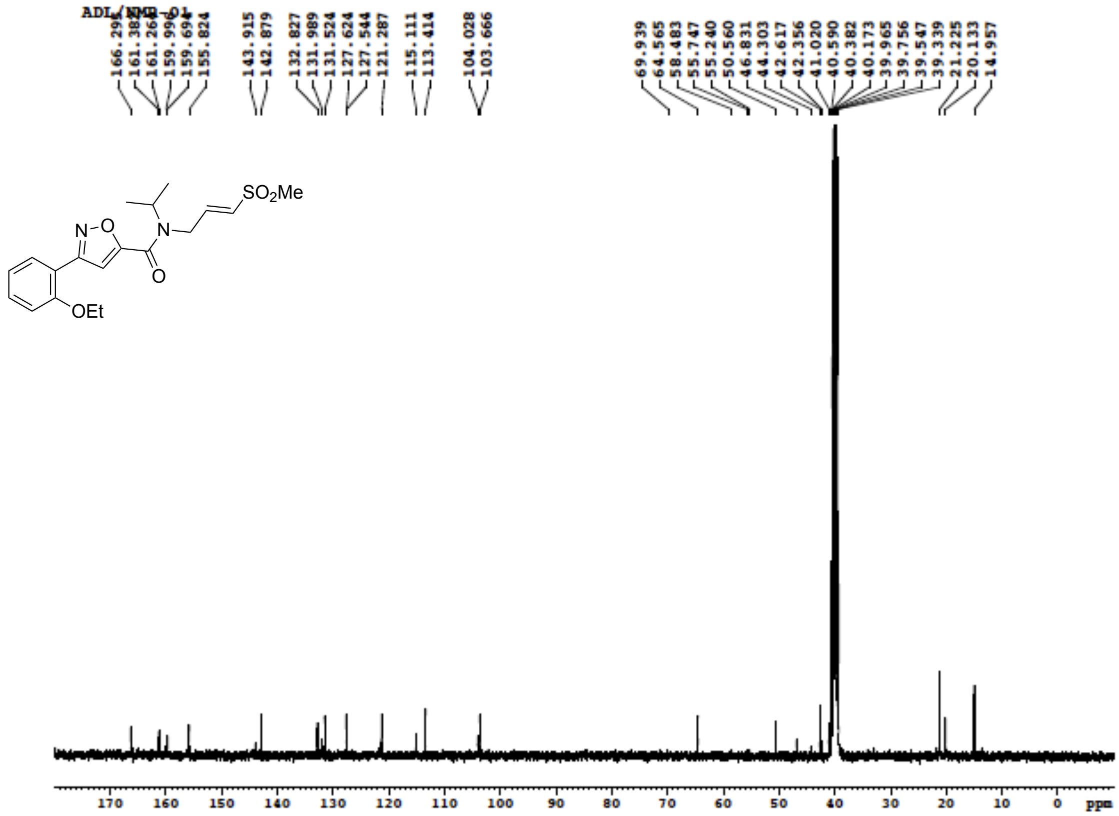

Figure S110: ^{1}H NMR (400 MHz, DMSO-d_6) for **23d**

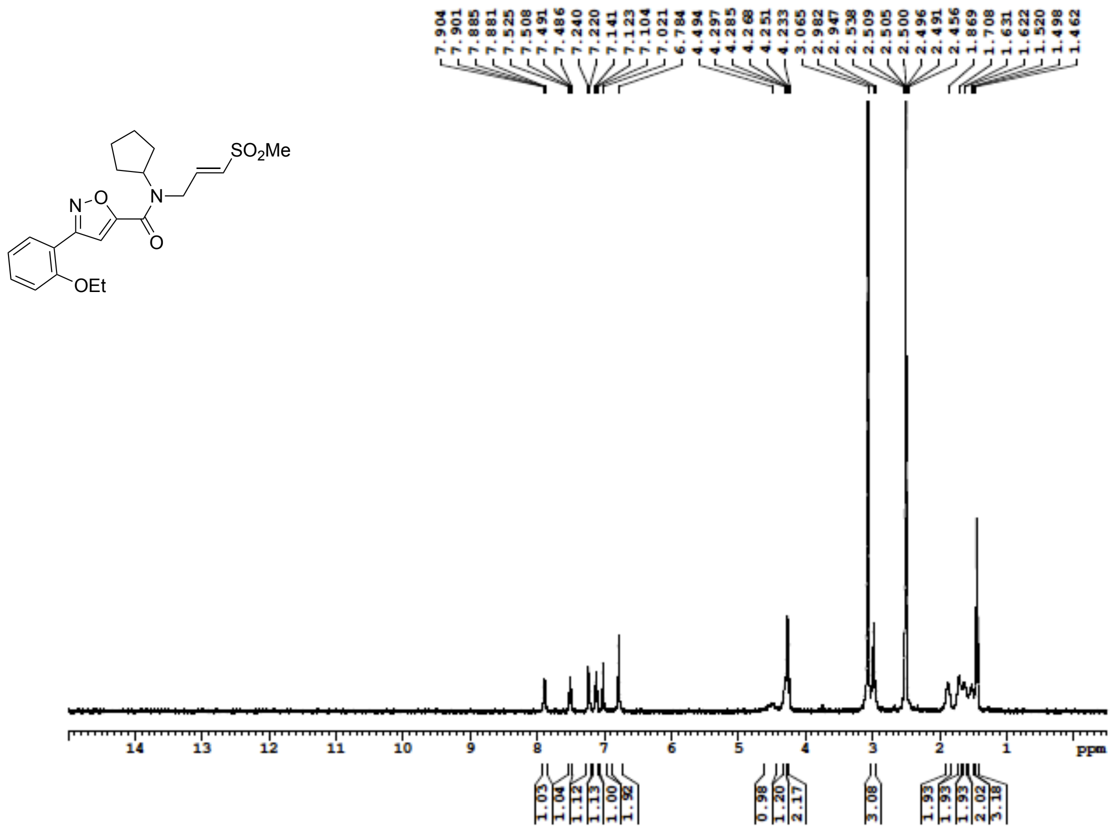

Figure S111: ^{13}C NMR (100 MHz, DMSO-d_6) for **23d**

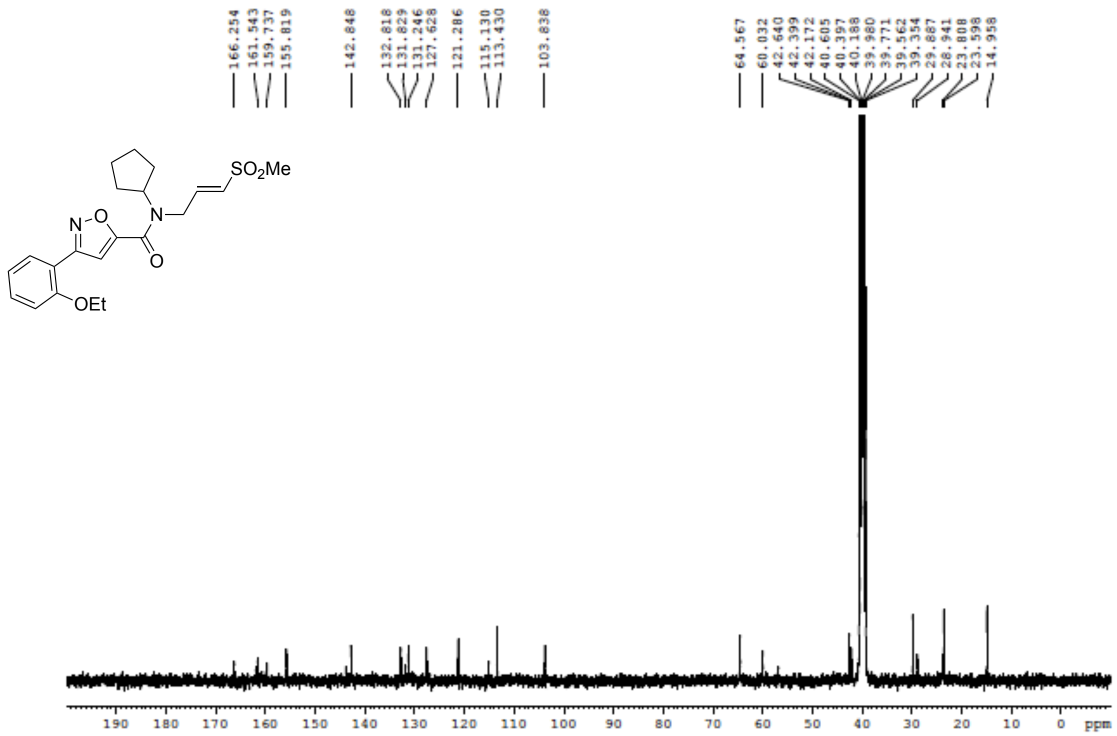

Figure S112: ^{1}H NMR (400 MHz, DMSO-d_6) for **23e**

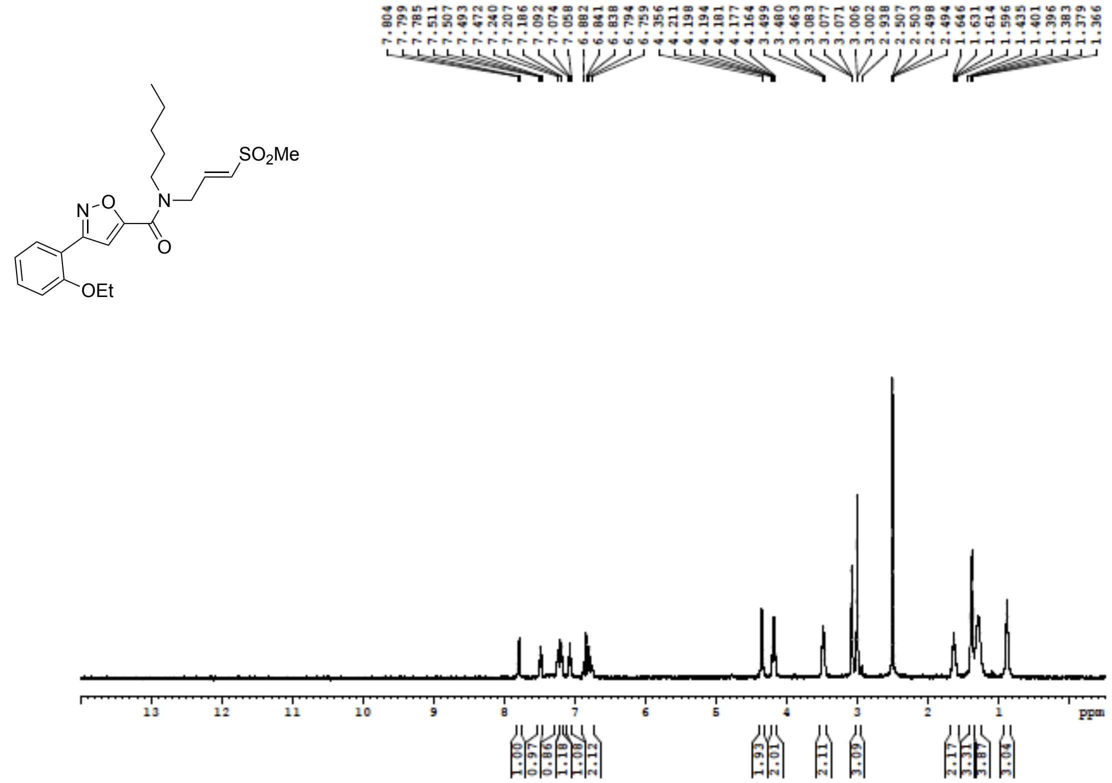

Figure S113: ^{13}C NMR (100 MHz, DMSO-d_6) for **23e**

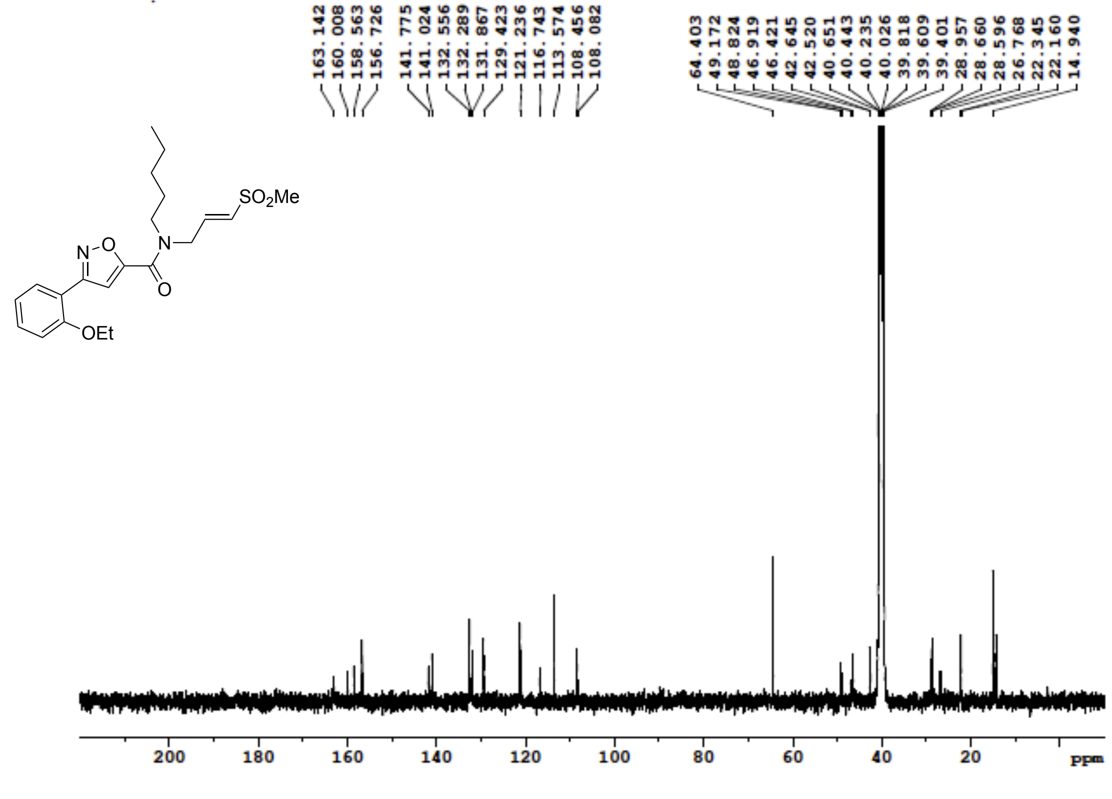

Figure S114: ^{1}H NMR (400 MHz, DMSO-d_6) for **23f**

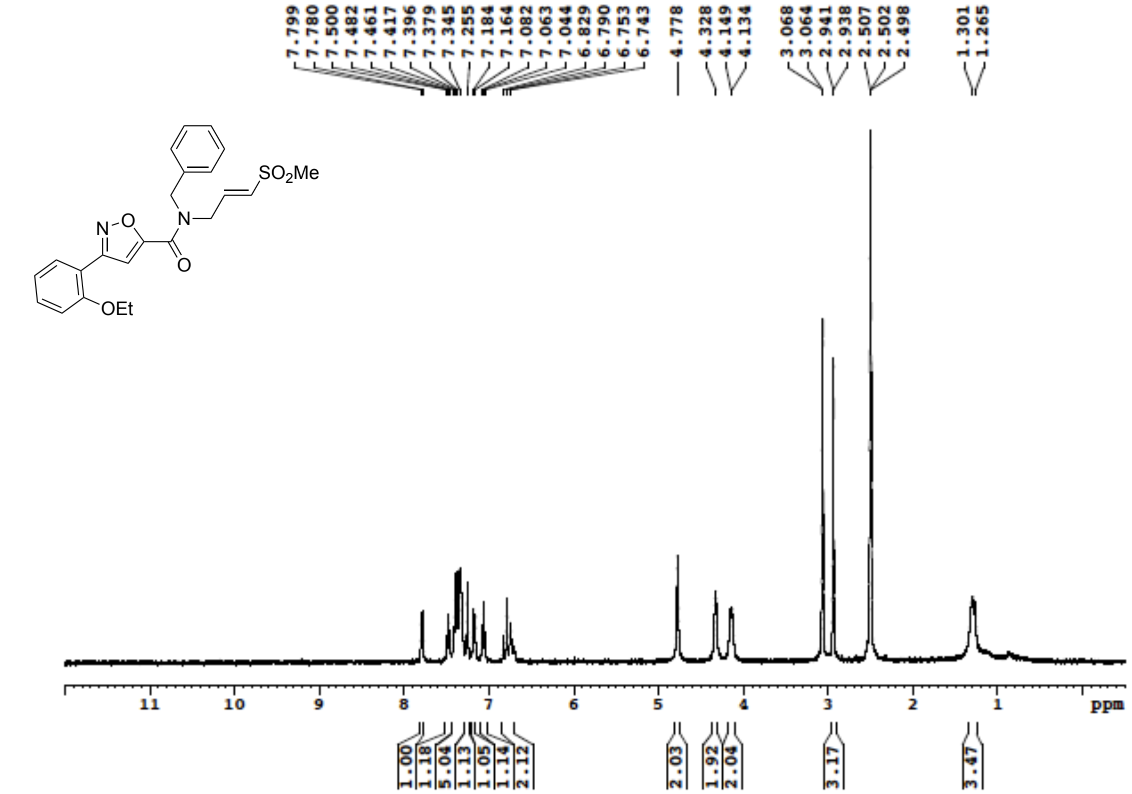

Figure S115: ^{13}C NMR (100 MHz, DMSO-d_6) for **23f**

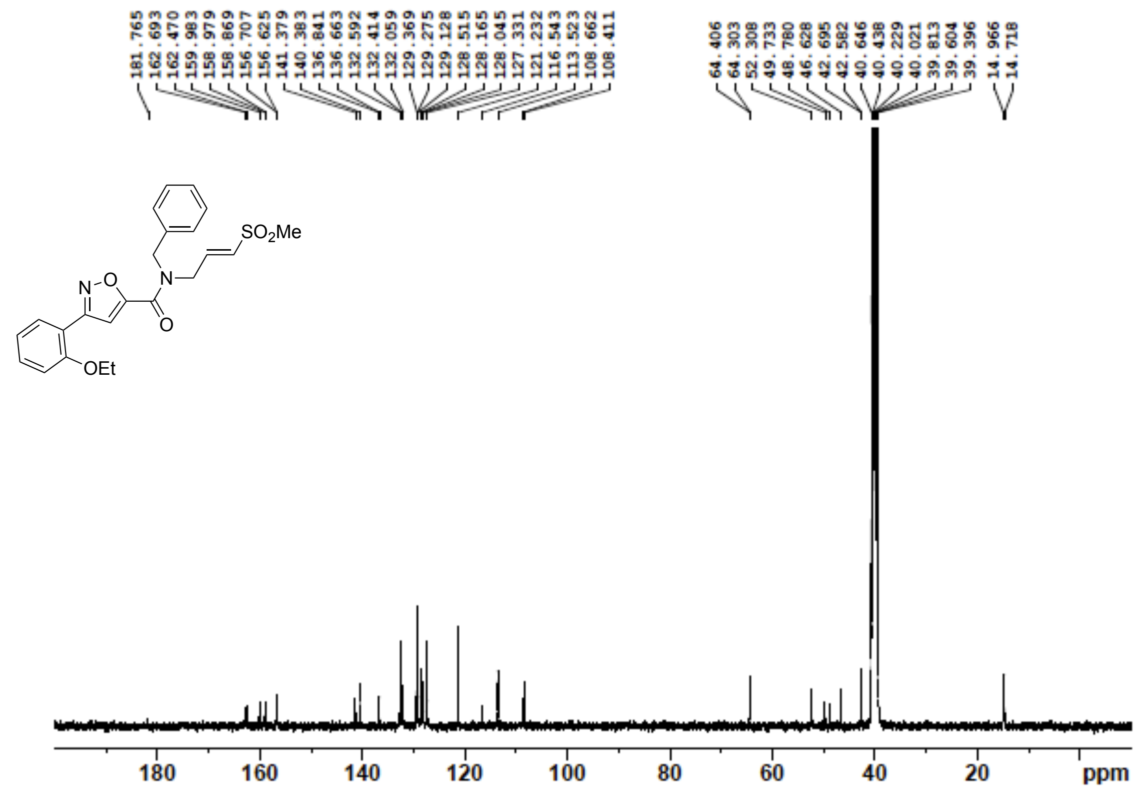

Figure S116: ^{1}H NMR (400 MHz, DMSO-d_6) for **24a**

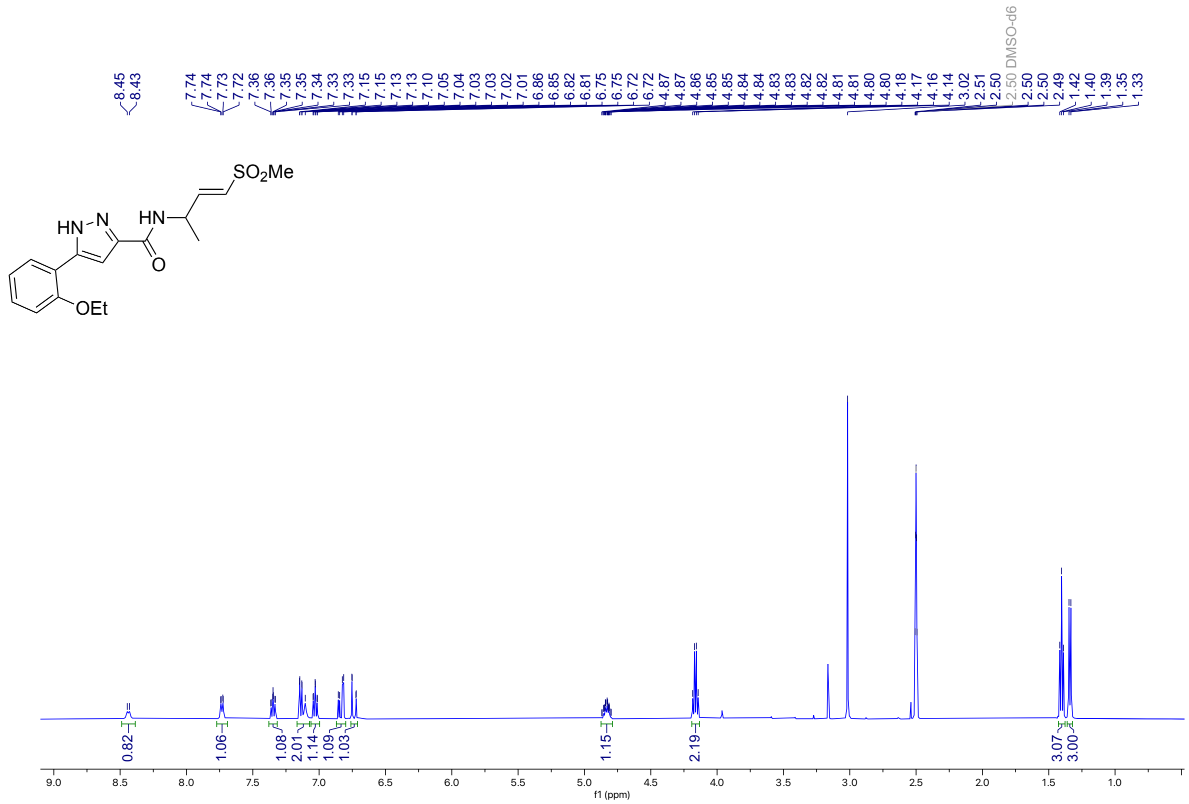

Figure S117: ^{13}C NMR (100 MHz, DMSO-d_6) for **24a**

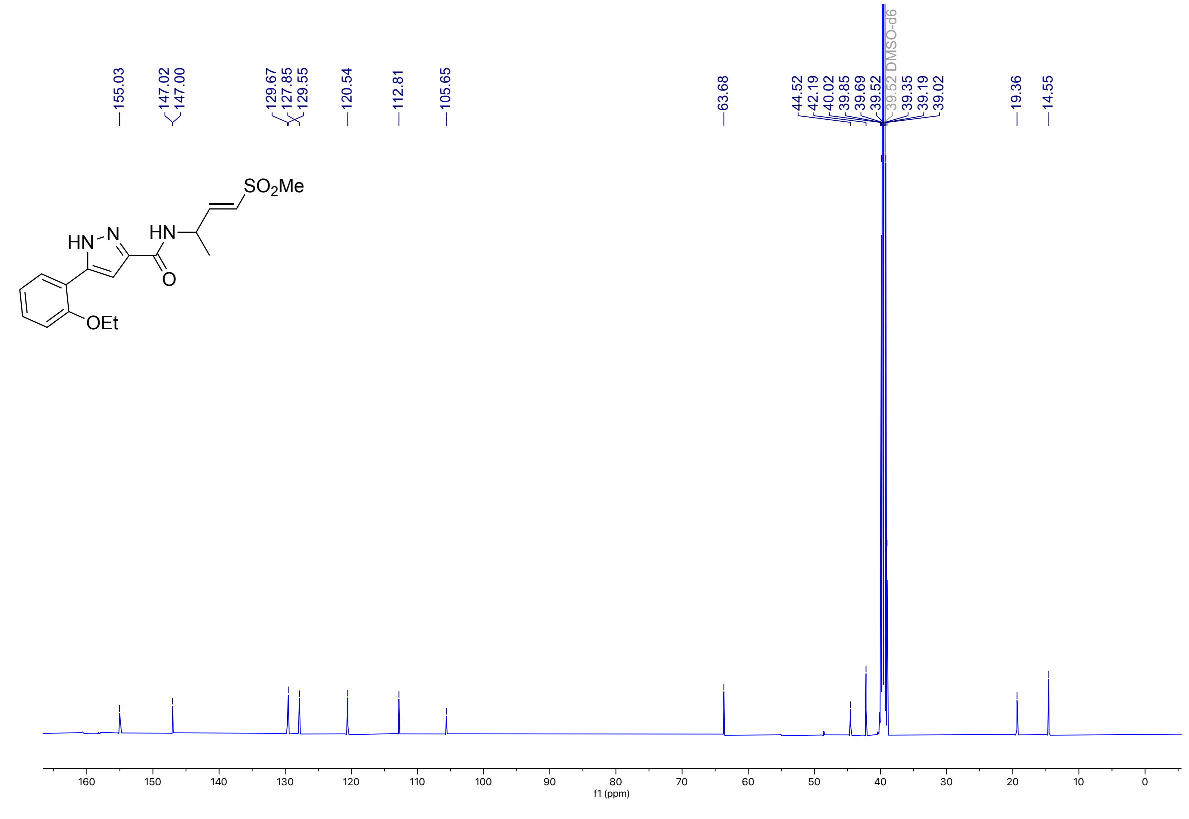

Figure S118: ^{1}H NMR (400 MHz, DMSO-d_6) for **24b**

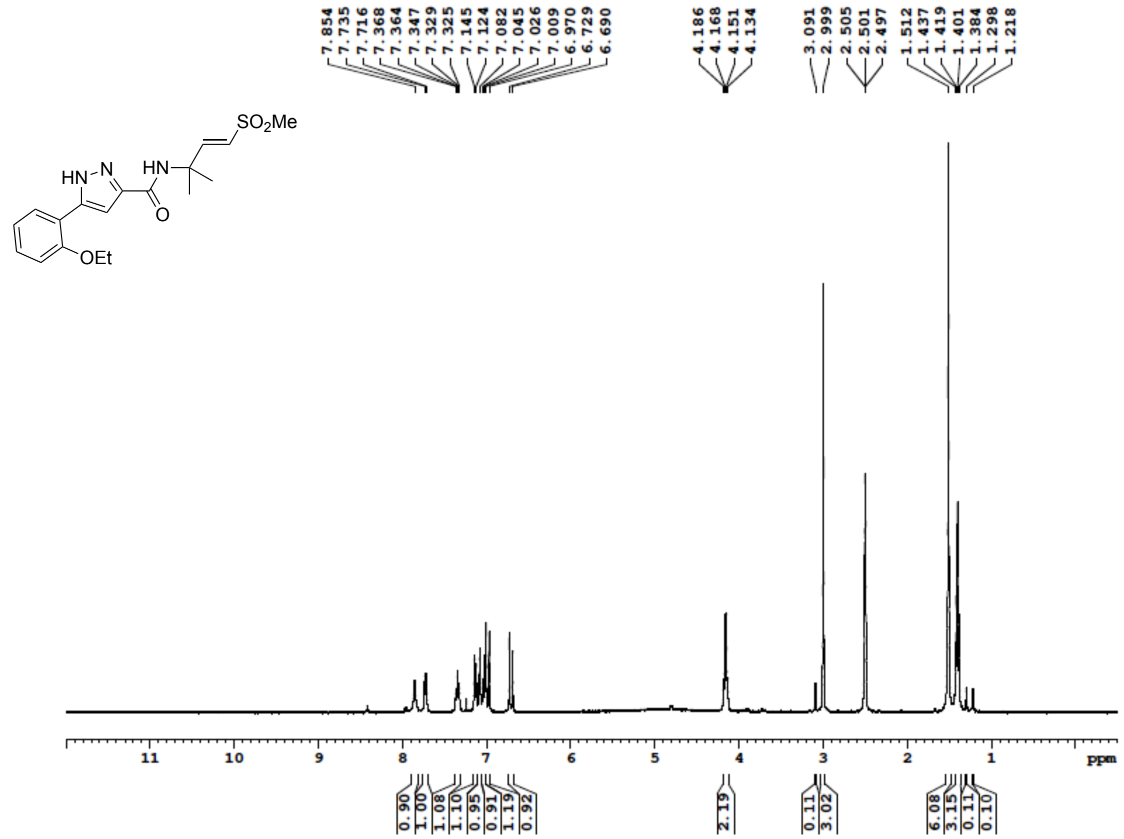

Figure S119: ^{13}C NMR (100 MHz, DMSO-d_6) for **24b**

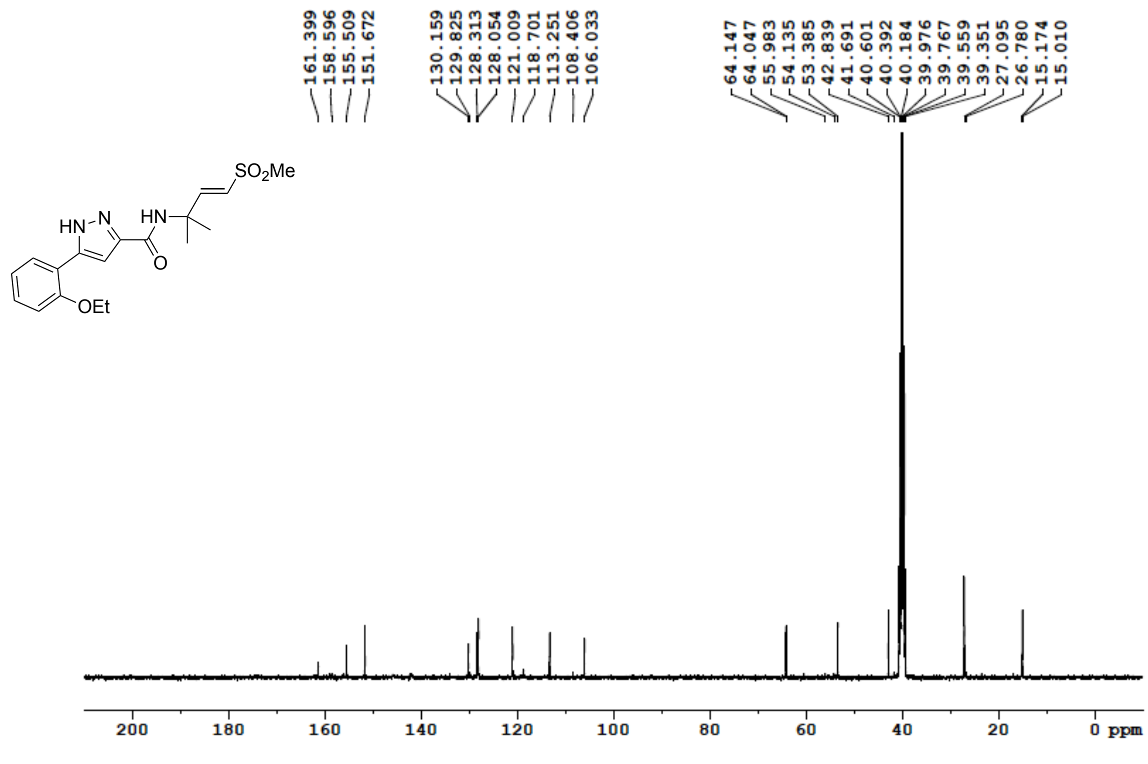

Figure S120: ^{1}H NMR (500 MHz, MeOD) for **24c**

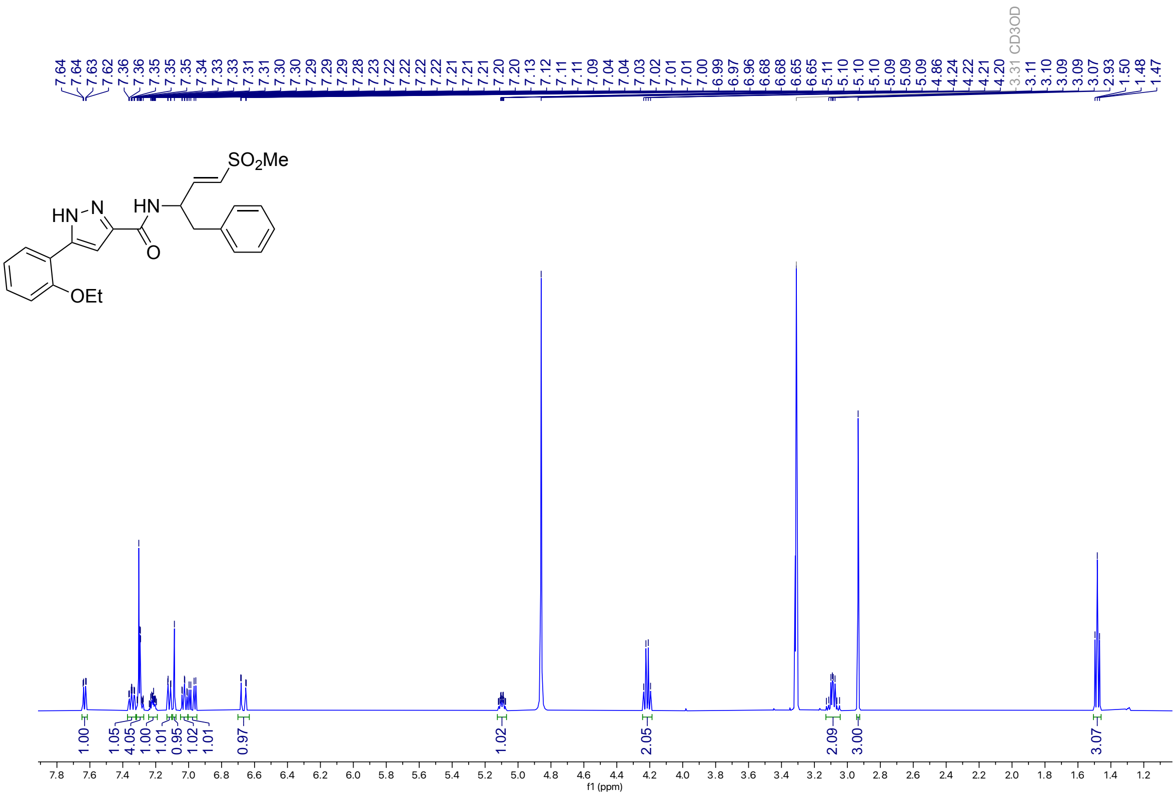

Figure S121: ^{13}C NMR (126 MHz, MeOD) for **24c**

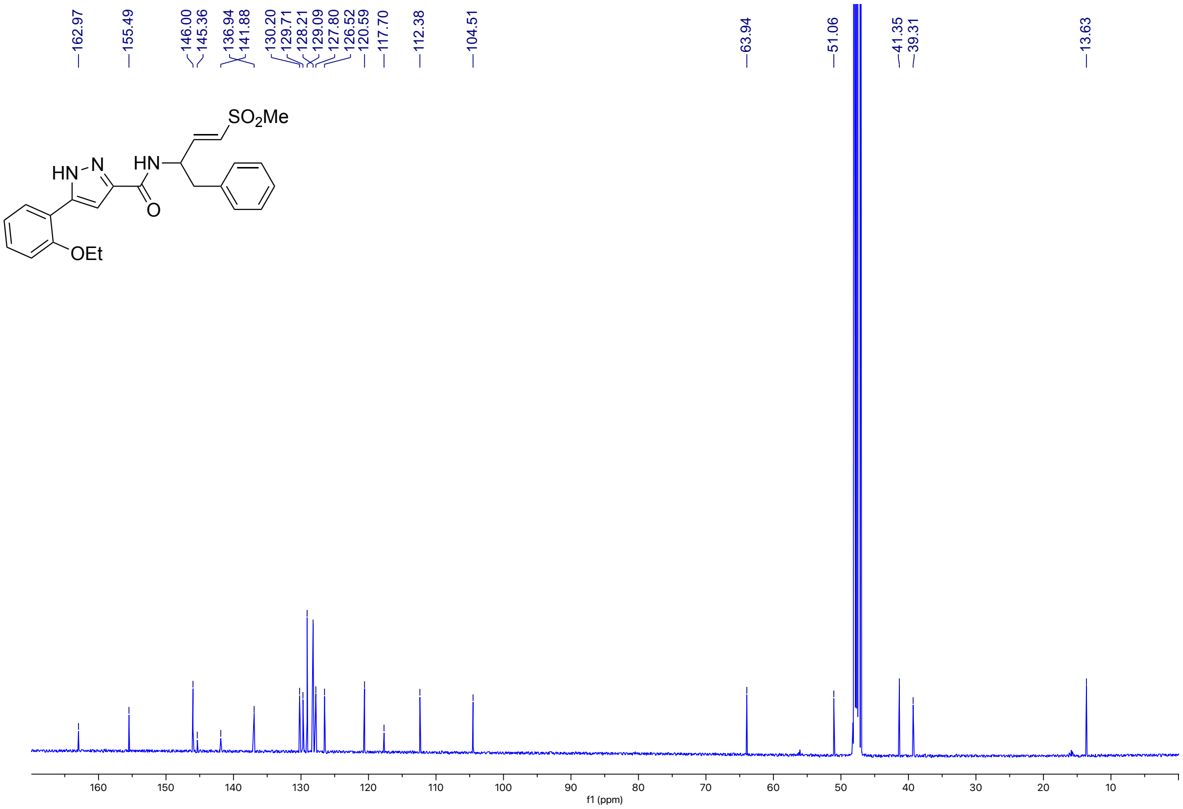

Figure S122: ^{1}H NMR (400 MHz, DMSO-d_6) for **24d**

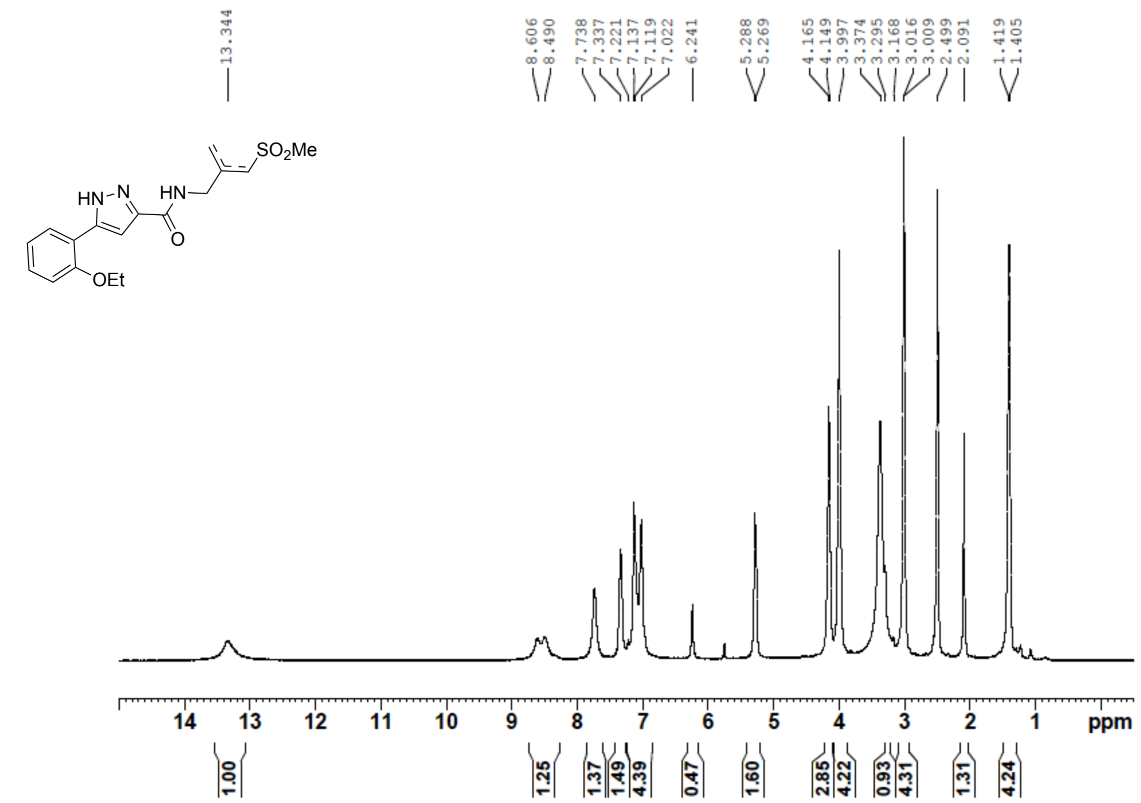

Figure S123: ^{13}C NMR (100 MHz, DMSO-d_6) for **24d**

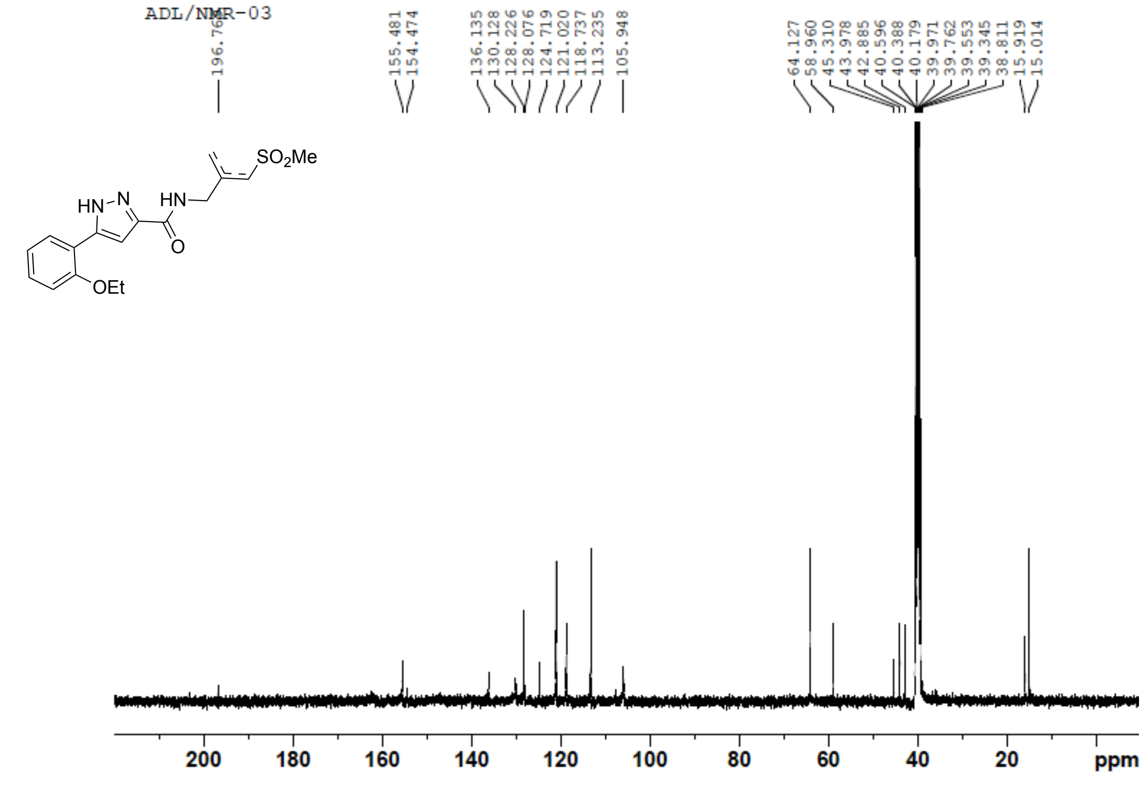

Figure S124: ^{1}H NMR (400 MHz, DMSO-d_6) for **24**e

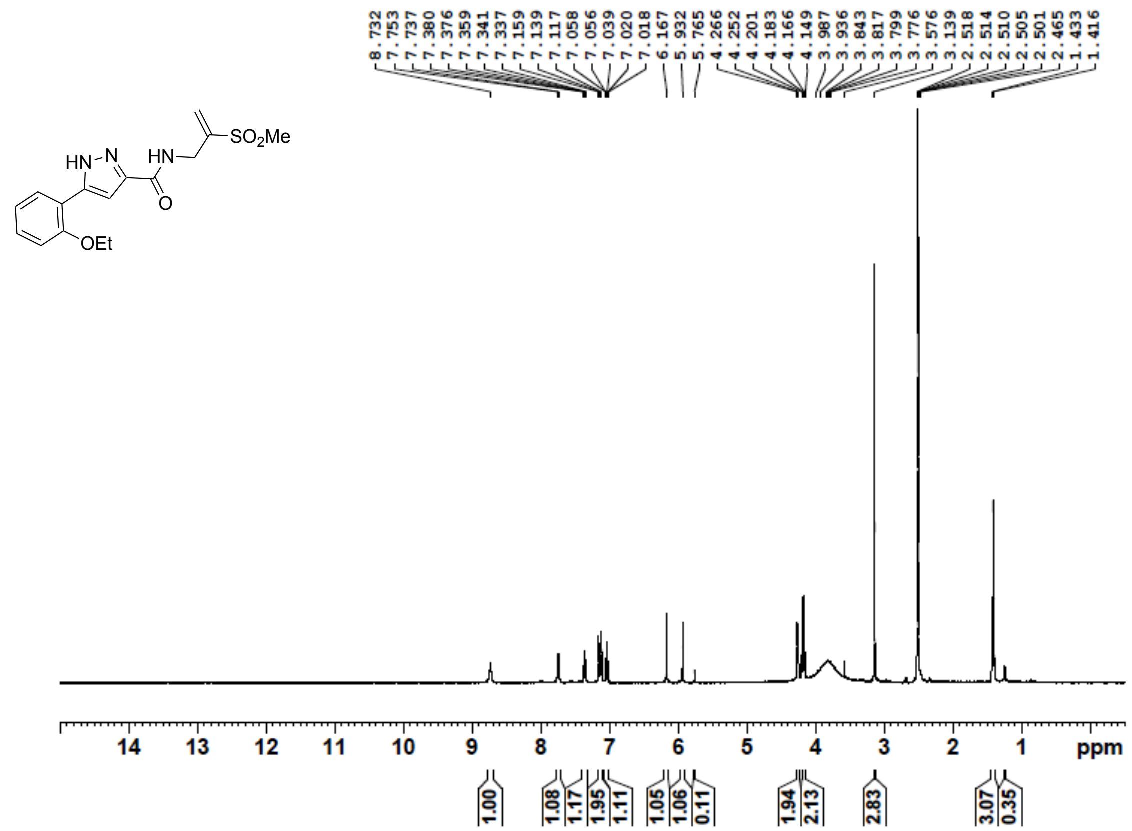

Figure S125: ^{13}C NMR (100 MHz, DMSO-d_6) for **24e**

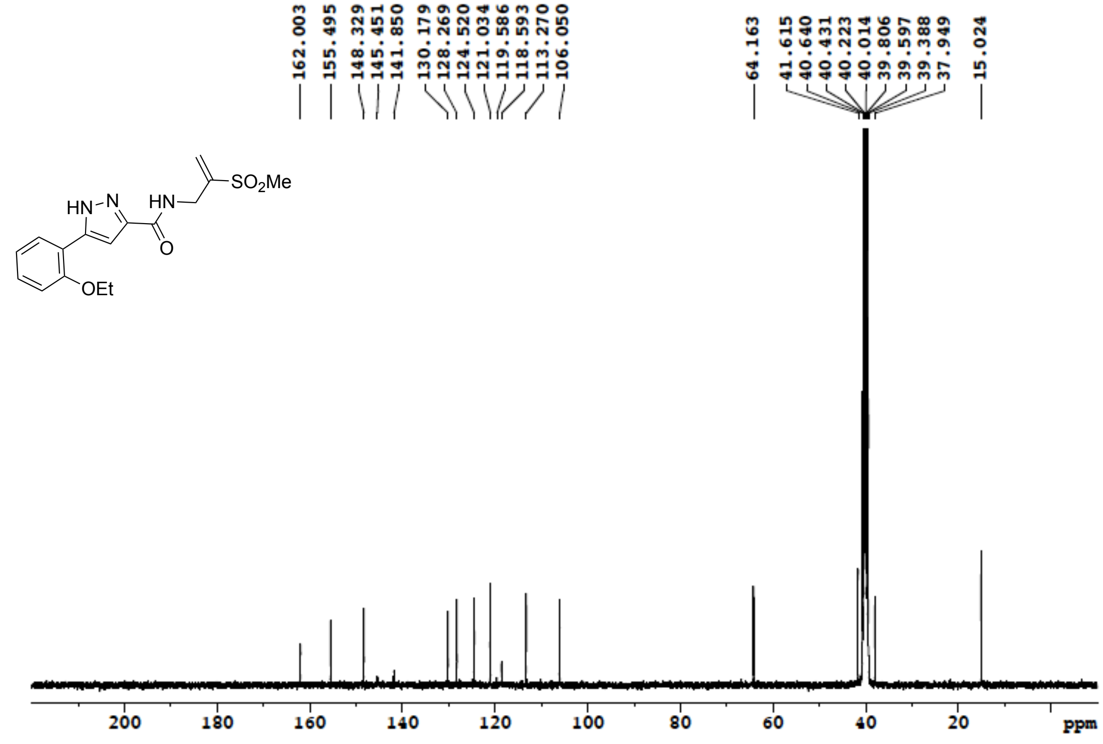

Figure S126: ^{1}H NMR (400 MHz, DMSO-d_6) for **24f**

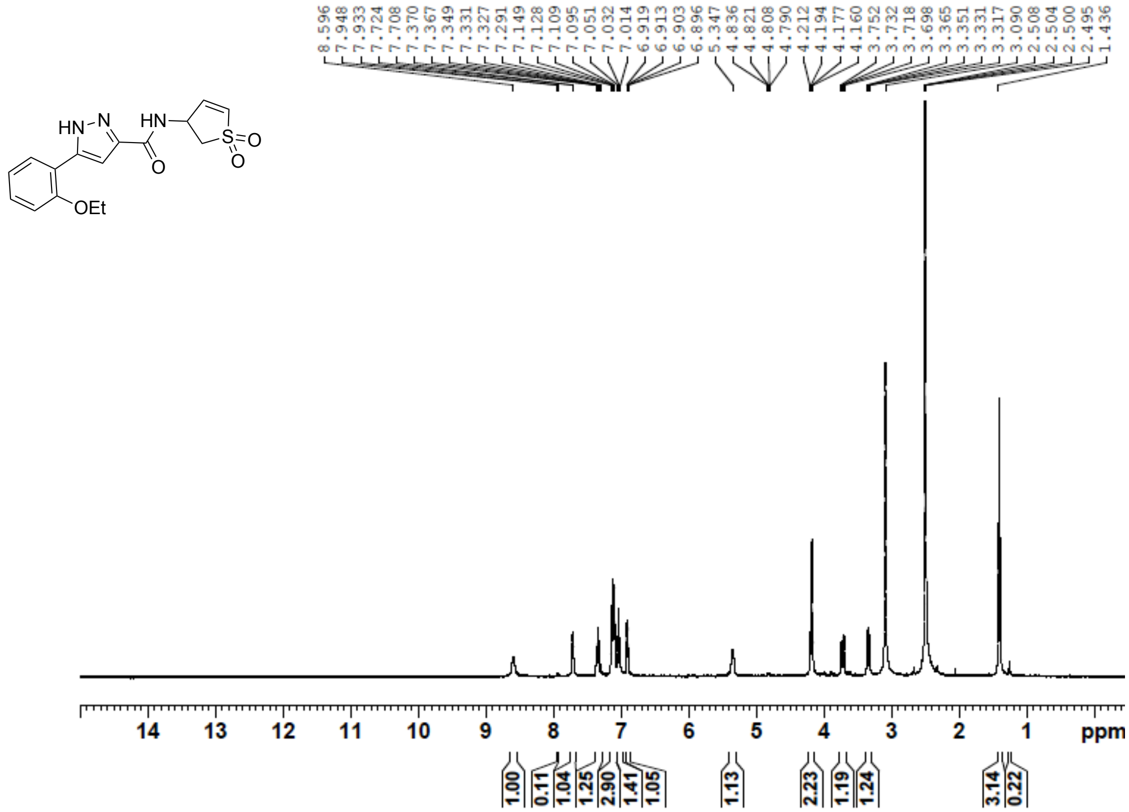

Figure S127: ^{13}C NMR (100 MHz, DMSO-d_6) for **24f**

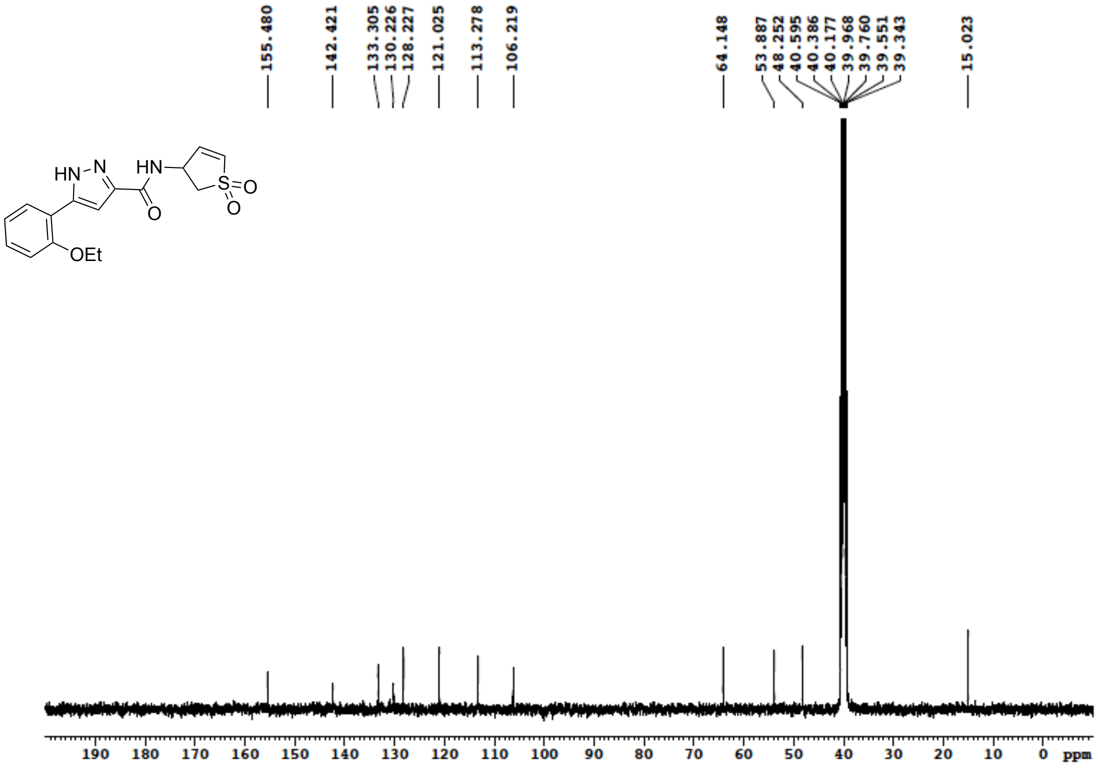

Figure S128: ^{1}H NMR (400 MHz, DMSO-d_6) for **25a**

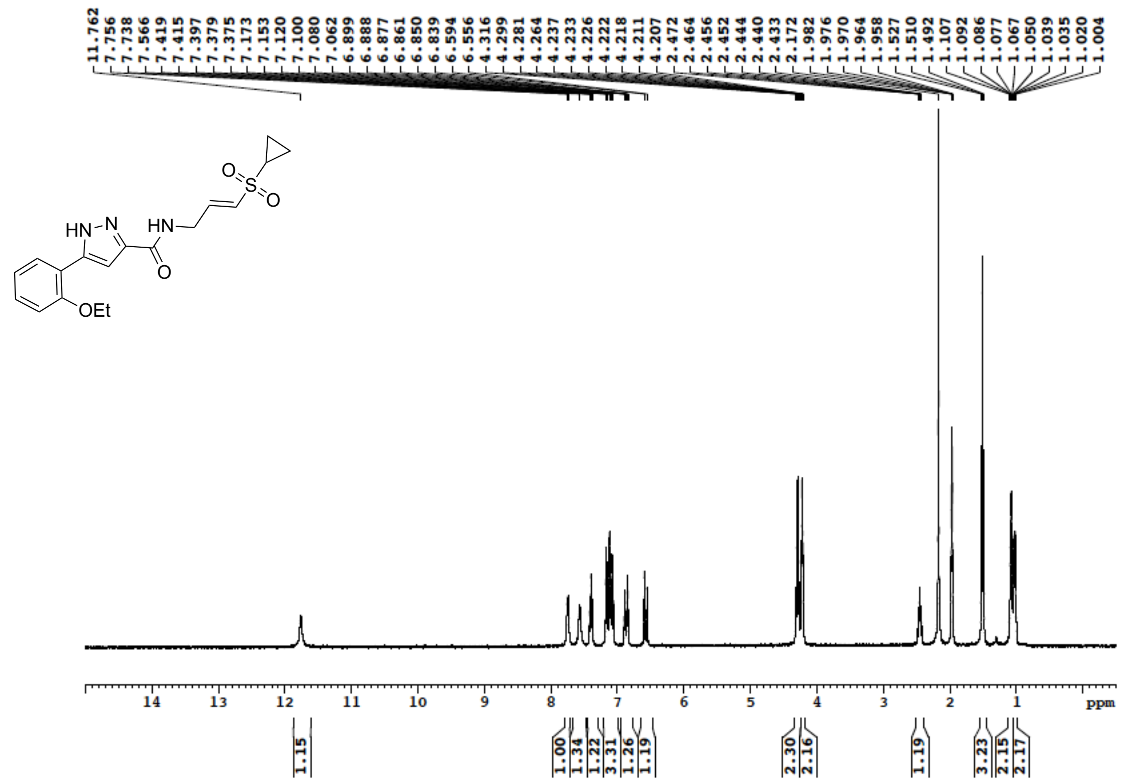

Figure S129: ^{13}C NMR (100 MHz, DMSO-d_6) for **25a**

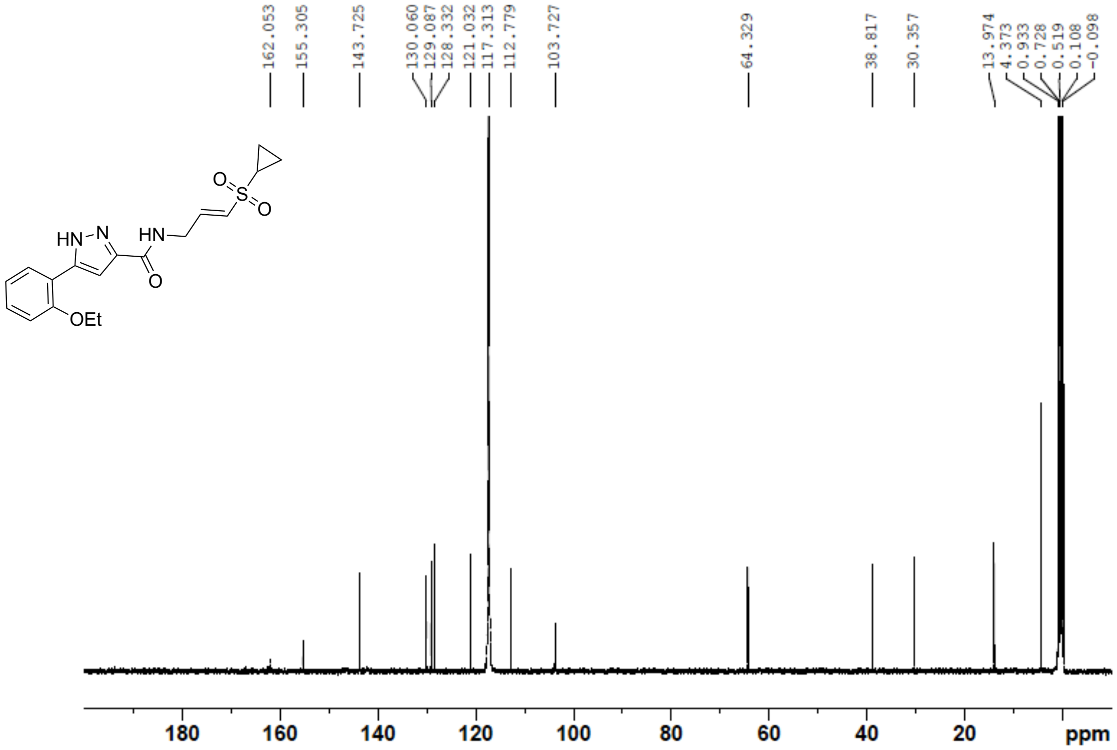

Figure S130: ^{1}H NMR (400 MHz, DMSO-d_6) for **25b**

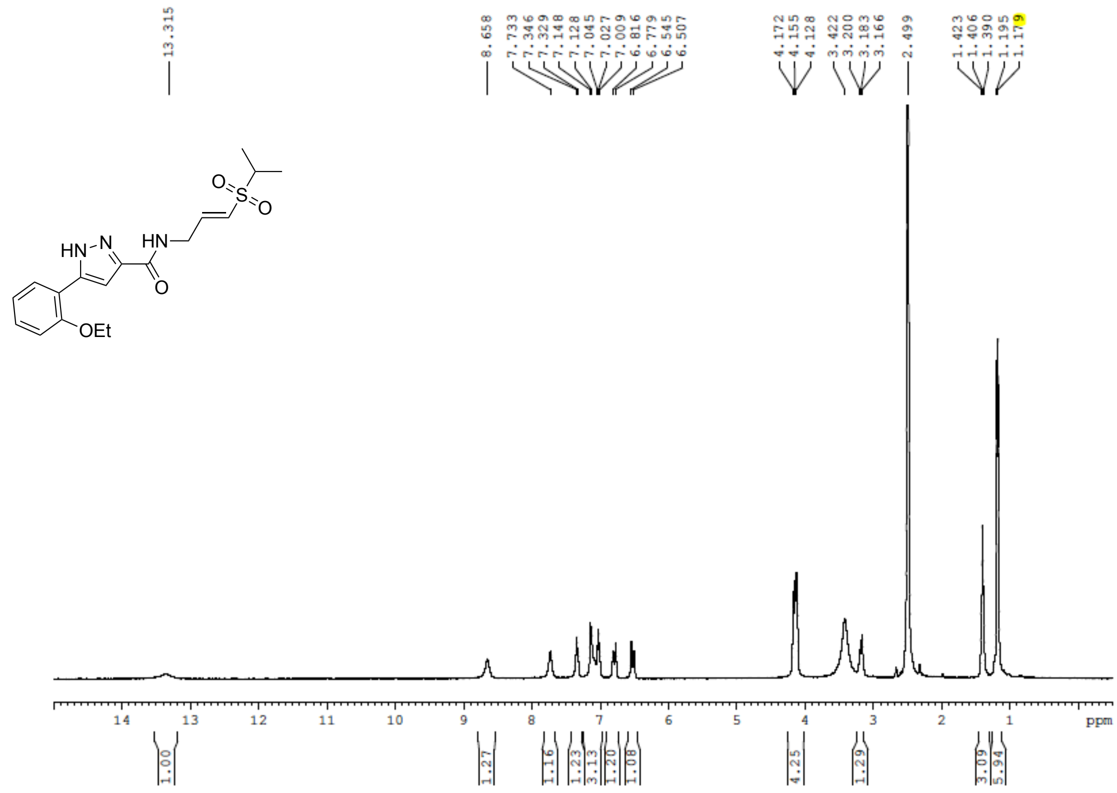

Figure S131: ^{13}C NMR (100 MHz, DMSO-d_6) for **25b**

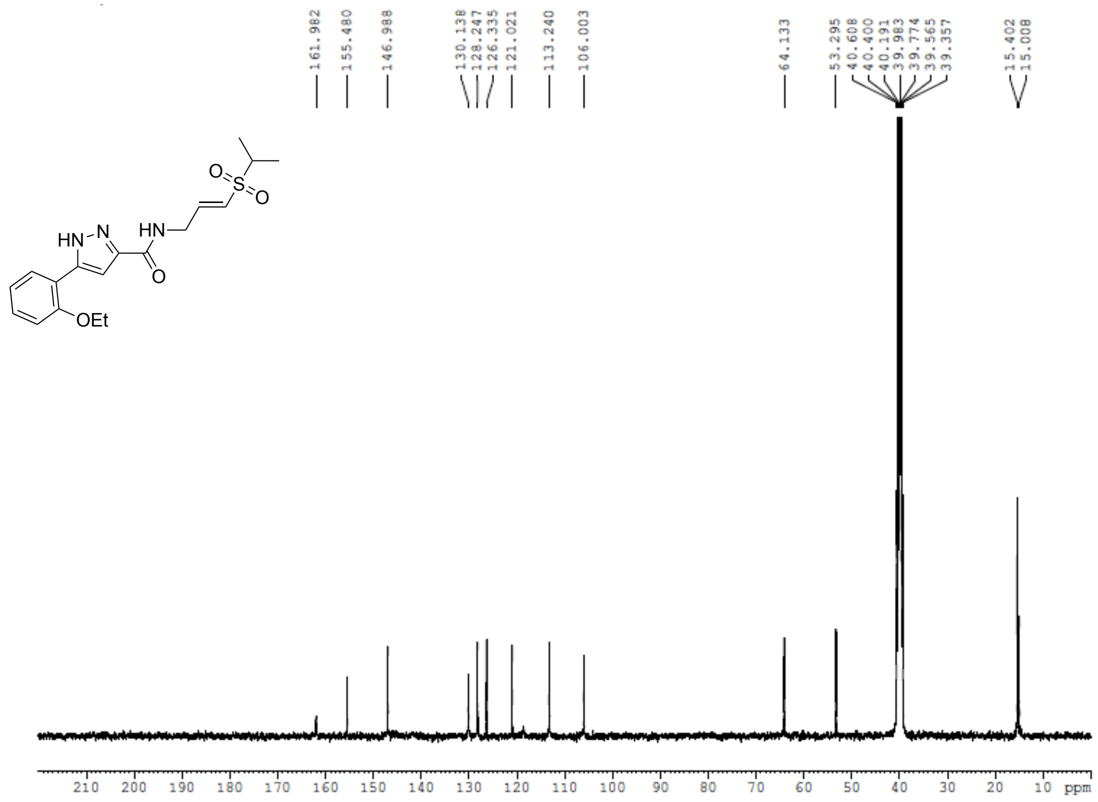

Figure S132: ^{1}H NMR (400 MHz, DMSO-d_6) for **25c**

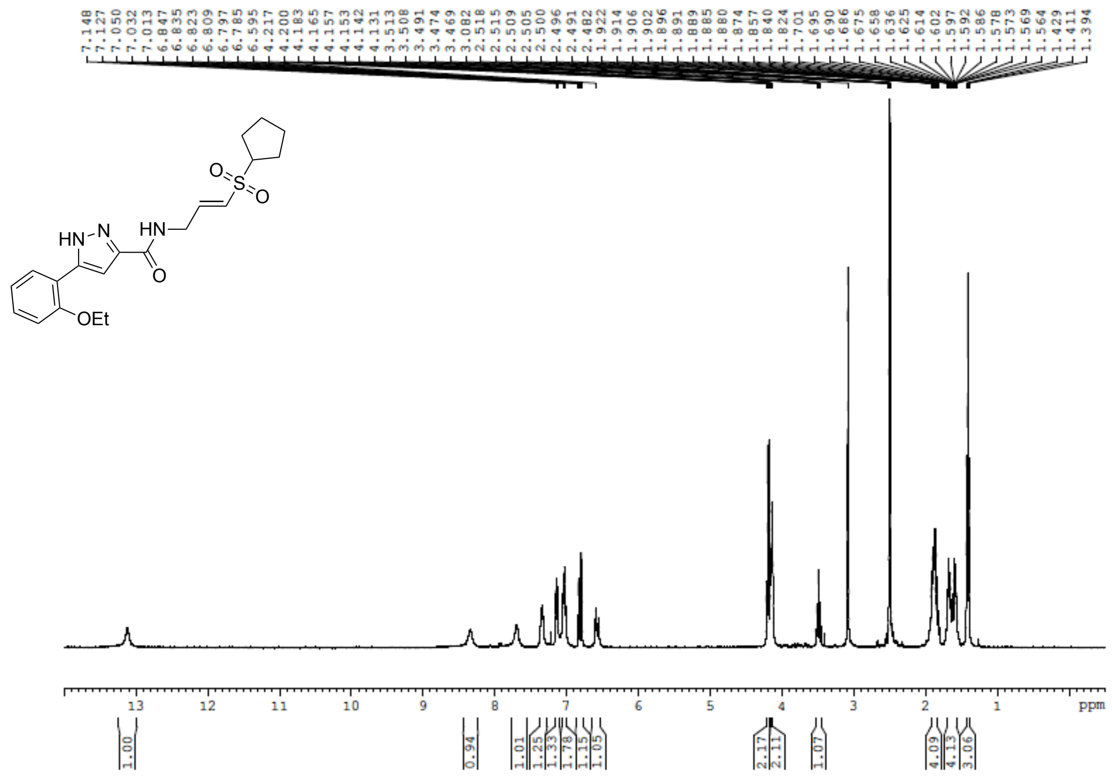

Figure S133: ^{13}C NMR (100 MHz, DMSO-d_6) for **25c**

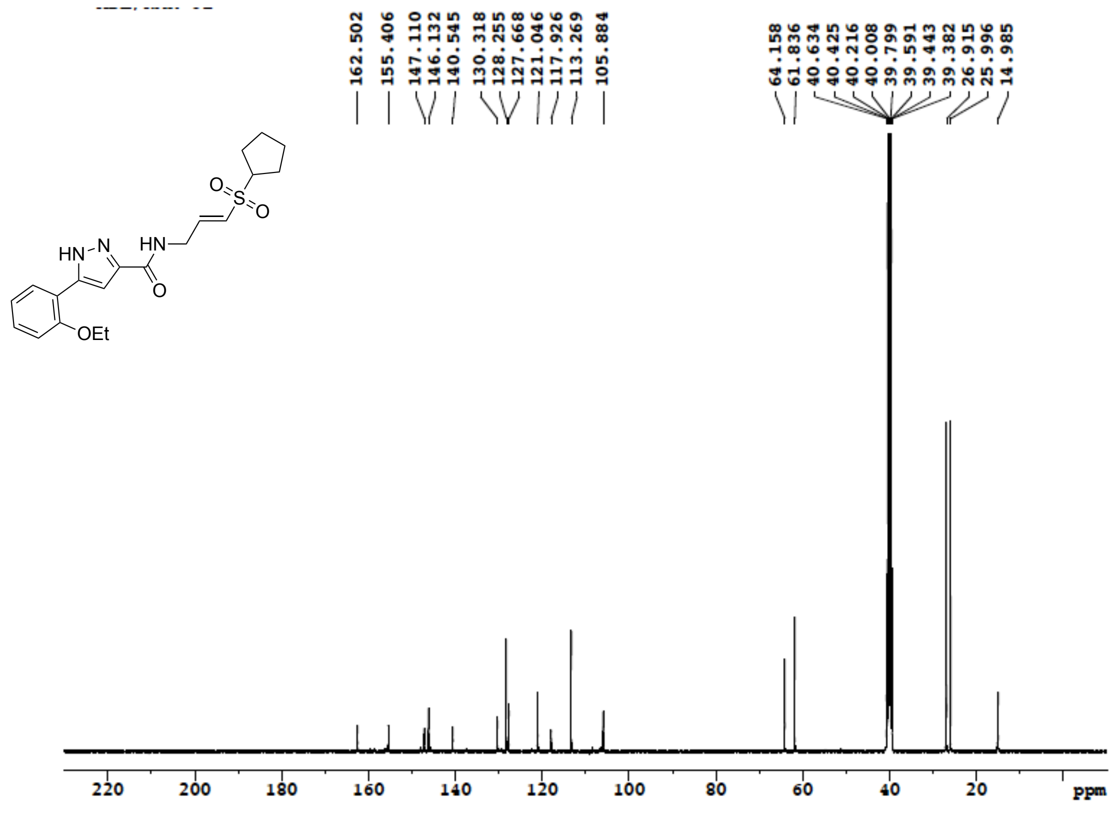

Figure S134: ^{1}H NMR (500 MHz, DMSO-d_6) for **25d**

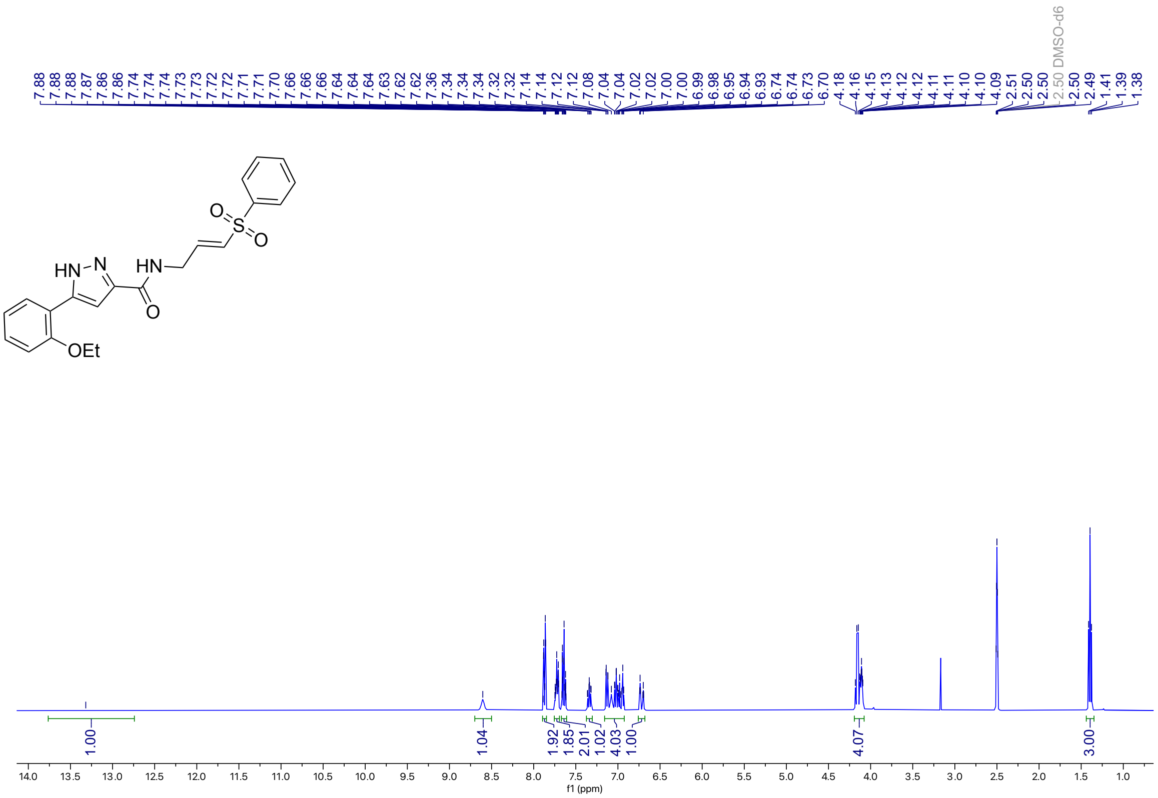

Figure S135: ^{13}C NMR (126 MHz, DMSO-d_6) for **25d**

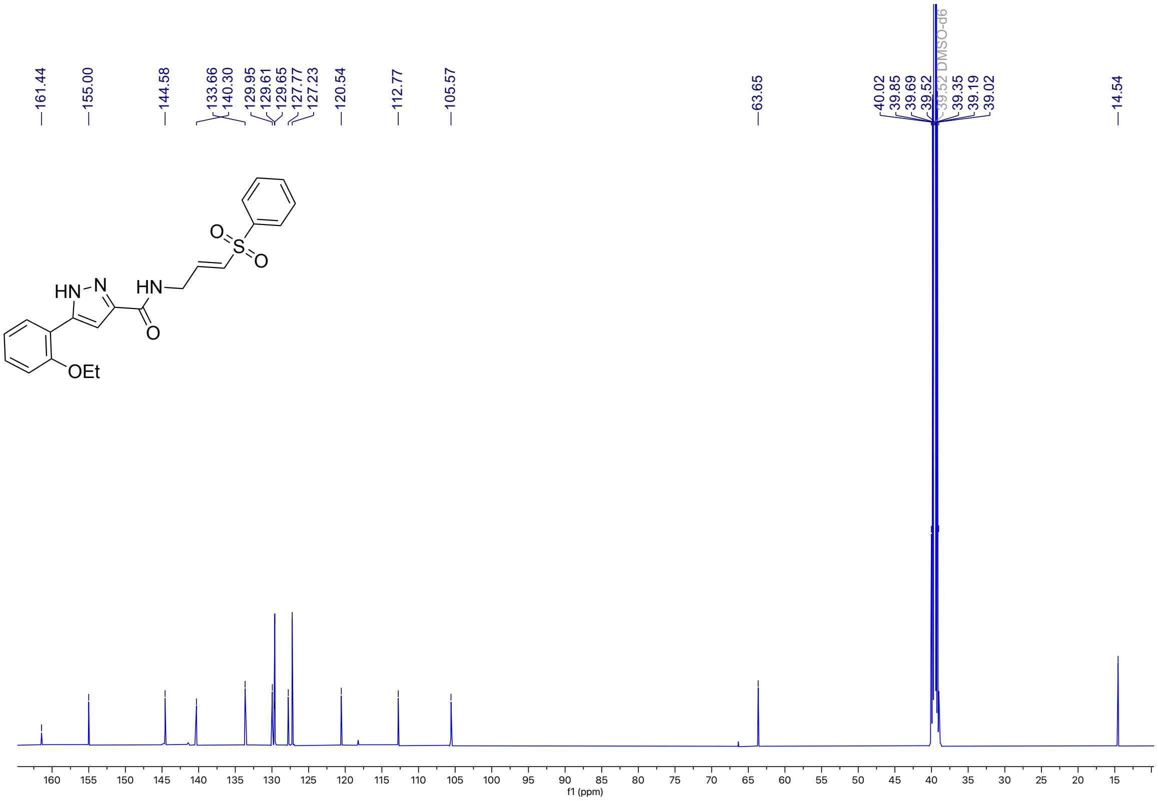

Figure S136: ^{1}H NMR (400 MHz, DMSO-d_6) for **25e**

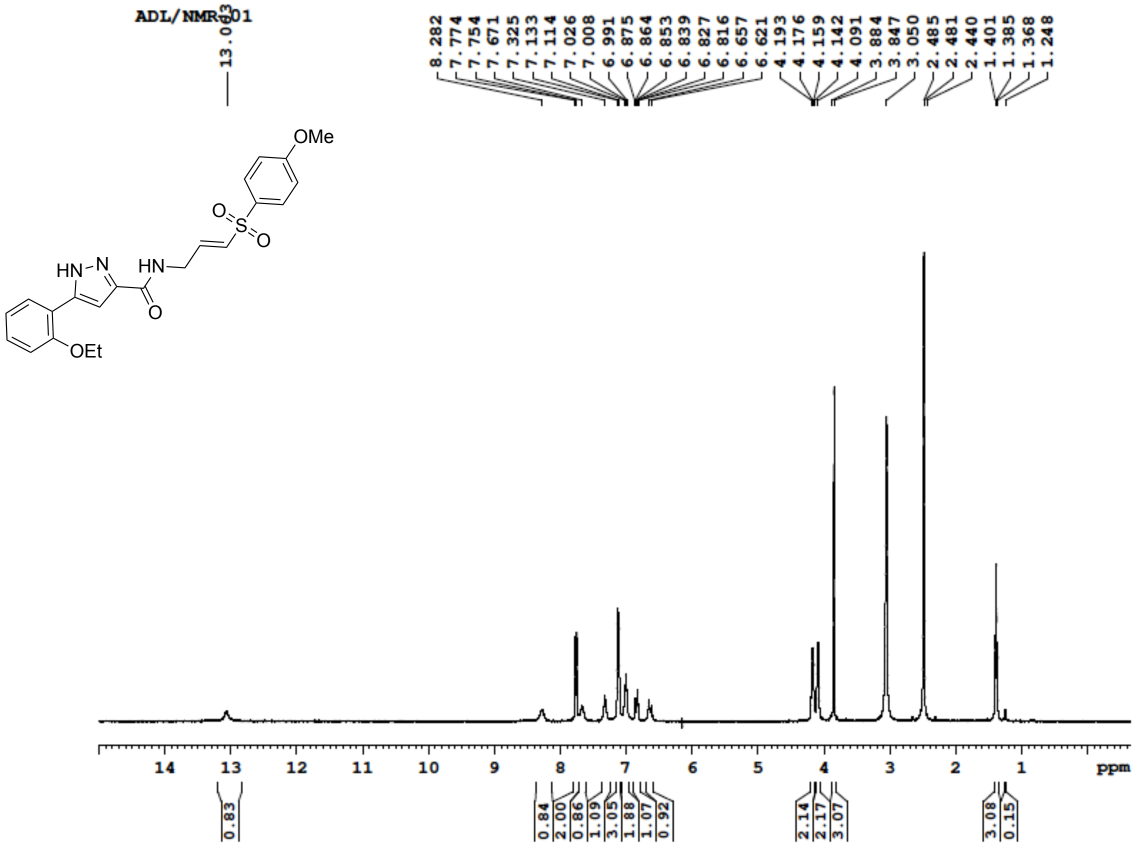

Figure S137: ^{13}C NMR (100 MHz, DMSO-d_6) for **25e**

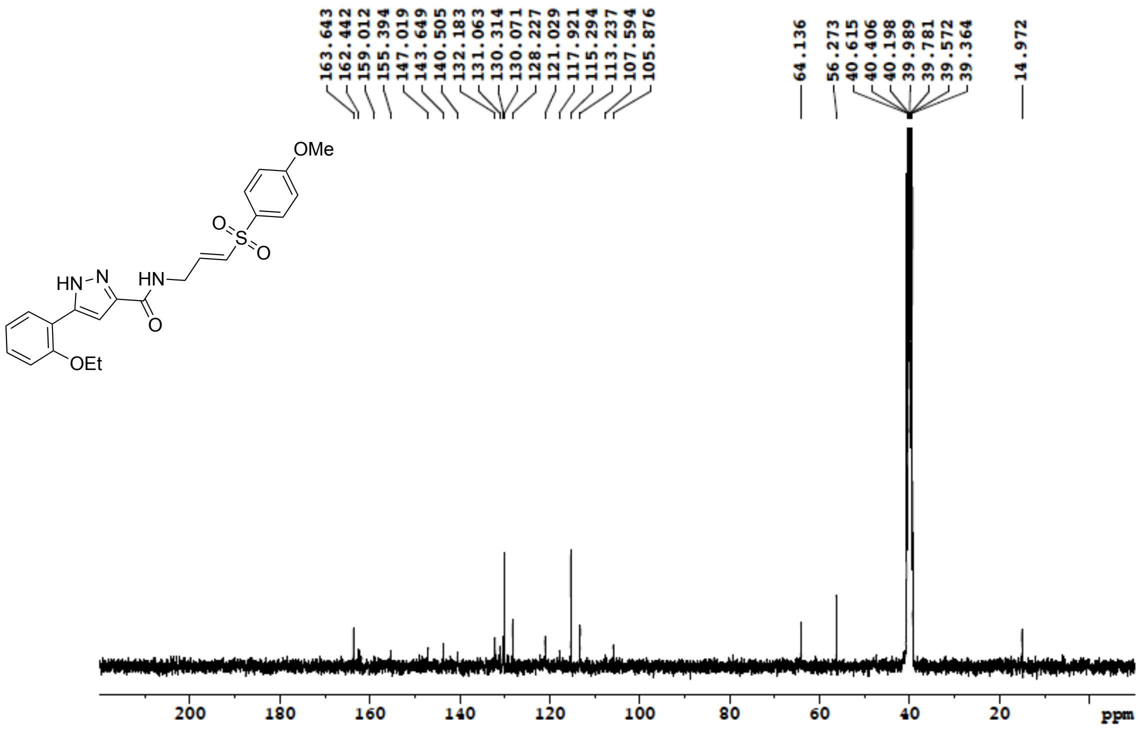

Figure S138: ^{1}H NMR (500 MHz, MeOD) for **25f**

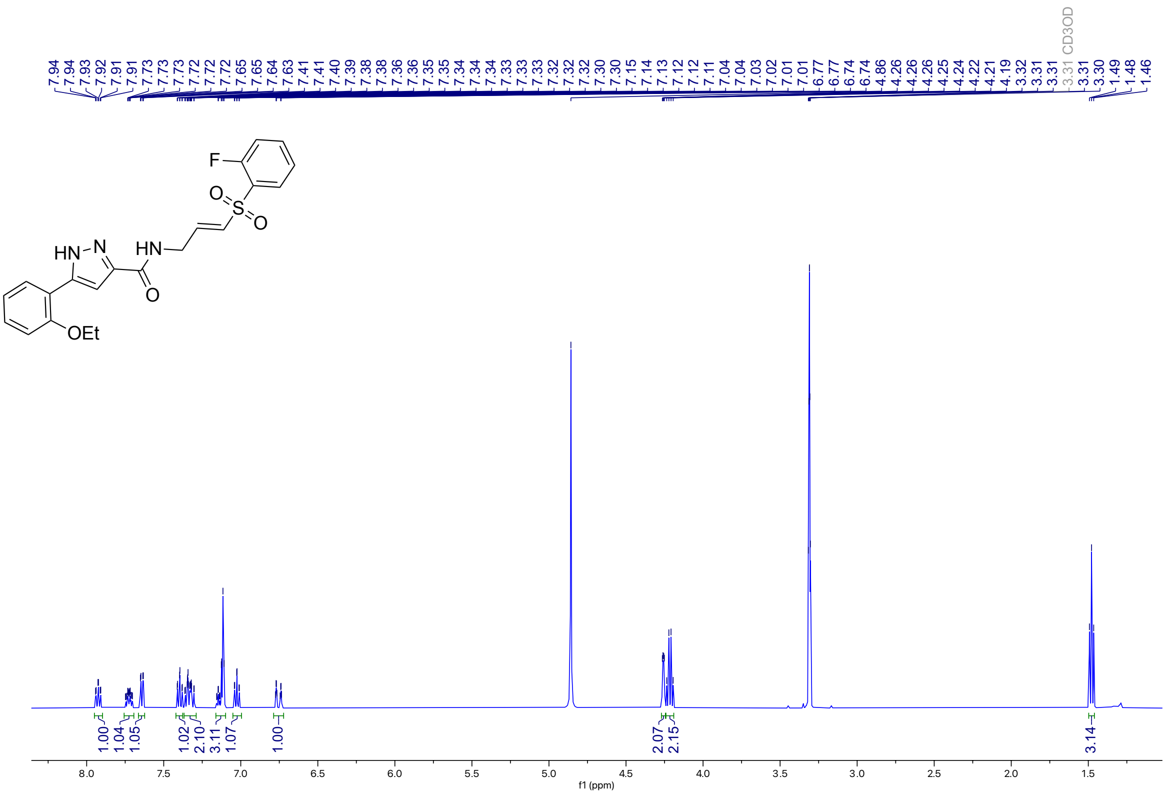

Figure S139: ^{13}C NMR (100 MHz, DMSO-d_6) for **25f**

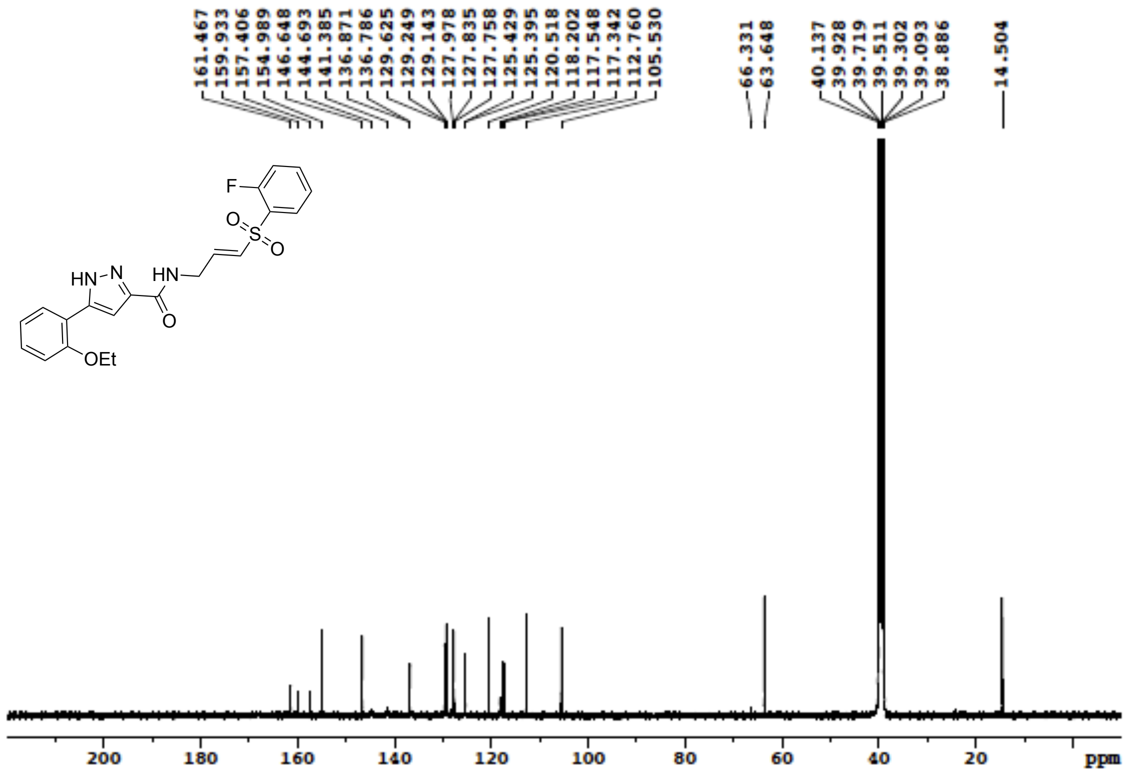

Figure S140: ^{1}H NMR (400 MHz, DMSO-d_6) for **25g**

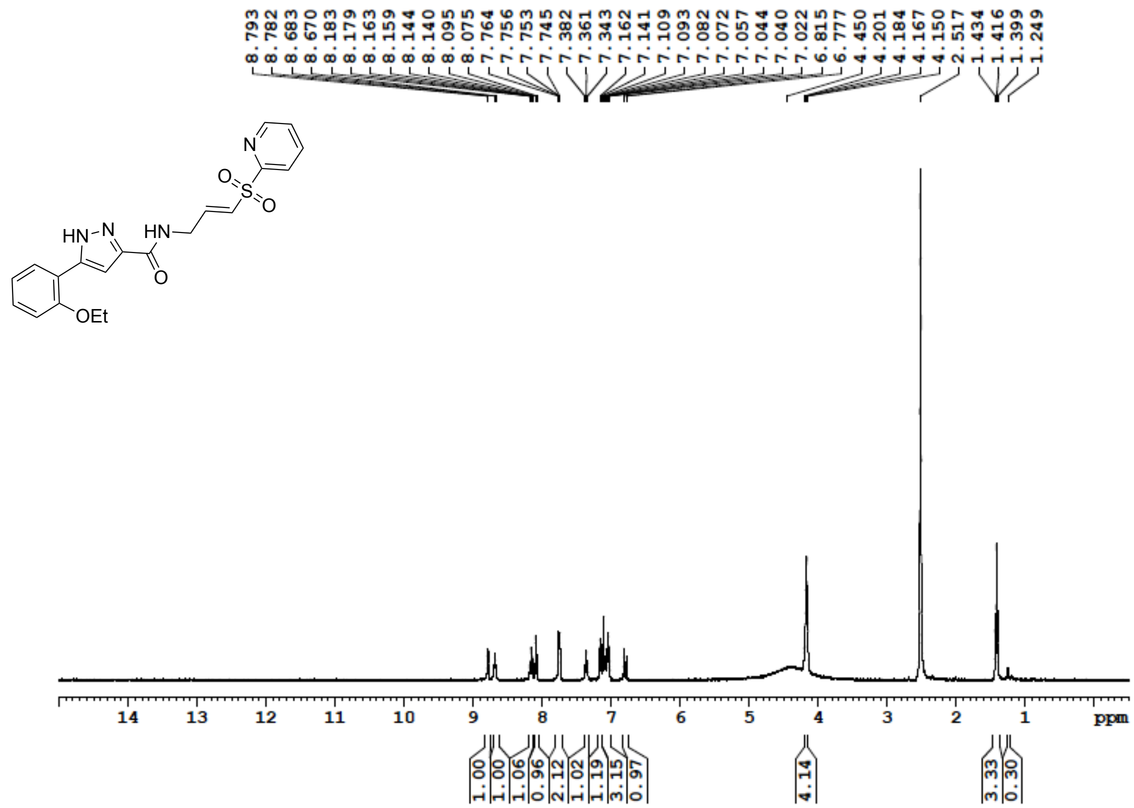

Figure S141: ^{13}C NMR (100 MHz, DMSO-d_6) for **25g**

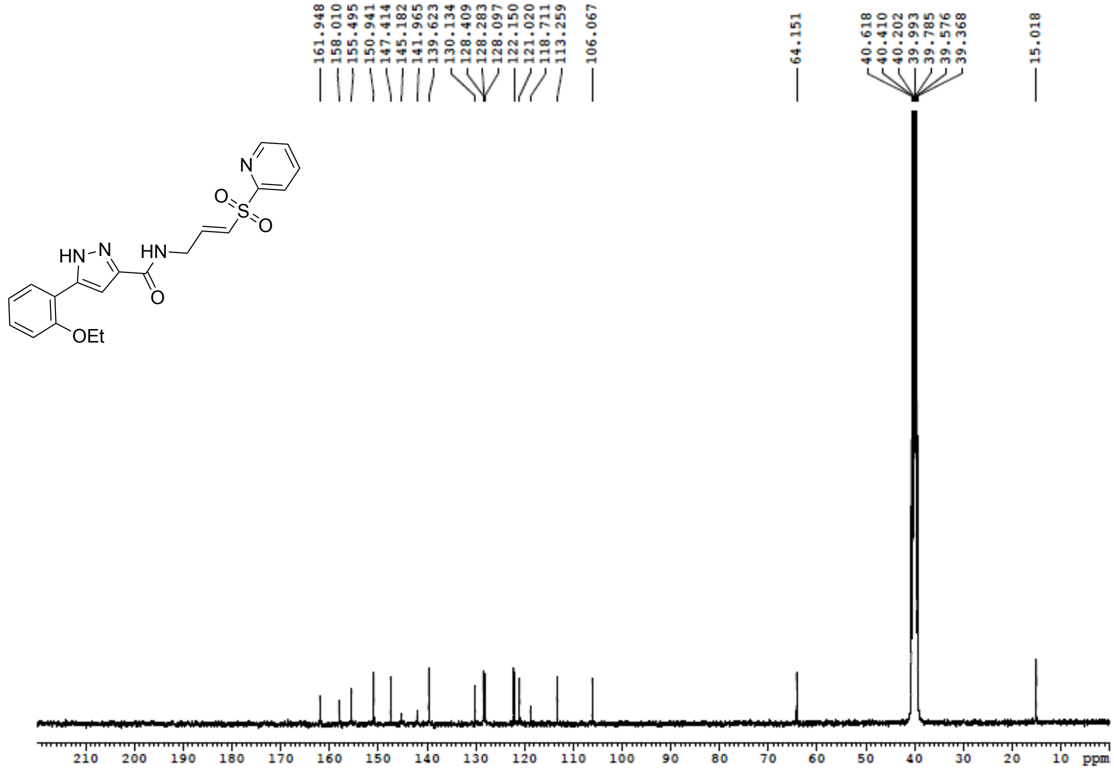

Figure S142: ^{1}H NMR (400 MHz, DMSO-d_6) for **26**

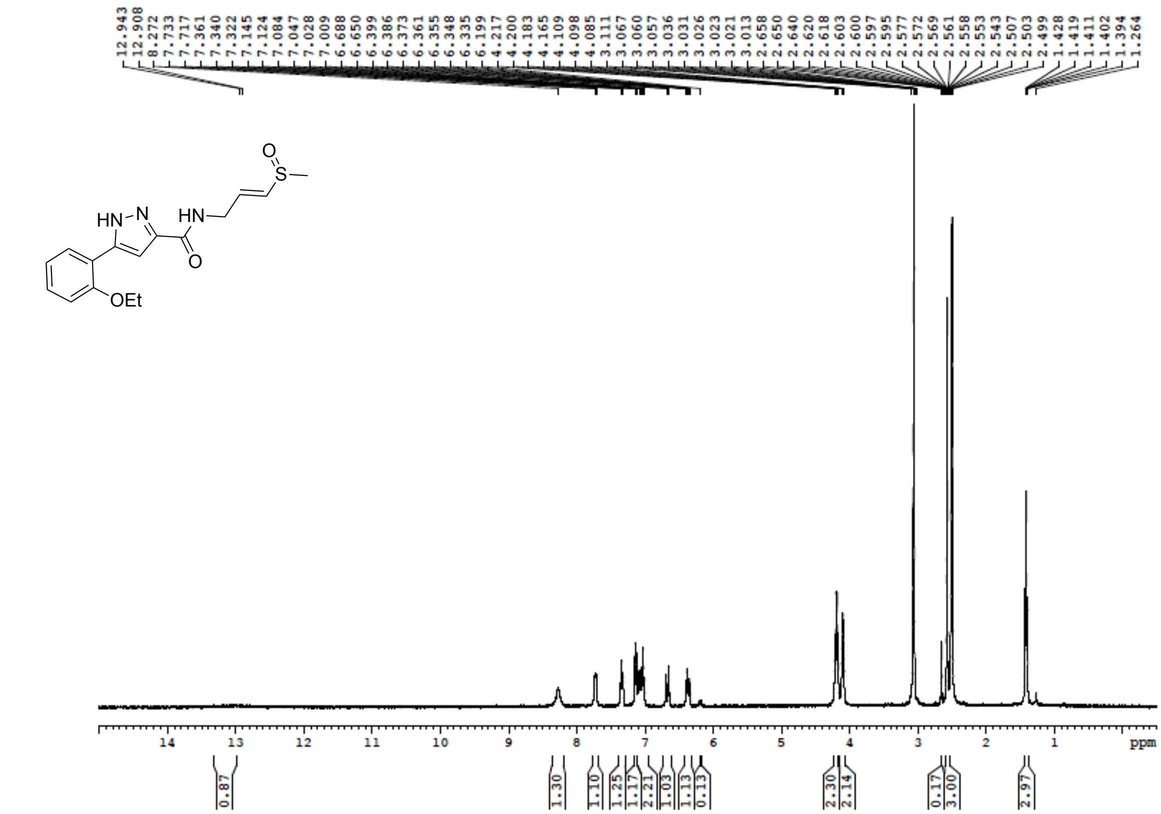

Figure S143: ^{13}C NMR (100 MHz, DMSO-d_6) for **26**

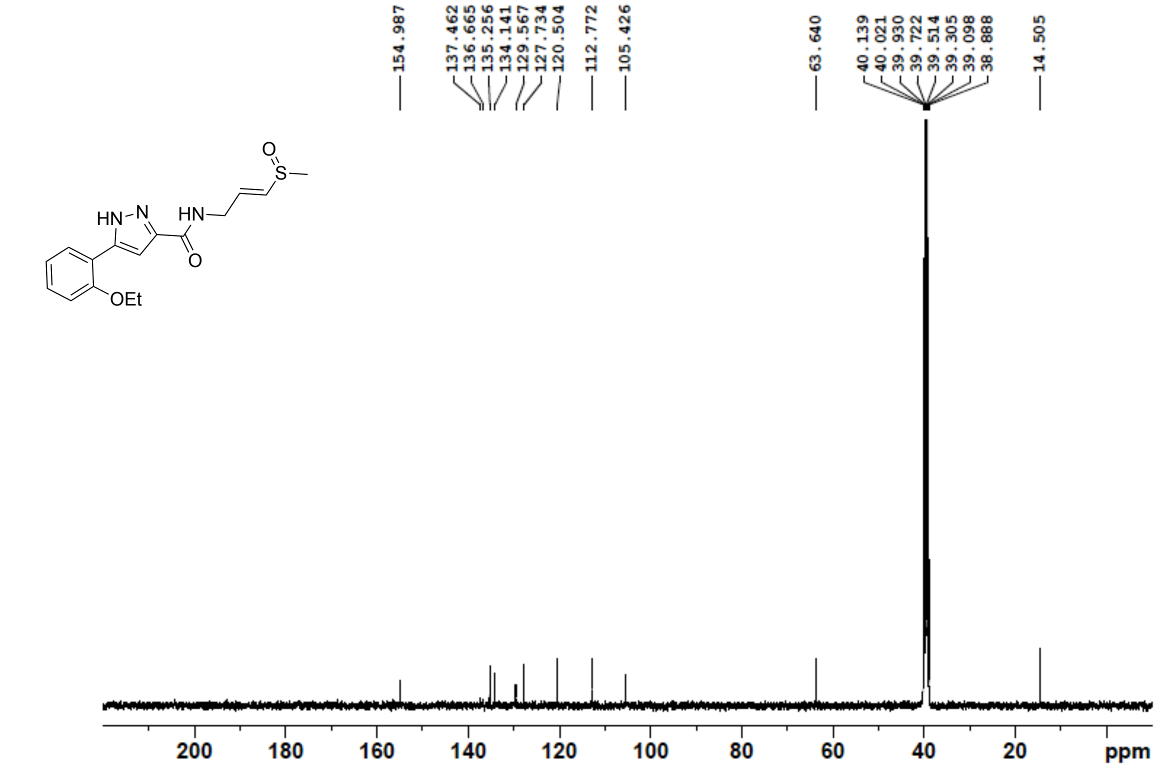

Figure S144: ^{1}H NMR (400 MHz, DMSO-d_6) for **27**

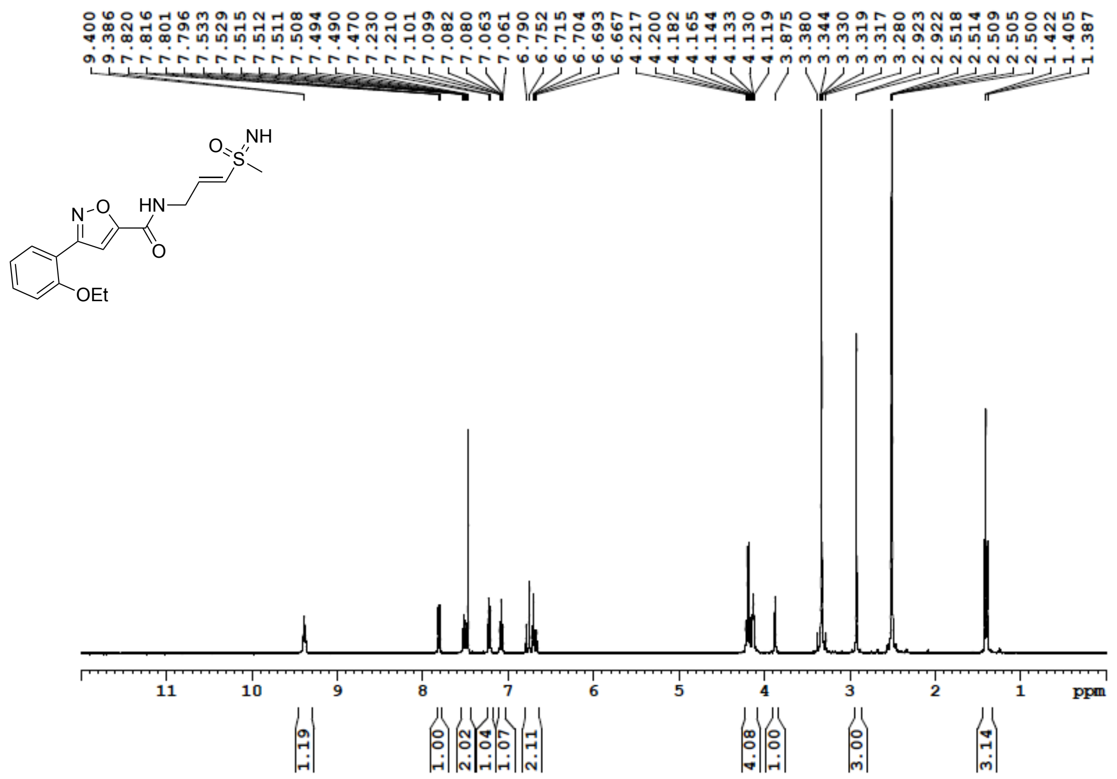

Figure S145: ^{13}C NMR (100 MHz, DMSO-d_6) for **27**

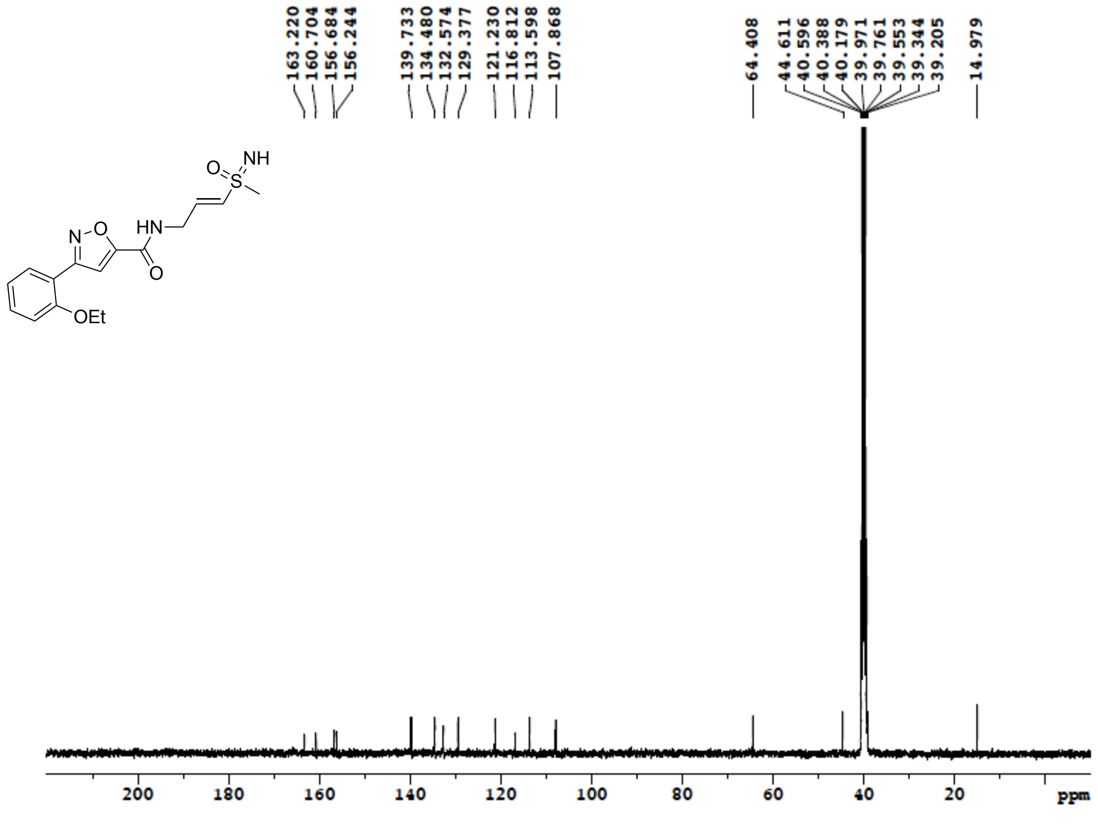

Figure S146: ^{1}H NMR (400 MHz, DMSO-d_6) for **28**

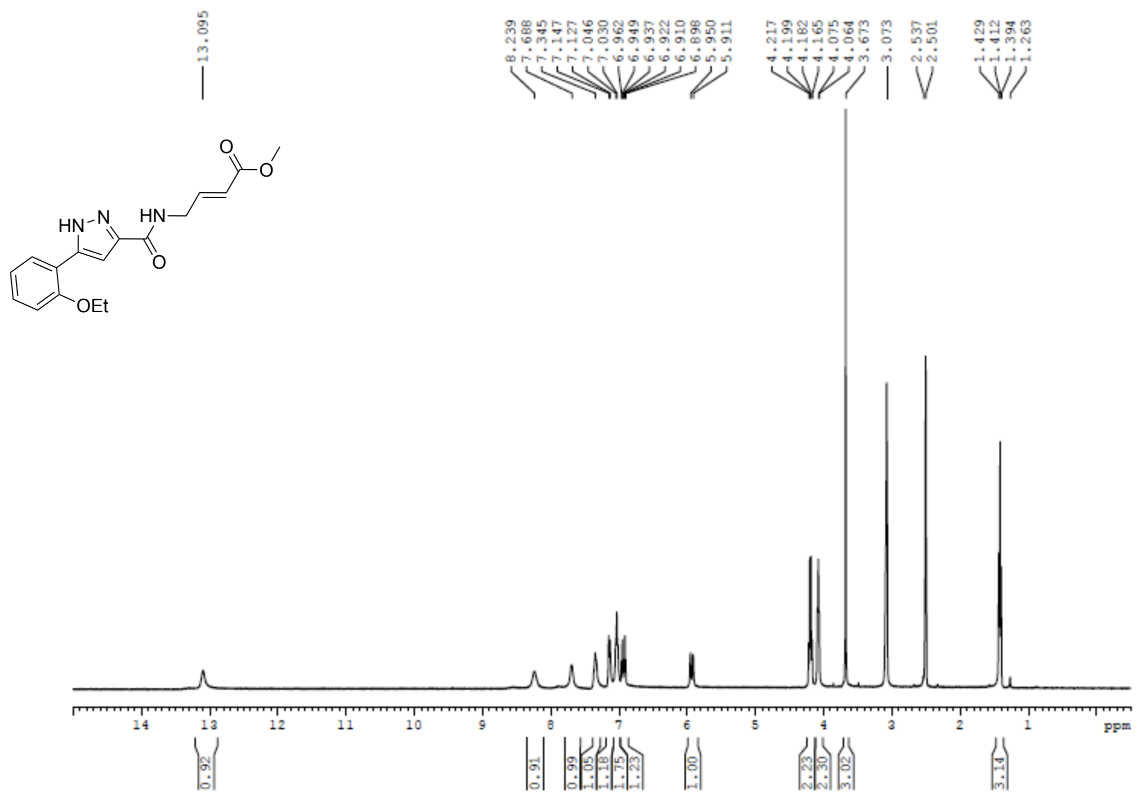

Figure S147: ^{13}C NMR (100 MHz, DMSO-d_6) for **28**

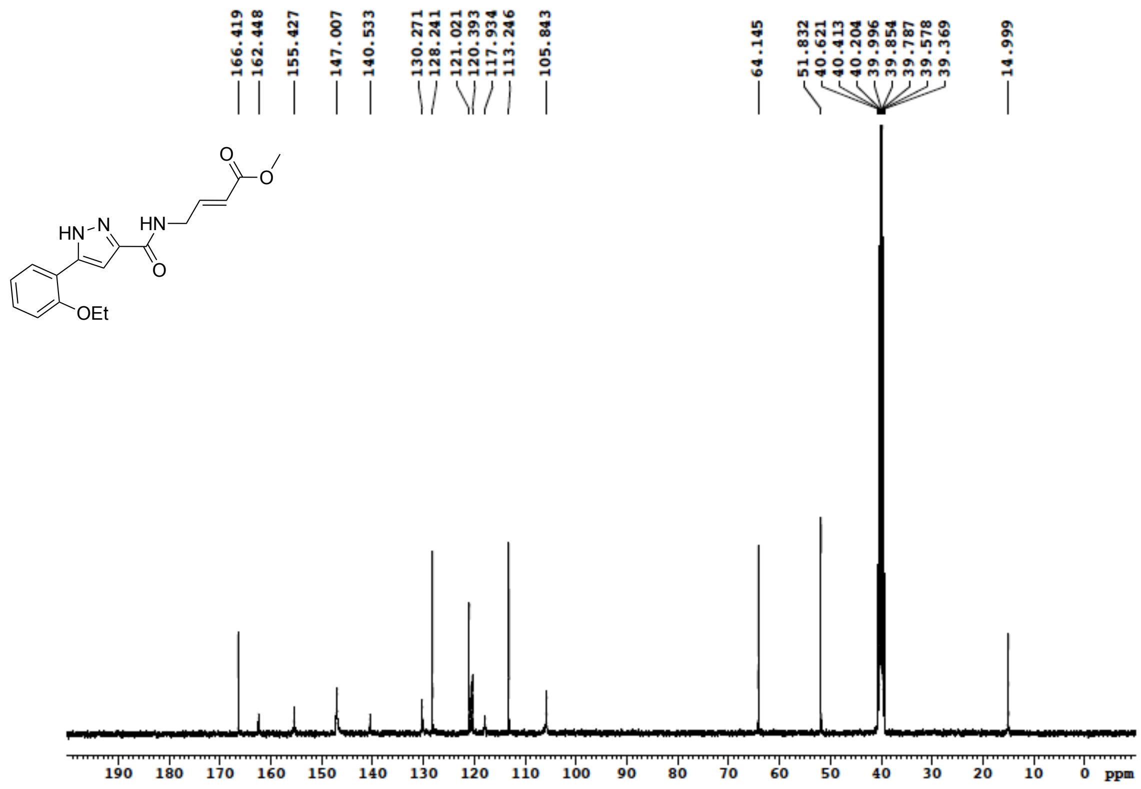

Figure S148: ^{1}H NMR (400 MHz, DMSO-d_6) for **29**

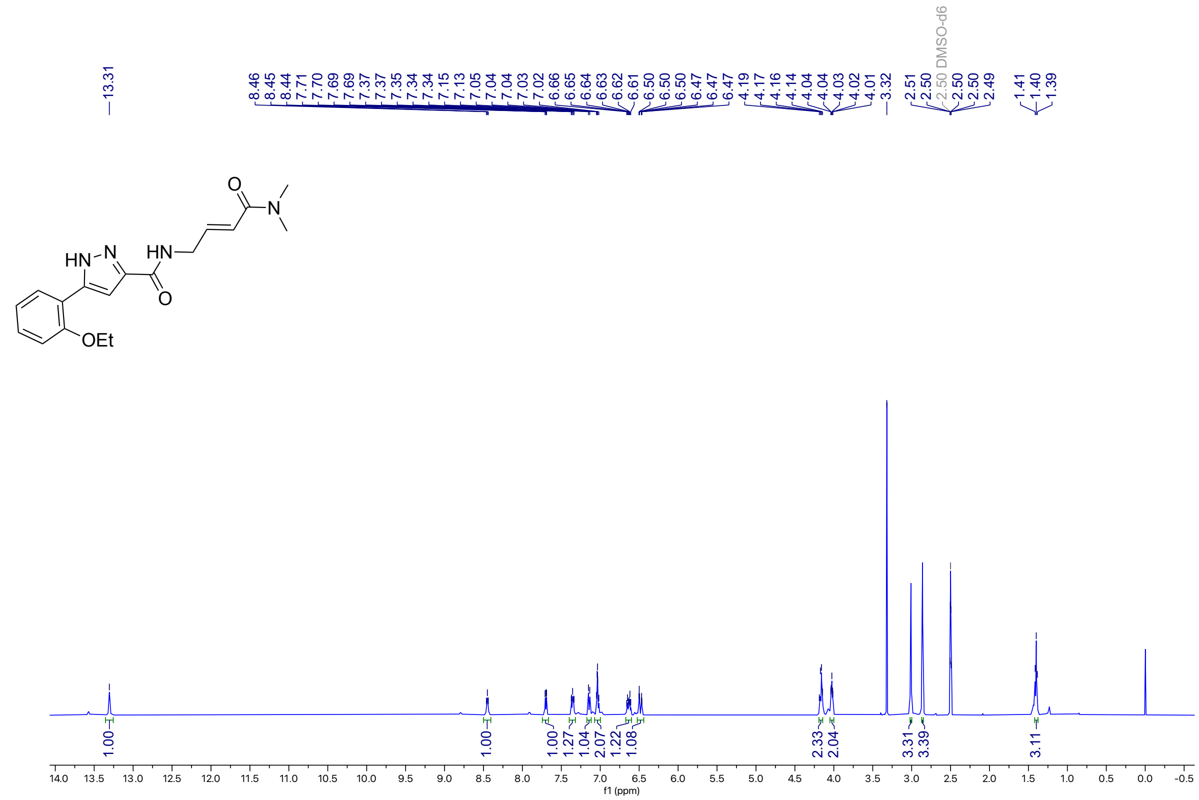

Figure S149: ^{13}C NMR (100 MHz, DMSO-d_6) for **29**

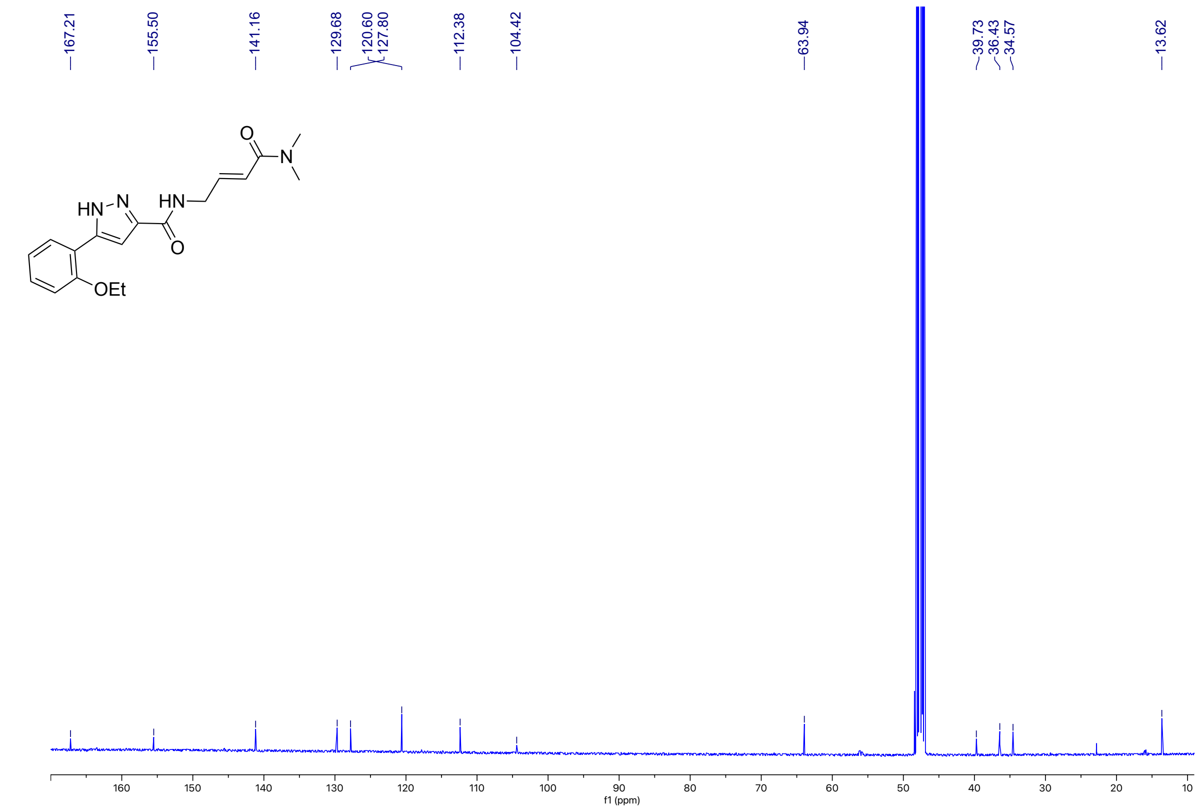

Figure S150: ^{1}H NMR (400 MHz, DMSO-d_6) for **30**

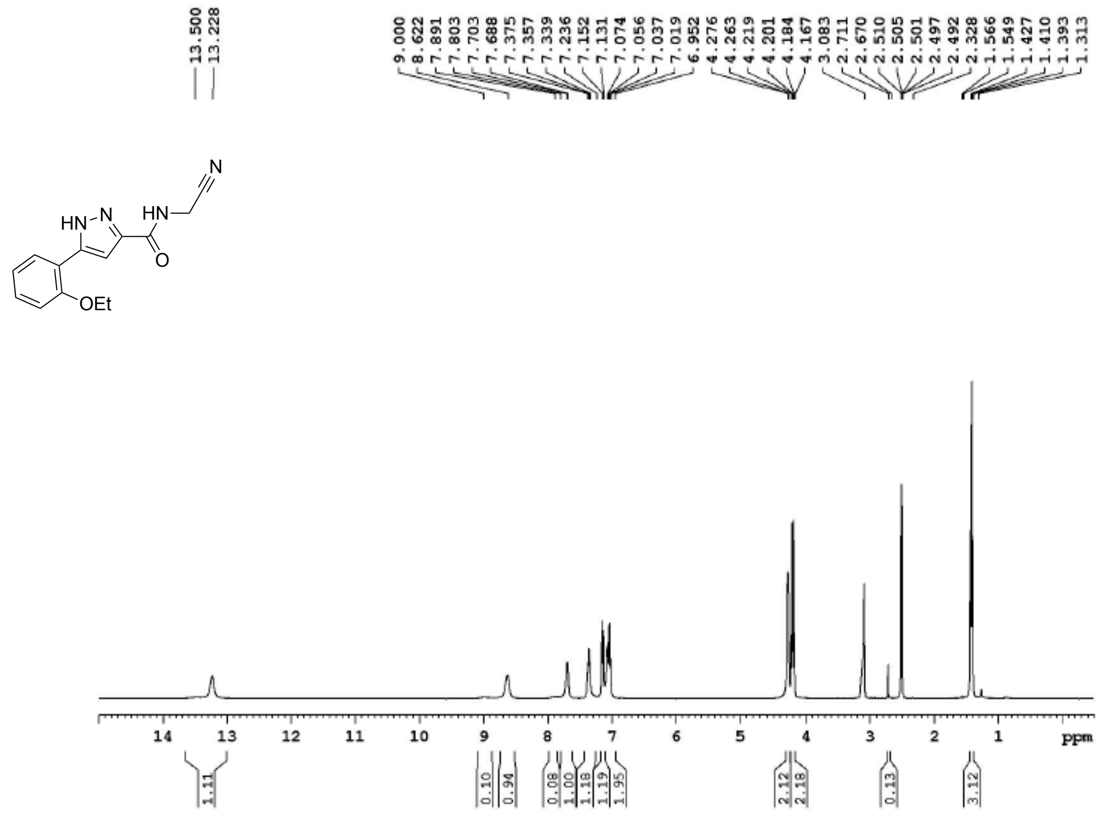

Figure S151: ^{13}C NMR (100 MHz, DMSO-d_6) for **30**

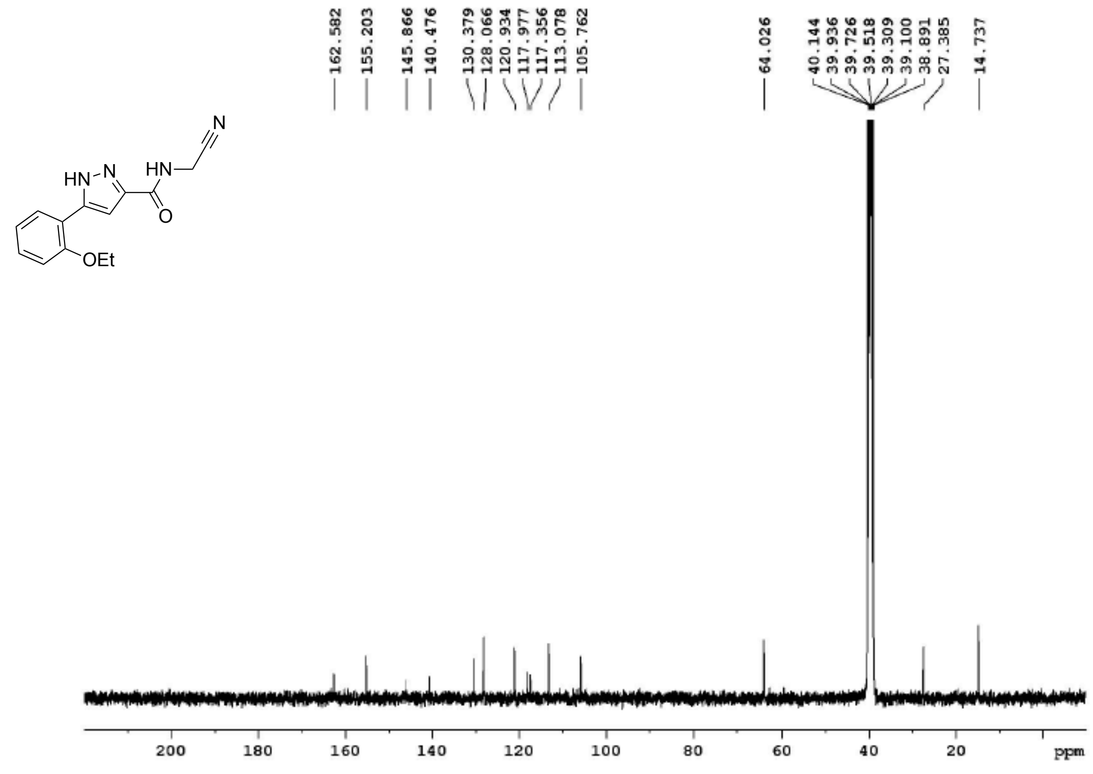

Figure S152: ^{1}H NMR (400 MHz, DMSO-d_6) for **31**

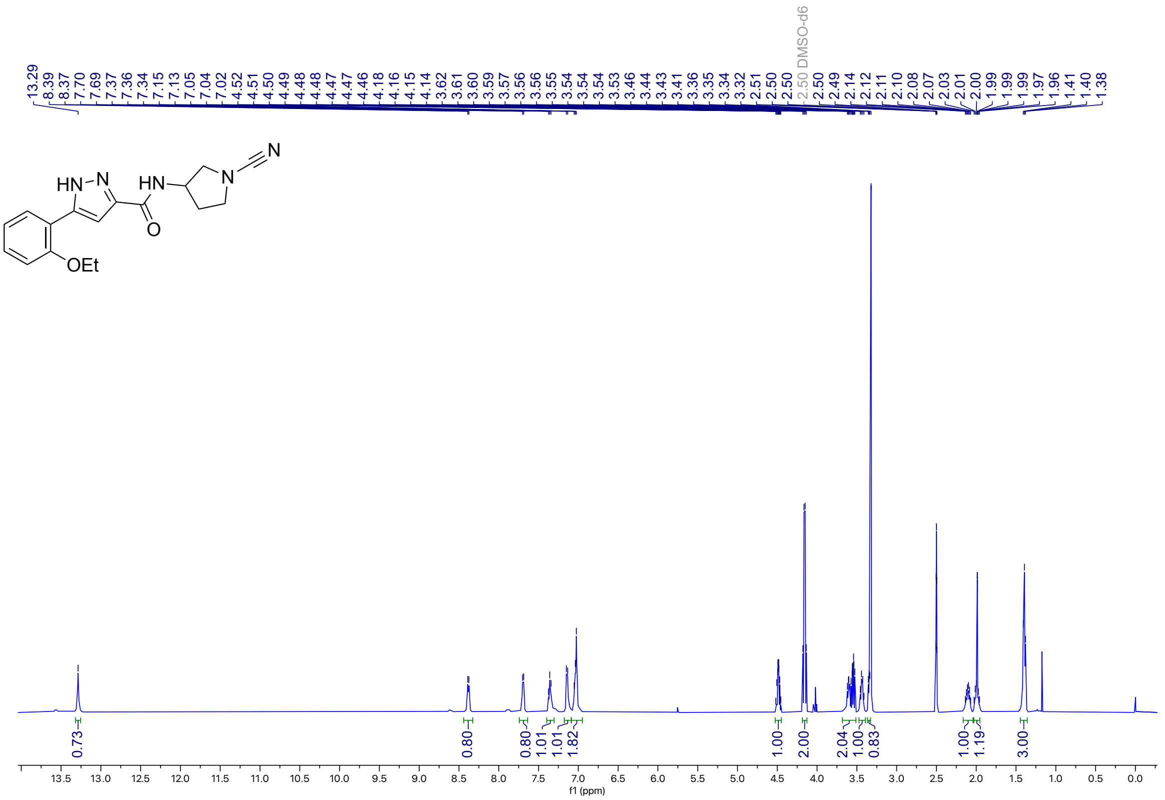

Figure S153: ^{13}C NMR (100 MHz, DMSO-d_6) for **31**

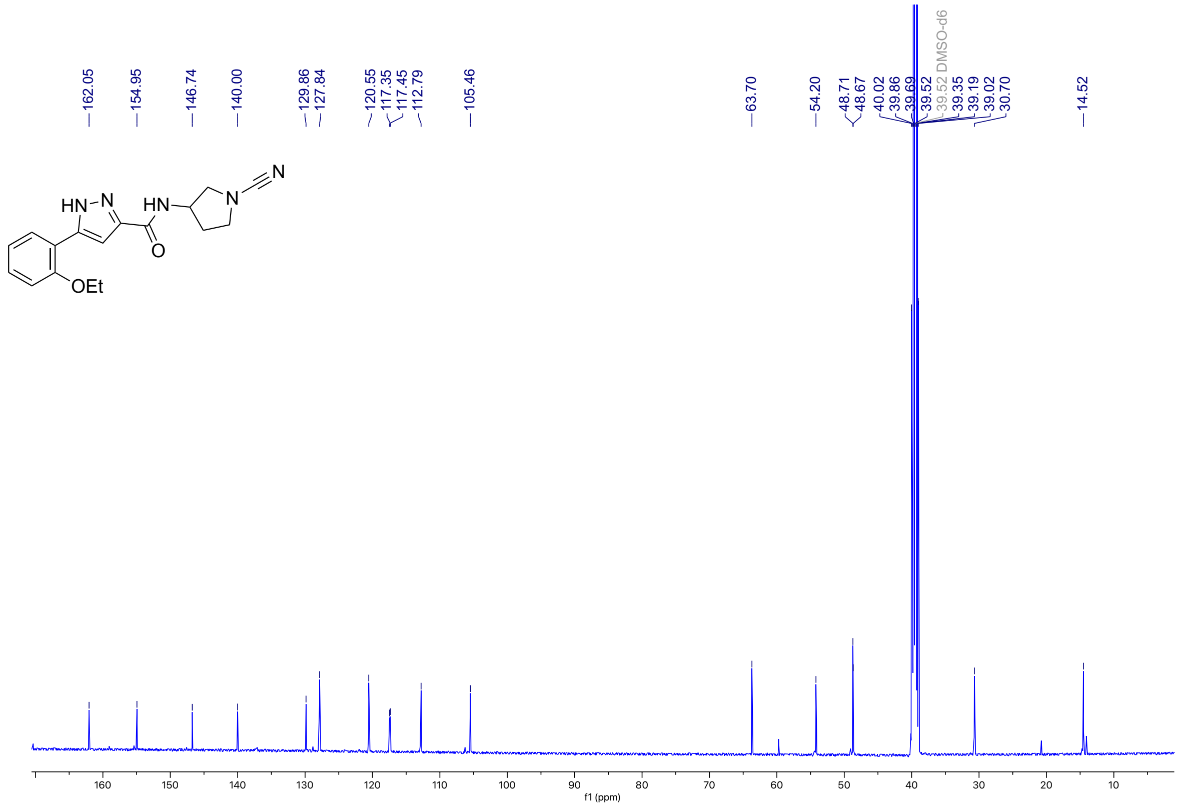

HPLC analysis of pyrazole analogs

Figure S154: HPLC trace of **1a**

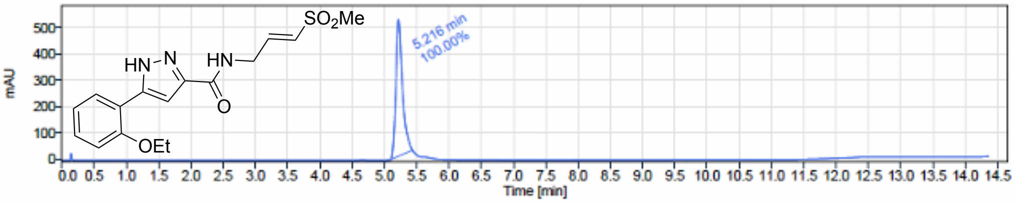

Signal Name DAD1A

RT (min)	Signal description	m/z	Purity UV(‰) / MS(%)	Area	Area%
5.216	DAD1A,Sig=254.0,5.0 Ref=360.0,80.0			3652.8	100.00

Figure S155: HPLC trace of **1b**

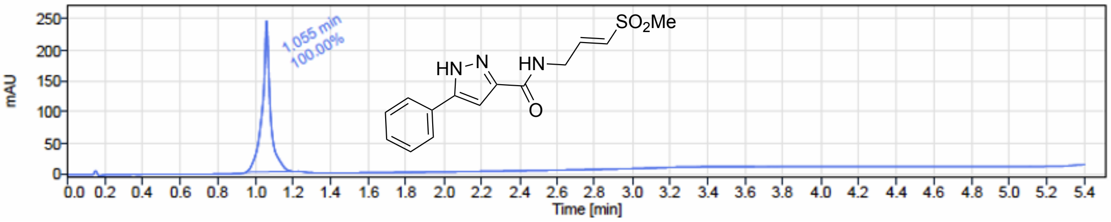

Signal Name DAD1A

RT (min)	Signal description	m/z	Purity UV(‰) / MS(%)	Area	Area%
1.055	DAD1A,Sig=254.0,5.0 Ref=360.0,80.0			734.1	100.00

Figure S156: HPLC trace of **1c**

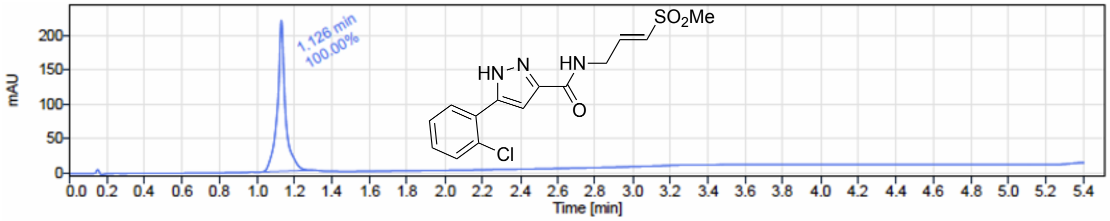

Signal Name DAD1A

RT (min)	Signal description	m/z	Purity UV(‰) / MS(%)	Area	Area%
1.126	DAD1A,Sig=254.0,5.0 Ref=360.0,80.0			642.7	100.00

Figure S157: HPLC trace of **1d**

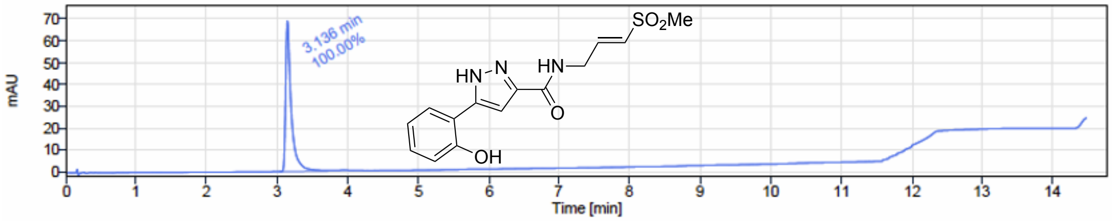

Signal Name DAD1A

RT (min)	Signal description	m/z	Purity UV(‰) / MS(%)	Area	Area%
3.136	DAD1A,Sig=254.0,5.0 Ref=360.0,80.0			378.6	100.00

Figure S158: HPLC trace of **1e**

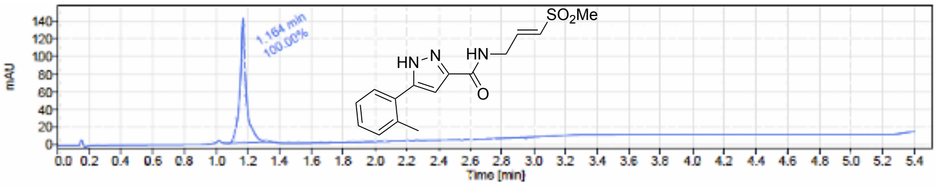

Signal Name	DAD1A				
RT (min)	Signal description	m/z	Purity UV(‰) / MS(%)	Area	Area%
1.164	DAD1A,Sig=254.0,5.0 Ref=360.0,80.0			395.4	100.00

Figure S159: HPLC trace of **1f**

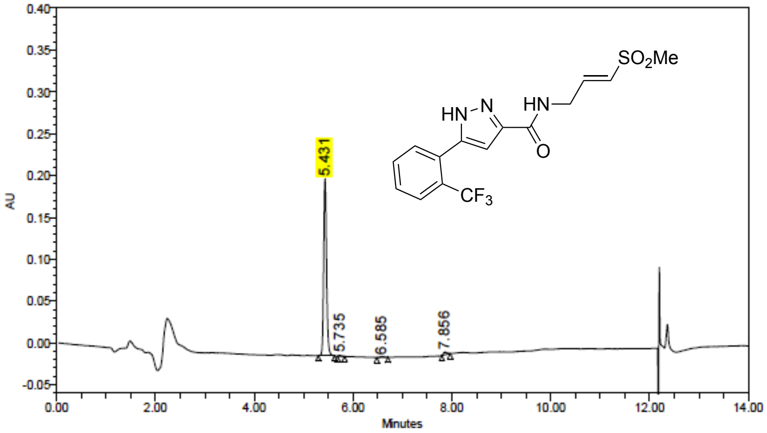

Peak Results

	Name	RT	Height (μV)	Area (μV*sec)	% Area
1		5.431	211624	855150	97.00
2		5.735	1387	5316	0.60
3		6.585	841	5208	0.59
4		7.856	3196	15943	1.81

Figure S160: HPLC trace of **1g**

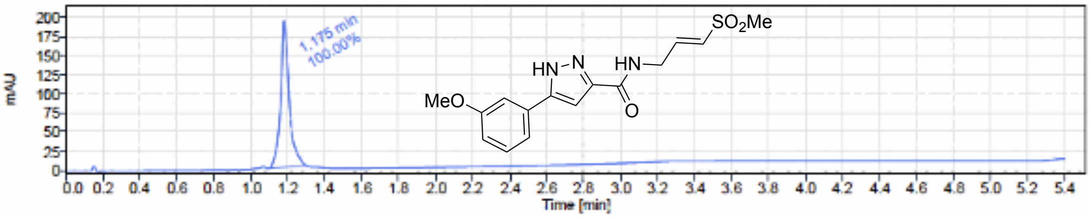

Signal Name	DAD1A				
RT (min)	Signal description	m/z	Purity UV(‰) / MS(%)	Area	Area%
1.175	DAD1A,Sig=254.0,5.0 Ref=360.0,80.0			614.4	100.00

Figure S161: HPLC trace of **1h**

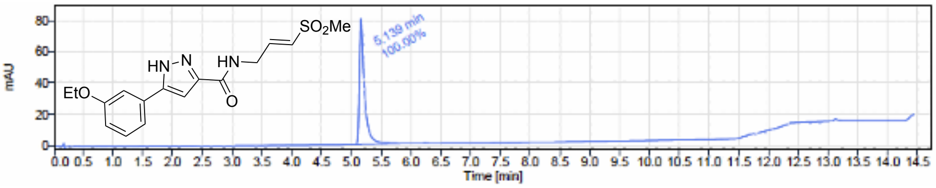

Signal Name DAD1A

RT (min)	Signal description	m/z	Purity UV(‰) / MS(%)	Area	Area%
5.139	DAD1A,Sig=254.0,5.0 Ref=360.0,80.0			477.5	100.00

Figure S162: HPLC trace of **1i**

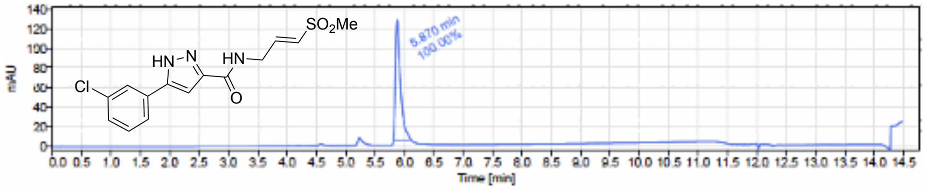

Signal Name DAD1A

RT (min)	Signal description	m/z	Purity UV(‰) / MS(%)	Area	Area%
5.870	* DAD1A,Sig=254.0,5.0 Ref=360.0,80.0 [b]			731.1	100.00

Figure S163: HPLC trace of **1j**

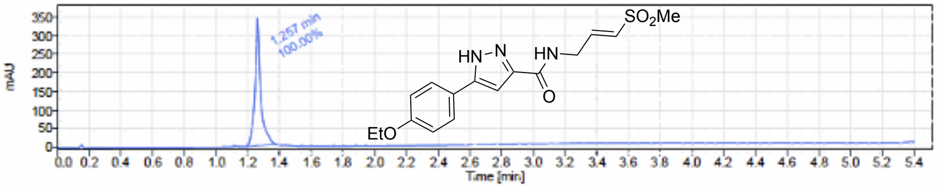

Signal Name DAD1A

RT (min)	Signal description	m/z	Purity UV(‰) / MS(%)	Area	Area%
1.257	DAD1A,Sig=254.0,5.0 Ref=360.0,80.0			878.5	100.00

Figure S164: HPLC trace of **1k**

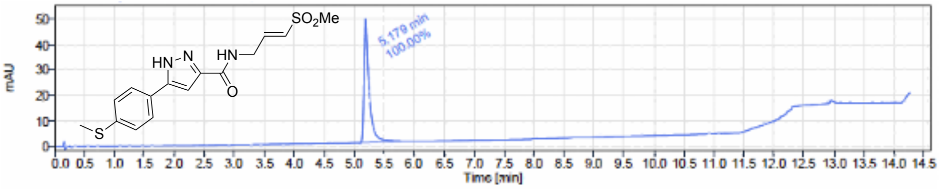

Signal Name DAD1A

RT (min)	Signal description	m/z	Purity UV(‰) / MS(%)	Area	Area%
5.179	DAD1A,Sig=254.0,5.0 Ref=360.0,80.0			290.4	100.00

Figure S165: HPLC trace of **1l**

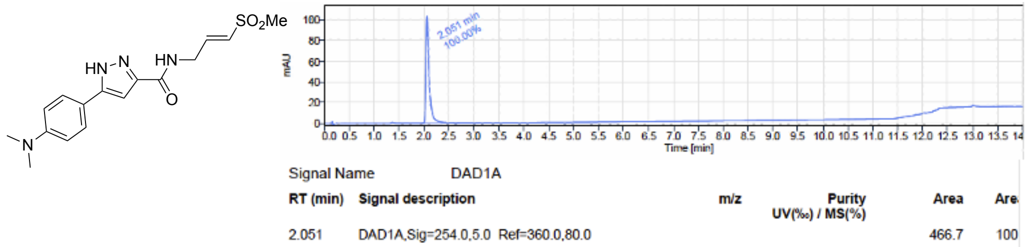

Signal Name DAD1A

RT (min)	Signal description	m/z	Purity UV(‰) / MS(%)	Area	Are
2.051	DAD1A,Sig=254.0,5.0 Ref=360.0,80.0			466.7	100

Figure S166: HPLC trace of **1m**

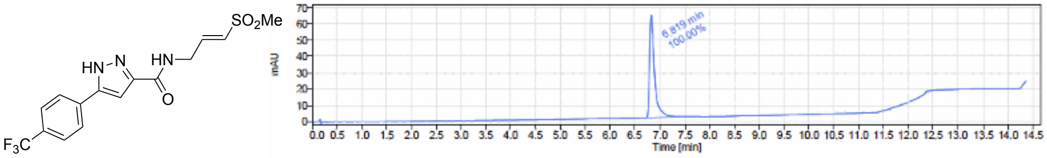

Signal Name DAD1A

RT (min)	Signal description	m/z	Purity UV(‰) / MS(%)	Area	Area%
6.819	DAD1A,Sig=254.0,5.0 Ref=360.0,80.0			439.1	100.00

Figure S167: HPLC trace of **1n**

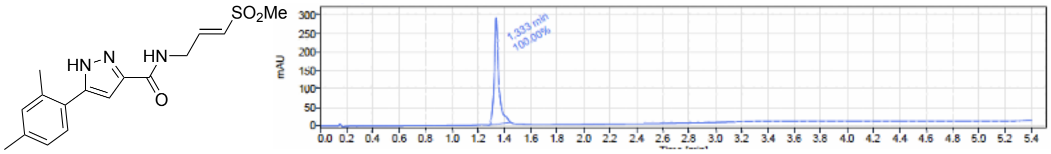

Signal Name DAD1A

RT (min)	Signal description	m/z	Purity UV(‰) / MS(%)	Area	Area%
1.333	DAD1A,Sig=254.0,5.0 Ref=360.0,80.0			684.7	100.00

Figure S168: HPLC trace of **1o**

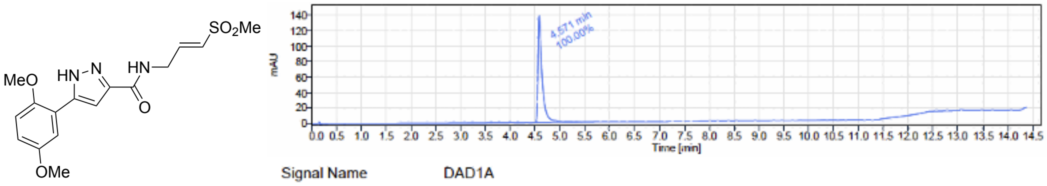

Signal Name DAD1A

RT (min)	Signal description	m/z	Purity UV(‰) / MS(%)	Area	Area%
4.571	DAD1A,Sig=254.0,5.0 Ref=360.0,80.0			798.8	100.00

Figure S169: HPLC trace of **1p**

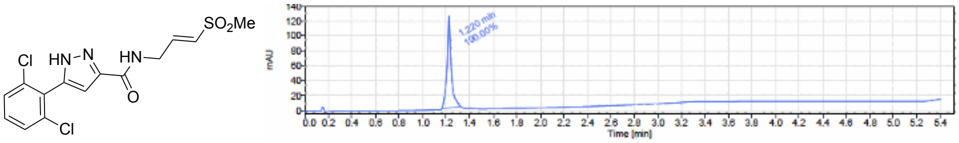

Signal Name DAD1A

RT (min)	Signal description	m/z	Purity UV(‰) / MS(%)	Area	Area%
1.220	DAD1A,Sig=254.0,5.0 Ref=360.0,80.0			307.5	100.00

Figure S170: HPLC trace of **1q**

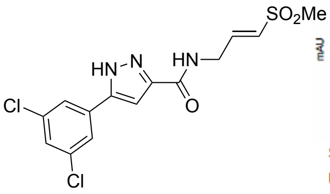

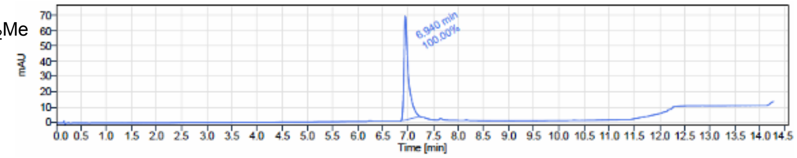

Signal Name DAD1A

RT (min)	Signal description	m/z	Purity UV(‰) / MS(%)	Area	Area%
6.940	DAD1A,Sig=254.0,5.0 Ref=360.0,80.0			420.4	100.00

Figure S171: HPLC trace of **2**

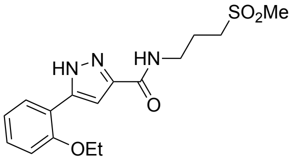

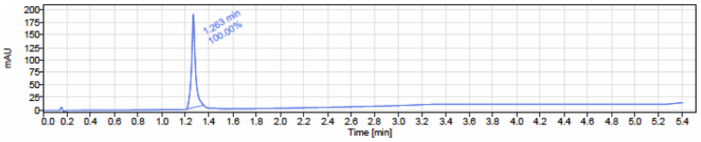

Signal Name DAD1A

RT (min)	Signal description	m/z	Purity UV(‰) / MS(%)	Area	Area%
1.263	DAD1A,Sig=254.0,5.0 Ref=360.0,80.0			431.7	100.00

Figure S172: HPLC trace of **3**

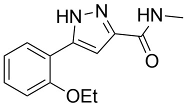

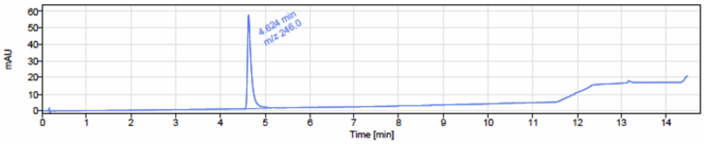

Signal Name DAD1A

RT (min)	Signal description	m/z	Purity UV(‰) / MS(%)	Area	Area%
4.624	DAD1A,Sig=254.0,5.0 Ref=360.0,80.0	246.0		339.8	100.00

Figure S173: HPLC trace of **4a**

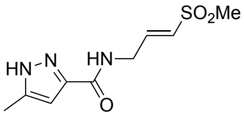

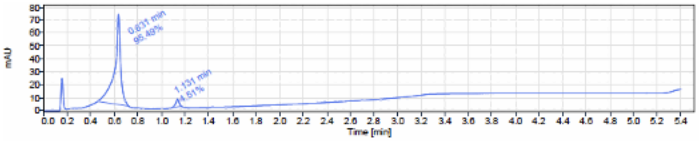

Signal Name DAD1A

RT (min)	Signal description	m/z	Purity UV(‰) / MS(%)	Area	Area%
0.631	DAD1A,Sig=254.0,5.0 Ref=360.0,80.0			252.5	95.49
1.131	DAD1A,Sig=254.0,5.0 Ref=360.0,80.0			11.9	4.51

Figure S174: HPLC trace of **4b**

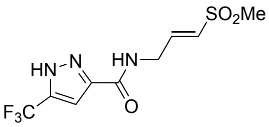

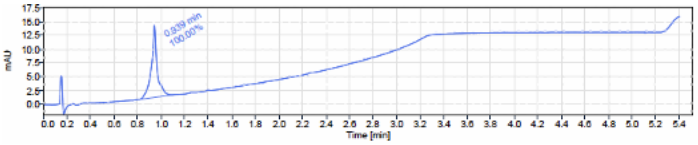

Signal Name DAD1A

RT (min)	Signal description	m/z	Purity UV(‰) / MS(%)	Area	Area%
0.939	DAD1A,Sig=254.0,5.0 Ref=360.0,80.0			45.0	100.00

Figure S175: HPLC trace of **4c**

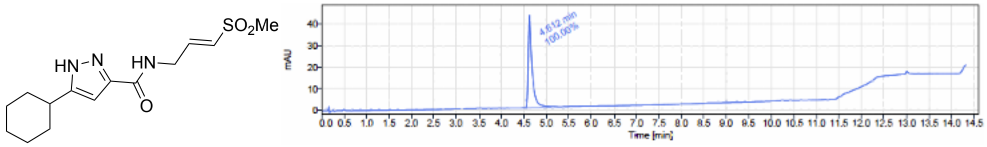

Signal Name	DAD1A				
RT (min)	Signal description	m/z	Purity UV(‰) / MS(%)	Area	Area%
4.612	DAD1A,Sig=254.0,5.0 Ref=360.0,80.0			255.1	100.00

Figure S176: HPLC trace of **4d**

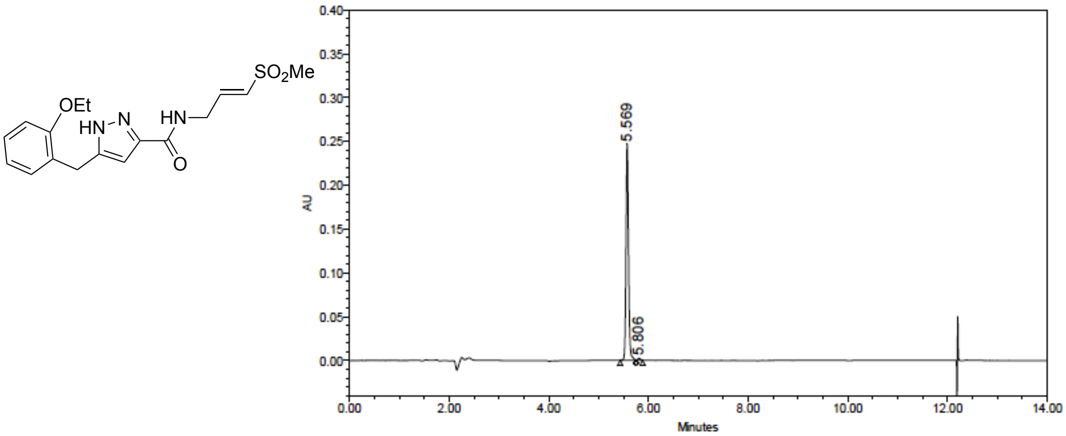

Peak Results

	Name	RT	Height (μV)	Area (μV*sec)	% Area
1		5.569	247581	863325	98.95
2		5.806	2444	9195	1.05

Figure S177: HPLC trace of **4e**

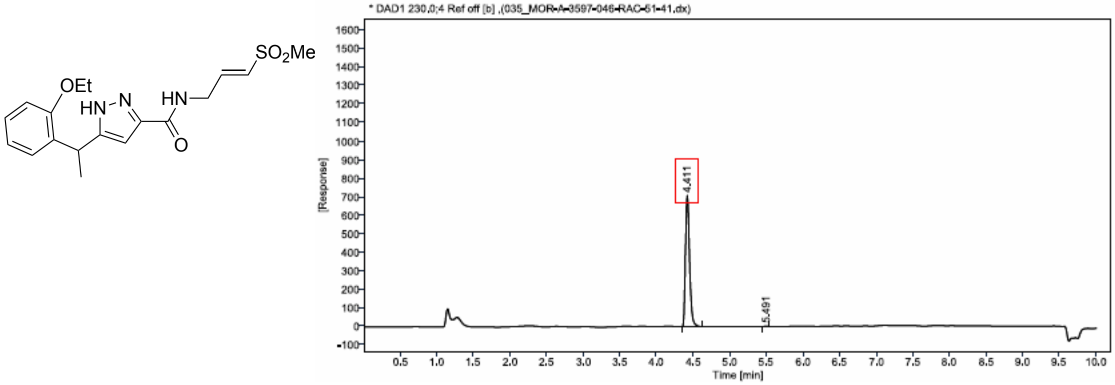

Sr.No.	RT	Area	Height	% Area
1	4,41	2712	705	99,90
2	5,49	3	1	0,10

Figure S178: HPLC trace of **4f**

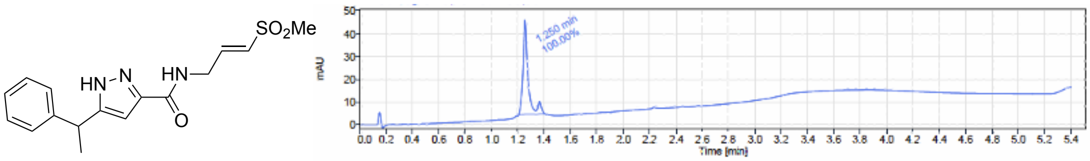

Signal Name	DAD1A				
RT (min)	Signal description	m/z	Purity UV(‰) / MS(%)	Area	Area%
1.250	DAD1A,Sig=254.0,5.0 Ref=360.0,80.0			112.1	100.00

Figure S179: HPLC trace of **4g**

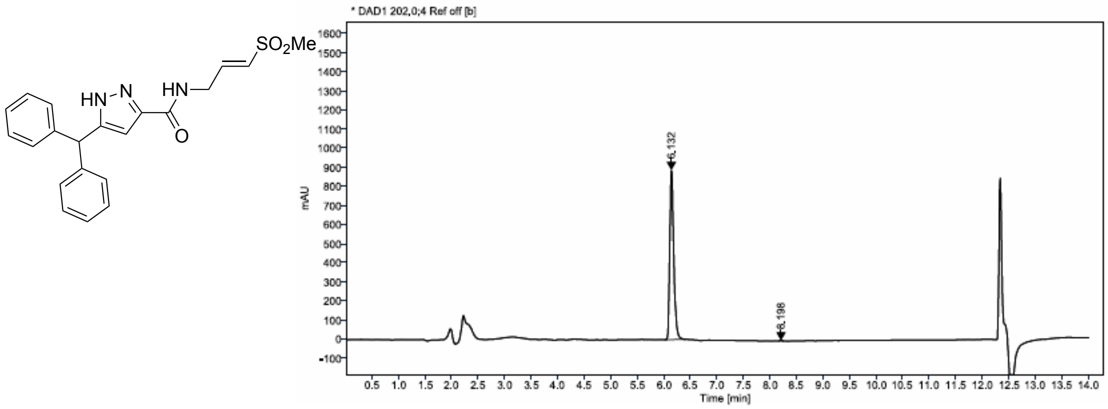

Sr.No.	RT	Area	Height	% Area
1	6.13	4622	888	99.86
2	8.20	6	2	0.14

Figure S180: HPLC trace of **4h**

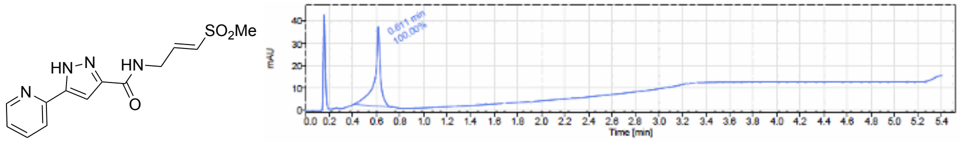

Signal Name	DAD1A				
RT (min)	Signal description	m/z	Purity UV(‰) / MS(%)	Area	Area%
0.611	DAD1A,Sig=254.0,5.0 Ref=360.0,80.0			125.2	100.00

Figure S181: HPLC trace of **4i**

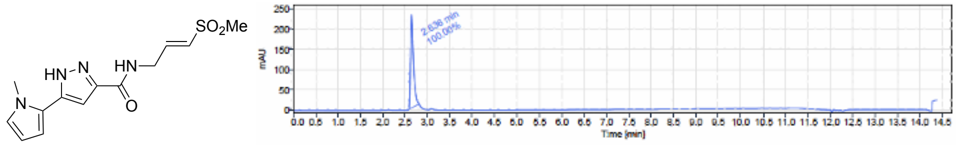

Signal Name	DAD1A				
RT (min)	Signal description	m/z	Purity UV(‰) / MS(%)	Area	Area%
2.636	* DAD1A,Sig=254.0,5.0 Ref=360.0,80.0 [b]			1065.7	100.00

Figure S182: HPLC trace of **5**

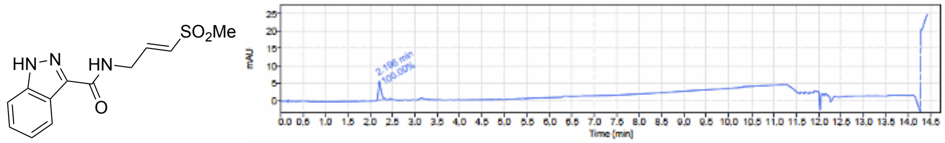

Signal Name DAD1A

RT (min)	Signal description	m/z	Purity UV(‰) / MS(%)	Area	Area%
2.196	* DAD1A,Sig=254.0,5.0 Ref=360.0,80.0 [b]			23.3	100.00

Figure S183: HPLC trace of **6**

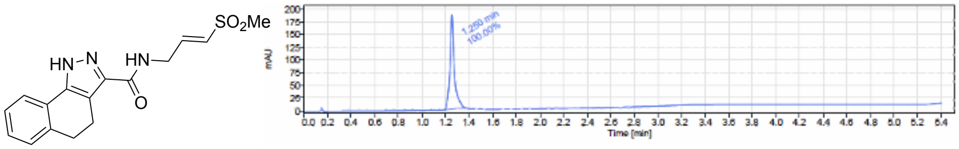

Signal Name DAD1A

RT (min)	Signal description	m/z	Purity UV(‰) / MS(%)	Area	Area%
1.250	DAD1A,Sig=254.0,5.0 Ref=360.0,80.0			474.0	100.00

Figure S184: HPLC trace of **7a**

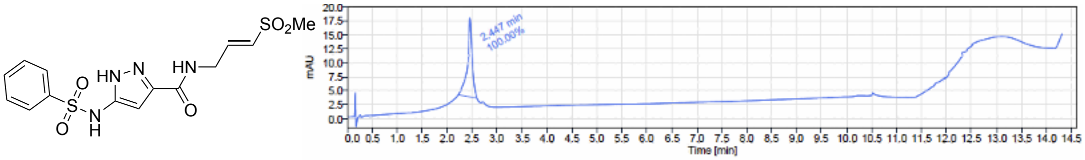

Signal Name DAD1A

RT (min)	Signal description	m/z	Purity UV(‰) / MS(%)	Area	Area%
2.447	DAD1A,Sig=254.0,5.0 Ref=360.0,80.0			94.0	100.00

Figure S185: HPLC trace of **7b**

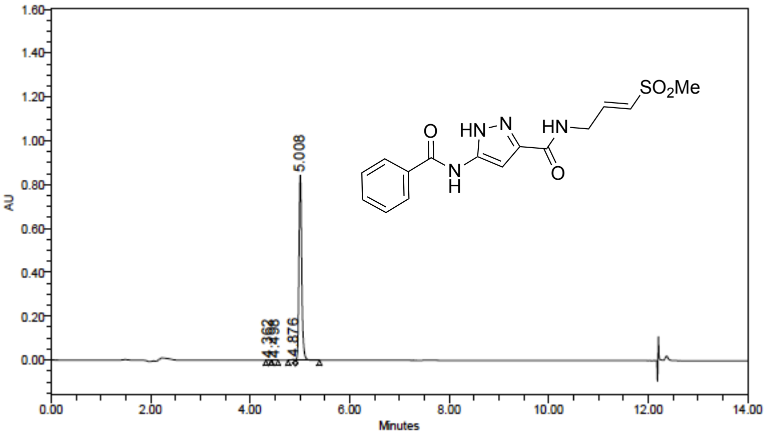

Peak Results

	Name	RT	Height (µV)	Area (µV*sec)	% Area
1		4.362	787	2309	0.07
2		4.498	732	2633	0.08
3		4.876	4643	21707	0.66
4		5.008	843836	3277199	99.19

Figure S186: HPLC trace of **7c**

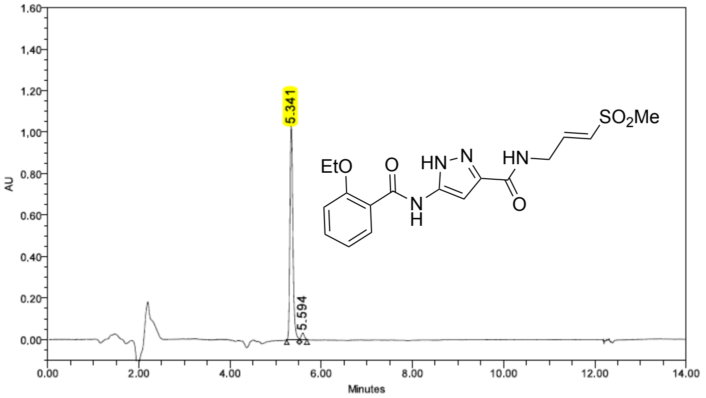

Peak Results

	Name	RT	Height (µV)	Area (µV*sec)	% Area
1		5,341	1028710	4618285	97,00
2		5,594	30827	142877	3,00

Figure S187: HPLC trace of **7d**

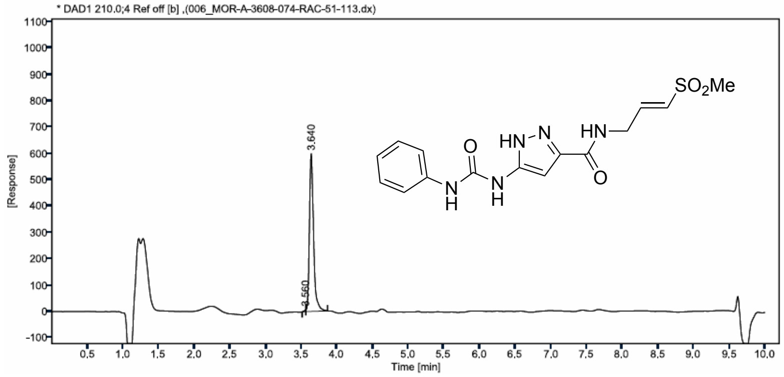

Sr.No.	RT	Area	Height	% Area
1	3.56	9	7	0.36
2	3.64	2435	598	99.64

Figure S188: HPLC trace of **7e**

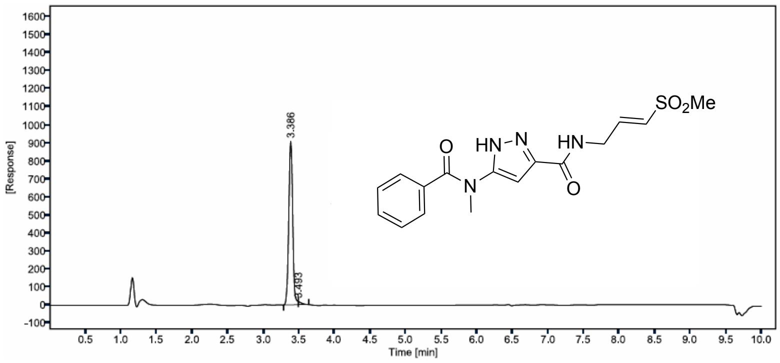

Sr.No.	RT	Area	Height	% Area
1	3.39	3486	906	98.47
2	3.49	54	18	1.53

Figure S189: HPLC trace of **7f**

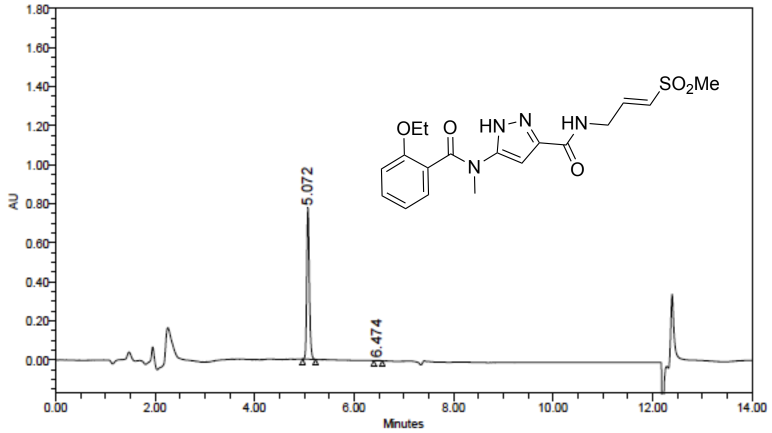

Peak Results

	Name	RT	Height (µV)	Area (µV*sec)	% Area
1		5.072	775596	2834909	99.63
2		6.474	2035	10557	0.37

Figure S190: HPLC trace of **8a**

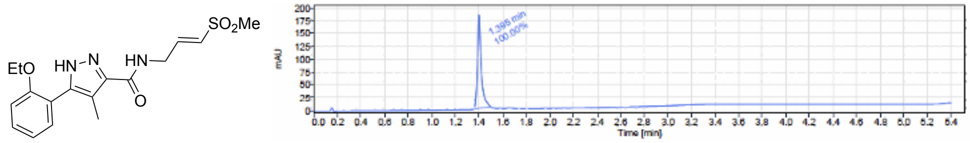

Signal Name DAD1A

RT (min)	Signal description	m/z	Purity UV(‰) / MS(%)	Area	Area%
1.395	DAD1A,Sig=254.0,5.0 Ref=360.0,80.0			408.6	100.00

Figure S191: HPLC trace of **8b**

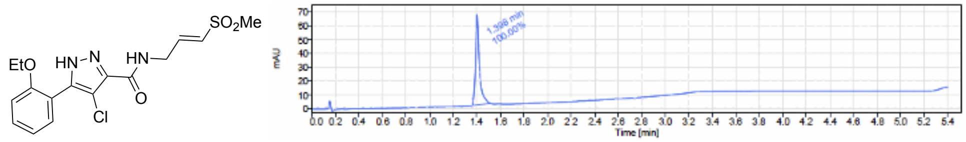

Signal Name DAD1A

RT (min)	Signal description	m/z	Purity UV(‰) / MS(%)	Area	Area%
1.398	DAD1A,Sig=254.0,5.0 Ref=360.0,80.0			150.6	100.00

Figure S192: HPLC trace of **8c**

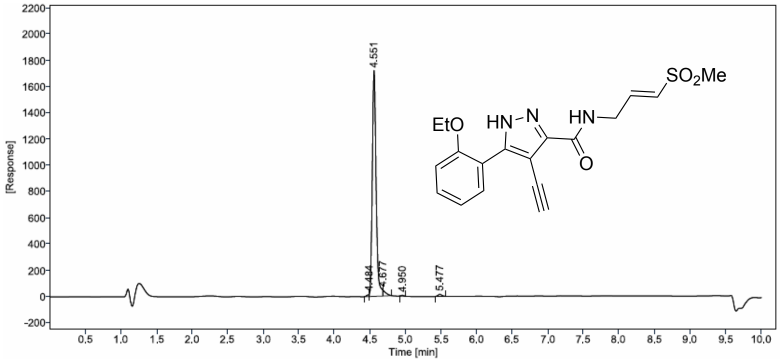

Sr.No.	RT	Area	Height	% Area
1	4.48	32	16	0.48
2	4,55	6471	1716	96,48
3	4.68	128	45	1.91
4	4.95	18	6	0.26
5	5,48	58	16	0,87

Figure S193: HPLC trace of **8d**

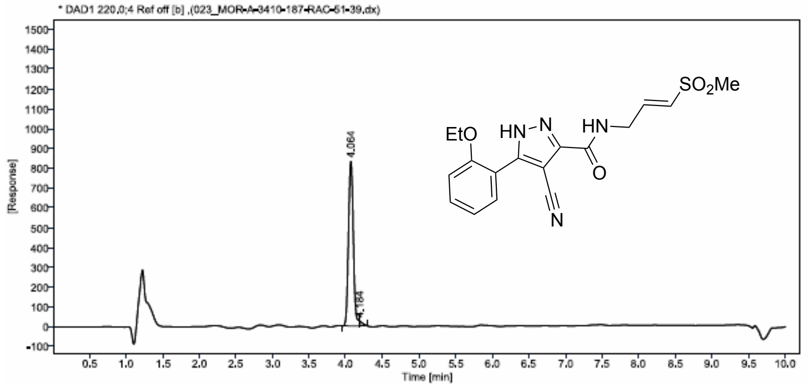

Sr.No.	RT	Area	Height	% Area
1	4,06	3792	831	98,33
2	4,18	64	24	1,67

Figure S194: HPLC trace of **24a**

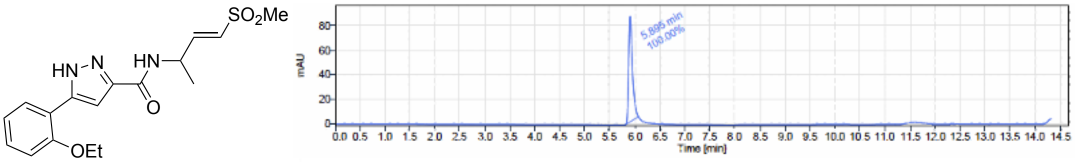

Signal Name DAD1A

RT (min)	Signal description	m/z	Purity UV(‰) / MS(%)	Area	Area%
5.895	* DAD1A,Sig=254.0,5.0 Ref=360.0,80.0 [b]			454.4	100.00

Figure S195: HPLC trace of **24b**

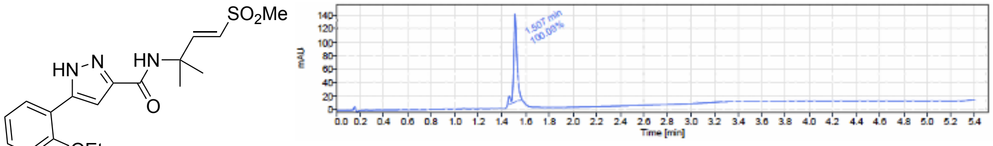

Signal Name DAD1A

RT (min)	Signal description	m/z	Purity UV(‰) / MS(%)	Area	Area%
1.507	DAD1A,Sig=254.0,5.0 Ref=360.0,80.0			258.8	100.00

Figure S196: HPLC trace of **24c**

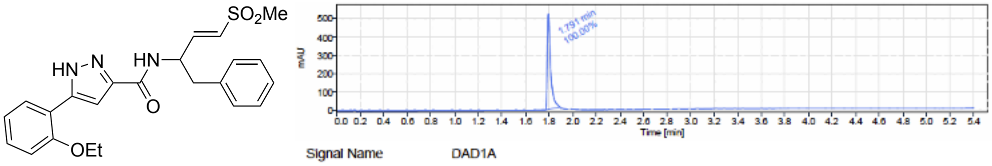

Signal Name DAD1A

RT (min)	Signal description	m/z	Purity UV(‰) / MS(%)	Area	Area%
1.791	DAD1A,Sig=254.0,5.0 Ref=360.0,80.0			1014.0	100.00

Figure S197: HPLC trace of **24d**

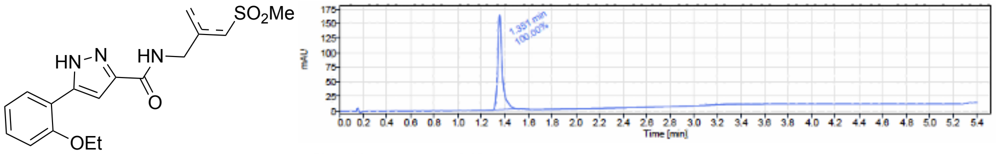

Signal Name DAD1A

RT (min)	Signal description	m/z	Purity UV(‰) / MS(%)	Area	Area%
1.351	DAD1A,Sig=254.0,5.0 Ref=360.0,80.0			432.6	100.00

Figure S198: HPLC trace of **24e**

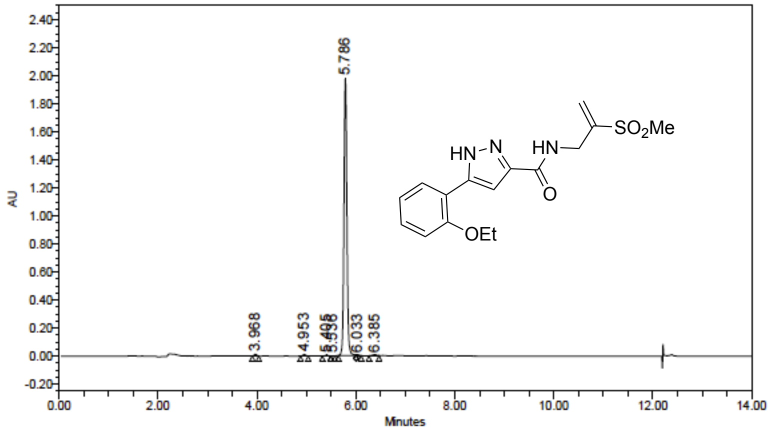

Peak Results

	Name	RT	Height (μV)	Area (μV*sec)	% Area
1		3.968	12629	43316	0.52
2		4.953	11227	40908	0.49
3		5.405	5007	18787	0.23
4		5.536	719	1895	0.02
5		5.786	1979110	8113963	97.85
6		6.033	7091	27342	0.33
7		6.385	7974	46424	0.56

Figure S199: HPLC trace of **24f**

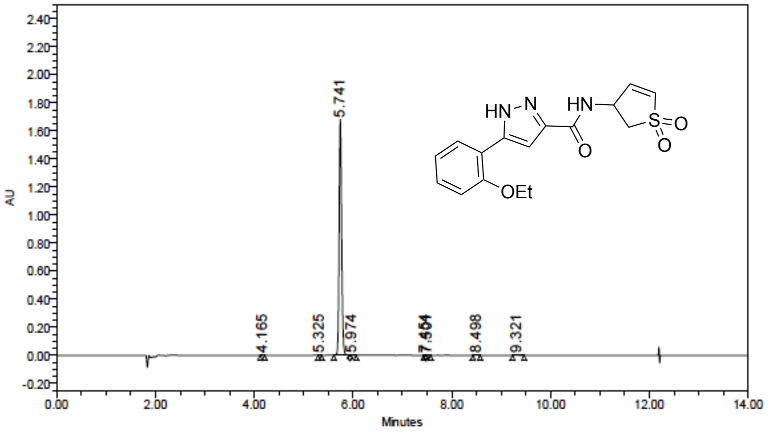

Peak Results

	Name	RT	Height (µV)	Area (µV*sec)	% Area
1		4.165	1627	4408	0.07
2		5.325	605	1471	0.02
3		5.741	1678516	5999346	99.74
4		5.974	1001	3939	0.07
5		7.454	200	416	0.01
6		7.501	292	858	0.01
7		8.498	602	2221	0.04
8		9.321	418	2103	0.03

Figure S200: HPLC trace of **25a**

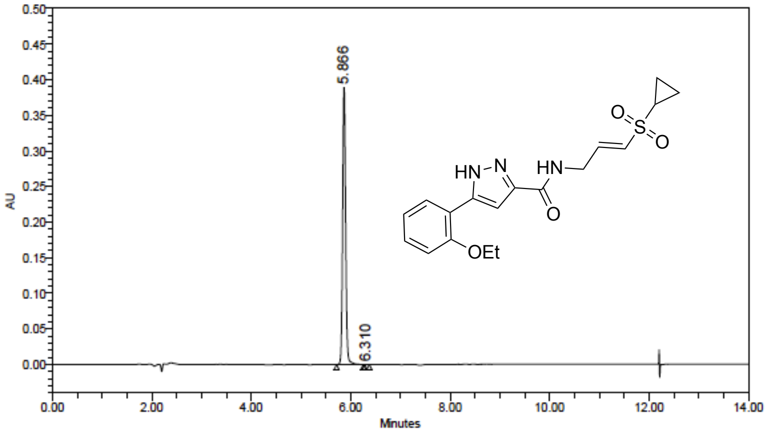

Peak Results

	Name	RT	Height (µV)	Area (µV*sec)	% Area
1		5.866	389753	1626082	99.99
2		6.310	67	231	0.01

Figure S201: HPLC trace of **25b**

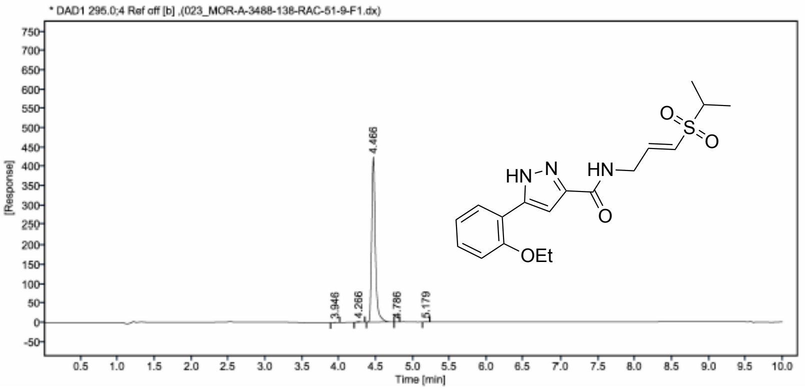

Sr.No.	RT	Area	Height	% Area
1	3.95	2	1	0.16
2	4.27	6	2	0.39
3	4.47	1486	424	99.25
4	4.79	1	0	0.07
5	5.18	2	1	0.12

Figure S202: HPLC trace of **25c**

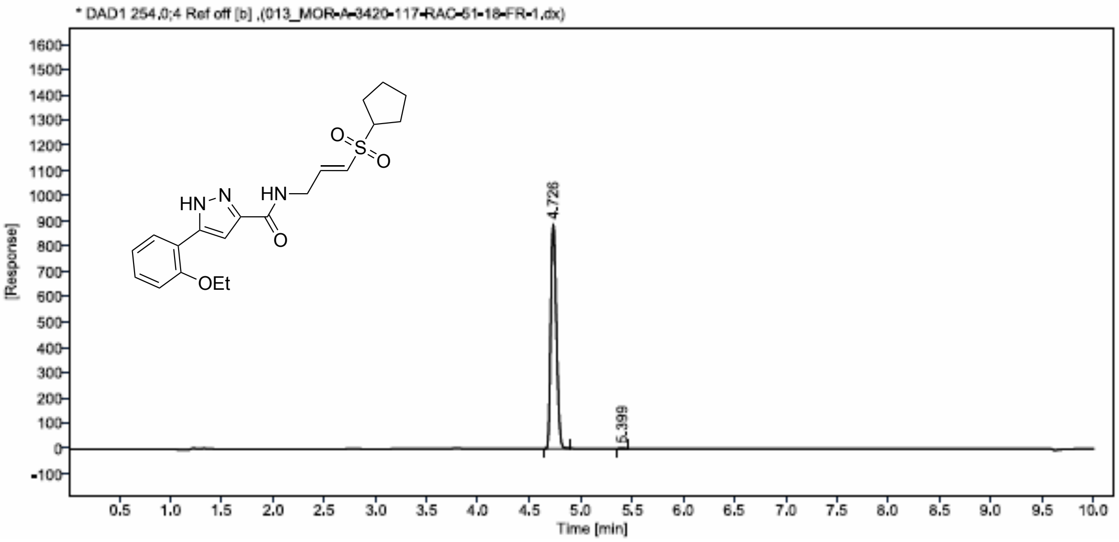

Sr.No.	RT	Area	Height	% Area
1	4,73	3383	889	99,81
2	5,40	7	2	0,19

Figure S203: HPLC trace of **25d**

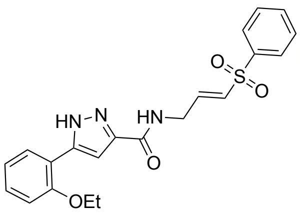

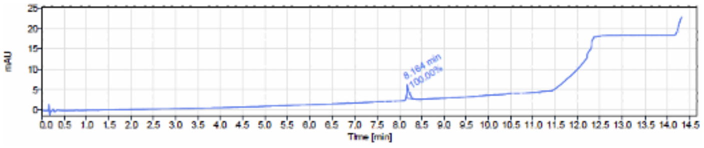

Signal Name DAD1A

RT (min)	Signal description	m/z	Purity UV(%) / MS(%)	Area	Area%
8.164	DAD1A,Sig=254.0,5.0 Ref=360.0,80.0			11.4	100.00

Figure S204: HPLC trace of **25e**

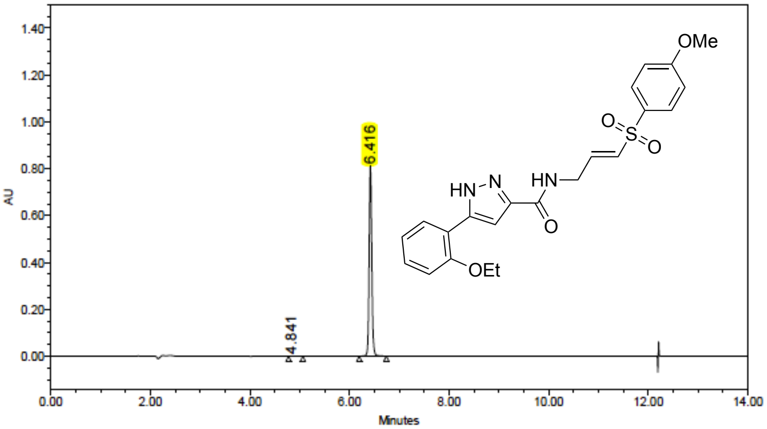

Peak Results

	Name	RT	Height (μV)	Area (μV*sec)	% Area
1		4.841	107	925	0.03
2		6.416	810051	3004935	99.97

Figure S205: HPLC trace of **25f**

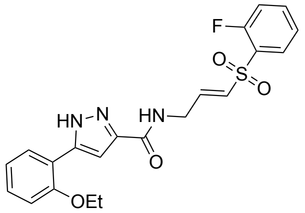

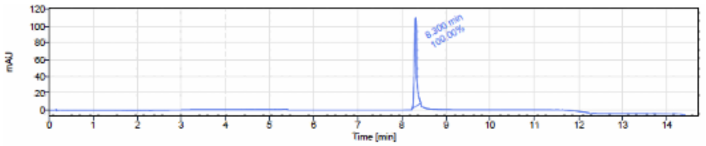

Signal Name DAD1A

RT (min)	Signal description	m/z	Purity UV(%) / MS(%)	Area	Area%
8.300	* DAD1A,Sig=254.0,5.0 Ref=360.0,80.0 [b]			399.6	100.00

Figure S206: HPLC trace of **25g**

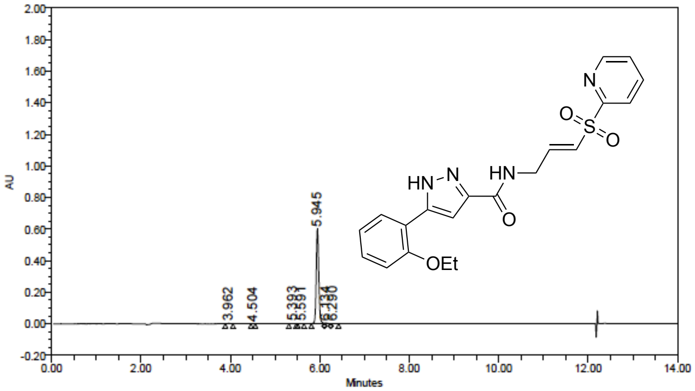

Peak Results

	Name	RT	Height (μV)	Area (μV*sec)	% Area
1		3.962	2116	7405	0.31
2		4.504	386	992	0.04
3		5.393	813	2776	0.12
4		5.591	693	2328	0.10
5		5.945	599754	2359373	98.53
6		6.134	3328	16267	0.68
7		6.290	1024	5466	0.23

Figure S207: Chiral SFC trace of (*R*)-**24a**

Sample	Sample Well	Inj. Vol.(ul)
MOR-A-3543-191-RAC-51-99-FR-01	2,5,A	12

File Name	Instrument Method	Inj. Time
MOR-A-3543-191-RAC-51-99-FR-01_1.tta	04_SFC_05_100	11-03-2024 17:40:13

Flow(ml/min)	Co-Solvent% Values	Back Pressure	Col. Temp. (C)	Report Date
4.00	5.0	100 Bar	40	11-03-2024
4.00	50.0			
4.00	50.0			

Column	Co-Solvent
CHIRALPAK IH (250*4.6mm, 5um)	0.1%NH3 IN IPA:ACN (50:50)

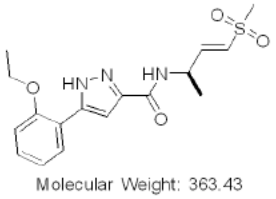

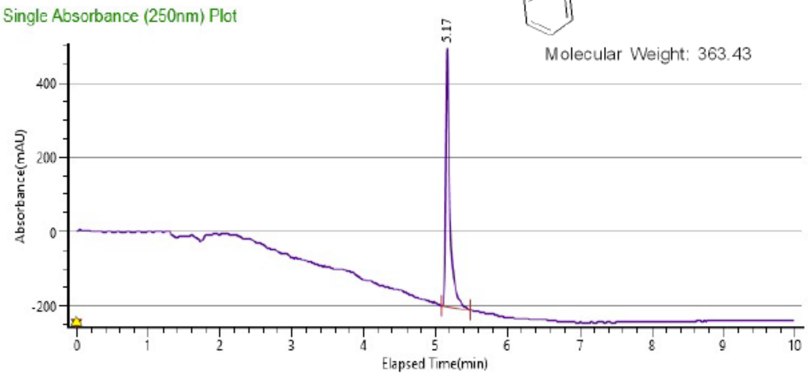

Peak Info[(W2998)SingleAbsorbance250]

Peak #	Ret. Time	Area	Height	Area %
1	5.17 min	2738.3965	693.9703	100.0000

Figure S208: Chiral SFC trace of (*S*)-**24a**

Sample	Sample Well	Inj. Vol.(ul)
MOR-A-3543-192-RAC-51-99-FR-02	2,6,A	12

File Name	Instrument Method	Inj. Time
MOR-A-3543-192-RAC-51-99-FR-02_1.tta	04_SFC_05_100	11-03-2024 17:53:23

Flow(ml/min)	Co-Solvent% Values	Back Pressure	Col. Temp. (C)	Report Date
4.00	5.0	99 Bar	40	11-03-2024
4.00	50.0			
4.00	50.0			

Column	Co-Solvent
CHIRALPAK IH (250*4.6mm, 5um)	0.1%NH3 IN IPA:ACN (50:50)

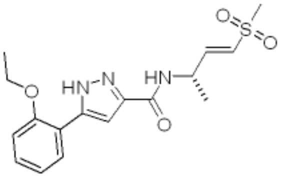

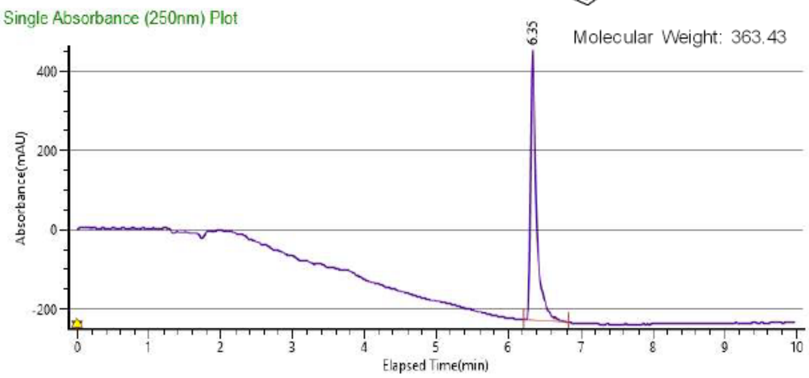

Peak Info[(W2998)SingleAbsorbance250]

Peak #	Ret. Time	Area	Height	Area %
1	6.35 min	3880.3869	680.3875	100.0000
